# Symmetrical Phosphinic Acids: Synthesis and Esterification Optimization toward Potential HIV Prodrugs

**DOI:** 10.1021/acsomega.4c05988

**Published:** 2024-09-27

**Authors:** Komal Hayat, Gemma Nixon, Qian Zhang, Magdalini Matziari

**Affiliations:** † Department of Chemistry, School of Science, Xi’an Jiaotong-Liverpool University, 111 Ren’ai Road, SIP, Suzhou, Jiangsu Province 215123, P. R. China; ‡ Department of Chemistry, University of Liverpool, Crown Street, Liverpool L69 7ZD, U.K.

## Abstract

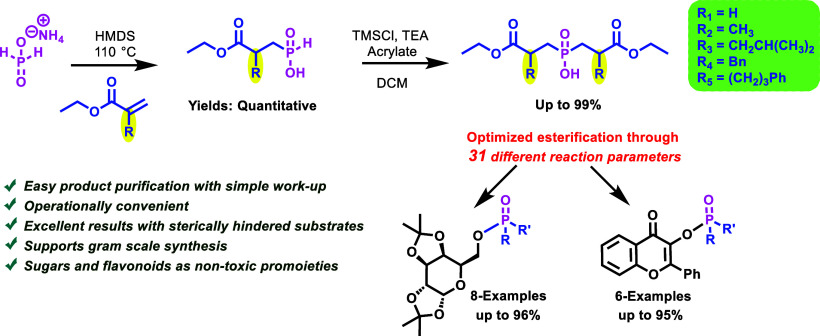

A highly efficient method to synthesize diverse symmetrical phosphinic acids with the potential to act as pivotal candidates in the design of HIV-1 protease inhibitors has been developed. Such compounds have been designed based on the enzyme–substrate specificity, and their elongated analogues are expected to demonstrate significant inhibition against the HIV-1 protease with IC_50_ values in the low nanomolar range. Moreover, a highly efficient esterification protocol with carbohydrates and flavonoids has been devised to address the inherent absorption challenges associated with phosphinic-based drugs. These esters not only exhibit low toxicity but also have the potential to generate flavonoid moieties in situ, which are associated with hepatoprotective effects, or naturally occurring carbohydrate metabolites. The methodology utilizes effective peptide coupling reagents, such as aminium-based TBTU and carbodiimide-based DIC, and affords the target products in excellent to quantitative yields. This research represents a promising avenue for the development of novel HIV-1 protease inhibitors with significant therapeutic benefits.

## Introduction

**Figure 1:**
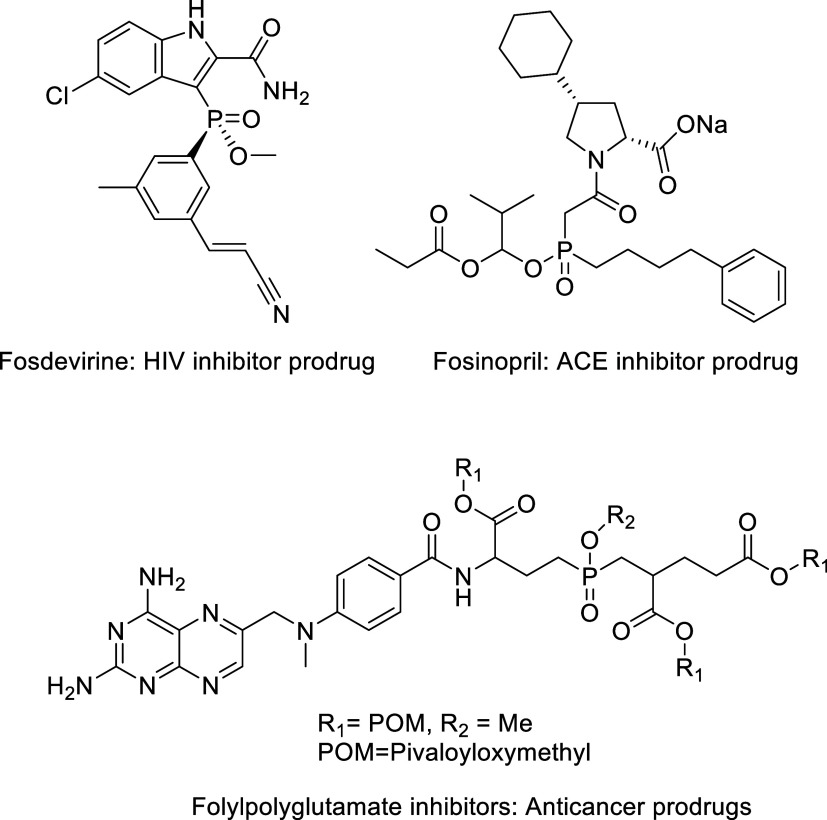
Structures of phosphinic acid prodrugs and drugs with clinical applicability.

Phosphate-phosphonate or phosphinate-containing compounds emerge as an important class of bioactive compounds exhibiting significant potential for treating various diseases.^1^ Specifically, the phosphinic moiety [−P(O)(OH)–CH_2_−], being an isostere of the amide bond upon hydrolysis, is a nonhydrolyzable mimic of the tetrahedral transition state. Thus, phosphinate pseudopeptides have attracted considerable attention, providing a wide range of potent inhibitors of proteolytic enzymes, particularly, metalloproteases and aspartic acid proteinases. However, they exhibit intrinsic absorption issues due to their limited membrane permeability and solubility. More specifically, the in vivo administration of phosphinic acids as drugs encounters constraints compared to natural peptides because of the highly polar −P(O)(OH)– group,^2,3^ rendering them negatively charged at physiological pH values. Therefore, their efficient penetration through the cell membranes becomes challenging. In order to overcome this hurdle, phosphinic compounds are frequently administered in prodrug forms to enhance their absorption, distribution, metabolism, and excretion (ADME) effects, enhancing diffusion across cell membranes and oral absorption with increased lipophilicity. A representative example is fosinopril (Figure 1), a hypertension drug marketed for more than 30 years. Following absorption and enzymatic hydrolysis, the prodrug releases the active drug within cells.^4^ Nevertheless, some unwanted metabolic or clearance mechanisms, such as the delayed conversion of a neutral alkyl ester of phosphinate, phosphonate, or phosphate in most tissues, make the selection of a prodrug moiety crucial. Taking these parameters into account, the improved synthetic methods toward phosphinates and their potential prodrugs are still attracting considerable interest (Figure 1).^1−3,5^

**Scheme 1:**
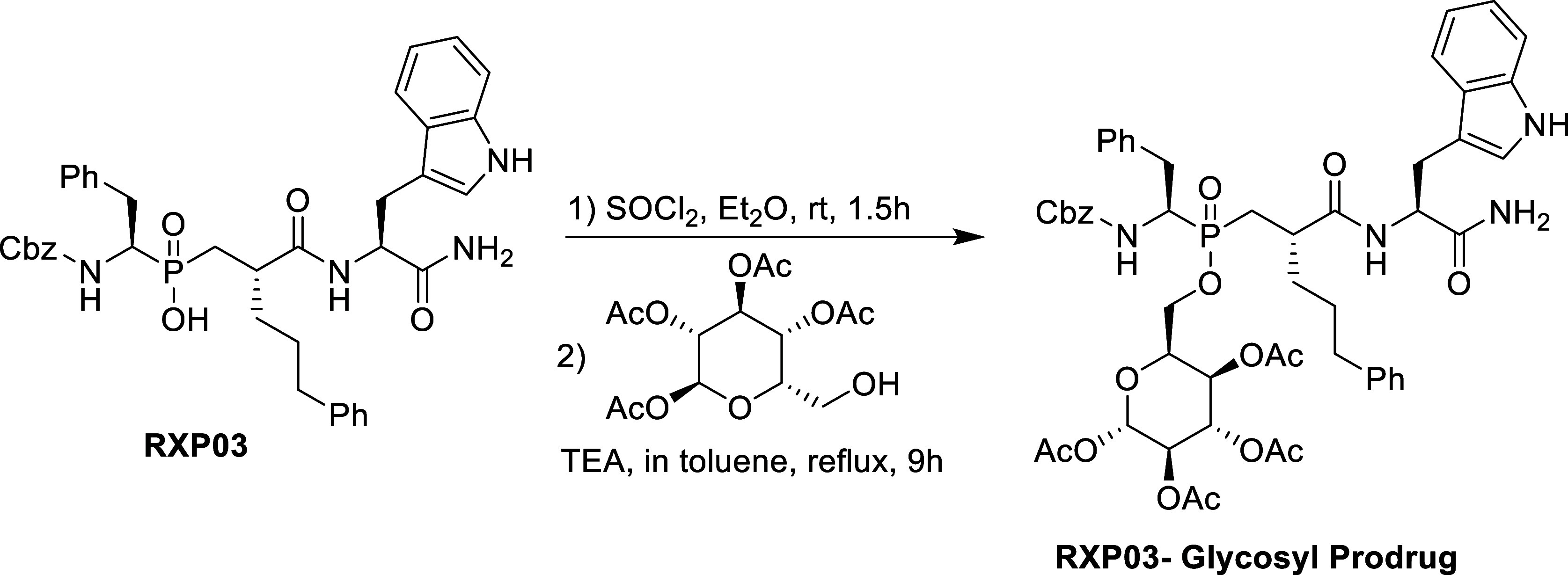
Conversion of RXP03 to Its Glycosyl Prodrug

Although numerous studies have investigated the synthesis of phosphinic and phosphonic esters, reports on the direct functionalization of [−P(O)(OH)−] compounds are limited, with most of them focusing on simple alkyl esters. Interestingly, several phosphinate esters have been developed and evaluated in clinical trials, showcasing enhanced efficacy compared to their parent drugs in drug delivery and ADME effects (Figure 1).^2,6−8^ However, no carbohydrates or flavonoid-based phosphinic prodrug has been documented; until recently, when the glycosyl prodrug of **RXP03** put an end to this gap, exploiting our previously developed esterification method (Scheme 1).^9,10^

**Figure 2:**
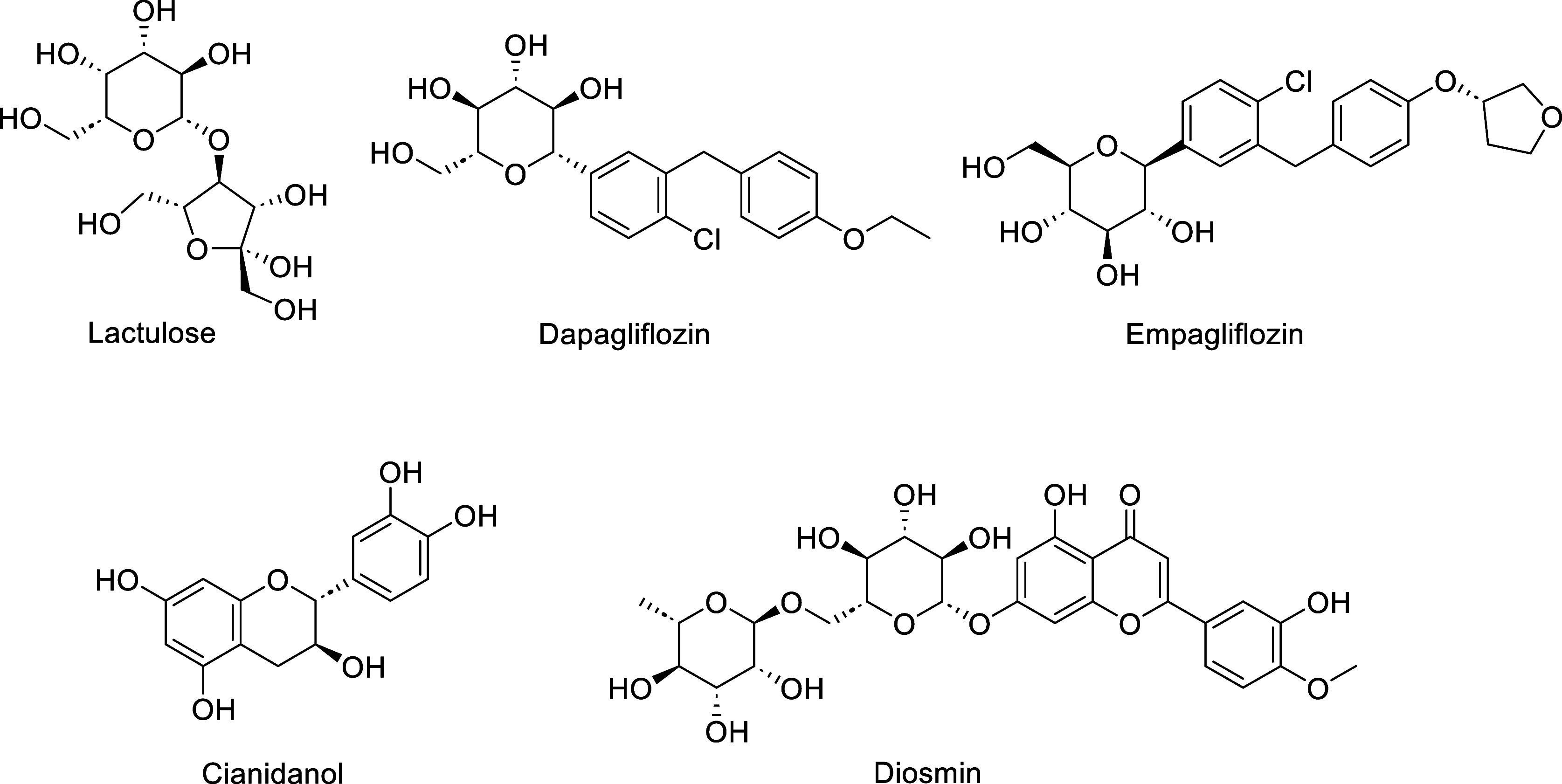
Structures of carbohydrates and flavonoid-based drugs.

Due to their therapeutic significance, carbohydrates and flavonoid-based prodrugs have been utilized in various drug conjugates, leveraging their antioxidant, anticancer, cardioprotective, neuroprotective, antidiabetic, and antiviral properties.^11−23^ For instance, lactulose is prescribed as a laxative,^24^ also used to treat or prevent certain conditions of the brain that are caused by liver failure, such as hepatic encephalopathy.^25^ Dapagliflozin and empagliflozin are used to treat type-2 diabetes.^26,27^ Cianidanol acts as a selective estrogen receptor beta agonist. Diosmin has been widely used as a phlebotonic and vascular protector for venous leg ulcers (Figure 2).

The predictive study for permeability enhancement of the glycosyl ester of **RXP03** (Scheme 1) revealed significant insights toward the improvement of drug delivery across biological barriers.^9^ Another aspect of incorporating carbohydrate moieties is that it might facilitate transport pathways across multiple biological barriers.^28−30^ Furthermore, the carbohydrate byproducts generated from ester cleavage are natural metabolites with the resulting drug lacking stereogenicity at the phosphorus atom. Therefore, employing the esterification strategy of phosphinic acids with selected carbohydrate derivatives offers the potential for producing less polar and hence more soluble variants of the phosphinate candidates. The method used for the discussed **RXP03** glycosyl prodrug employed transition-metal-free acylation of the phosphinic acid esterification reaction with carbohydrates and yielded the esters in excellent yields.^10^ Nevertheless, that method required the use of highly corrosive and moisture-sensitive SOCl_2_, also generating HCl, which is incompatible with acid-sensitive functional groups. As part of our ongoing efforts in this area, we herein report the efficient and controllable esterification of symmetrical phosphinic acids with carbohydrates and flavonoids under mild conditions using the coupling reagents TBTU and DIC. The inclusion of flavonoids in this study is motivated by their potential to generate flavonoid moieties in situ, which are renowned for their protective effect against liver injury.^31^ Furthermore, their use as fluorophores for studying cell imaging in therapeutic applications provides another compelling advantage. Notably, the protocol reported herein has enabled the synthesis of a wide range of esterification products in high to quantitative yields.

**Figure 3:**
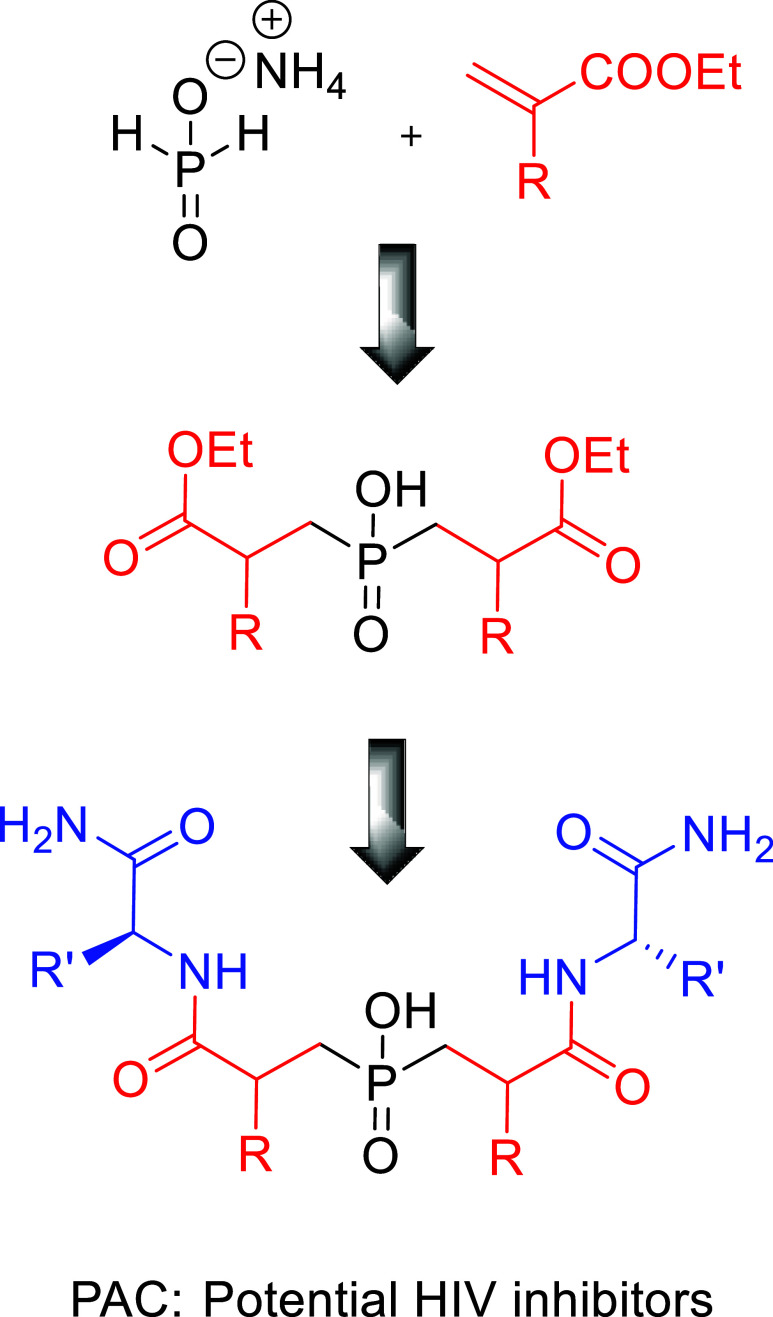
Outline of the design of potential inhibitors of HIV protease.

Another goal of this study was to synthesize the central building blocks based on symmetrical phosphinic motifs. These blocks can be further incorporated into peptide sequences for the design of HIV-1 protease inhibitors as potential HIV therapeutics. Generally, phosphinic pseudopeptides are analogues intended to mimic a high-energy intermediate involved in hydrolysis.^32−34^ Various nonhydrolyzable moieties, including the amino acid statine, secondary amines, hydroxyethylenes, hydroxyethylamines, and ketones, have been incorporated in these peptides to replace the scissile peptide bond.^35^ Given that HIV-1 protease is a *C*_2_-symmetric, homodimeric enzyme, several researchers have attempted to develop inhibitors with symmetrical features in the late 20th century.^36−39^ One of the pioneering work of Abdel-Meguid et al.^33^ laid a solid foundation to investigate the development of potential inhibitors of HIV protease exploiting symmetrical phosphinic motifs. However, the lack of efficient methods for synthesizing such phosphinic-based compounds and their intrinsic absorption issues have limited the exploration of this research area to date, resulting in relatively few studies. In the light of the discussed findings and continuation of our prior work on the synthesis of the P-analogue of lopinavir^40^ and the encouraging results from carbohydrate prodrugs,^9^ we herein report the synthesis of symmetrical phosphinic acids, using simple and efficient methods, yielding excellent results. These phosphinates incorporate alkyl chains at P_1_ and P_1_′ positions akin to glycine, alanine, leucine, phenylalanine, and the non-natural phenylpropyl chain, employing either commercially available or easily accessible acrylates.^41^ These compounds have been synthesized with the aim of developing the potential inhibitors of HIV protease designated as “PAC”, the generic structure is illustrated in Figure 3.

## Results and Discussion

### Synthesis of Phosphinic Pseudopeptides **3a** and **3b**

**Scheme 2:**
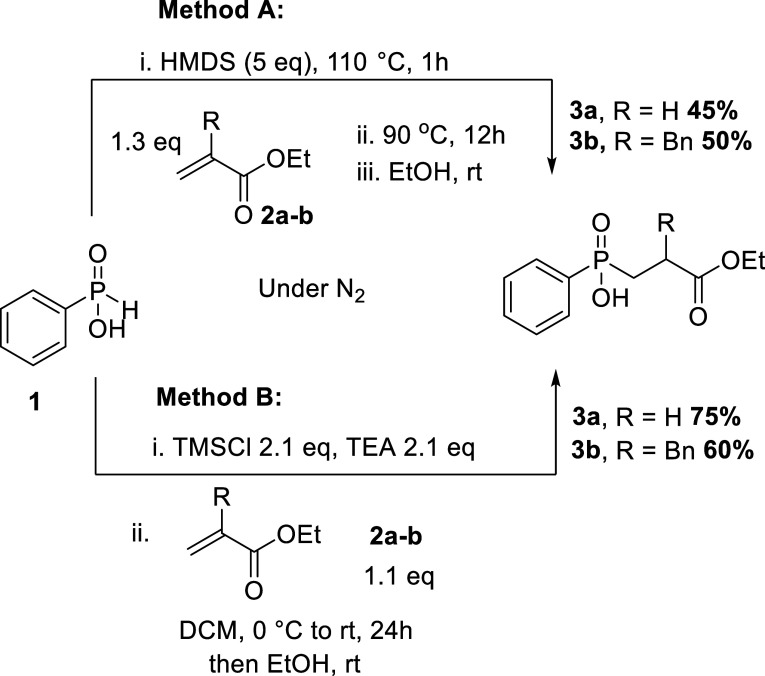
Synthesis of Phosphinic Pseudopeptides **3a** and **3b** by HMDS and TMSCl/TEA Activation

To assess the feasibility and efficiency of the proposed method and to evaluate the impact of steric hindrance in the esterification reactions, we initially examined compounds **3a** and **3b**. These compounds can be easily synthesized by using the commercially available phenyl phosphinic acid PhPO_2_H_2_**1** and acrylates with side chains akin to glycine and phenylalanine. The target compounds were obtained by the Michael addition of **1** to the respective acrylates **2a** and **2b**.^41^ Two activation/silylation agents, hexamethyldisilazane (Scheme 2, method A) and trimethylsilyl chloride (TMSCl) (Scheme 2, method B), have been applied to afford the target compounds in moderate yields. However, with hexamethyldisilazane (HMDS) activation, the products were formed with some inseparable polymerized byproducts as impurities. TMSCl provided higher yields without byproduct formation, owing to the milder conditions involved.^42^

### Attempts for One-Pot Synthesis of Symmetrical Pseudopeptides

For the development of bis-alkylated symmetrical phosphinates, we commenced our study via a one-pot strategy starting from hypophosphorous ammonium salt.^43^ We performed a few attempts, summarized in Table 1, using HMDS and TMSCl as activating agents for the Michael addition to acrylate **2b**. Nevertheless, the reaction mixture mainly consisted of the monoalkylated phosphinic acid as the major product rather than the desired bis-alkylated one; the conversion rates and yields were determined by ^31^P NMR (entries 1 and 4). Besides, even at an elevated temperature (110 °C), the desired product was not formed; instead, the mixture contained proportionally more byproducts, attributed to possible acrylate’s polymerization (entries 2–3).

**Table 1:** Attempts to Synthesize Symmetrical Phosphinates via a One-Pot Method

entry	silylating agent	acrylate	time (h)	temperature (°C)	outcome
1	HMDS^a^: 3 equiv	**2b** 3 equiv	24	rt	RP(O)(OH)H
2	HMDS^a^: 3 equiv	**2b** 3 equiv	24	100 °C	RP(O)(OH)H > RP(O)(OH)R
3	HMDS^a^: 5 equiv	**2b** 5 equiv	12	100 °C	byproducts ≥ RP(O)(OH)R > RP(O)(OH)H
4	TMSCl/TEA 5equiv in DCM	**2b** 5 equiv	24	rt	mixture (SM + RP(O)(OH)H)

a Activated hypophosphorous ammonium salt with HMDS at 110 °C for 1 h.

**Scheme 3:**
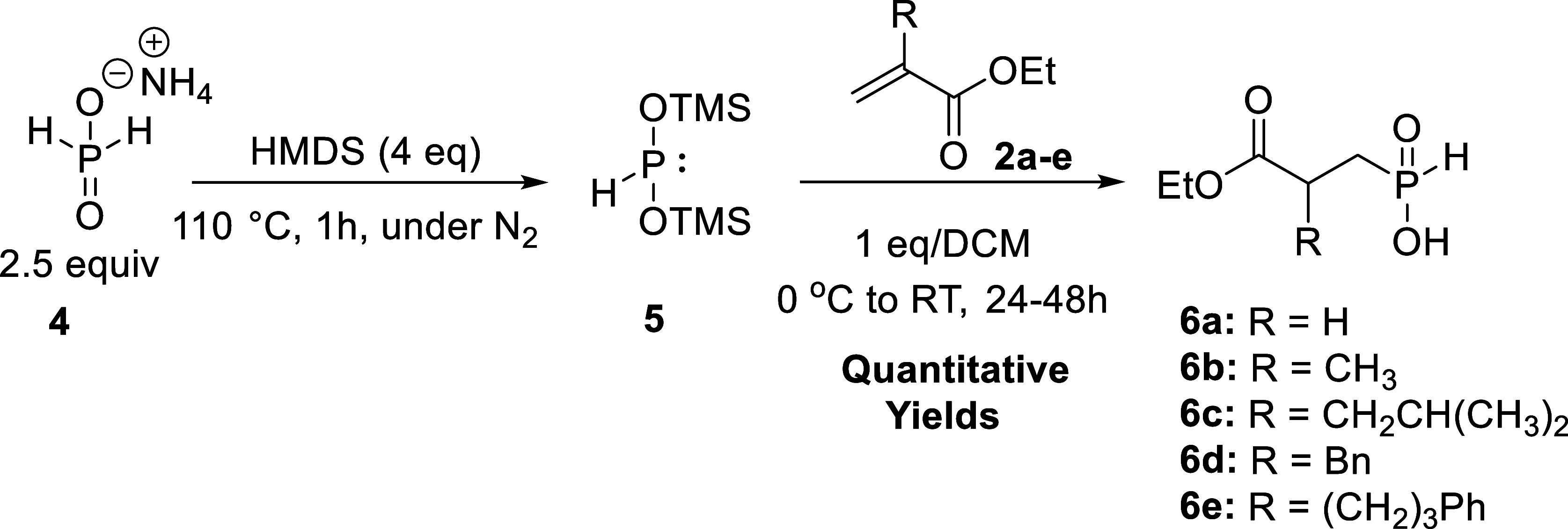
Synthesis of Symmetrical Phosphinic Acids: Step 1

Since the attempts to synthesize the target bis-alkylated products in one pot were not successful, we further explored the study with a two-step procedure. The activation of the hypophosphorus ammonium salt **4** was achieved by heating in HMDS at 110 °C to provide the bis(trimethylsilyl) hypophosphite intermediate **5** (Scheme 3). The activation was followed by a phospha-Michael reaction with differently substituted acrylates **2a–e** (1 equiv) serving as Michael acceptors to achieve the formation of the corresponding phosphinic acids **6a–e** (Scheme 3). Following several rounds of optimization involving adjustments of the molar ratio, temperature, and reaction times, the products were obtained in quantitative yields. The purification process involved simple workup procedures and no need for column chromatography purification.

**Scheme 4:**
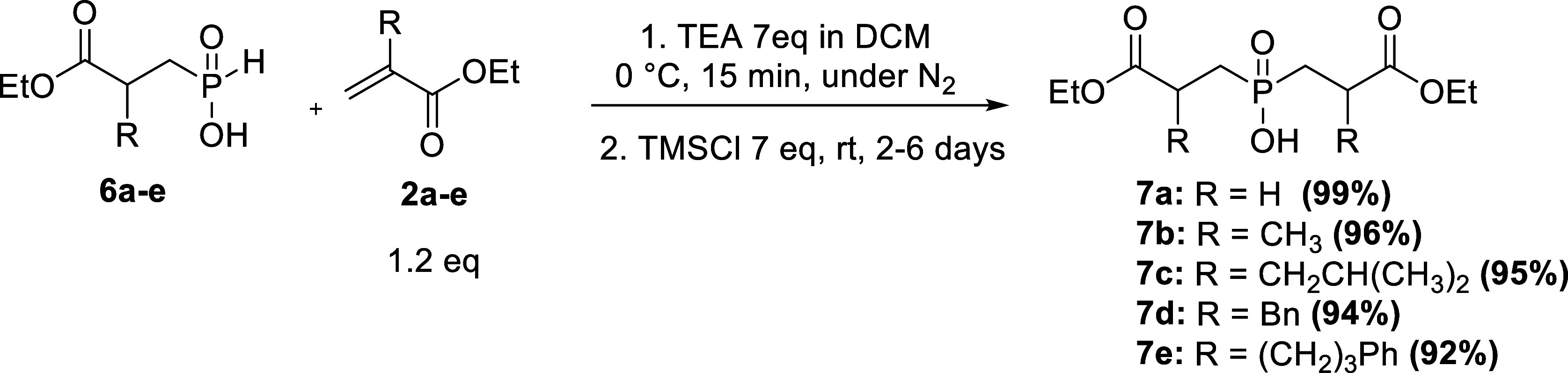
Synthesis of Symmetrical Phosphinic Acids: Step 2

The second P–C bond formation was achieved via activation of phosphinic acids **6a–e** using TMSCl in the presence of TEA. Although the reaction required a longer time for complete conversion, it proceeded smoothly, and the yields were high. The target compounds were purified chromatographically^44^ (Scheme 4).

In conclusion, after a thorough investigation of different methods for synthesizing the desired phosphinates, the sequential two-step methodology was found to be optimal for generating a diverse library of symmetrical phosphinic acid in excellent yields of up to 99%.

### Esterification Optimization of Phosphinic Acid **3a** with Galactose Acetonide

The investigation started using phosphinic acid **3a** and diacetone-d-galactose **8** as coupling partners, employing TEA as a base. A variety of coupling reagents has been assessed, such as DIC (diisopropylcarbodiimide), EDC (1-ethyl-3-(3-(dimethylamino)propyl)carbodiimide), HATU (1-[bis(dimethylamino)methylene]-1*H*-1,2,3-triazolo[4,5-*b*]pyridinium 3-oxid hexafluorophosphate), HBTU (2-(1*H*-benzotriazole-1-yl)-1,1,3,3-tetramethyluronium hexafluorophosphate), BOP (benzotriazole-1-yl-oxy-tris(dimethylamino)-phosphonium hexafluorophosphate), and PyBOP (benzotriazol-1-yloxytripyrrolidinophosphonium hexafluorophosphate) in DMF with diverse molar ratios. None of them afforded the product in considerable yield. Even at elevated temperatures, product **9a** was formed in low to moderate yields (Table 2, entries 1–6). However, employing TBTU ((*N*,*N*,*N*′,*N*′-tetramethyl-*O*-(benzotriazol-1-yl) uronium tetrafluoro borate)) as the coupling reagent in DMF afforded the ester product **9a** in 62% yield in 3 h which increased to 85% within 8 h (Table 2, entries 7–8). Inspired by this encouraging result, the optimization of the reaction was performed with a variety of solvents, and selected data are summarized in Table 2 (entries 9–13). The yield of the reaction was further increased by increasing the temperature, affording the product in a shorter reaction time both in DCE and in DMF. Overall, the highest conversion to the target product was obtained in DCE with 93% yield at rt and 96% at 75 °C (Table 2, entries 13 and 15).

**Table 2:**
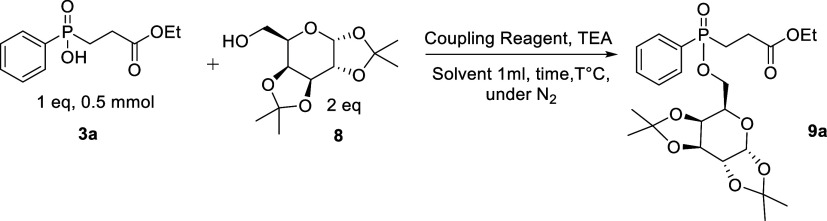
Optimization of Reaction Conditions for Esterification with Carbohydrates

entry	coupling reagent	temperature (°C)	*T* (h)	solvent	yield (%)
1	DIC	80	3	DMF	21^a^
2	EDC	80	3	DMF	36^a^
3	HATU	80	3	DMF	46^a^
4	HBTU	80	3	DMF	37^a^
5	BOP	80	3	DMF	40^a^
6	PyBOP	80	3	DMF	38^a^
7	TBTU	rt	3	DMF	62^a^
8	TBTU	rt	8	DMF	85^b^
9	TBTU	rt	8	CHCl_3_	45^b^
10	TBTU	rt	8	CH_3_CN	58^b^
11	TBTU	rt	8	dioxane	62^b^
12	TBTU	rt	8	DCM	83^b^
13	TBTU	rt	8	DCE	93^b^
14	TBTU	80	3	DMF	90^b^
15	TBTU	75	3	DCE	96^b^

a Reactions were carried out using **3a** (0.6 mmol), coupling reagent (0.6–1 mmol), TEA (1 mmol), and **8** (1 mmol) in DMF (1 mL) for 3 h.

b **3a** (0.5 mmol), TBTU (0.5 mmol), TEA (1 mmol), and **8** (1 mmol) in dry solvents (1 mL).

**Scheme 5:**
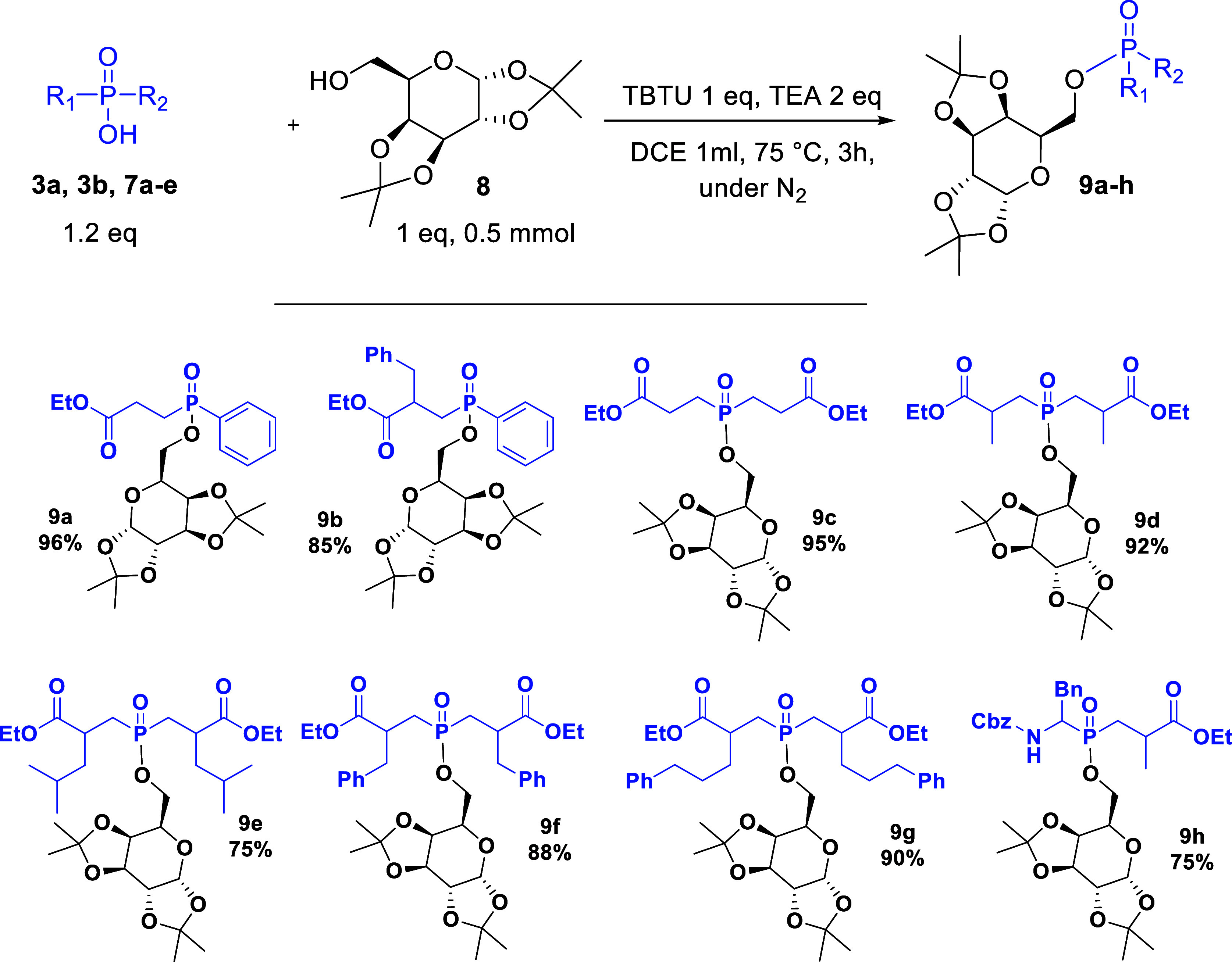
Substrates’ Scope with Variously Substituted Phosphinic Acids’ Esterification Phosphinic acid (0.6 mmol), diacetone-d-galactose **8** (0.5 mmol), TBTU (0.6 mmol), and TEA (1 mmol) in DCE (1 mL) under N_2_ for 3 h at 80 °C.

With the optimal conditions in hand (Table 2, entry 15), the method’s applicability was subsequently assessed for a variety of substituted phosphinates, focusing on symmetrical units, which afforded the desired products in excellent yields (Scheme 5). At the outset, we evaluated the impact of substituents attached to the α-position of phosphinic acids. As expected, the phosphinic acid **3b** bearing the benzyl group side chain furnished the product **9b** in a lower yield than the unsubstituted phosphinic acid **3a**, which can be attributed to steric hindrance. Similarly, the yields for the esterification of the symmetrical acids followed the same pattern, with the lowest recorded (75%) being for **9e**, that bears the bulkiest *sec*-butyl group. With the polarity of compounds **9c–g** being similar to that of the galactose acetonide **8**, we modified the molar ratios of the coupling partners and employed the carbohydrate component as the limiting reagent along with adjustments of other reagents accordingly, so as to facilitate chromatographic purification (Scheme 5). Therefore, compounds **9c–9h** were synthesized with these optimized conditions: phosphinic acid (0.6 mmol), diacetone-d-galactose **8** (0.5 mmol), TBTU (0.6 mmol), and TEA (1 mmol) in DCE (1 mL) under N_2_ for 3 h at 80 °C, affording the products in up to 96% yield. Besides, in order to compare the efficiency of this protocol with our previous method based on SOCl_2_,^10^ product **9h** was synthesized, the outcome of the current method provided the product in increased yield, 75%. Hence, our study revealed that the developed protocol not only tolerated the steric hindrance of isopropylidene ketal carbohydrate-protecting groups and the phosphinic side chains but also provided a milder and more convenient method for generating carbohydrate-based phosphinic esters even on a large scale, yielding excellent results.

**Scheme 6:**
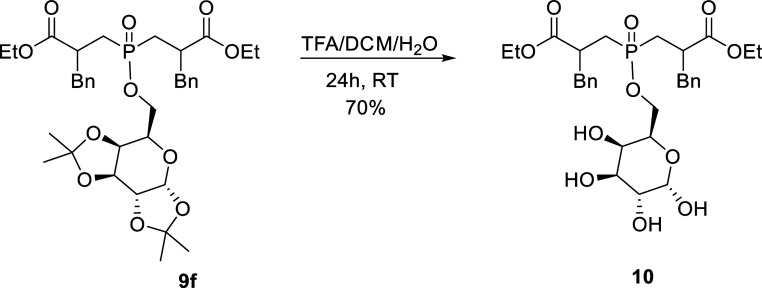
Removal of the Isopropylidene Group

Keeping in mind that the target carbohydrate prodrugs need to be unmasked to optimally penetrate the cell membranes by increasing the hydrophilicity, the acetonide-protecting groups need to be removed. As a representative case, the removal of the isopropylidene groups of compound **9f** was performed by using TFA/DCM/H_2_O^45^ and resulted in compound **10** with a satisfactory yield (Scheme 6).

### Esterification Optimization of Phosphinic Acid **3a** with Flavonoids

The study of esterification was further extended to flavones as the ester coupling partner. For this study, we chose the phosphinic acid **3a** and 3-hydroxyflavone **11** as coupling partners, employing TEA as a base. Our first approach was to employ the optimal coupling reagent identified in carbohydrate ester formation studies. Interestingly, the efficiency of TBTU in this case was remarkably low, as confirmed by ^31^P NMR analysis following isolation (Table 3, entry 1). This might be attributed to the entirely different nature of the hydroxyl group in flavone compared to the corresponding hydroxyl group of carbohydrates. This prompted us to evaluate numerous other coupling reagents including CDI (*N*,*N*-carbonyldiimidazole), DCC (dicyclohexylcarbodiimide), EDC (1-ethyl-3-(3-dimethylaminopropyl)carbodiimide hydrochloride), HATU (1-[bis(dimethylamino)methylene]-1*H*-1,2,3-triazolo[4,5-*b*]pyridinium-3-oxid hexafluorophosphate), and BOP (benzotriazole-1-yl-oxy-tris(dimethylamino)-phosphoniumhexafluorophosphate), entries 2–8. Their efficiency was found to follow the order: BOP > DCC > HATU > EDC > TBTU > CDI. Therefore, employing BOP, different solvents (DCE, DMF, DCM, CH_3_CN, THF, DMF, and 1,4-dioxane) were screened, and optimum results were obtained with DMF, affording **12a** in 43% yield (Table 3 entries 8–13). However, in pursuit of improving the results and finding the BOP/DMF combination unsatisfactory, we encompassed another commonly used carbodiimide coupling reagent, DIC in our investigation (entries 14–18). The efficiency of DIC surpassed all other screened coupling reagents, and the yield was improved to 57%. However, changing the solvent from DMF to DCE decreased the yield to 37% (entry 15). Furthermore, by increasing the temperature to 80 °C, the yield of the product significantly increased to 99% in DMF and 92% in DCE, as determined by ^31^P NMR (entries 16, 17). Surprisingly, the isolated yield was found to be 57% (entry 17) which presented a significant conflict with spectroscopic and TLC analyses. This has been attributed to decomposition of the product during column purification. The hydrolysis of the ester **12a** was evident by the reappearance of the starting phosphinic acid, collected through the flash column and confirmed by NMR. Taking advantage of the significant polarity difference between the phosphinic acid and the ester, and with the aim to avoid a column purification, we modified the reaction conditions (entry 18), which afforded the pure products by simple workup with outstanding results. Based on the screening results, the optimal conditions for this esterification reaction were therefore established as follows: phosphinic acid (0.6 mmol), 3-hydroxyflavone **11** (0.5 mmol), DIC (0.65 mmol), and TEA (1 mmol) in DMF (1 mL) under N_2_ for 3 h at 80 °C (Table 3, entry 18).

**Table 3:**
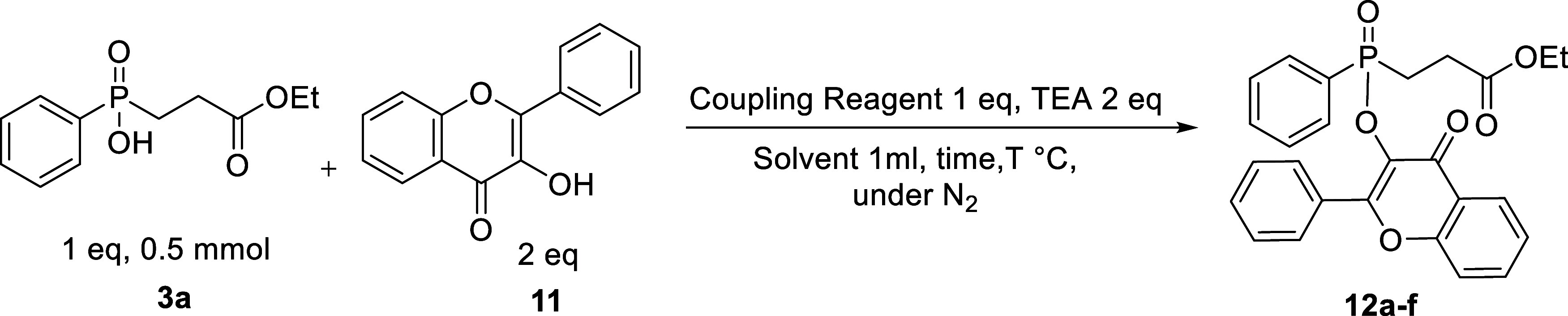
Optimization of Reaction Conditions for Flavonol Esterification

entry	coupling reagent	temperature (°C)	*T* (h)	solvent	^31^P NMR yield %
1	TBTU	rt	12	DMF	13^a^
2	CDI	rt	12	DMF	4.8^a^
3	DCC	rt	12	DMF	25.3^a^
4	EDC	rt	12	DMF	11.5^a^
5	EDC/HOBT	rt	12	DMF	3^a^
6	HATU	rt	12	DMF	10^a^
7	HATU/HOBt	rt	12	DMF	2^a^
8	BOP	rt	12	DMF	43^a^
9	BOP	rt	12	DCE	30^a^
10	BOP	rt	12	DCM	40^a^
11	BOP	rt	12	CH_3_CN	28^a^
12	BOP	rt	12	THF	38^a^
13	BOP	rt	12	dioxane	10^a^
14	DIC	rt	12	DMF	57^a^
15	DIC	rt	12	DCE	37^a^
16	DIC	80	12	DMF	99^a^(57% yield)^b^
17	DIC	80	12	DCE	92^a^
18	DIC	80	3	DMF	99^c^(95% yield)^b^

a Reactions were carried out using **3a** (0.5 mmol), coupling reagent (0.55 mmol), TEA (1 mmol), and **11** (1 mmol) in a dry solvent (1 mL).

b Yield, determined after purification.

c **3a** (0.6 mmol), DIC (0.65 mmol), TEA (1 mmol), and **11** (0.5 mmol) in a dry solvent (1 mL) for 3 h at 80 °C, simple workup afforded the pure product, and no column required.

**Scheme 7:**
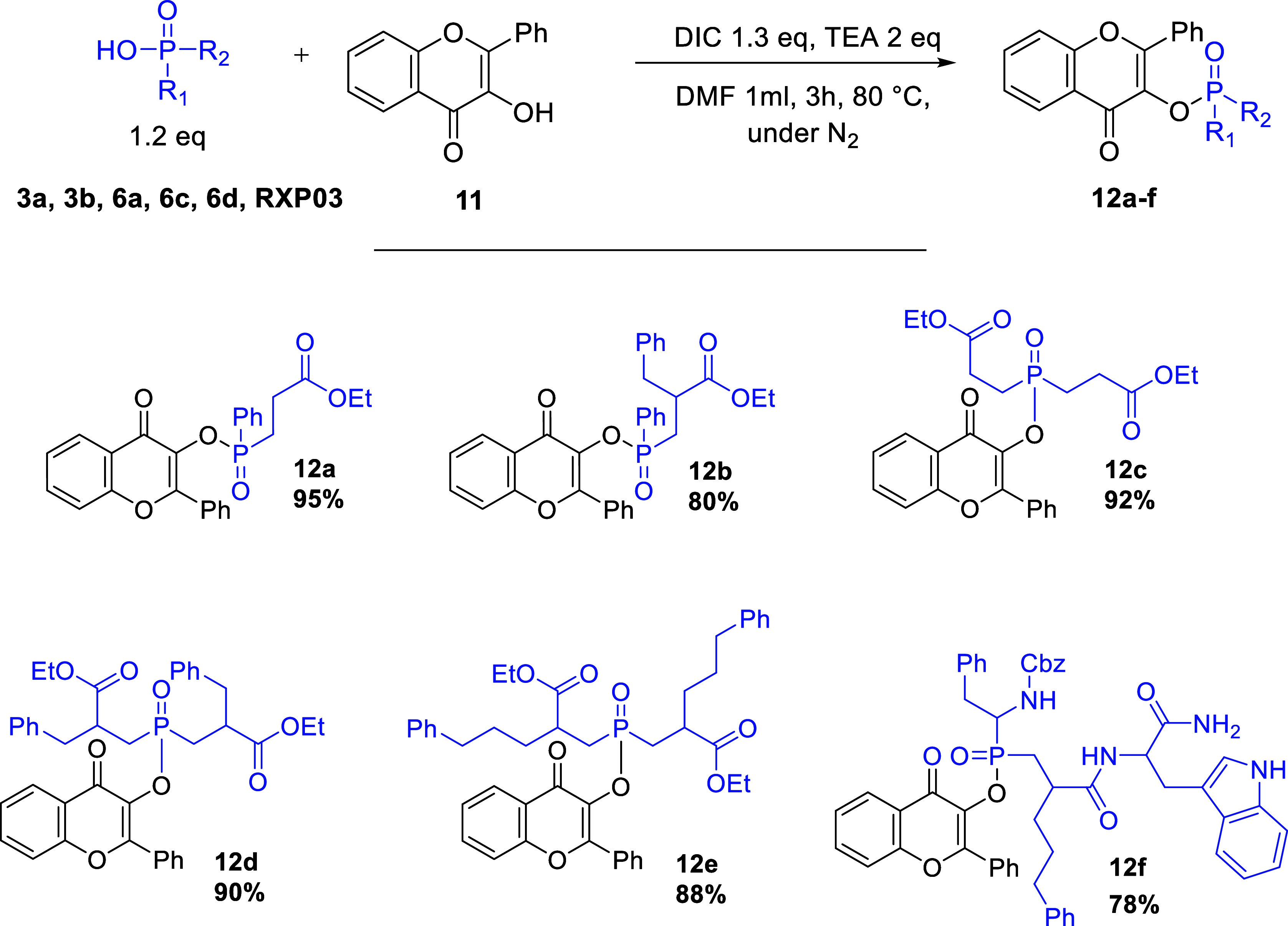
Substrates’ Scope with Variously Substituted Phosphinic Acid Esterification with Flavonol Phosphinic acid (0.6 mmol), 3-hydroxyflavone **11** (0.5 mmol), DIC (0.65 mmol), and TEA (1 mmol) in DMF (1 mL) under N_2_ for 3 h at 80 °C.

In order to widen the scope of this methodology and to compare the method’s applicability with the carbohydrate esterification approach, the study was extended to a panel of phosphinic acids bearing various substituents with different electronic and steric properties. The target products were produced in excellent yields, up to 95%, as summarized in Scheme 7, proving the method’s efficiency. Furthermore, the incorporation of various symmetrical phosphinic acids bearing different alkyl chains in the investigation also proved equally successful, with the formation of **12c–e** in yields up to 92%. In addition, we performed the reaction toward the formation of the flavonol ester of **RXP03**,^46^ the method afforded the product **12f** in high yield (78%). The developed esterification method showed wide applicability to a broad range of substrates; however, as mentioned earlier, these products are prone to hydrolysis, which may limit their applicability as prodrugs by releasing the parent phosphinic acid in an untimely fashion. Nevertheless, to the best of our knowledge, this is the first time that flavone-based prodrug moieties have been reported with phosphinic acids; aiming at a new approach for using such fluorophore-based compounds. Hence, after the successful synthesis of a library of novel phosphinic esters, our next objective is to elongate the structures presented herein, so as to develop potent HIV-1 protease inhibitors and their corresponding prodrugs.

## Conclusions

Phosphinic acids are increasingly recognized for their crucial role in understanding and influencing biological processes. In our pursuit of developing *C*_2_-symmetrical HIV protease inhibitors to complement the enzyme’s symmetry, we synthesized the basic building blocks for the phosphinic pseudopeptides, that can be further elongated. These compounds incorporate diverse alkyl groups at the P_1_ and P_1_′ positions via easily accessible acrylates, in excellent to quantitative yields and also manifest comparable efficiency at 20 g-scale synthesis. Additionally, we devised an improved, efficient, and controllable method for generating phosphinic esters by directly esterifying symmetrical [−P(O)(OH)−] acids with carbohydrates and flavonoids. To our knowledge, this approach marks the first instance of establishing esters with such nontoxic and advantageous properties through operationally simple means, employing effective coupling agents under mild conditions. Notably, our protocol demonstrates robust tolerance toward sterically hindered substrates while circumventing the use of metals and harsh conditions. All reactions proceed with high efficiency, granting fast access to various novel organophosphorus compounds. The straightforward workup, simple purification process, mild reactions’ conditions, high yields, and clean reaction profiles render this method a compelling and valuable addition to current methodologies. We anticipate that this esterification strategy will facilitate the construction of pseudopeptide libraries without constraints on the peptide length or bulk of the functional group present in side chains and also will serve in the synthesis of other biologically active molecules and catalytic ligands. Implementation of this attractive methodology to prepare biologically relevant phosphinate analogues is underway and will be reported in due course.

## Experimental Section

### General Experimental Information

All glassware was oven-dried, and all reactions using air-sensitive materials were carried out under a N_2_ atmosphere. Dry solvents, where needed, were procured by an Inert PureSolv^TM^ Solvent Purification System (Et_2_O, THF, and DCM). All other solvents were of AR grade and used without further purification unless stated otherwise. Commercially available reagents were purchased from Aladdin, TCI, and Adamas and were used without further purification. All column chromatography purifications were conducted using silica gel (230–400 mesh, Silicycle) hand-packed with varying ratios of hexanes and ethyl acetate (hexanes/EtOAc) or dichloromethane and methanol (DCM/MeOH) unless otherwise indicated. Silica G TLC plates (Sorbtech, polyester backed, thickness 200 μM, and fluorescence UV254) were used for monitoring the reaction progress. For NMR analysis, all samples were dissolved in CDCl_3_ (D-99.8%, +0.05% v/v tetramethylsilane (TMS)) unless stated otherwise. ^1^H, ^31^P, and ^13^C NMR spectra were recorded on Bruker Avance III 400 and 600 spectrometers and processed with MestreNova software. Chemical shifts are reported in ppm and coupling constants are reported in hertz (Hz). ^1^H NMR spectra in CDCl_3_ are referenced to TMS at 0.00 ppm and reported using the format: chemical shift (ppm) [multiplicity (s = singlet, d = doublet, t = triplet, q = quartet, m = multiplet, and app = apparent), coupling constant(s) (*J* in Hz), and integral]. ^13^C NMR spectra are referenced to CDCl_3_ at 77.0 ppm. ^31^P NMR spectra are referenced to H_3_PO_4_.

#### General Procedure for the Preparation of Synthons **3a–b**

##### Method A: HMDS Activation

To an oven-dried flask equipped with a stirring bar, phenylphosphinic acid **1** (12.0 mmol) was placed, followed by HMDS (5 equiv, 60.0 mmol) and stirred at 110 °C for 1 h under N_2_ until the reaction mixture became a clear solution. Then, the reaction mixture was cooled to 90 °C, and the acrylate was added dropwise (1.3 equiv, 15.6 mmol) over 20 min. The reaction mixture was allowed to stir at rt overnight. The reaction was quenched upon completion with a dropwise addition of absolute EtOH (50 mL). The volatiles were removed under vacuum, and the residue was dissolved in 5% NaHCO_3_ and extracted with Et_2_O (2 × 30 mL). The aqueous layer was acidified using HCl 1 M to pH = 1, extracted with EtOAc (2 × 30 mL), dried over Na_2_SO_4_, and concentrated. The residue was purified by column chromatography using DCM/MeOH/AcOH (7/0.2/0.2) as eluent and products were obtained as white solids.

##### Method B: TMS Activation

In an ice-cold suspension of phenylphosphinic acid **1** (14 mmol) in dry DCM (28 mL), Et_3_N (2.1 equiv) was added followed by chlorotrimethylsilane (2.1 equiv) at 0 °C, under an N_2_ atmosphere. The resulting solution was stirred for 3 h at rt, then cooled to 0 °C, and the appropriate acrylate was added (1.1 equiv) dropwise dissolved in DCM (7 mL). The solution was stirred at rt for 24 h or until completion. Absolute EtOH (30 mL) was added slowly at 0 °C and stirring was continued for 30 min at rt. The solvents were evaporated and the residue was dissolved in 5% NaHCO_3_ and extracted with Et_2_O (2 × 30 mL). The aqueous layer was acidified with HCl 1 M to pH = 1, extracted with EtOAc (2 × 30 mL), dried over Na_2_SO_4_, and concentrated. Purification by column chromatography using DCM/MeOH/AcOH (7/0.2/0.2) as eluent afforded the products as white solids.

##### (3-Ethoxy-3-oxopropyl)(phenyl)phosphinic Acid (**3a**)

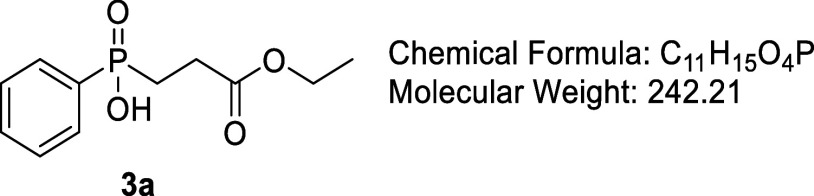



White solid (75%), mp: 41 °C. *R*_*f*_ (DCM/MeOH/AcOH = 8/0.8/0.2) 0.36. ^1^H NMR (400 MHz, CDCl_3_, δ): 7.70 (td, *J* = 8.3, 3.9 Hz, 2H), 7.52–7.45 (m, 1H), 7.44–7.34 (m, 2H), 4.04 (qd, *J* = 7.2, 1.7 Hz, 2H), 2.45 (q, *J* = 8.9 Hz, 2H), 2.13 (dt, *J* = 15.2, 8.5 Hz, 2H), 1.19 (dt, *J* = 7.1, 3.5 Hz, 3H). ^13^C NMR (101 MHz, CDCl_3_): δ 172.1 (d, *J* = 19.1 Hz), 132.2, 131.2, 131.1, 128.5, 128.4, 60.8, 26.91, 25.8, 14.1 (d, *J* = 102.0 Hz). ^31^P NMR (162 MHz, CDCl_3_): δ 42.72. HRMS (ESI) *m*/*z*: calcd for C_11_H_15_O_4_PNa [M + Na]^+^ 265.0600; found, 265.0594.

##### (2-Benzyl-3-ethoxy-3-oxopropyl)(phenyl)phosphinic Acid (**3b**)

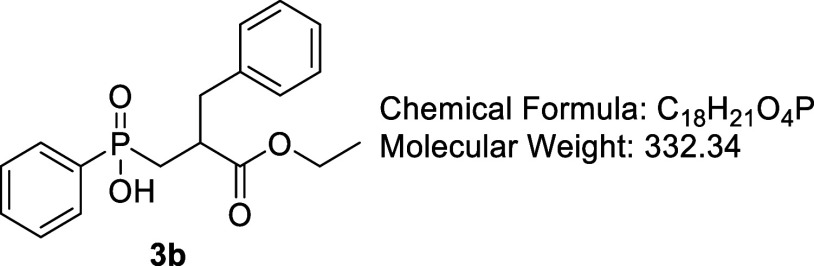



White solid (60%), mp: 72 °C. *R*_*f*_ (DCM/MeOH/AcOH = 8/0.8/0.2) 0.38. ^1^H NMR (600 MHz, CDCl_3_, δ): 7.69–7.66 (m, 2H), 7.49–7.46 (m, 1H), 7.36 (td, *J* = 7.6, 3.1 Hz, 2H), 7.22–7.16 (m, 3H), 7.06–7.04 (m, 2H), 3.88–3.78 (m, 2H), 2.95 (dtd, *J* = 12.1, 8.0, 5.2 Hz, 1H), 2.87 (app d, *J* = 7.4 Hz, 2H), 2.31 (ddd, *J* = 15.4, 13.6, 8.4 Hz, 1H), 1.98 (td, *J* = 15.5, 5.1 Hz, 1H), 1.00 (t, *J* = 7.1 Hz, 3H). ^13^C NMR (151 MHz, CDCl_3_): δ 173.90 (d, *J* = 7.5 Hz), 138.01, 132.1, 132.1, 131.3, 131.2, 129.1, 128.4, 128.3, 128.3, 126.6, 60.5, 41.2 (d, *J* = 3.0 Hz), 39.55(d, *J* = 10.5 Hz), 31.9 (d, *J* = 101 Hz), 13.9. ^31^P NMR (162 MHz, CDCl_3_): δ 43.24. MS (ESI) *m*/*z* calcd for HRMS (ESI) *m*/*z*: calcd for C_18_H_21_O_4_PNa [M + Na]^+^ 355.1067; found, 355.1069.

#### General Procedure for Phosphinic Acids **6a–e**

In an oven-dried flask, NH_4_H_2_PO_2_ (4.52 g, 39.25 mmol, 2.5 equiv) and HMDS (13.1 mL, 62.8 mmol, 4 equiv) were heated at 110 °C for 1 h under a continuous flow of N_2_ until a transparent solution was formed. Subsequently, the reaction mixture was cooled to 0 °C and dry DCM (40 mL) was added. To this cold stirring mixture, a solution of the acrylates **2a–e**, (15.7 mmol, 1 equiv) in dry DCM (20 mL), was added dropwise over 20 min. The mixture was stirred at rt for 24–48 h. The progress of the reaction was monitored by TLC. Upon completion, MeOH (40 mL) was added slowly at 0 °C and stirring was continued for 30 min at rt. The volatiles were evaporated under vacuum, and the residue was acidified to pH = 1 with 3 M HCl, taken up with DCM, and washed with 1 M HCl. The organic phase was dried over Na_2_SO_4_ and evaporated in vacuo to yield the products as pale-yellow oils.

##### (3-Ethoxy-3-oxopropyl)phosphinic Acid (**6a**)

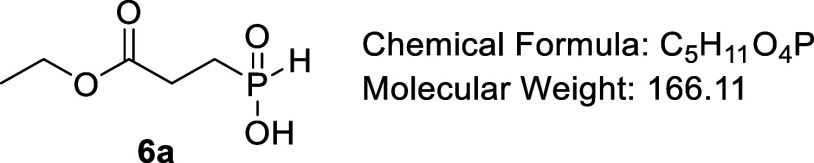



Pale-yellow viscous oil. Quantitative yield. *R*_*f*_ (DCM/MeOH/AcOH = 7/0.7/0.15) 0.29. ^1^H NMR (400 MHz, CDCl_3_, δ): 7.18 (d, *J* = 564 Hz, 1H), 4.14 (q, *J* = 7.2 Hz, 2H), 2.61 (dt, *J* = 15.5, 7.5 Hz, 2H), 2.05 (dtd, *J* = 14.9, 7.7, 2.1 Hz, 2H), 1.24 (t, *J* = 7.2 Hz, 3H). ^13^C NMR (101 MHz, CDCl_3_): δ 171.9 (d, *J* = 13.1 Hz), 61.2, 26.1 (d, *J* = 3.0 Hz), 24.4 (d, *J* = 95.9 Hz), 14.1. ^31^P NMR (162 MHz, CDCl_3_): δ 35.5. MS (ESI) *m*/*z*: calcd for C_5_H_10_O_4_P^–^ [M – H]^−^ 165.0; found, 165.0.

##### (3-Ethoxy-2-methyl-3-oxopropyl)phosphinic Acid (**6b**)

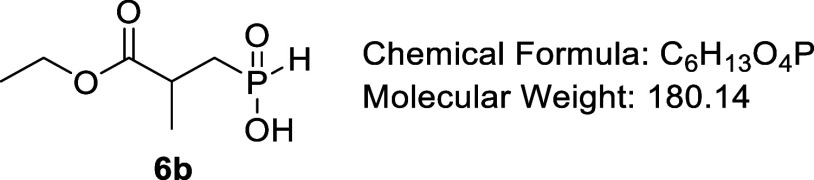



Pale-yellow viscous oil. Quantitative yield. *R*_*f*_ (DCM/MeOH/AcOH = 7/0.7/0.15) 0.32. ^1^H NMR (400 MHz, CDCl_3_, δ): 7.17 (d, ^1^*J*_P–H_ = 564 Hz, 1H), 4.13 (q, *J* = 7.2 Hz, 2H), 2.87 (dt, *J* = 13.3, 6.9 Hz, 1H), 2.24–2.14 (m, 1H), 1.88–1.77 (m, 1H), 1.29 (d, *J* = 7.2 Hz, 3H), 1.23 (t, *J* = 7.3 Hz, 3H). ^13^C NMR (101 MHz, CDCl_3_): δ 174.9, 174.8, 61.1, 33.6, 33.6, 33.3, 32.4, 18.7, 18.5, 14.1. ^31^P NMR (162 MHz, CDCl_3_): δ 34.19. MS (ESI) *m*/*z*: calcd for C_6_H_12_O_4_P^–^ [M – H]^−^ 179.1; found, 179.1.

##### (2-(Ethoxycarbonyl)-4-methylpentyl)phosphinic Acid (**6c**)

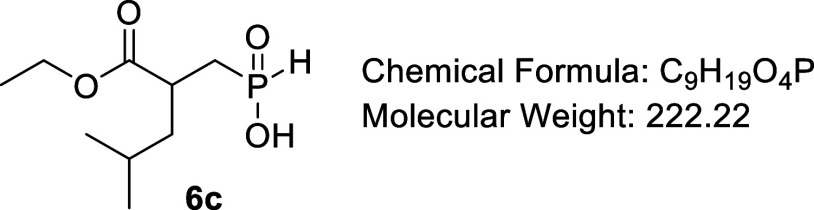



Pale-yellow viscous oil; quantitative yield. *R*_*f*_ (DCM/MeOH/AcOH = 7/0.8/0.4) = 0.42. ^1^H NMR (400 MHz, CDCl_3_, δ): 7.16 (d, ^1^*J*_P–H_ = 564 Hz, 1H), 4.15 (q, *J* = 7.1 Hz, 2H), 2.82 (ddt, *J* = 18.1, 12.5, 6.4 Hz, 1H), 2.18–2.04 (m, 1H), 1.90–1.81 (m, 1H), 1.62 (ddp, *J* = 26.1, 13.0, 6.9 Hz, 2H), 1.38 (dd, *J* = 13.0, 6.7 Hz, 1H), 1.26 (t, *J* = 7.1 Hz, 3H), 0.90 (dd, *J* = 12.7, 6.3 Hz, 6H). ^13^C NMR (101 MHz, CDCl_3_): δ 174.8 (d, *J* = 6.0 Hz), 61.0, 42.6 (d, *J* = 12.1 Hz), 37.0, 31.6, (d, *J* = 94.9 Hz), 25.7, 22.5, 22.1, 14.1. ^31^P NMR (162 MHz, CDCl_3_): δ 35.38. MS (ESI) *m*/*z*: calcd for C_9_H_18_O_4_P^–^ [M – H]^−^ 221.1; found, 221.1.

##### (2-Benzyl-3-ethoxy-3-oxopropyl)phosphinic Acid (**6d**)

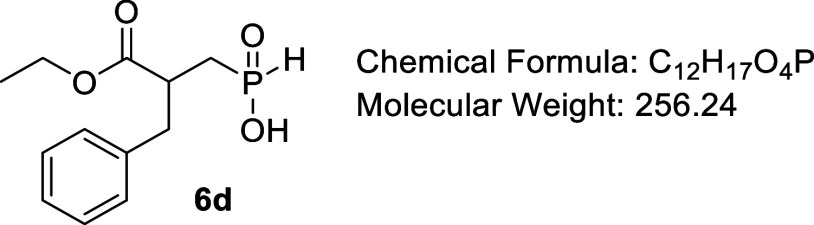



Pale-yellow viscous oil; quantitative yield. *R*_*f*_ (DCM/MeOH/AcOH = 7/0.8/0.4) = 0.53. ^1^H NMR (400 MHz, CDCl_3_, δ): 7.29 (t, *J* = 7.2 Hz, 2H), 7.24 (d, *J* = 7.1 Hz, 1H), 7.18–7.15 (m, 2H), 7.08 (d, ^1^*J*_P–H_ = 564 Hz, 1H), 4.13 (q, *J* = 7.2 Hz, 2H), 3.10–3.04 (m, 2H), 2.91 (dt, *J* = 12.8, 5.2 Hz, 1H), 2.12 (td, *J* = 14.6, 8.8 Hz, 1H), 1.90–1.80 (m, 1H), 1.20 (t, *J* = 7.1 Hz, 3H). ^13^C NMR (151 MHz, CDCl_3_): δ 172.9, 136.8, 128.4, 127.8, 126.1, 60.3, 39.9, 38.4, 29.7 (d, *J* = 95.1 Hz), 13.3. ^31^P NMR (243 MHz, CDCl_3_): δ 34.58. MS (ESI) *m*/*z*: calcd for C_12_H_16_O_4_P^–^ [M – H]^−^ 255.0; found, 255.0.

##### (2-(Ethoxycarbonyl)-5-phenylpentyl)phosphinic Acid (**6e**)

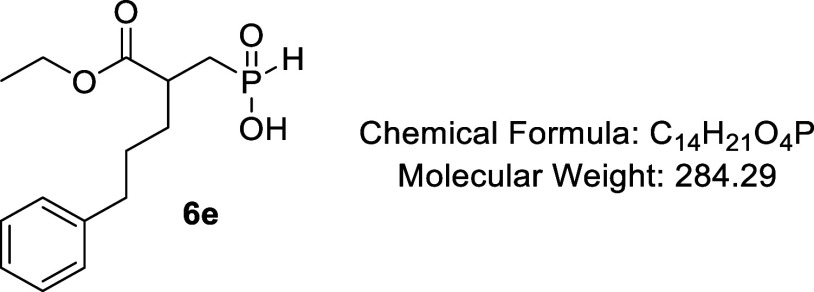



Pale-yellow viscous oil; quantitative yield. *R*_*f*_ (DCM/MeOH/AcOH = 7/0.8/0.4) = 0.58. ^1^H NMR (400 MHz, CDCl_3_, δ): 7.26–7.22 (m, 2H), 7.17–7.11 (m, 3H), 6.96 (d, ^1^*J*_P–H_ = 564 Hz, 1H), 4.11 (q, *J* = 7.3 Hz, 2H), 2.84–2.75 (m, 1H), 2.58 (app t, *J* = 7.0 Hz, 2H), 2.22–2.12 (m, 1H), 1.81–1.56 (m, 5H), 1.21 (t, *J* = 7.1 Hz, 3H). ^13^C NMR (151 MHz, CDCl_3_): δ 175.4 (d, *J* = 6.0 Hz), 162.6, 142.1, 128.3, 128.3, 128.3, 125.7, 60.4, 39.8, 36.5, 35.6, 34.5, 33.8 (d, *J* = 10.1 Hz), 32.5 (d, *J* = 92.1 Hz), 31.4, 29.7, 28.7, 14.2. MS (ESI) *m*/*z*: calcd for C_14_H_20_O_4_P^–^ [M – H]^−^ 283.1; found, 283.1.

#### General Procedure for the Synthesis of Symmetrical Phosphinic Acids **7a–e**

In an oven-dried flask, the phosphinic acids **6a–e** (1.0 equiv) in dry DCM (2 mL for 1 mmol of the phosphinic acid) were added, followed by the addition of Et_3_N (7.0 equiv) and acrylate **2a–e** (1.2 equiv). The mixture was cooled to 0 °C and purged with N_2_ for 15 min. Subsequently, TMSCl (7.0 equiv) was added to the reaction mixture dropwise over 15 min. The temperature was slowly increased to rt, and the clear solution was stirred for 48–72 h (or until completion, monitored by ^31^P NMR). MeOH (1 mL for 1 mmol) was added dropwise and stirring was continued at rt for 30 min. Removal of volatiles under vacuum afforded the crude product as a pale-yellow viscous oil. The crude product was dissolved in EtOAc, and the resulting solution was washed with 1 M HCl (three times) and brine, dried over Na_2_SO_4_, and concentrated in vacuo. The residue was purified by column chromatography using DCM/MeOH/AcOH 7/0.5/0.1–0.5 as the eluent.

##### Bis(3-ethoxy-3-oxopropyl)phosphinic Acid (**7a**)

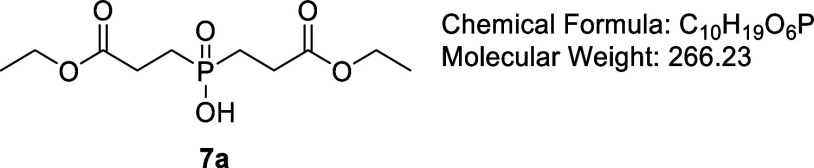



Yellow solid (99%), mp: 58 °C. *R*_*f*_ (DCM/MeOH/AcOH = 7/0.5/0.5) = 0.33. ^1^H NMR (400 MHz, CDCl_3_, δ): 4.12(q, *J* = 7.2 Hz, 4H), 2.63–2.56 (m, 4H), 2.02 (dt, *J* = 14.7, 8.1 Hz, 4H), 1.23 (t, *J* = 7.2 Hz, 6H). ^13^C NMR (101 MHz, CDCl_3_): δ 172.1 (d, *J* = 16.0 Hz), 61.0, 26.5 (d, *J* = 3.0 Hz), 24.0 (d, *J* = 94.9 Hz), 14.1. ^31^P NMR (162 MHz, CDCl_3_): δ 55.73. HRMS (ESI) *m*/*z*: calcd for C_10_H_19_O_6_P [M + H]^+^ 267.0992; found, 267.0993.

##### Bis(3-ethoxy-2-methyl-3-oxopropyl)phosphinic Acid (**7b**)

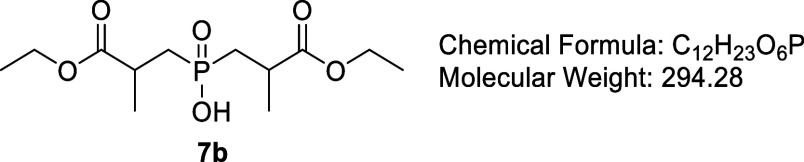



Pale-yellow viscous oil (96%); *R*_*f*_ (DCM/MeOH/AcOH = 7/0.5/0.5) = 0.45. ^1^H NMR (600 MHz, CDCl_3_, δ): 4.17–4.13 (m, 4H), 2.93–2.82 (m, 2H), 2.28 (dddd, *J* = 19.2, 11.7, 7.4, 4.8 Hz, 2H), 1.89–1.78 (m, 2H), 1.35–1.30 (m, 6H), 1.27 (td, *J* = 7.1, 2.7 Hz, 6H). ^13^C NMR (101 MHz, CDCl_3_): δ 175.3 (d, *J* = 9.1 Hz), 61.49, 34.04 (d, *J* = 3.0 Hz), 33.3 (d, *J* = 95.95 Hz), 19.1, 18.9, 14.5. ^31^P NMR (243 MHz, CDCl_3_): δ 55.86, 55.73. HRMS (ESI) *m*/*z*: calcd for C_12_H_23_O_6_PNa [M + Na]^+^ 295.1305; found, 295.1306.

##### Bis(2-(ethoxycarbonyl)-4-methylpentyl)phosphinic Acid (**7c**)

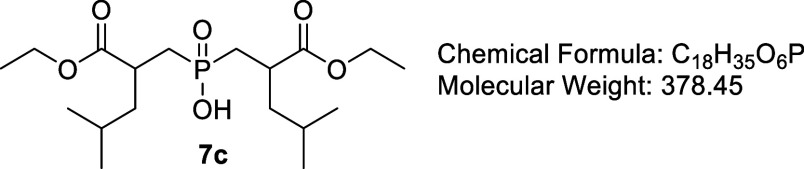



Pale-yellow viscous oil (98%); *R*_*f*_ (DCM/MeOH/AcOH = 7/0.5/0.5) = 0.6. ^1^H NMR (400 MHz, CDCl_3_, δ): 4.11 (qd, *J* = 7.3, 2.8 Hz, 4H), 2.81–2.76 (m, 2H), 2.12 (qd, *J* = 13.6, 6.8 Hz, 2H), 1.75 (tdd, *J* = 15.0, 7.2, 4.3 Hz, 1H), 1.59 (dd, *J* = 13.7, 5.2 Hz, 1H), 1.54–1.49 (m, 1H), 1.35 (dt, *J* = 13.0, 6.7 Hz, 1H), 1.23 (t, *J* = 7.2 Hz, 6H), 0.87 (dd, *J* = 16.5, 6.2 Hz, 12H). ^13^C NMR (101 MHz, CDCl_3_): δ 175.3 (d, *J* = 6.0 Hz), 60.8, 43.4, 37.4 (d, *J* = 2.0 Hz), 32.0 (d, *J* = 91.9 Hz), 31.9 (d, *J* = 91.9 Hz), 25.8, 22.8, 21.9, 14.1. ^31^P NMR (162 MHz, CDCl_3_): δ 55.32, 5522. HRMS (ESI) *m*/*z*: calcd for C_18_H_35_O_6_PNa [M + Na]^+^ 401.2060; found, 401.2060.

##### Bis(2-benzyl-3-ethoxy-3-oxopropyl)phosphinic Acid (**7d**)

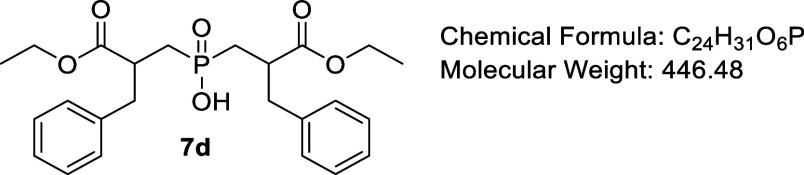



Pale-yellow viscous oil (92%); *R*_*f*_ (DCM/MeOH/AcOH = 7/0.5/0.5) = 0.82. ^1^H NMR (600 MHz, CDCl_3_, δ): 7.29–7.22 (m, 4H), 7.22–7.17 (m, 2H), 7.17–7.09 (m, 4H), 4.08–3.96 (m, 4H), 3.05 (s, 2H), 2.94 (app q, *J* = 10.5 Hz, 4H), 2.18–2.11 (m, 2H), 1.81 (td, *J* = 13.9, 6.5 Hz, 2H), 1.10 (td, *J* = 7.2, 2.7 Hz, 6H). ^13^C NMR (101 MHz, CDCl_3_): δ 174.1 (d, *J* = 6.0 Hz), 137.9, 129.2, 128.4, 126.7, 60.8, 41.0 (d, *J* = 7.0 Hz), 39.7 (d, *J* = 11.1 Hz), 31.1 (d, *J* = 93.9 Hz), 14.0. ^31^P NMR (162 MHz, CDCl_3_): δ 56.28, 56.19. HRMS (ESI) *m*/*z*: calcd for C_24_H_31_O_6_PNa [M + Na]^+^ 469.1750; found, 469.1746.

##### Bis(2-(ethoxycarbonyl)-5-phenylpentyl)phosphinic Acid (**7e**)

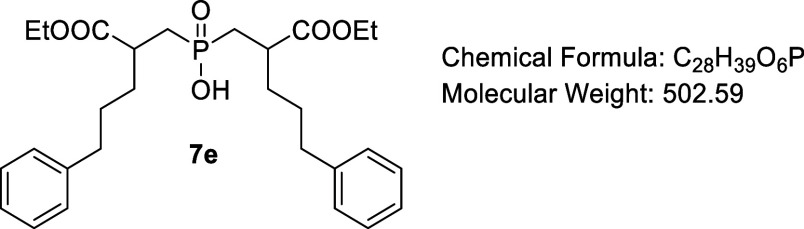



Pale-yellow oil (94%); *R*_*f*_ (DCM/MeOH/AcOH = 7/0.5/0.5) = 0.91. ^1^H NMR (400 MHz, CDCl_3_, δ): 7.24 (t, *J* = 7.4 Hz, 4H), 7.17–7.11 (m, 6H), 4.11 (q, *J* = 7.3 Hz, 4H), 2.80 (ddt, *J* = 12.3, 8.1, 4.2 Hz, 2H), 2.58 (t, *J* = 7.0 Hz, 4H), 2.18 (ddd, *J* = 19.6, 13.3, 9.7 Hz, 2H), 1.81–1.56 (m, 10H), 1.21 (t, *J* = 7.1 Hz, 6H). ^13^C NMR (151 MHz, CDCl_3_): δ 174.4 (d, *J* = 7.5 Hz), 161.6, 141.1, 127.3, 127.3, 127.2, 124.7, 59.4, 38.8, 35.5, 34.6, 33.5, 32.9 (d, *J* = 16.6 Hz), 32.8 (d, *J* = 10.6 Hz), 31.5 (d, *J* = 90.1 Hz), 28.7, 27.66, 13.2. ^31^P NMR (162 MHz, CDCl_3_): δ 56.66, 56.59. HRMS (ESI) *m*/*z*: calcd for C_28_H_39_O_6_P [M + H]^+^ 503.2557; found, 503.2554.

#### General Procedure for the Preparation of Compounds **9a–g**

A mixture of the phosphinic acid **3a**, **3b**, **7a–e** (0.6 mmol), diacetone-d-galactose (0.5 mmol), TBTU (0.6 mmol), and Et_3_N (1 mmol) was dissolved in DCE under a N_2_ atmosphere, stirred at rt for 6–12 h or at 75 °C for 3 h. After completion of the reaction, monitored by TLC, the solvent was removed under reduced pressure, and the residue was dissolved in DCM. The crude product was washed with H_2_O (2 × 15 mL), then treated with brine, dried with Na_2_SO_4_, and concentrated to afford the product in almost pure form. However, further purification was achieved by passing the crude product through a short silica gel column using hexane/EtOAc (1/1–5/1) as an eluent.

##### Ethyl 3-(Phenyl(((3a*S*,5*S*,5a*R*,8a*R*,8b*S*)-2,2,7,7-tetramethyltetrahydro-5*H*-bis([1,3]dioxolo)[4,5-*b*:4′,5′-*d*]pyran-5-yl)methoxy)phosphoryl)propanoate (**9a**)

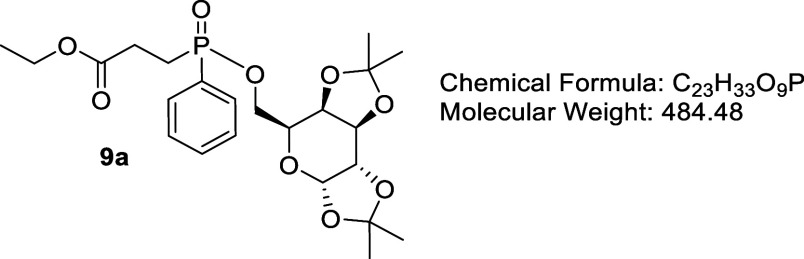



Pale-yellow oil (96%); *R*_*f*_ (EtOAc/hexane = 7/3) = 0.41. ^1^H NMR (600 MHz, CDCl_3_, δ): 7.77–7.72 (m, 2H), 7.52–7.49 (m, 1H), 7.44–7.41 (m, 2H), 5.47 (dd, *J* = 18.7, 5.0 Hz, 1H), 4.57–4.53 (m, 1H), 4.27–4.25 (m, 1H), 4.16 (dd, *J* = 7.9, 1.9 Hz, 1H), 4.14–4.06 (m, 1H), 4.05–4.00 (m, 3H), 3.88 (ddd, *J* = 11.1, 9.7, 7.8 Hz, 1H), 2.63–2.43 (m, 2H), 2.30–2.12 (m, 2H), 1.51 (s, 2H), 1.44 (s, 1H), 1.35 (s, 1H), 1.32 (s, 2H), 1.29–1.26 (m, 4H), 1.23 (s, 2H), 1.15 (td, *J* = 7.1, 1.8 Hz, 3H). ^13^C NMR (101 MHz, CDCl_3_): δ 172.1 (d, *J* = 19.19 Hz), 132.6, 132.5, 132.54, 131.8, 131.7, 131.7, 131.6, 130.4, 128.8, 128.7, 128.6, 109.2 (d, *J* = 69.7 Hz), 109.1 (d, *J* = 70.7 Hz), 96.21 (d, *J* = 6.06 Hz), 70.8, 70.6, 70.6, 70.5, 70.4, 67.7, 67.7, 66.9, 66.8, 63.7, 63.6, 63.1, 63.0, 60.8, 60.7, 26.8, 26.6, 26.6, 25.9, 25.9, 25.8, 25.8, 25.6, 25.5, 24.9, 24.9, 24.6, 24.5, 24.3, 14.1. ^31^P NMR (162 MHz, CDCl_3_): δ 44.84, 44.52. HRMS (ESI) *m*/*z*: calcd for C_23_H_33_O_9_PNa [M + Na]^+^ 507.1754; found, 507.1743.

##### Ethyl 2-Benzyl-3-(phenyl(((3a*S*,5*S*,5a*R*,8a*R*,8b*S*)-2,2,7,7-tetramethyltetrahydro-5*H*-bis([1,3]dioxolo)[4,5 *b*:4′,5′-*d*]pyran-5-yl)methoxy)phosphoryl)propanoate (**9b**)

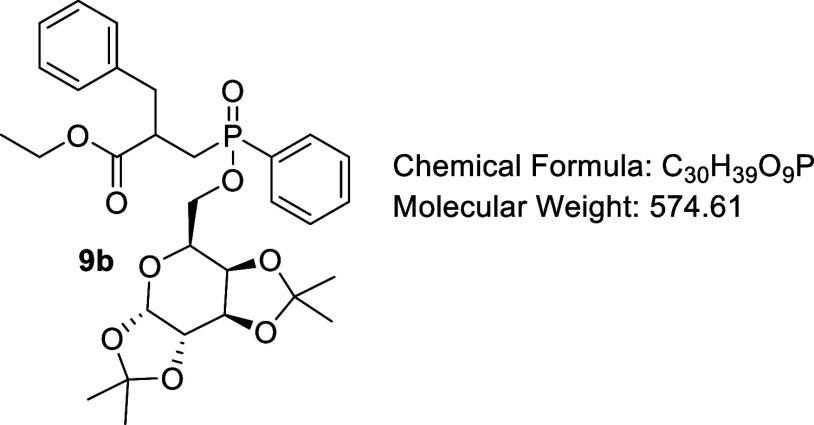



Pale-yellow oil (85%); *R*_*f*_ (EtOAc/hexane = 7/3) = 0.54. ^1^H NMR (400 MHz, CDCl_3_, δ): 7.78–7.71 (m, 2H), 7.54–7.41 (m, 3H), 7.24–7.06 (m, 5H), 5.48 (dt, *J* = 11.2, 3.7 Hz, 1H), 4.57 (td, *J* = 7.5, 2.5 Hz, 1H), 4.31–4.28 (m, 1H), 4.21–4.15 (m, 1H), 4.07–3.91 (m, 3H), 3.83–3.71 (m, 2H), 3.09–3.00 (m, 1H), 2.99–2.87 (m, 2H), 2.49–2.32 (m, 1H), 2.19–2.09 (m, 1H), 1.50–1.45 (m, 3H), 1.37–1.23 (m, 12H). ^13^C NMR (101 MHz, CDCl_3_): δ 173.8 (d, *J* = 7.1 Hz) 137.9, 132.5, 132.5, 132.5, 132.0, 131.9, 131.9, 131.9, 131.8, 131.7, 129.2, 129.1, 129.0, 128.7, 128.6, 128.6, 128.6, 128.6, 128.5, 128.5, 128.4, 128.4, 128.4, 128.3, 128.3, 126.6, 126.6, 109.5, 109.5, 109.9, 108.8, 108.7, 96.2, 96.1, 70.8, 70.7, 70.6, 70.5, 70.4, 70.3, 67.7, 67.6, 67.5, 67.4, 66.8, 66.7, 65.5, 63.6, 63.5, 63.5, 62.9, 62.8, 62.8, 60.7, 60.5, 60.5, 41.1, 41.0, 40.9, 39.5, 39.5, 39.4, 38.6, 31.5, 30.5, 30.5, 29.7, 26.0, 25.9, 25.9, 25.8, 24.9, 24.9, 24.4, 24.3, 21.0, 19.2, 13.8. ^31^P NMR (162 MHz, CDCl_3_): δ 44.07, 43.84, 43.59, 43.36. HRMS (ESI) *m*/*z*: calcd for C_30_H_39_O_9_PNa [M + Na]^+^ 597.2223; found, 597.2211.

##### Ethyl 3-((3-Ethoxy-3-oxopropyl)(((3a*R*,5*R*,5a*S*,8a*S*,8b*R*)-2,2,7,7-tetramethyltetrahydro-5*H*-bis([1,3]dioxolo)[4,5-*b*:4′,5′-*d*]pyran-5-yl)methoxy)phosphoryl) Propanoate (**9c**)

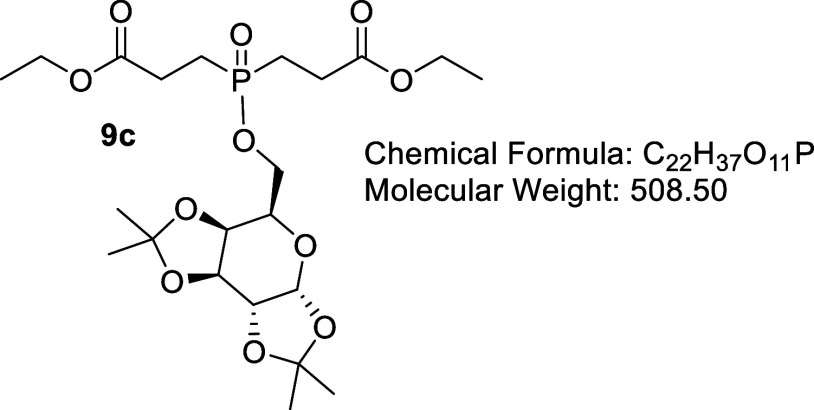



Pale-yellow viscous oil (95%); *R*_*f*_ (EtOAc/hexane = 7/3) = 0.33. ^1^H NMR (400 MHz, CDCl_3_, δ): 5.48 (d, *J* = 5.0 Hz, 1H), 4.57 (dd, *J* = 8.0, 2.5 Hz, 1H), 4.28 (dd, *J* = 5.1, 2.4 Hz, 1H), 4.18–4.16 (m, 1H), 4.10 (q, *J* = 7.0 Hz, 6H), 3.96–3.92 (m, 1H), 2.66–2.53 (m, 4H), 2.11–2.00 (m, 4H), 1.50 (s, 3H), 1.39 (s, 3H), 1.28 (s, 6H), 1.21 (td, *J* = 7.1, 1.7 Hz, 6H). ^13^C NMR (101 MHz, CDCl_3_): δ 172.3, 172.0, 109.6, 108.8, 96.2, 70.7, 70.6, 67.4, 67.3, 63.5, 63.4, 60.9, 60.9, 38.6, 29.7, 26.8, 26.7, 26.5, 26.4, 25.9, 25.9, 24.9, 24.4, 24.0, 23.9, 23.1, 22.9, 14.2. ^31^P NMR (162 MHz, CDCl_3_): δ 56.30. HRMS (ESI) *m*/*z*: calcd for C_22_H_37_O_11_P [M + H]^+^ 509.2146; found, 509.2150.

##### Ethyl 3-((3-Ethoxy-2-methyl-3-oxopropyl)(((3a*R*,5*R*,5a*S*,8a*S*,8b*R*)-2,2,7,7-tetramethyltetrahydro-5*H*-bis([1,3]dioxolo)[4,5-*b*:4′,5′-*d*]pyran-5-yl)methoxy)phosphoryl)-2-methylylpropanoate (**9d**)

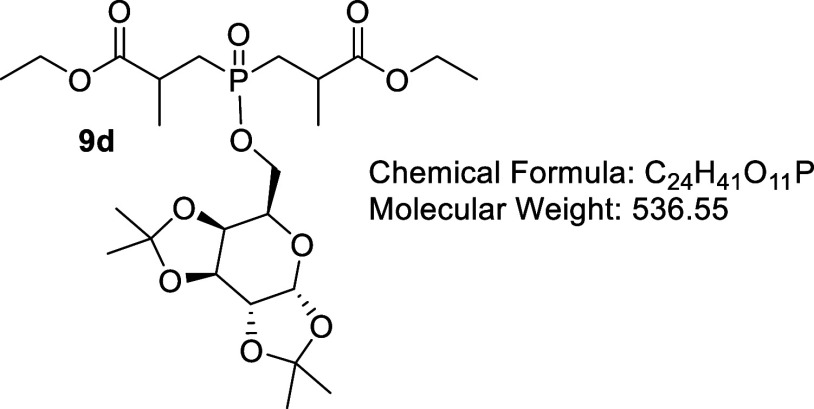



Pale-yellow viscous oil (92%); *R*_*f*_ (EtOAc/hexane = 7/3) = 0.37. ^1^H NMR (600 MHz, CDCl_3_, δ): 5.45 (d, *J* = 5.7 Hz, 1H), 4.54 (dd, *J* = 8.0, 2.5 Hz, 1H), 4.25 (dd, *J* = 5.2, 2.6 Hz, 1H), 4.15 (d, *J* = 2.2 Hz, 1H), 4.11–4.02 (m, 5H), 4.01–3.95 (m, 1H), 3.94–3.91 (m, 1H), 2.88–2.78 (m, 2H), 2.30–2.14 (m, 2H), 1.82–1.69 (m, 2H), 1.46 (s, 3H), 1.36 (s, 3H), 1.27–1.23 (m, 12H), 1.19 (td, *J* = 7.2, 2.7 Hz, 6H). ^13^C NMR (151 MHz, CDCl_3_): δ 175.4, 109.5, 108.7, 96.2, 70.66, 70.4, 67.4, 67.3, 60.8, 60.3, 38.6, 34.0, 34.0, 33.9, 33.9, 33.8, 33.8, 33.7, 33.7, 25.9, 25.9, 24.9, 24.4, 21.0, 19.1, 19.5, 14.1. ^31^P NMR (243 MHz, CDCl_3_): δ 54.95, 54.74, 54.61, 54.30. HRMS (ESI) *m*/*z*: calcd for C_24_H_41_O_11_P [M + H]^+^ 537.2459; found, 537.2459.

##### Ethyl 2-(((2-(Ethoxycarbonyl)-4-methylpentyl)(((3a*R*,5*R*,5a*S*,8a*S*,8b*R*)-2,2,7,7-tetramethyltetrahydro-5*H*-bis([1,3]dioxolo)[4,5-*b*:4′,5′-*d*]pyran-5-yl)methoxy)phosphoryl)methyl)-4-methylpentanoate (**9e**)

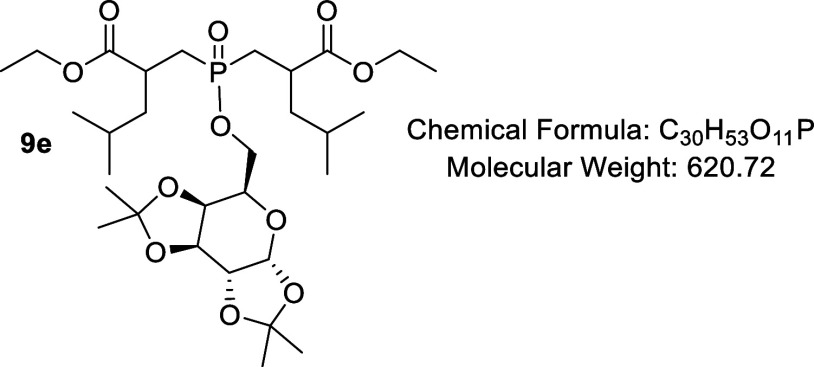



Pale-yellow viscous oil (75%); *Rf* (EtOAc/hexane = 7/3) = 0.45. ^1^H NMR (600 MHz, CDCl_3_, δ): 5.53–5.46 (m, 1H), 4.57 (ddd, *J* = 8.4, 5.1, 2.1 Hz, 1H), 4.30–4.26 (m, 1H), 4.19–4.17 (m, 1H), 4.14–4.06 (m, 5H), 3.94 (t, *J* = 5.8 Hz, 1H), 3.85–3.78 (m, 1H), 2.83–2.78 (m, 2H), 2.20–2.05 (m, 2H), 1.88–1.72 (m, 2H), 1.59–1.54 (m, 2H), 1.49 (app d, *J* = 4.5 Hz, 6H), 1.34 (m, 1H), 1.29 (app d, *J* = 9.1 Hz, 9H), 1.22 (ddd, *J* = 7.1, 5.7, 4.0 Hz, 6H), 0.88 (dd, *J* = 6.7, 2.7 Hz, 6H), 0.84 (d, *J* = 6.5 Hz, 6H). ^13^C NMR (101 MHz, CDCl_3_): δ 175.2 (d, *J* = 5.1 Hz, 1H), 109.4, 109.3, 108.7, 108.6, 96.2, 71.5, 70.7, 70.7, 70.6, 70.6, 70.5, 70.4, 68.1, 67.3, 67.2, 67.1, 67.0, 63.2, 63.1, 63.1, 63.0, 63.0, 62.9, 62.8, 62.2, 60.7, 60.6, 60.6, 60.5, 60.33, 43.4, 43.4, 43.3, 43.3, 37.5, 37.4, 37.4, 26.0, 25.9, 25.9, 25.9, 25.8, 24.9, 24.5, 24.4, 24.4, 24.3, 22.9, 22.8, 22.8, 21.9, 21.9, 21.8, 21.8, 20.9, 14.2, 14.1. ^31^P NMR (162 MHz, CDCl_3_): δ 54.42, 54.11, 54.06, 53.62. HRMS (ESI) *m*/*z*: calcd for C_30_H_53_O_11_P [M + H]^+^ 621.3398; found, 621.3405.

##### Ethyl 2-Benzyl-3-((2-benzyl-3-ethoxy-3-oxopropyl)(((3a*R*,5*R*,5a*S*,8a*S*,8b*R*)-2,2,7,7-tetramethyltetrahydro-5*H*-bis([1,3]dioxolo)[4,5-*b*:4′,5′-*d*]pyran-5-yl)methoxy)phosphoryl)propanoate (**9f**)

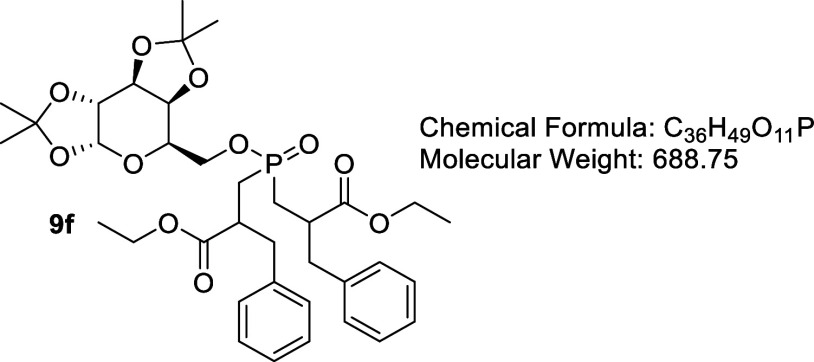



Pale-yellow viscous oil (88%); *R*_*f*_ (EtOAc/hexane = 7/3) = 0.75. ^1^H NMR (600 MHz, CDCl_3_, δ): 7.26 (dtd, *J* = 7.7, 5.5, 2.7 Hz, 4H), 7.21–7.18 (m, 2H), 7.16–7.13 (m, 4H), 5.53–5.49 (m, 1H), 4.59 (dq, *J* = 8.0, 1.8 Hz, 1H), 4.31 (td, *J* = 5.2, 2.4 Hz, 1H), 4.18 (ddt, *J* = 11.7, 7.9, 2.0 Hz, 1H), 4.10–4.00 (m, 6H), 3.99–3.95 (m, 1H), 3.10–3.02 (m, 2H), 2.98–2.87 (m, 4H), 2.27–2.07 (m, 2H), 1.92–1.77 (m, 2H), 1.52 (s, 1H), 1.49 (d, *J* = 3.2 Hz, 2H), 1.42 (dd, *J* = 7.7, 3.7 Hz, 3H), 1.32–1.30 (m, 6H), 1.13–1.08 (m, 6H). ^13^C NMR (151 MHz, CDCl_3_): δ 174.11 (d, *J* = 3.02 Hz, 1H), 137.9, 129.2, 129.1, 128.4, 128.3, 128.3, 126.7, 109.5, 108.7, 96.2, 70.7, 70.4, 67.3, 60.8, 60.7, 41.1, 41.0, 39.9, 39.8, 25.9, 24.9, 24.4, 24.4, 14.0. ^31^P NMR (243 MHz, CDCl_3_): δ 54.56, 54.21, 53.71. HRMS (ESI) *m*/*z*: calcd for C_36_H_49_O_11_P [M + H]^+^ 689.3085; found, 689.3075.

##### Ethyl 2-(((2-(Ethoxycarbonyl)-5-phenylpentyl)(((3a*R*,5*R*,5a*S*,8a*S*,8b*R*)-2,2,7,7-tetramethyltetrahydro-5*H*-bis([1,3]dioxolo)[4,5-*b*:4′,5′-*d*]pyran-5-yl)methoxy)phosphoryl)methyl)-5-phenylpentanoate (**9g**)

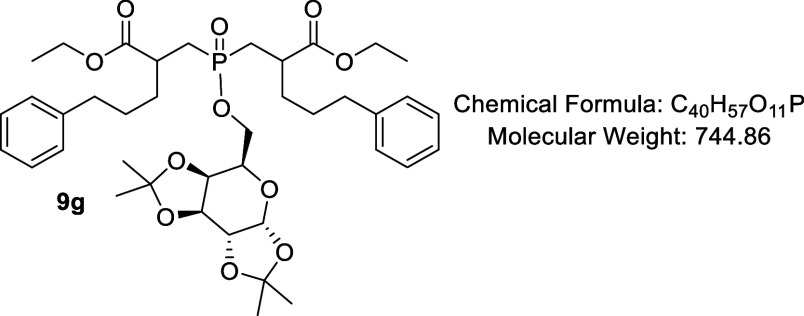



Pale-yellow viscous oil (90%); *R*_*f*_ (EtOAc/hexane = 7/3) = 0.82. ^1^H NMR (600 MHz, CDCl_3_, δ): 7.26–7.23 (m, 4H), 7.17–7.12 (m, 6H), 5.47 (ddd, *J* = 10.0, 5.0, 1.5 Hz,1H), 4.59–4.57 (m, 1H), 4.29–4.28 (m, 1H), 4.19–4.17 (m, 1H), 4.15–4.09 (m, 4H), 4.09–4.03 (m, 2H), 3.97–3.95 (m, 1H), 2.83–2.79 (m, 2H), 2.61–2.58 (m, 4H), 2.26–2.09 (m, 2H), 1.87–1.77 (m, 2H), 1.71–1.65 (m, 4H), 1.63–1.57 (m, 4H), 1.50 (s, 3H), 1.40 (s, 3H), 1.29 (s, 6H), 1.24–1.21 (m, 6H). ^13^C NMR (151 MHz, CDCl_3_): δ 173.7 (d, *J* = 6.0 Hz), 140.9, 127.3, 127.3, 124.8, 108.5, 107.7, 95.2, 69.7, 69.4, 59.8, 59.70, 38.0, 37.9, 34.5, 32.5, 32.5, 27.5, 27.5, 27.4, 25.0, 24.9, 23.9, 23.5, 23.4, 13.2. ^31^P NMR (243 MHz, CDCl_3_): δ 54.81, 54.39, 54.35, 53.90. HRMS (ESI) *m*/*z*: calcd for C_40_H_57_O_11_P [M + H]^+^ 745.3711; found, 745.3711.

##### Ethyl 3-((1-(((Benzyloxy)carbonyl)amino)-2-phenylethyl)(((3a*R*,5*R*,5a*S*,8a*S*,8b*R*)-2,2,7,7-tetramethyltetrahydro-5*H*-bis([1,3]dioxolo)[4,5-*b*:4′,5′-*d*]pyran-5-yl)methoxy)phosphoryl)-2-methylpropanoate (**9h**)

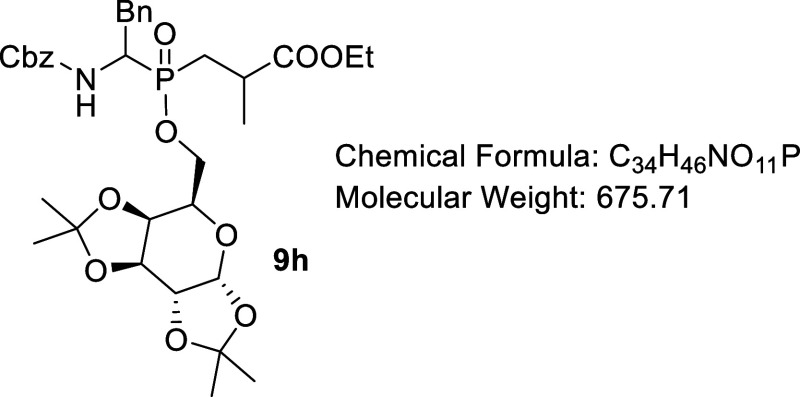



White solid (75%); *R*_*f*_ (EtOAc/hexane = 7/3) = 0.73. ^1^H NMR (600 MHz, CDCl_3_, δ): 7.30–7.14 (m, 10H), 5.61–5.52 (m, 1H), 5.02–4.90 (m, 2H), 4.61 (dd, *J* = 10.7, 6.8 Hz, 1H), 4.39–4.29 (m, 2H), 4.26–4.18 (m, 1H), 4.17–4.08 (m, 6H), 4.03 (dt, *J* = 13.8, 9.0 Hz, 1H), 3.44–3.19 (m, 1H), 3.01–2.92 (m, 1H), 2.91–2.79 (m, 1H), 2.72–2.60 (m, 2H), 2.20–2.10 (m, 2H), 2.03 (s, 1H), 1.54–1.50 (m, 1H), 1.45–1.41 (m, 4H), 1.31–1.28 (m, 5H), 5.26–1.22 (m, 5H). ^13^C NMR (151 MHz, CDCl_3_): δ 172.1, 156.1, 136.4, 129.2, 129.2, 129.1, 128.4, 128.3, 127.9, 127.8, 127.6, 126.7, 109.9, 108.8, 96.3, 70.8, 70.8, 70.7, 70.7, 70.6, 70.4, 66.8, 60.8, 60.3, 50.8, 33.9, 26.4, 25.9, 25.9, 24.9, 24.8, 24.4, 21.0, 14.2. ^31^P NMR (243 MHz, CDCl_3_): δ 53.80, 53.70, 52.83, 52.79. HRMS (ESI) *m*/*z*: calcd for C_34_H_46_NO_11_P [M + Na]^+^ 698.2700; found, 698.2698.

##### Ethyl 2-Benzyl-3-((2-benzyl-3-ethoxy-3-oxopropyl)(((2*R*,3*R*,4*S*,5*R*,6*S*)-3,4,5,6-tetrahydroxytetrahydro-2*H*-pyran-2-yl)methoxy)phosphoryl)propanoate (**10**)

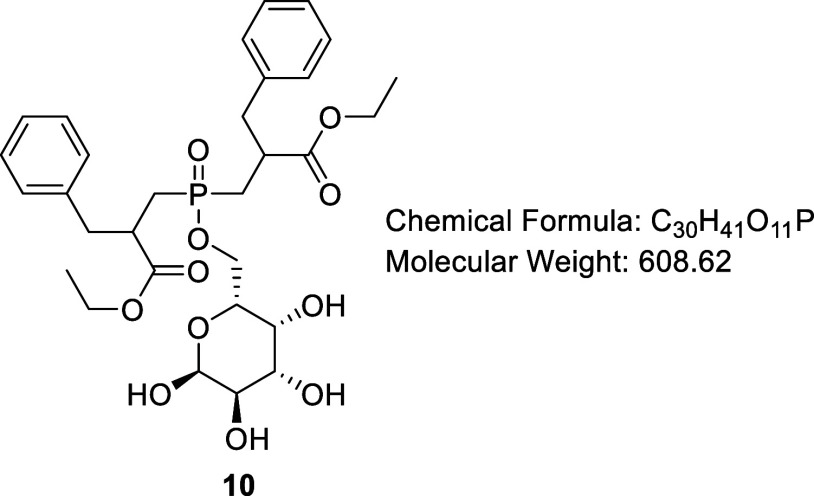



To the stirring mixture of protected ester **9f** (0.2 mmol) in 2 mL of DCM/H_2_O (2 mL/1 mL), 1 mL of trifluoroacetic acid was added, and the resulting mixture was stirred at rt for 24 h. Solvents were removed in vacuo, and the residue was dissolved in DCM and washed with 1 M HCl followed by brine, dried with Na_2_SO_4,_ and concentrated to afford **10** as a pale-yellow viscous oil (70%); *R*_*f*_ (MeOH/DCM = 1/9) = 0.35. ^1^H NMR (600 MHz, CDCl_3_, δ): 7.28–7.22 (m, 4H), 7.21–7.17 (m, 2H), 7.10 (dp, *J* = 19.9, 6.9 Hz, 4H), 5.79–5.76 (m, 3H), 5.29–5.25 (m, 1H), 4.37 (dd, *J* = 93.1, 7.4 Hz, 1H), 4.19–4.08 (m, 1H), 4.08–3.95 (m, 5H), 3.94–3.88 (m, 1H), 3.87–3.83 (m, 1H), 3.70–3.53 (m, 1H), 3.08–2.88 (m, 5H), 2.87–2.75 (m, 2H), 2.29–2.07 (m, 2H), 1.89–1.68 (m, 2H), 1.14–1.03 (m, 6H). ^13^C NMR (151 MHz, CDCl_3_): δ 173.1, 136.6, 136.5, 128.1, 128.1, 128.1, 127.5, 127.5, 127.5, 127.4, 125.8, 125.8, 60.1, 60.1, 60.1, 52.4, 40.3, 40.2, 40.1, 38.9, 38.8, 37.6, 28.7, 12.9. ^31^P NMR (162 MHz, CDCl_3_): δ 59.51, 59.35, 59.15, 59.00, 58.99, 58.89, 58.79, 58.73, 58.56, 58.41, 58.26, 58.14, 58.07, 57.89. HRMS (ESI) *m*/*z*: calcd for C_30_H_41_O_11_PNa [M + Na]^+^ 631.2278; found, 631.2279.

#### General Procedure for the Preparation of Compounds **12a–g**

To a mixture of the phosphinic acid **3a**, **3b**, **6a**, **6c**, **6d**, **RXP03** (0.6 mmol), and 3-hydroxyflavone (0.5 mmol), 1 mL dry DMF was added followed by the addition TEA (1 mmol) and DIC (0.6 mmol) under N_2_. The mixture was stirred at rt for 12 h or 80 °C for 3 h. The reaction mixture was diluted with Et_2_O (15 mL), washed with H_2_O (2 × 15 mL), brine, dried with Na_2_SO_4_, and concentrated to afford the pure product.

##### Ethyl 3-((4-Oxo-2-phenyl-4*H*-chromen-3-yl)oxy)((phenyl)phosphoryl)propanoate (**12a**)

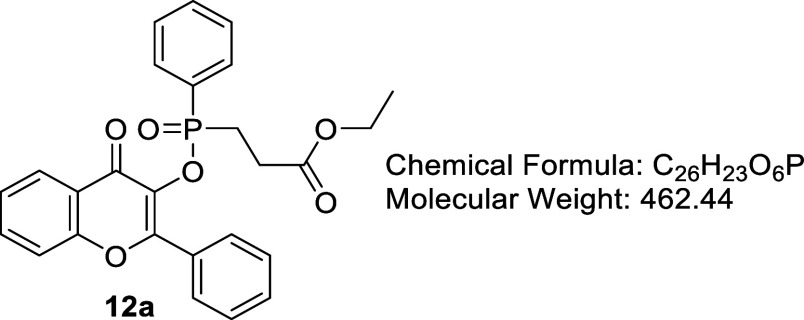



Pale-yellow viscous oil (95%); *R*_*f*_ (EtOAc/hexane = 7/3) = 0.55. ^1^H NMR (400 MHz, CDCl_3_, δ): 8.27–8.24 (m, 1H), 7.79–7.67 (m, 5H), 7.55–7.50 (m, 2H), 7.41 (tt, *J* = 7.6, 4.5 Hz, 4H), 7.33–7.28 (m, 2H), 4.06 (qd, *J* = 7.2, 1.4 Hz, 2H), 2.80–2.68 (m, 1H), 2.63–2.47 (m, 3H), 1.20 (t, *J* = 7.1 Hz, 3H). ^13^C NMR (101 MHz, CDCl_3_): δ 173.4, 172.0 (d, *J* = 13.6 Hz), 156.6, 156.6, 155.4, 133.9, 133.9, 132.4, 132.4, 131.4, 130.9, 130.9, 130.8, 130.1, 130.0, 128.9, 128.4, 128.3, 128.2, 126.1, 125.0, 123.7, 118.0, 60.8, 41.9, 27.0, 26.2 (d, *J* = 97.8 Hz), 23.5, 14.1. ^31^P NMR (162 MHz, CDCl_3_): δ 47.06. HRMS (ESI) *m*/*z*: calcd for C_26_H_23_O_6_P [M + H]^+^ 463.1305; found, 463.1305.

##### Ethyl 2-Benzyl-3-(((4-oxo-2-phenyl-4*H*-chromen-3-yl)oxy)(phenyl)phosphoryl)propanoate (**12b**)

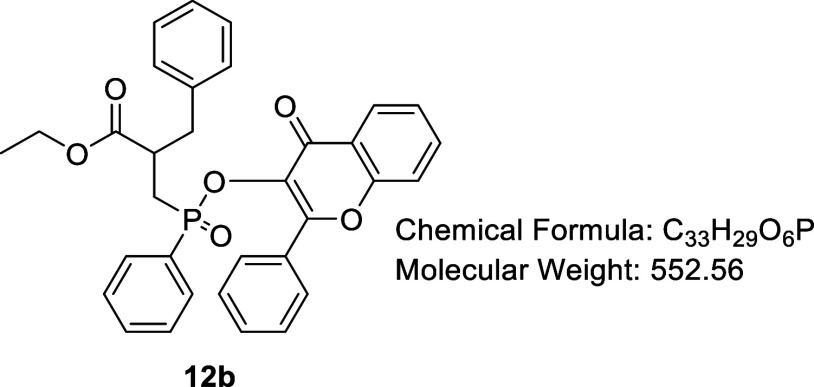



Pale-yellow viscous oil (80%); *R*_*f*_ (EtOAc/hexane = 7/3) = 0.65. ^1^H NMR (600 MHz, CDCl_3_, δ): 8.15–8.14 (m, 1H), 7.66–7.61 (m, 3H), 7.57–7.52 (m, 2H), 7.38 (t, *J* = 7.4 Hz, 2H), 7.29–7.23 (m, 4H),7.18–7.13 (m, 2H), 7.11–7.07 (m, 2H), 6.95 (dd, *J* = 21.6, 7.4 Hz, 2H), 3.82–3.76 (m, 1H), 3.60–3.55 (m, 1H), 3.47–3.42 (m, 1H), 2.95–2.89 (m, 2H), 2.84 (dd, *J* = 10.7, 3.9 Hz, 1H), 2.72–2.67 (m, 1H), 0.76 (t, *J* = 7.1 Hz, 3H). ^13^C NMR (151 MHz, CDCl_3_): δ 172.7, 172.6, 172.3, 172.2, 155.6, 155.5, 154.3, 136.9, 136.9, 132.8, 132.8, 131.3, 131.2, 129.8, 128.1, 128.0, 127.8, 127.8, 127.3, 127.2, 127.2, 127.1, 127.1, 125.5, 125.4, 125.1, 125.0, 123.9, 123.8, 122.6, 122.6, 116.9, 116.9, 59.6, 59.3, 40.4, 38.9, 38.8, 38.4, 38.3, 31.8, 31.1, 28.6, 22.4, 12.9, 12.7. ^31^P NMR (162 MHz, CDCl_3_): δ 46.29, 45.83. HRMS (ESI) *m*/*z*: calcd for C_33_H_29_O_6_P [M + H]^+^ 553.1774; found, 553.1767.

##### Ethyl 3-((3-Ethoxy-3-oxopropyl)((4-oxo-2-phenyl-4*H*-chromen-3-yl)oxy)phosphoryl)propanoate (**12c**)

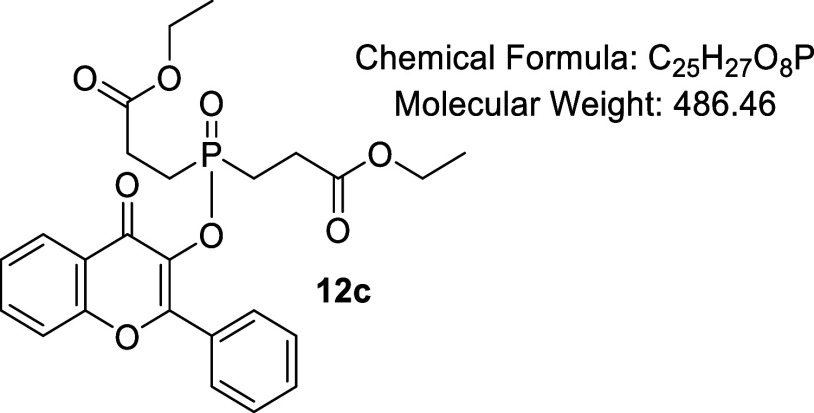



Pale-yellow viscous oil (92%); *R*_*f*_ (EtOAc/hexane = 7/3) = 0.35. ^1^H NMR (400 MHz, CDCl_3_, δ): 8.25–8.22 (m, 2H), 7.87–7.86 (m, 1H), 7.70–7.66 (m, 1H), 7.59–7.38 (m, 5H), 4.13–4.08 (m, 4H), 3.83–3.80 (m, 1H), 2.66–2.53 (m, 2H), 2.46–2.38 (m, 1H), 2.36–2.22 (m, 3H), 1.90 (q, *J* = 8.5 Hz, 1H), 1.22 (t, *J* = 6.9 Hz, 6H). ^13^C NMR (101 MHz, CDCl_3_): δ 173.5, 172.1 (d, *J* = 6.0 Hz), 157.2, 155.4, 134.1, 133.7, 131.3, 130.2, 128.9, 128.6, 127.8, 126.1, 125.5, 125.2, 124.5, 123.6, 118.1, 60.9, 41.9, 25.2 (d, *J* = 90.9 Hz), 24.7, 14.2. ^31^P NMR (162 MHz, CDCl_3_): δ 60.76. HRMS (ESI) *m*/*z*: calcd for C_25_H_27_O_8_P [M + H]^+^ 487.1516; found, 487.1509.

##### Ethyl 2-Benzyl-3-((2-benzyl-3-ethoxy-3-oxopropyl)((4-oxo-2-phenyl-4*H*-chromen-3-yl)oxy)phosphoryl)propanoate (**12d**)

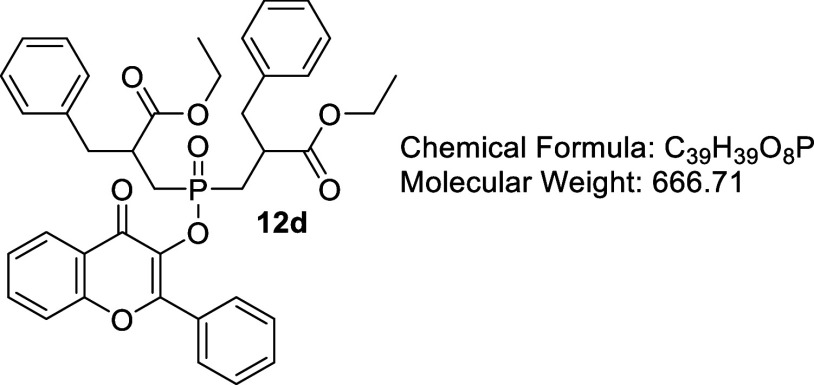



Pale-yellow viscous oil (90%); *R*_*f*_ (EtOAc/hexane = 7/3) = 0.60. ^1^H NMR (400 MHz, CDCl_3_, δ): 8.27 (q, *J* = 7.4 Hz, 1H), 7.99–7.89 (m, 1H), 7.72 (t, *J* = 7.6 Hz, 1H), 7.54 (t, *J* = 5.6 Hz, 3H), 7.44 (t, *J* = 7.5 Hz, 1H), 7.32–7.05 (m, 12H), 4.11–3.91 (m, 4H), 3.07 (td, *J* = 12.3, 6.0 Hz, 2H), 3.02–2.92 (m, 3H), 2.89 (q, *J* = 7.3 Hz, 1H), 2.49 (qd, *J* = 14.4, 9.0 Hz, 1H), 2.18 (dddd, *J* = 25.6, 15.4, 11.3, 6.5 Hz, 2H), 1.83 (t, *J* = 14.5 Hz, 1H), 1.15–1.06 (m, 6H). ^13^C NMR (151 MHz, CDCl_3_): δ 173.02 (d, *J* = 4.53 Hz), 172.1, 154.3, 136.9, 132.9, 130.2, 128.2, 128.1, 127.9, 127.6, 127.3, 127.2, 127.2, 125.6, 125.5, 125.0, 124.0, 116.9, 59.6, 40.3, 38.6, 30.8 (d, *J* = 89.9 Hz) 22.3, 12.9. ^31^P NMR (162 MHz, CDCl_3_): δ 59.08, 58.67, 57.83. HRMS (ESI) *m*/*z*: calcd for C_39_H_39_O_8_P [M + H]^+^ 667.2455; found, 667.2460.

##### Ethyl 2-(((2-(Ethoxycarbonyl)-5-phenylpentyl)((4-oxo-2-phenyl-4*H*-chromen-3-yl)oxy)phosphoryl)methyl)-5-phenylpentanoate (**12e**)

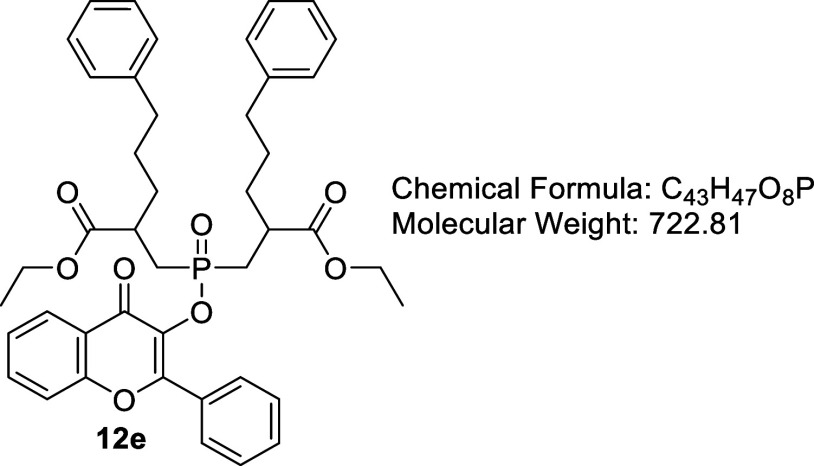



Pale-yellow viscous oil (88%); *R*_*f*_ (EtOAc/hexane = 7/3) = 0.85. ^1^H NMR (400 MHz, CDCl_3_, δ): 8.30 (dd, *J* = 16.6, 7.8 Hz, 1H), 7.67 (t, *J* = 7.8 Hz, 1H), 7.52 (ddd, *J* = 23.0, 14.7, 7.7 Hz, 2H), 7.34 (t, *J* = 7.5 Hz, 1H), 7.26 (t, *J* = 7.5 Hz, 6H), 7.18 (d, *J* = 7.2 Hz, 2H), 7.12 (d, *J* = 7.5 Hz, 6H), 4.13 (q, *J* = 6.7 Hz, 4H), 2.91–2.75 (m, 2H), 2.62–2.49 (m, 4H), 2.12–1.95 (m, 2H), 1.60 (s, 10H), 1.20 (t, *J* = 7.1 Hz, 6H). ^13^C NMR (101 MHz, CDCl_3_): δ 176.5 (d, *J* = 5.1 Hz), 176.4, 174.1, 157.3, 155.2, 142.1, 133.4, 131.5, 129.9, 128.5, 128.3, 128.3, 127.9, 125.7, 125.6, 124.3, 121.3, 118.1, 60.7, 42.0, 40.1, 40.1, 40.06, 35.7, 34.2, 34.1, 34.0, 33.9, 33.3, 32.4, 28.8 (d, *J* = 3.0 Hz), 23.5, 14.2. ^31^P NMR (162 MHz, CDCl_3_): δ 54.80, 54.45, 53.94.

##### Benzyl(1-((2-((1-amino-3-(1*H*-indol-3-yl)-1-oxopropan-2-yl)carbamoyl)-5-phenylpentyl)((4-oxo-2-phenyl-4*H*-chromen-3-yl)oxy)phosphoryl)-2-phenylethyl)carbamate (**12f**)

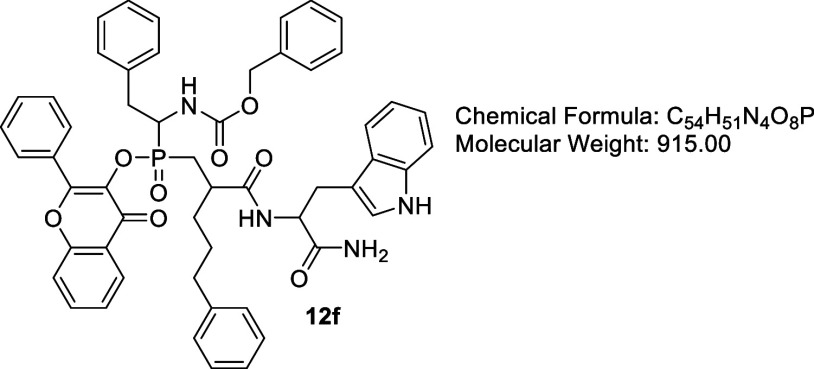



Pale-yellow viscous oil (78%); *R*_*f*_ (EtOAc/hexane = 7/3) = 0.3. ^13^C NMR (101 MHz, CDCl_3_): δ 177.2, 176.9, 173.6, 162.7, 157.6, 156.2, 155.3, 145.3, 141.6, 138.7, 136.3, 133.6, 131.2, 130.2, 128.9, 128.6, 128.5, 128.4, 128.4, 127.9, 127.8, 125.9, 125.4, 124.5, 124.0, 122.3, 120.8, 119.9, 118.4, 118.3, 111.9, 66.8, 58.9, 41.8, 36.5, 35.5, 31.4, 29.7, 28.3, 23.5. ^31^P NMR (162 MHz, CDCl_3_): δ 53.43. HRMS (ESI) *m*/*z*: calcd for C_54_H_51_N_4_O_8_P [M + H]^+^ 915.3517; found, 915.3512.

## Supporting Information Available

Copies of NMR and mass spectra for all the compounds (PDF)
