# Supplementary material for: Symmetrical Phosphinic Acids: Synthesis and Esterification Optimization toward Potential HIV Prodrugs
Source: ACS Omega. 2024 Sep 27;9(40):41742–57. doi: 10.1021/acsomega.4c05988 (PMC11465283; doi:10.1021/acsomega.4c05988)
Supplement: Supplementary file 1 — ao4c05988_si_001.pdf [file ao4c05988_si_001.pdf]

Symmetrical phosphinic acids: Synthesis and esterification optimization towards potential HIV prodrugs

Komal Hayat,[1,2] Gemma Nixon,[2] Qian Zhang,[1] and Magdalini Matziari[1,*]

[1] *Department of Chemistry, Xi'an Jiaotong-Liverpool University, 111 Ren'ai Road, SIP, Suzhou, Jiangsu Province, 215123, P. R. China*

[2] *Department of Chemistry, University of Liverpool, Crown Street, Liverpool, L69 7ZD, UK*

SUPPORTING INFORMATION

Copies of ^{1}HNMR, ^{13}C NMR, ^{31}P NMR, and Mass Spectra

Table of contents

Compound **3a** S5
Compound **3b** S7
Compound **6a** S9
Compound **6b** S10
Compound **6c** S12
Compound **6d** S13
Compound **6e** S15
Compound **7a** S16
Compound **7b** S18
Compound **7c** S20
Compound **7d** S22
Compound **7e** S24
Compound **9a** S26
Compound **9b** S28
Compound **9c** S30
Compound **9d** S32
Compound **9e** S34
Compound **9f** S36
Compound **9g** S38
Compound **9h** S40
Compound **10** S42
Compound **12a** S44
Compound **12b** S46
Compound **12c** S48
Compound **12d** S50
Compound **12e** S52
Compound **12f** S54

Table of Figures

Figure S1. ^{1}HNMR of compound **3a** S5

Figure S2. ^{13}CNMR of compound **3a** S5

Figure S3. ^{31}PNMR of compound **3a** S6

Figure S4. MS spectrum of compound **3a** S6

Figure S5. ^{1}HNMR of compound **3b** S7

Figure S6. ^{13}CNMR of compound **3b** S7

Figure S7. ^{31}PNMR of compound **3b** S8

Figure S8. MS spectrum of compound **3b** S8

Figure S9.^{1}HNMR of compound **6a** S9

Figure S10. ^{13}CNMR of compound **6a** S9

Figure S11. ^{31}PNMR of compound **6a** S10

Figure S12. ^{1}HNMR of compound **6b** S10

Figure S13. ^{13}CNMR of compound **6b** S11

Figure S14. ^{31}PNMR of compound **6b** S11

Figure S15. ^{1}HNMR of compound **6c** S12

Figure S16. ^{13}CNMR of compound **6c** S12

Figure S17. ^{31}PNMR of compound **6c** S13

Figure S18. ^{1}HNMR of compound **6d** S13

Figure S19. ^{13}CNMR of compound **6d** S14

Figure S20. ^{31}PNMR of compound **6d** S14

Figure S21. ^{1}HNMR of compound **6e** S15

Figure S22. ^{13}CNMR of compound **6e** S15

Figure S23. ^{31}PNMR of compound **6e** S16

Figure S24. ^{1}HNMR of compound **7a** S16

Figure S25. ^{13}CNMR of compound **7a** S17

Figure S26. ^{31}PNMR of compound **7a** S17

Figure S27. MS spectrum of compound **7a** S18

Figure S28. 1HNMR of compound **7b** S18

Figure S29. ^{13}CNMR of compound **7b** S19

Figure S30. ^{31}PNMR of compound **7b** S19

Figure S31. MS spectrum of compound **7b** S20

Figure S32. ^{1}HNMR of compound **7c** S20

Figure S33. ^{13}CNMR of compound **7c** S21

Figure S34. ^{31}PNMR of compound **7c** S21

Figure S35. MS spectrum of compound **7c** S22

Figure S36. ^{1}HNMR of compound **7d** S22

Figure S37. ^{13}CNMR of compound **7d** S37

Figure S38. ^{31}PNMR of compound **7d** S28

Figure S39. MS spectrum of compound **7d** S24

Figure S40. ^{1}HNMR of compound **7e** S24

Figure S41. ^{13}CNMR of compound **7e** S25

Figure S42. ^{31}PNMR of compound **7e** S25

Figure S43. MS spectrum of compound **7e** S26

Figure S44. 1HNMR of compound **9a** S26
Figure S45. ^{13}CNMR of compound **9a** S27
Figure S46. ^{31}PNMR of compound **9a** S27
Figure S47. MS spectrum of compound **9a** S28
Figure S48. ^{1}HNMR of compound **9b** S28
Figure S49. 13CNMR of compound **9b** S29
Figure S50. ^{31}PNMR of compound **9b** S29
Figure S51. MS spectrum of compound **9b** S30
Figure S52. ^{1}HNMR of compound **9c** S30
Figure S53. ^{13}CNMR of compound **9c** S31
Figure S54. ^{31}PNMR of compound **9c** S31
Figure S55. MS spectrum of compound **9c** S32
Figure S56. ^{1}HNMR of compound **9d** S32
Figure S57. ^{13}CNMR of compound **9d** S33
Figure S58. ^{31}PNMR of compound **9d** S33
Figure S59. MS spectrum of compound **9d** S34
Figure S 60. ^{1}HNMR of compound **9e** S34
Figure S61. 13CNMR of compound **9e** S35
Figure S62. ^{31}PNMR of compound **9e** S35
Figure S63. MS spectrum of compound **9e** S36
Figure S64. ^{1}HNMR of compound **9f** S36
Figure S65. ^{13}CNMR of compound **9f** S37
Figure S66. ^{31}PNMR of compound **9f** S37
Figure S67. MS spectrum of compound **9f** S38
Figure S68. ^{1}HNMR of compound **9g** S38
Figure S69. ^{13}CNMR of compound **9g** S39
Figure S70. ^{31}PNMR of compound **9g** S39
Figure S71. MS spectrum of compound **9g** S40
Figure S72. ^{1}HNMR of compound **9h** S40
Figure S73. ^{13}CNMR of compound **9h** S41
Figure S74. ^{31}PNMR of compound **9h** S41
Figure S75. MS spectrum of compound **9h** S42
Figure S76. ^{1}HNMR of compound **10** S42
Figure S77. ^{13}CNMR of compound **10** S43
Figure S78. ^{31}PNMR of compound **10** S43
Figure S79. MS spectrum of compound **10** S44
Figure S80. ^{1}HNMR of compound **12a** S44
Figure S81. ^{13}CNMR of compound **12a** S45
Figure S82. ^{31}PNMR of compound **12a** S45
Figure S83. MS spectrum of compound **12a** S46
Figure S84. ^{1}HNMR of compound **12b** S46
Figure S85. ^{13}CNMR of compound **12b** S47
Figure S86. ^{31}PNMR of compound **12b** S47
Figure S87. MS spectrum of compound **12b** S48
Figure S88. ^{1}HNMR of compound **12c** S48

Figure S89. ^{13}CNMR of compound **12c** S49
Figure S90. ^{31}PNMR of compound **12c** S49
Figure S91. MS spectrum of compound **12c** S50
Figure S92. ^{1}HNMR of compound **12d** S50
Figure S93.^{13}CNMR of compound **12d** S51
Figure S94. ^{31}PNMR of compound **12d** S51
Figure S95. MS spectrum of compound **12d** S52
Figure S96. ^{1}HNMR of compound **12e** S52
Figure S97. ^{13}CNMR of compound **12e** S53
Figure S98. ^{31}PNMR of compound **12e** S53
Figure S99. ^{13}CNMR of compound **12f** S54
Figure S100. ^{31}PNMR of compound **12f** S54
Figure S101. MS spectrum of compound **12f** S55

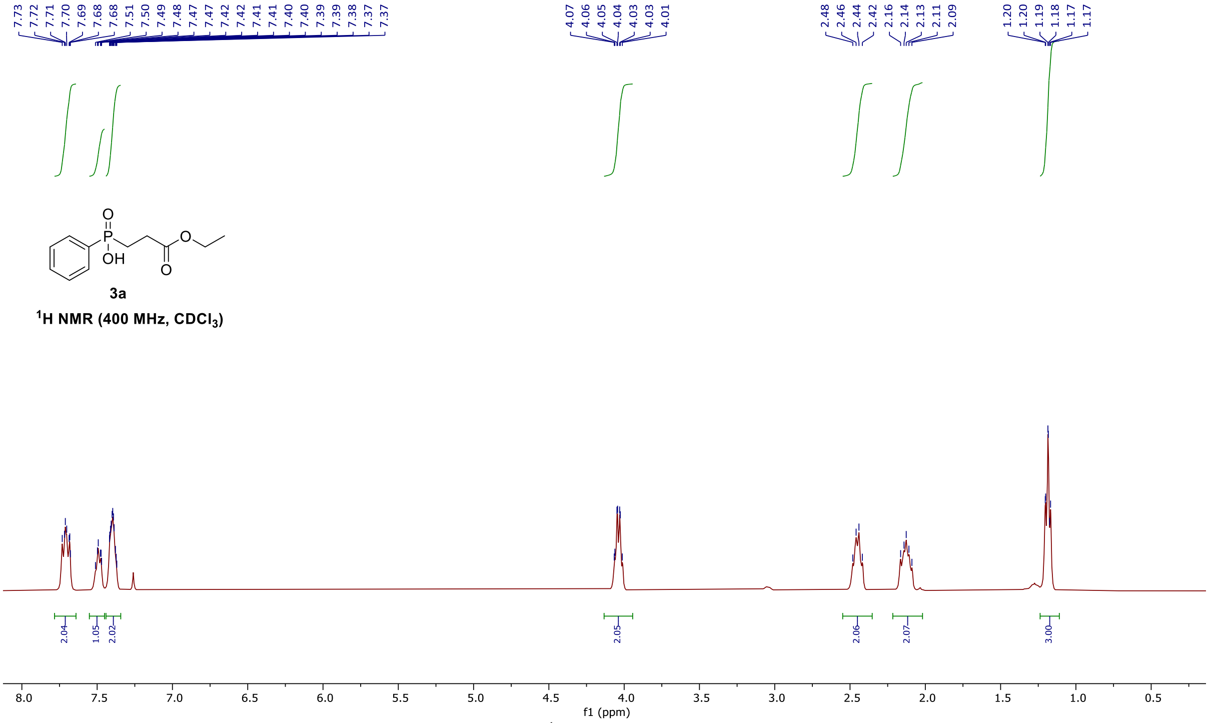

Figure S1. [1]*HNMR of compound* ***3a***

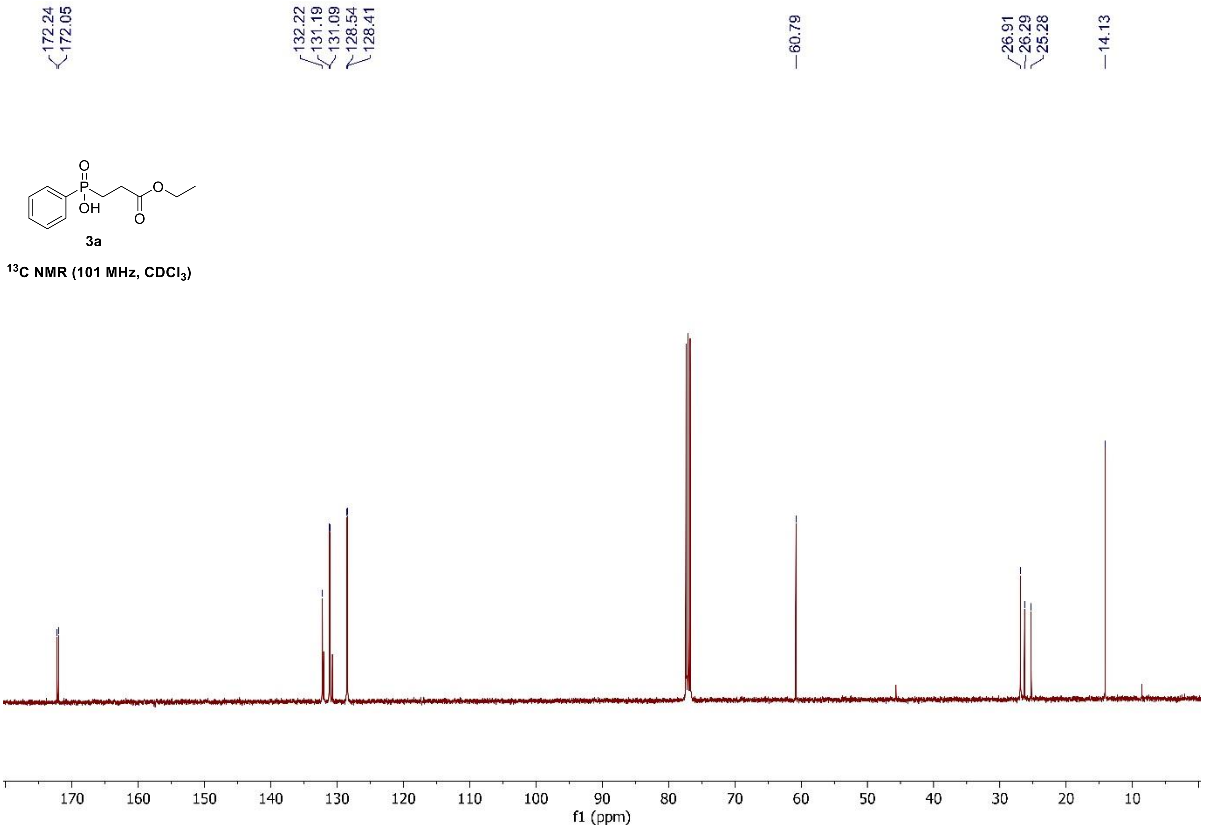

Figure S2. [13]*CNMR of compound* ***3a***

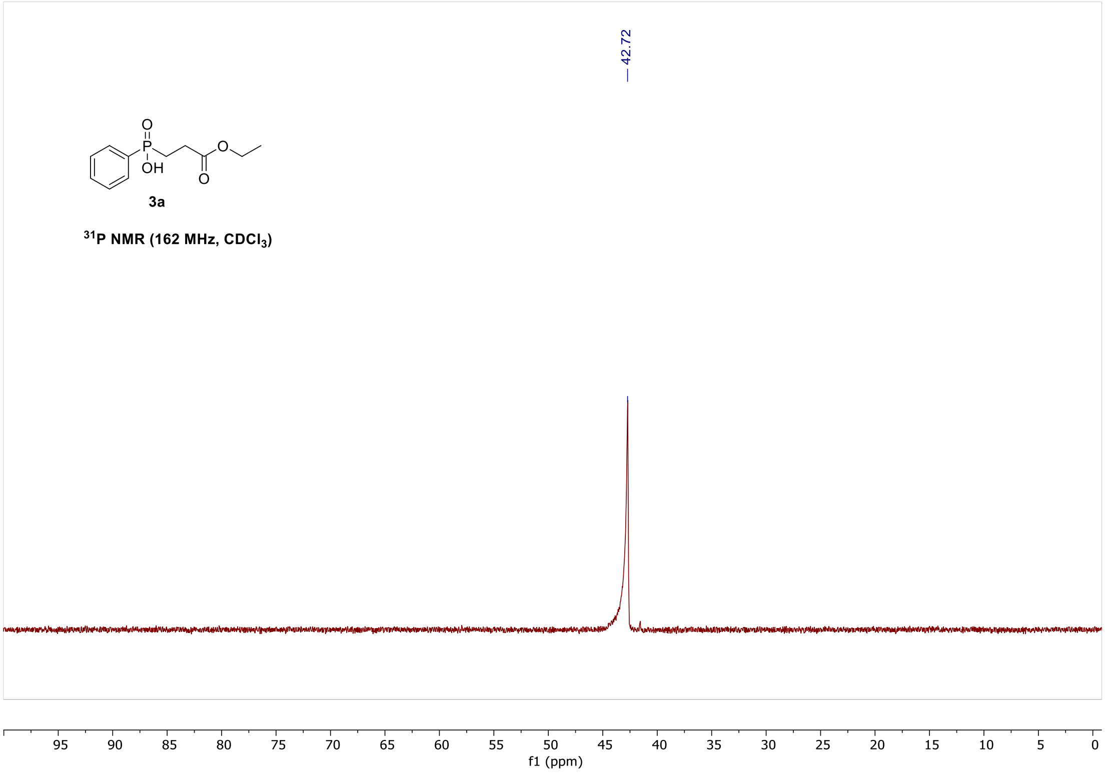

Figure S3. ^{31}PNMR of compound ***3a***

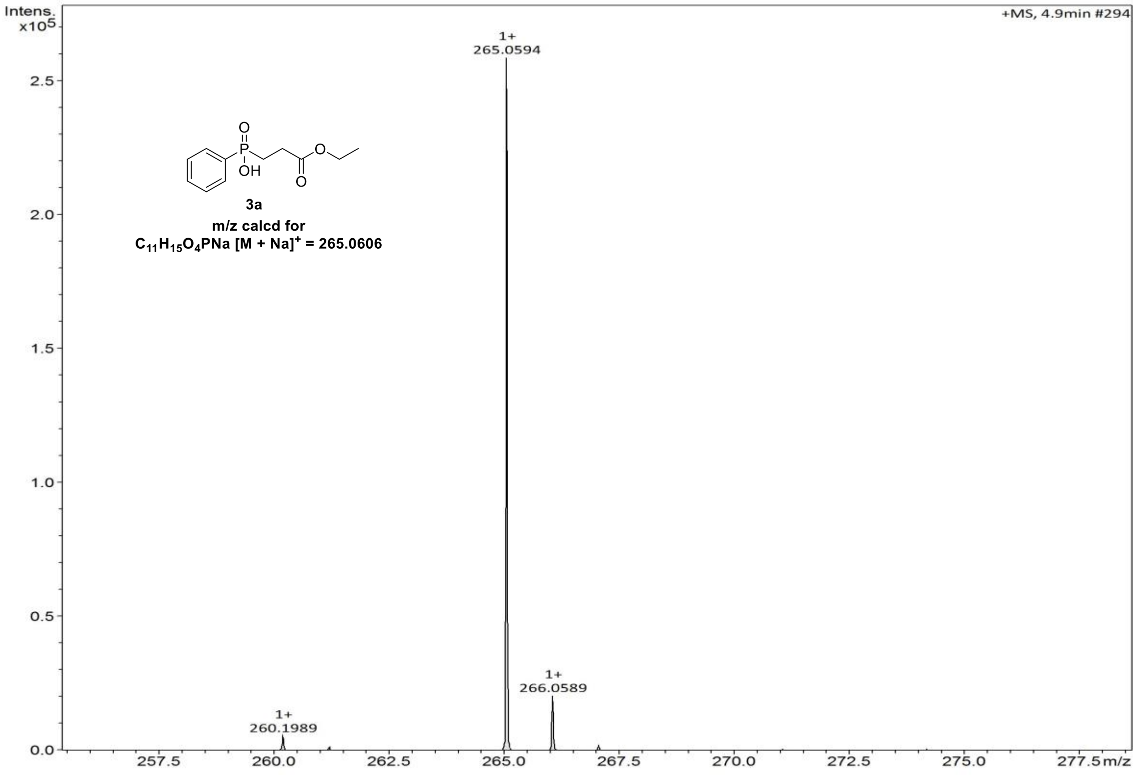

Figure S4. MS spectrum of compound ***3a***

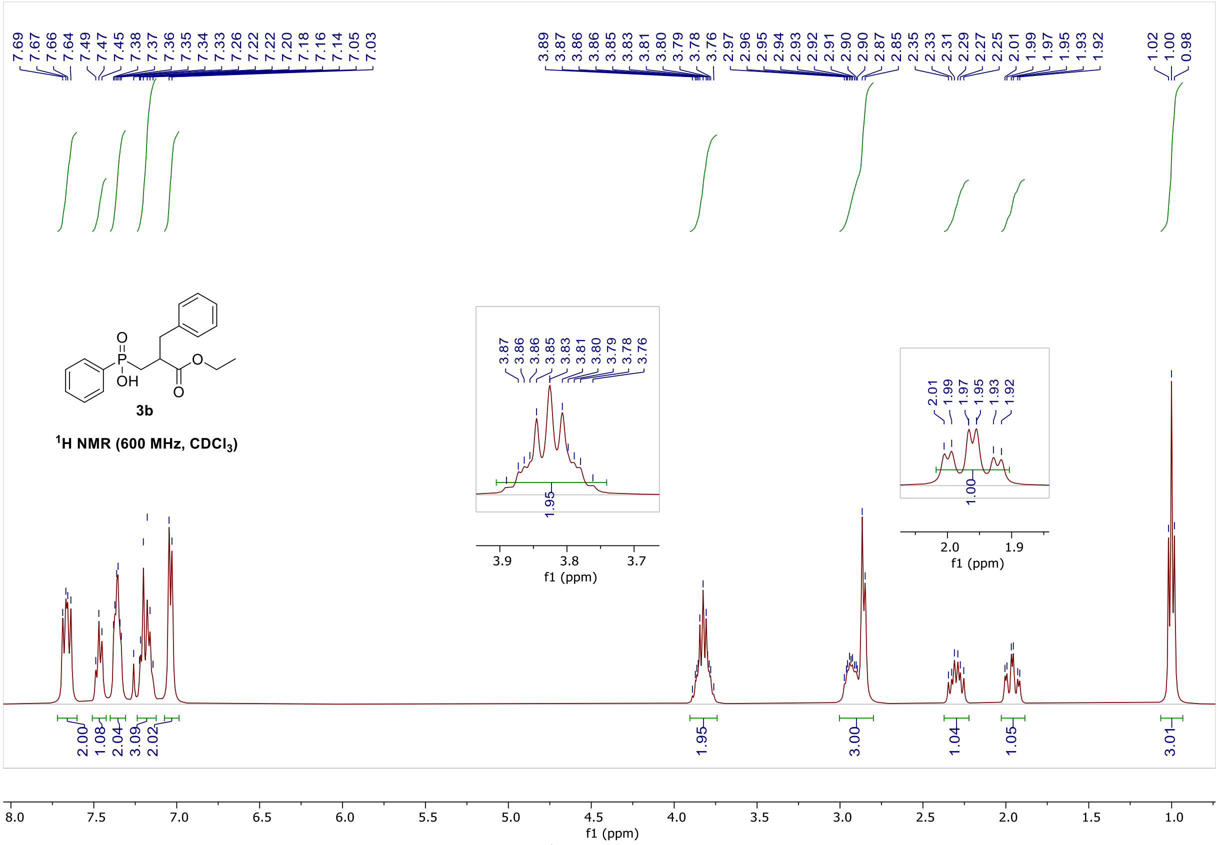

Figure S5. ^{1}HNMR of compound ***3b***

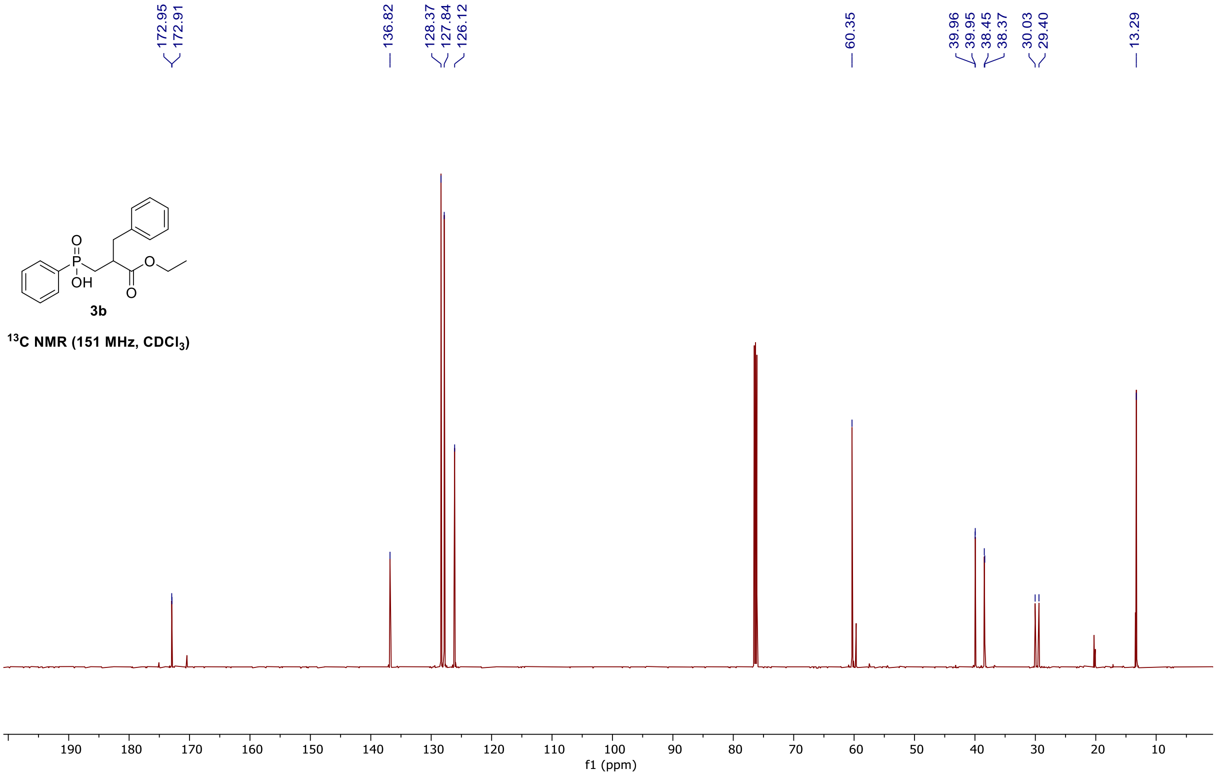

Figure S6. ^{13}CNMR of compound ***3b***

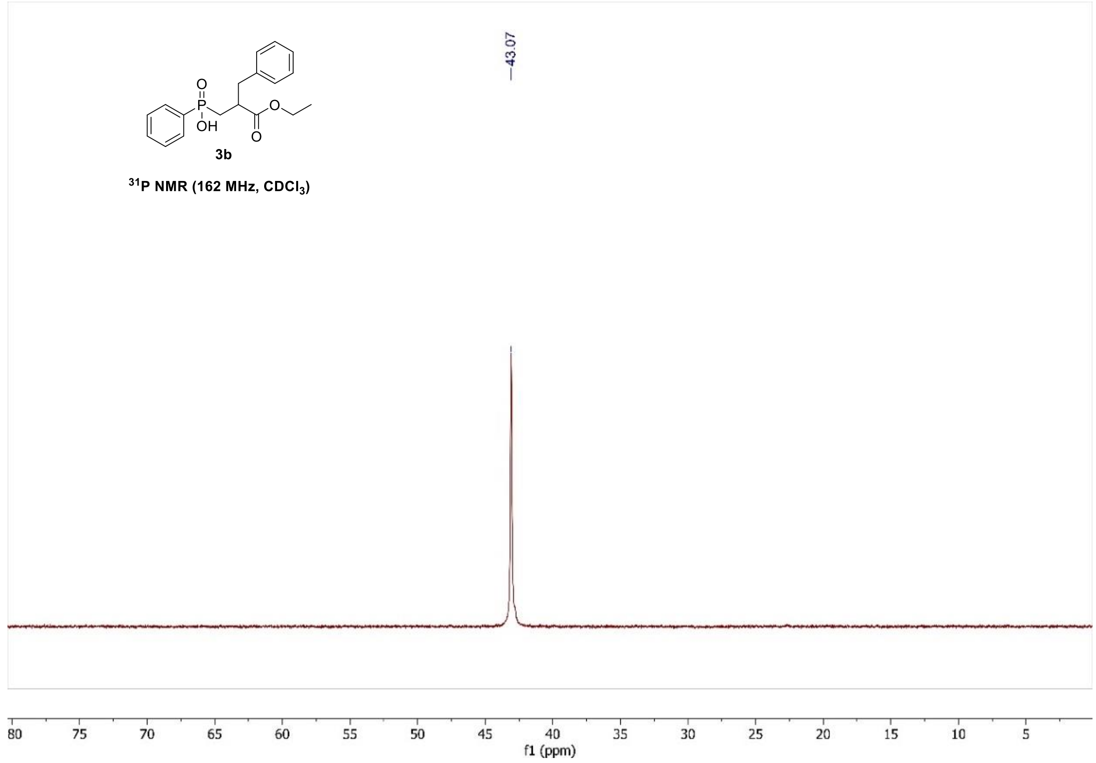

Figure S7. ^{31}PNMR of compound ***3b***

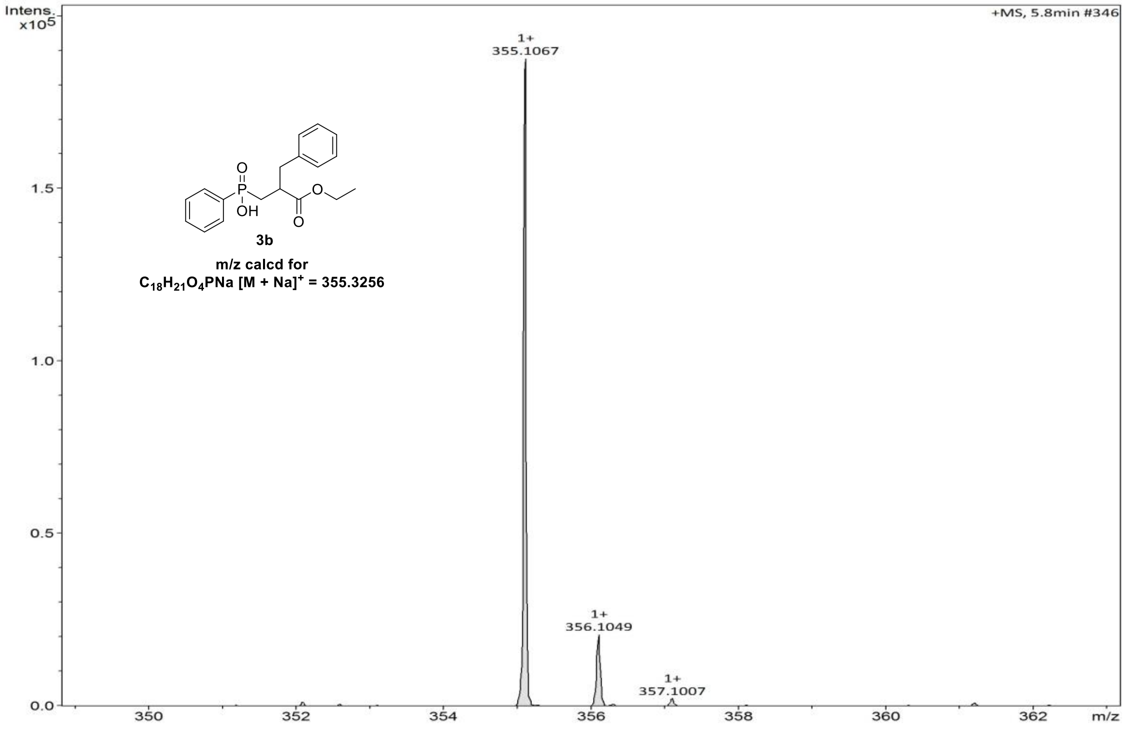

Figure S8. MS spectrum of compound ***3b***

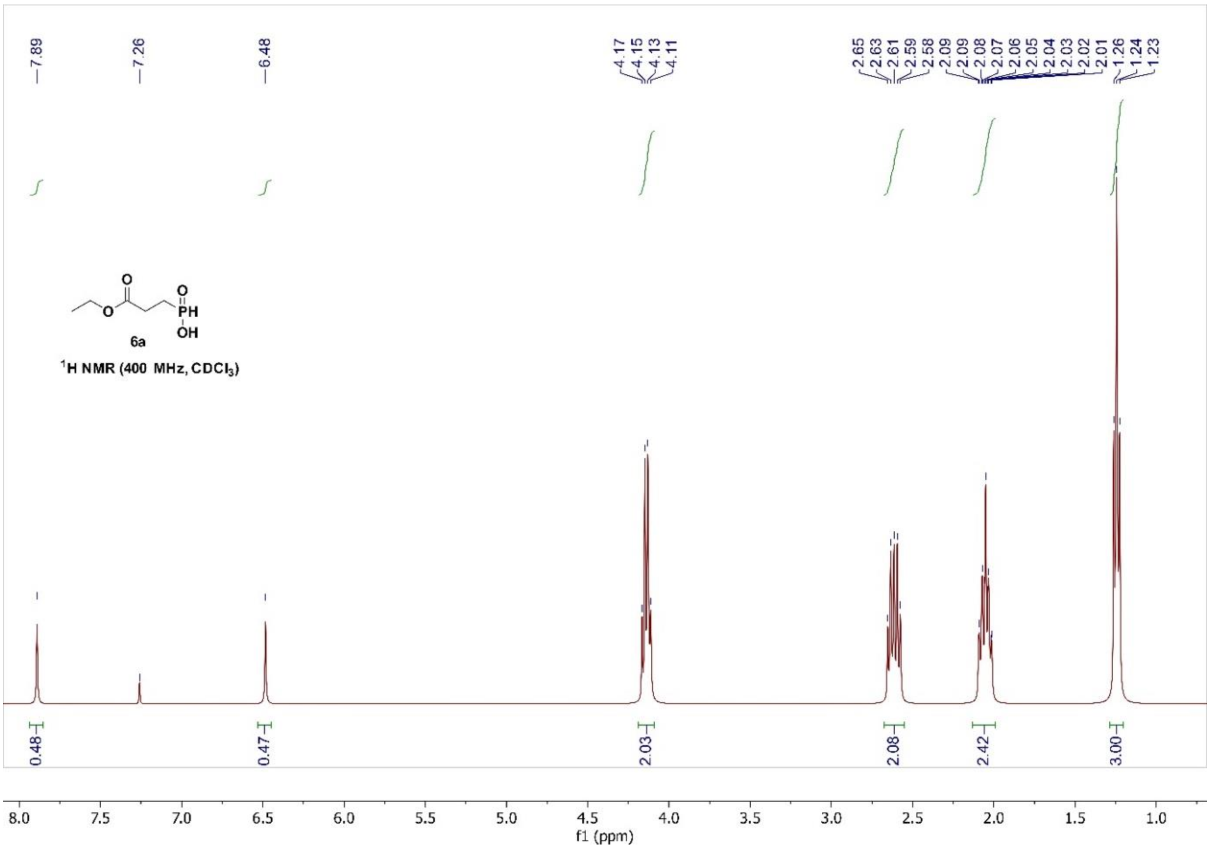

Figure S9. ^{1}HNMR of compound ***6a***

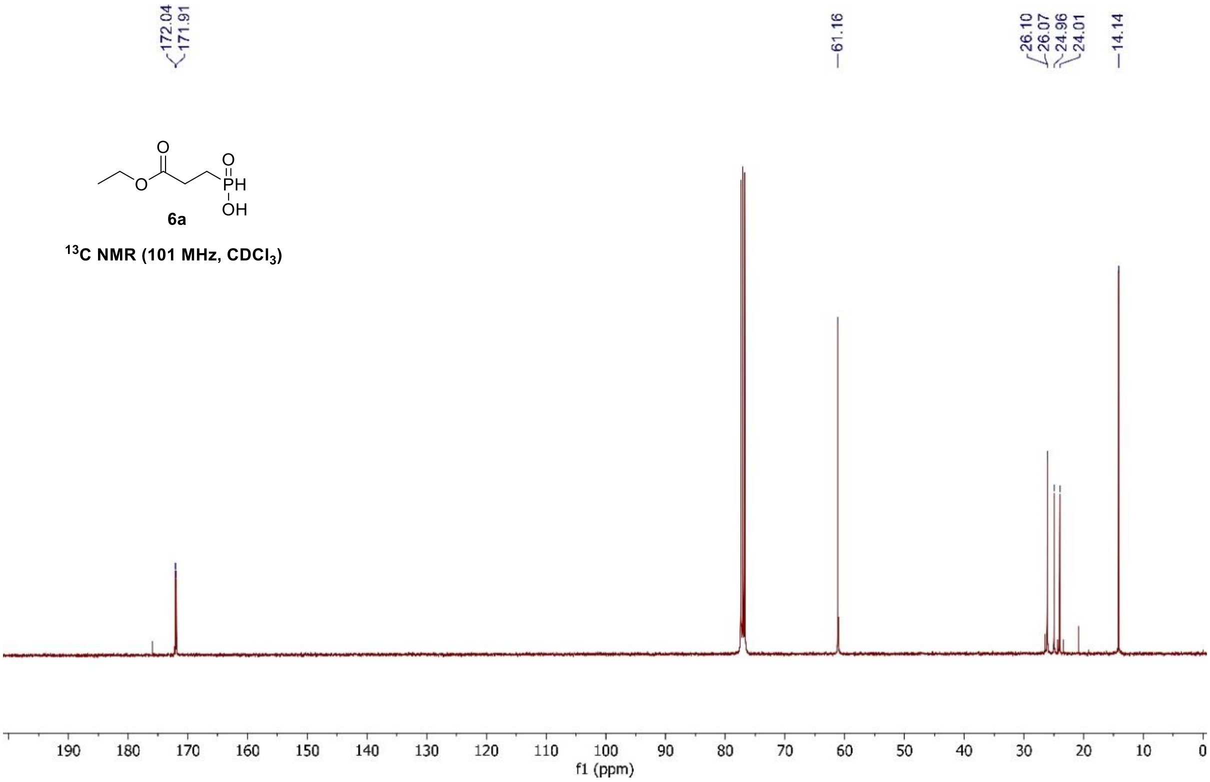

Figure S10. ^{13}CNMR of compound ***6a***

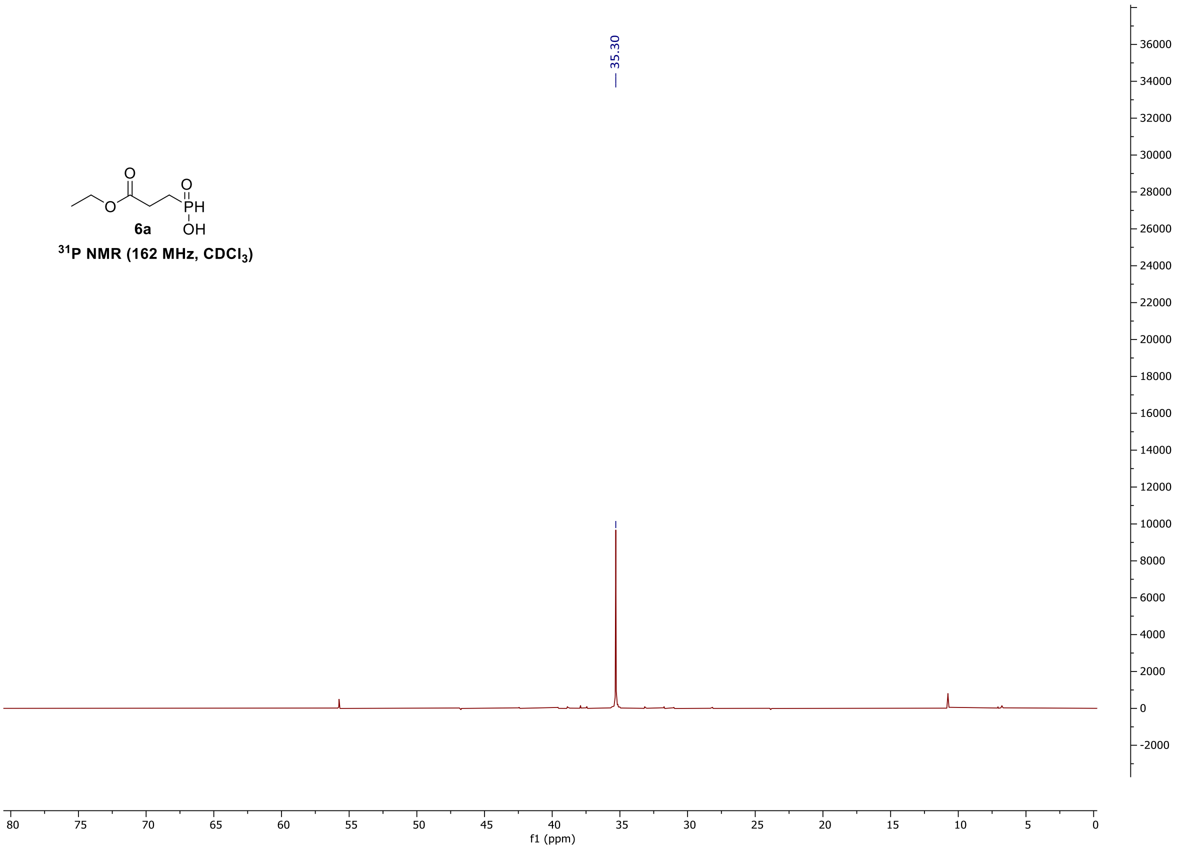

*Figure S11. ^{31}PNMR of compound **6a***

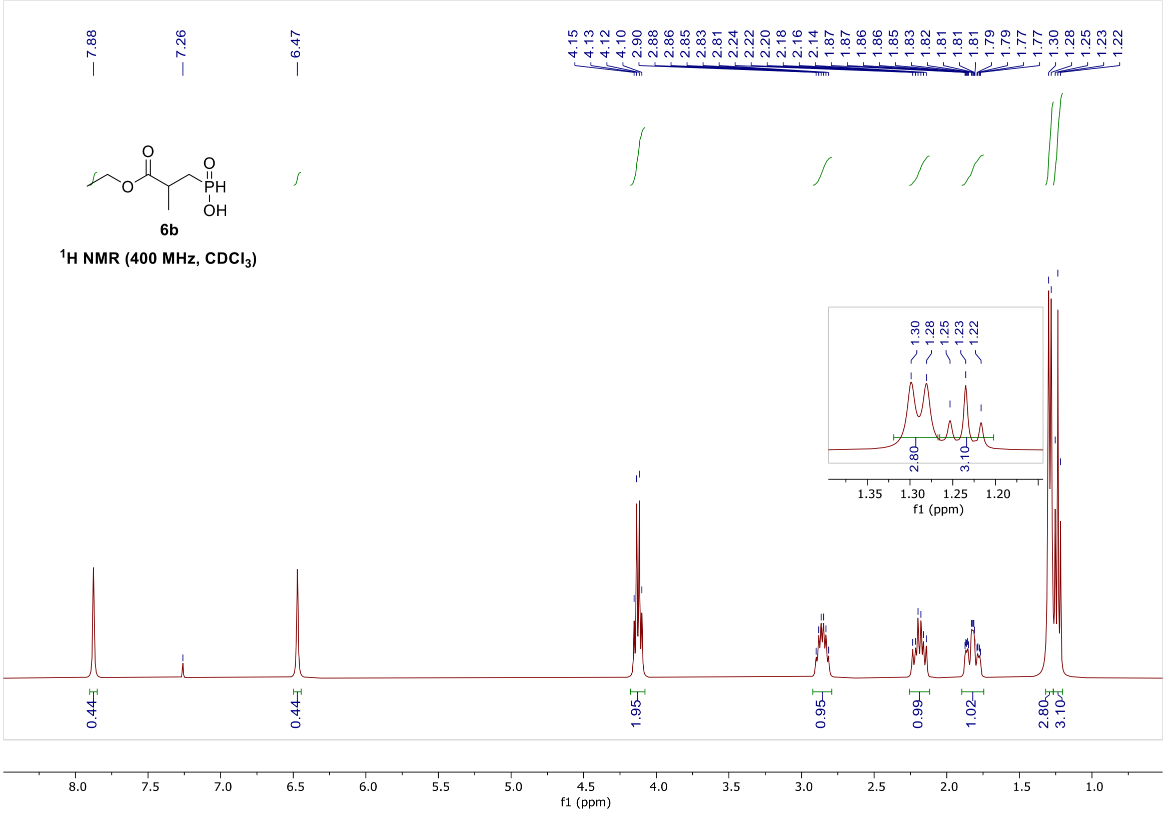

*Figure S12. ^{1}HNMR of compound **6b***

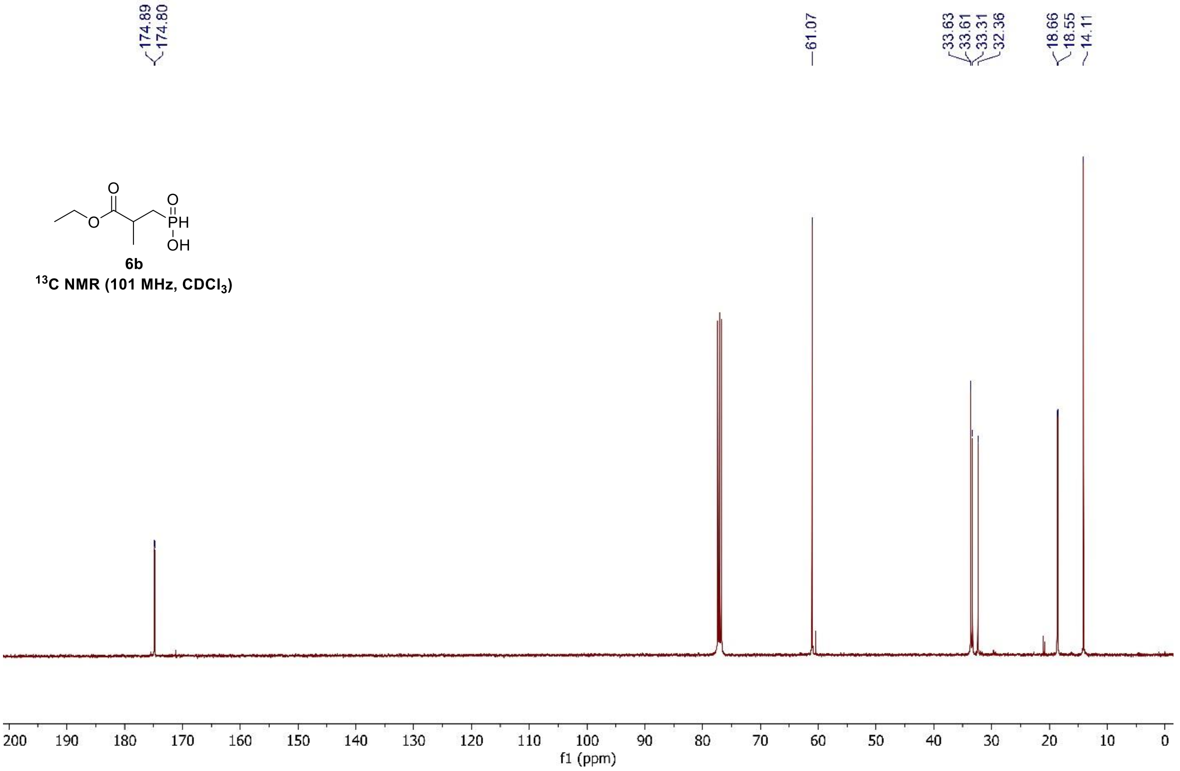

*Figure S13. ^{13}CNMR of compound **6b***

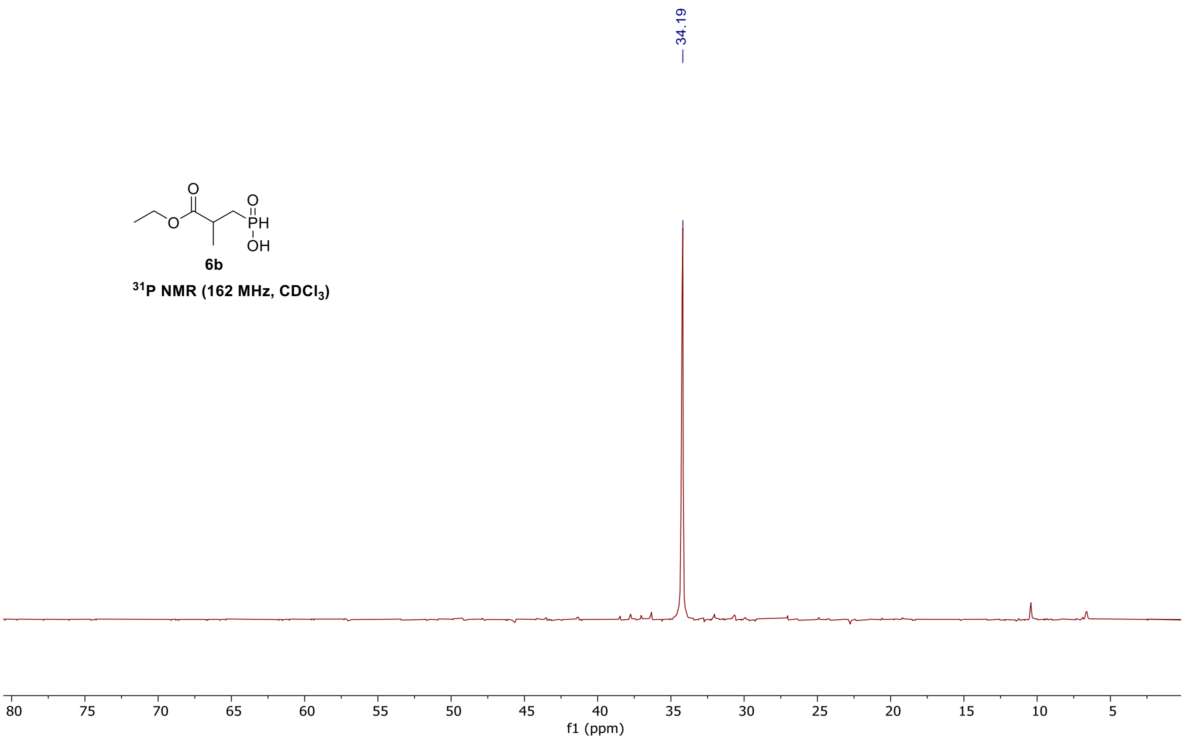

*Figure S14. ^{31}PNMR of compound **6b***

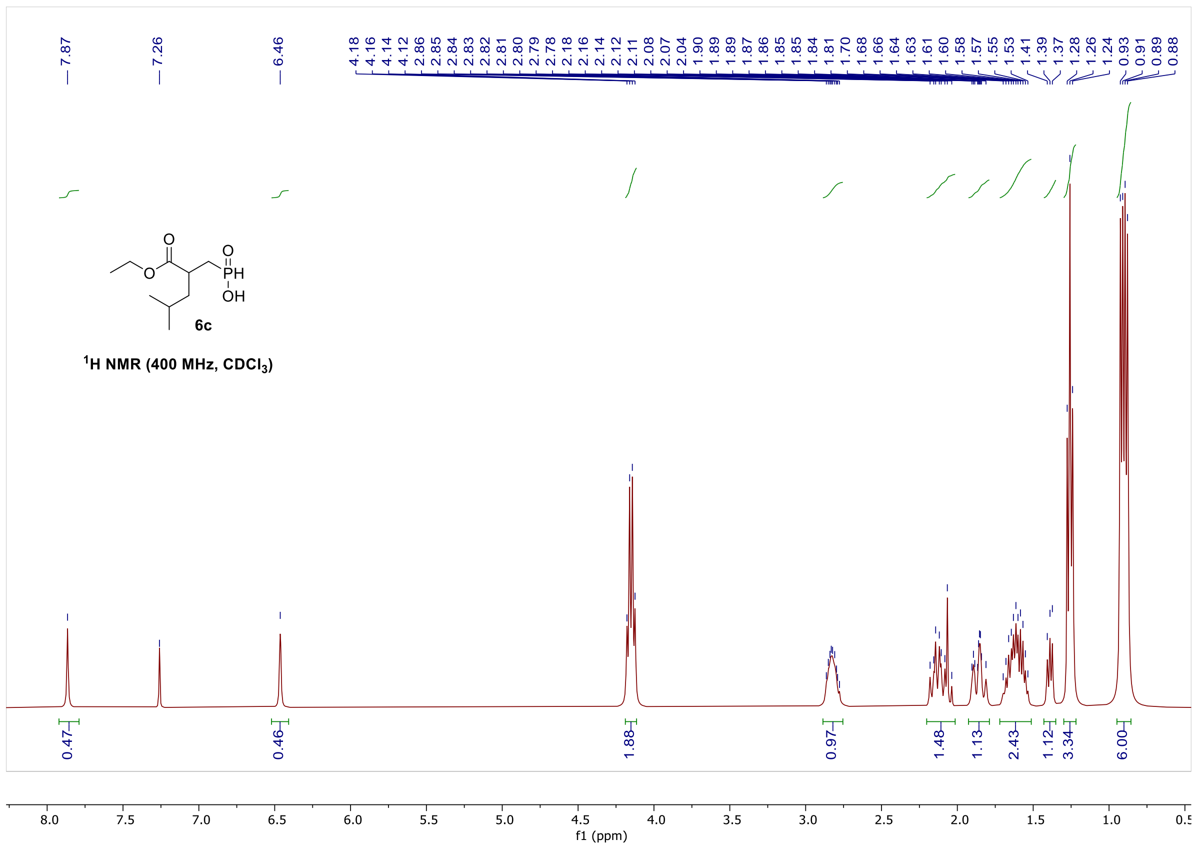

Figure S15. ^{1}HNMR of compound **6c**

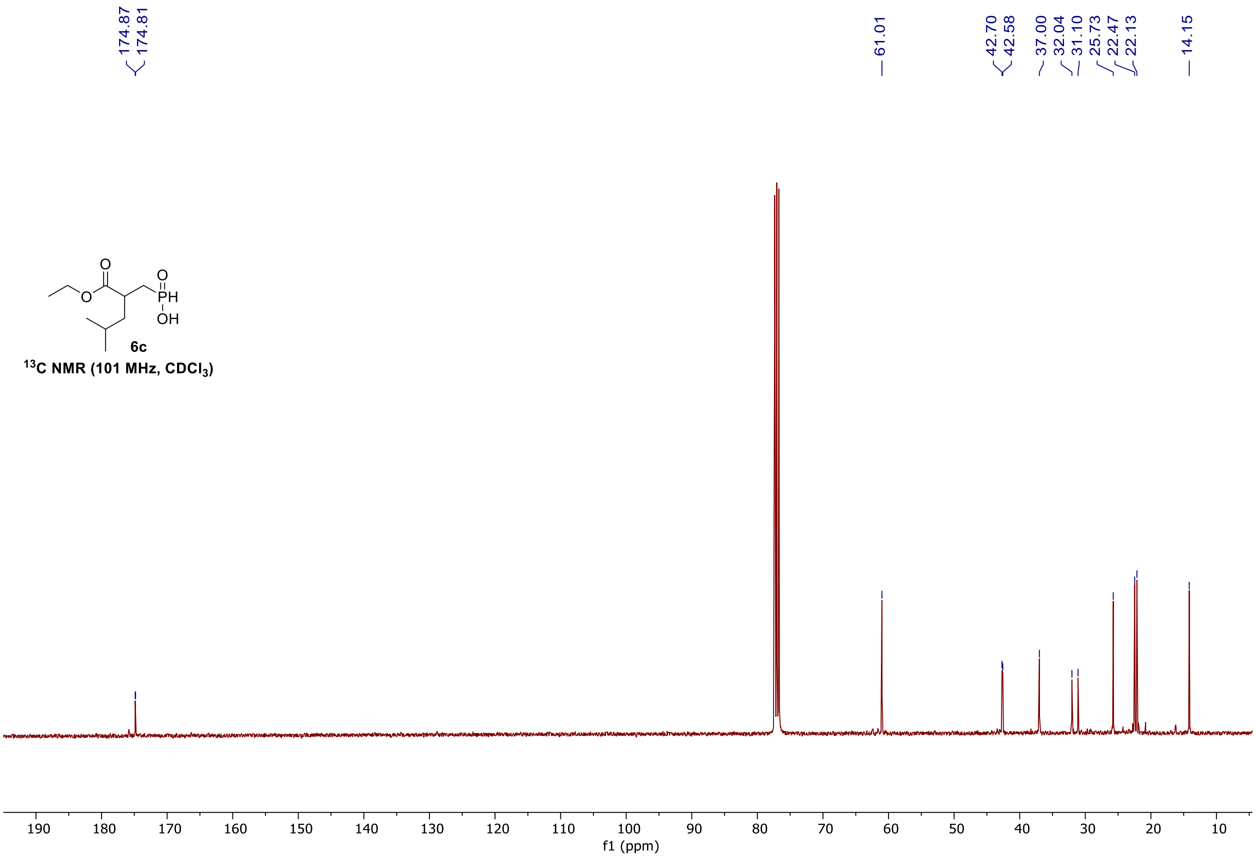

Figure S16. ^{13}CNMR of compound **6c**

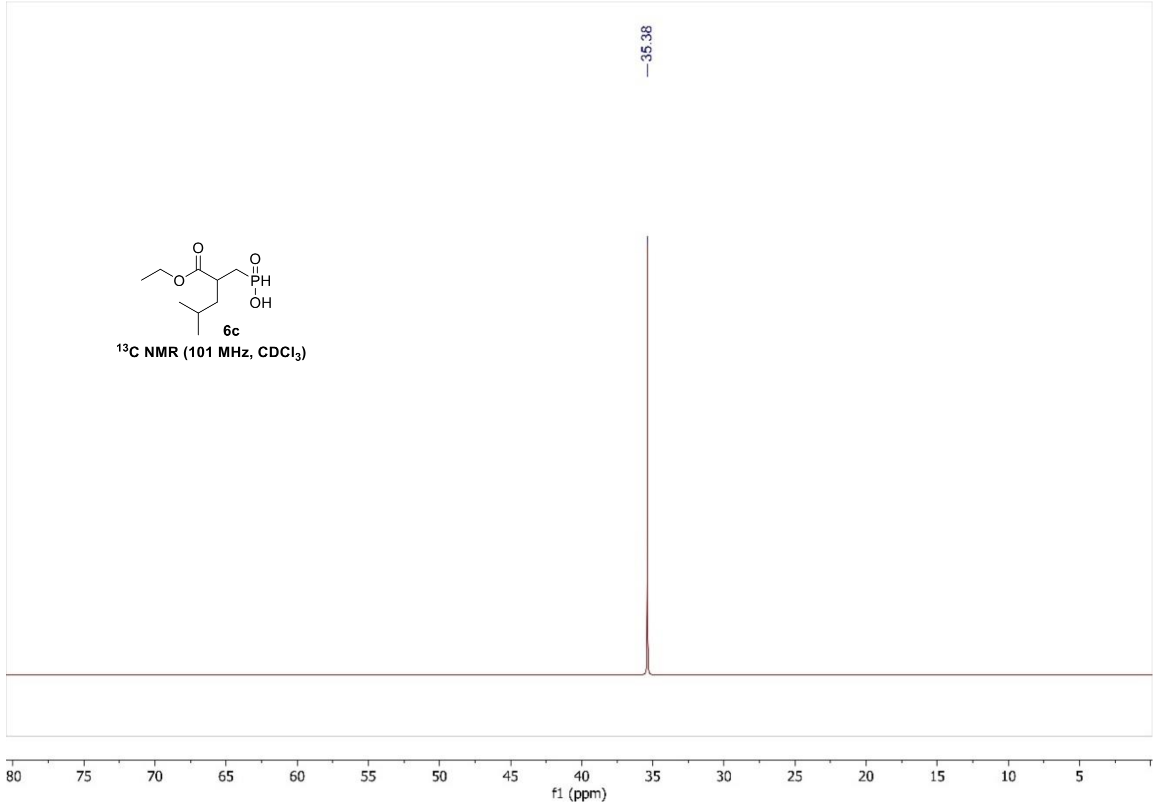

Figure S17. ^{31}PNMR of compound ***6c***

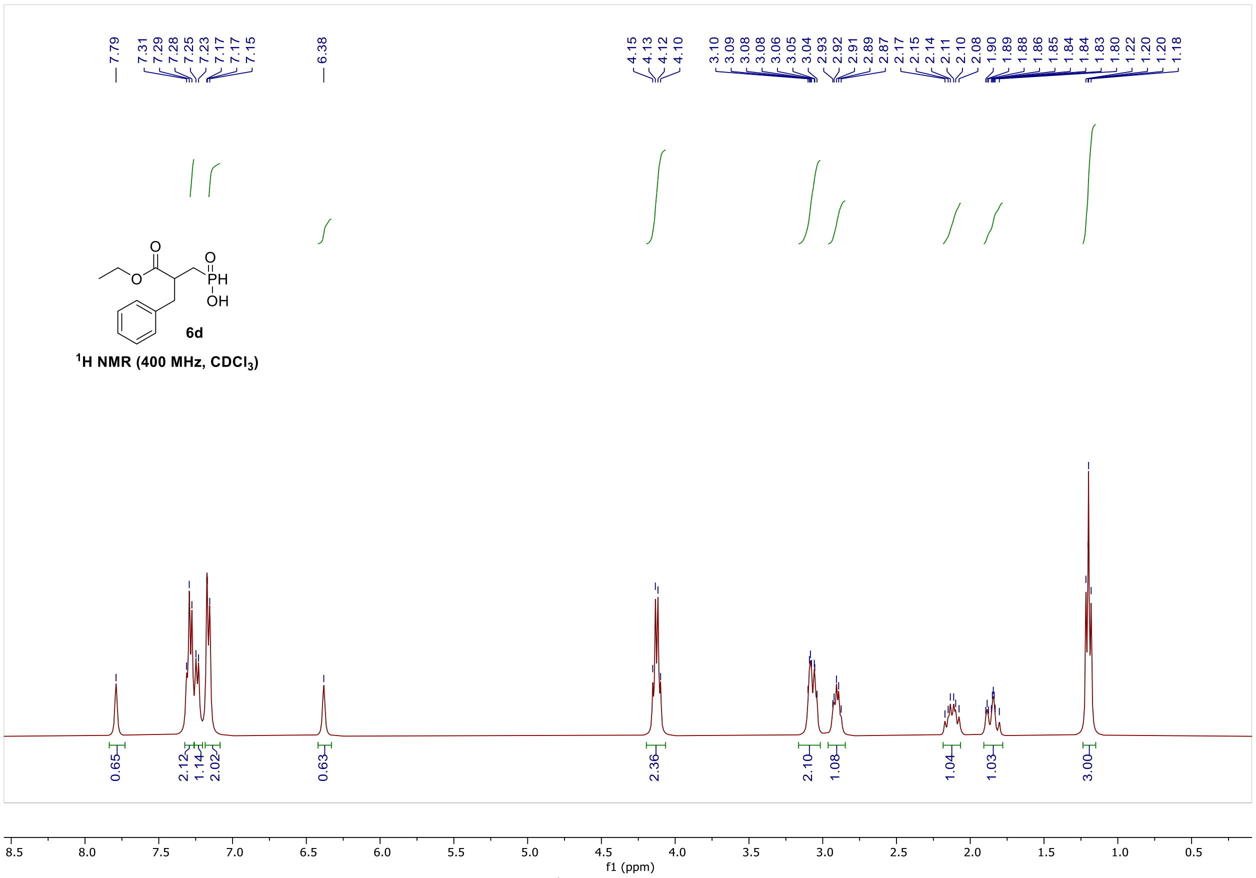

Figure S18. ^{1}HNMR of compound ***6d***

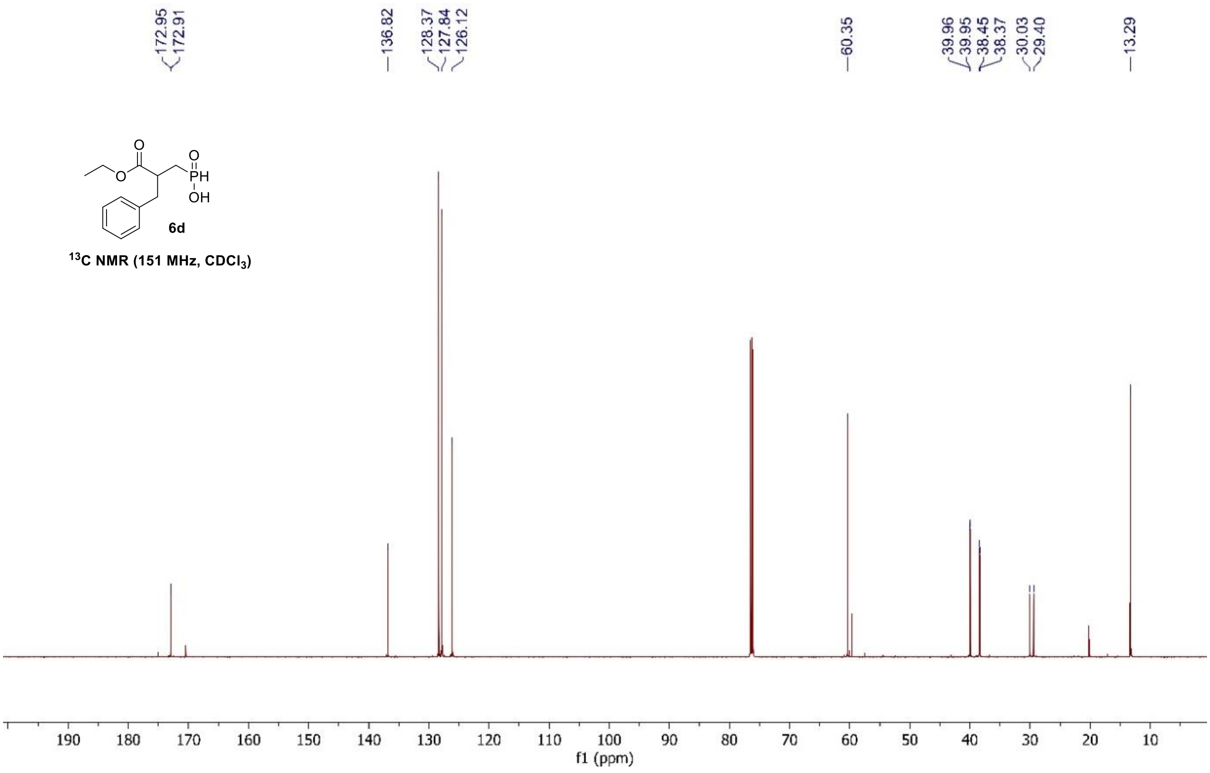

Figure S19. ^{13}CNMR of compound ***6d***

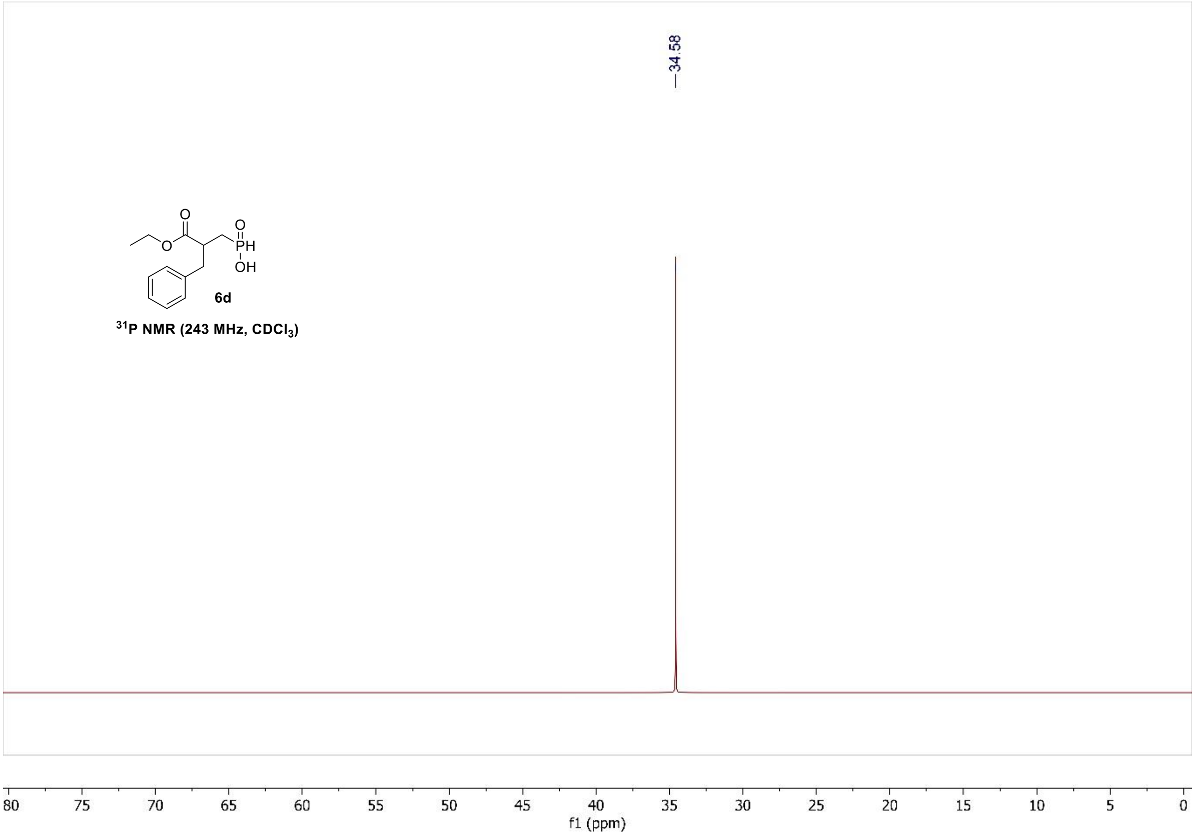

Figure S20. ^{31}PNMR of compound ***6d***

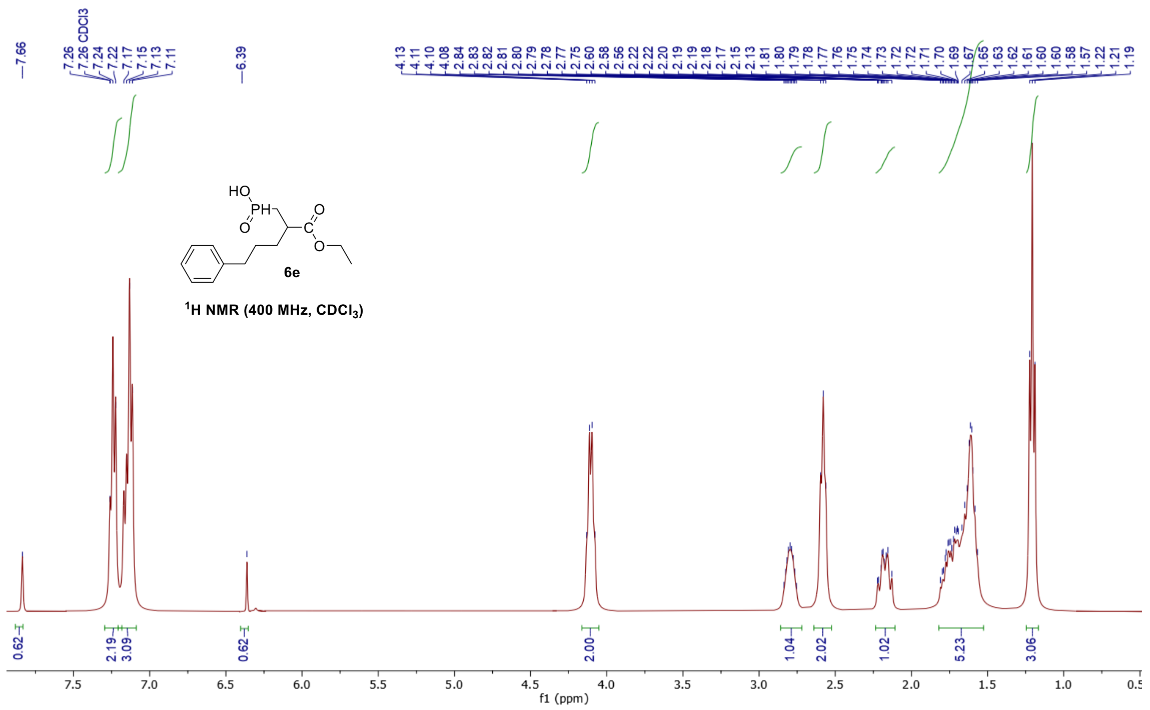

Figure S21. ^{1}HNMR of compound ***6e***

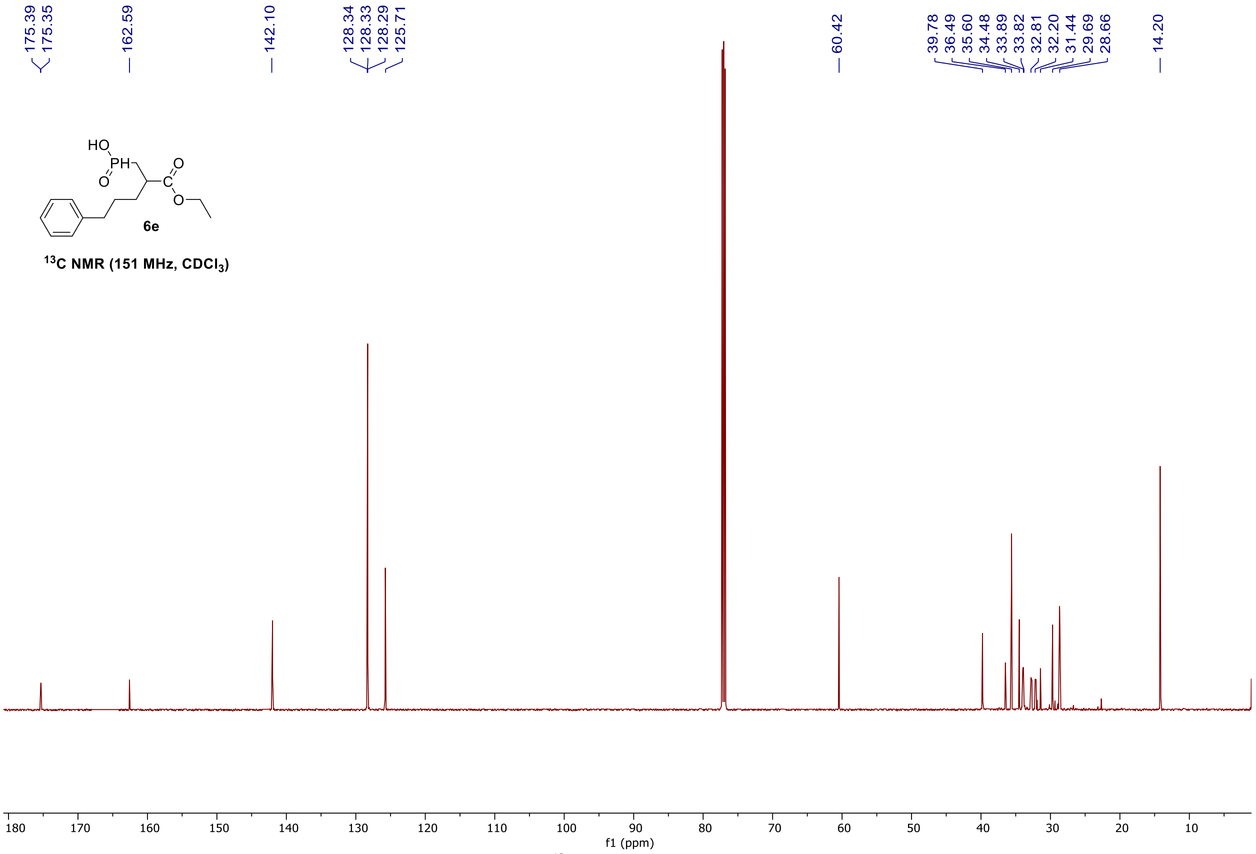

Figure S22. ^{13}CNMR of compound ***6e***

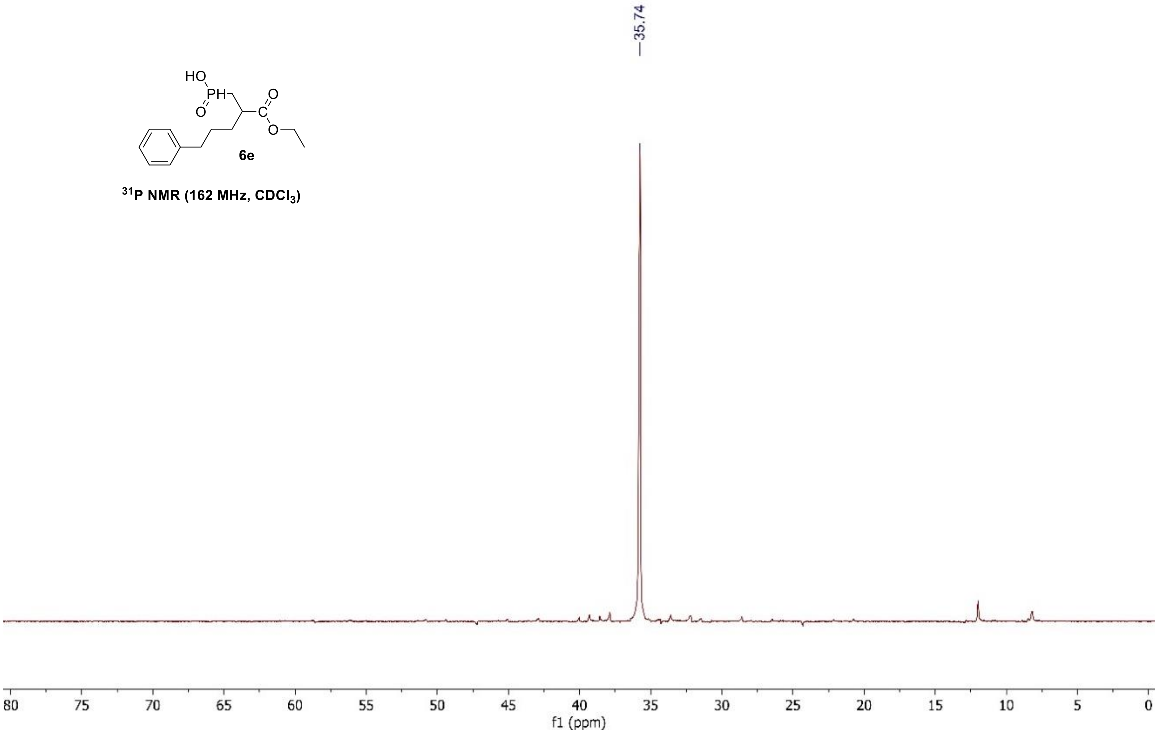

*Figure S23. ^{31}PNMR of compound **6e***

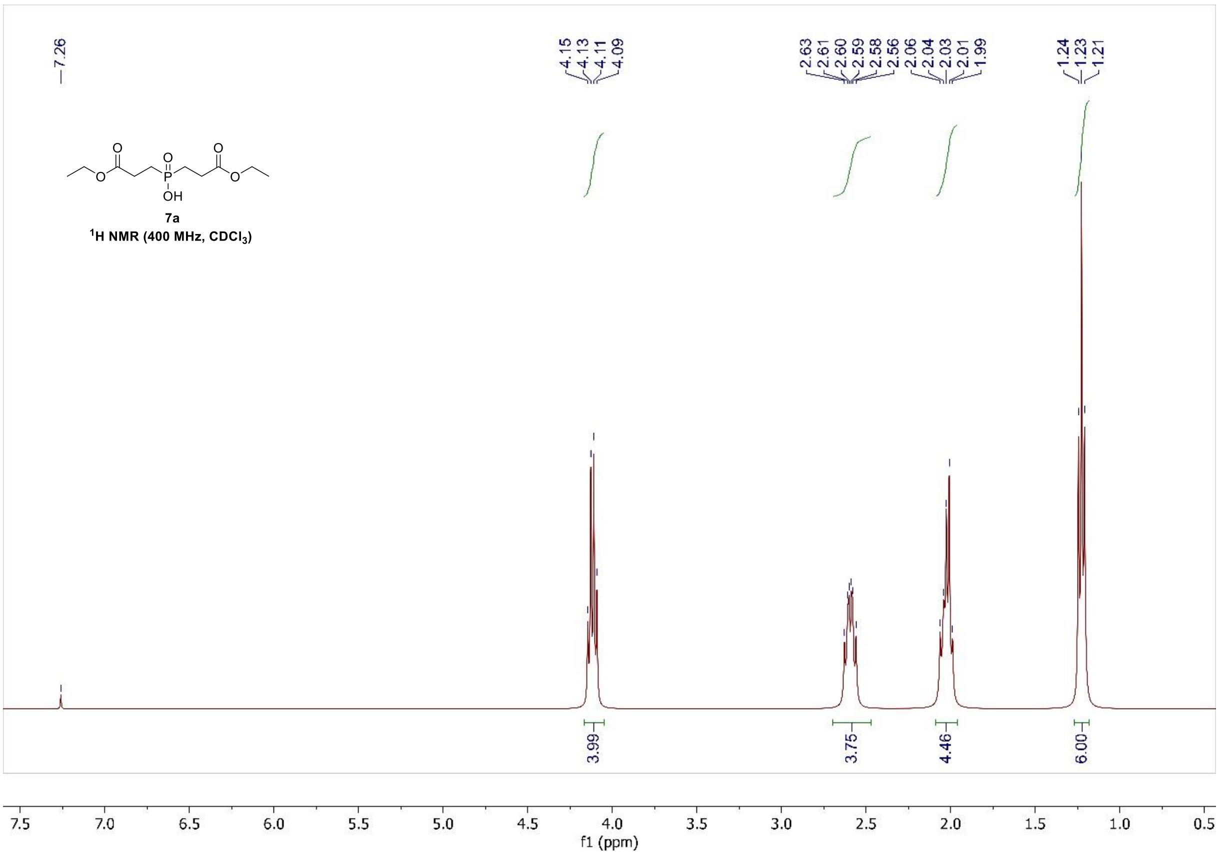

*Figure S24. ^{1}HNMR of compound **7a***

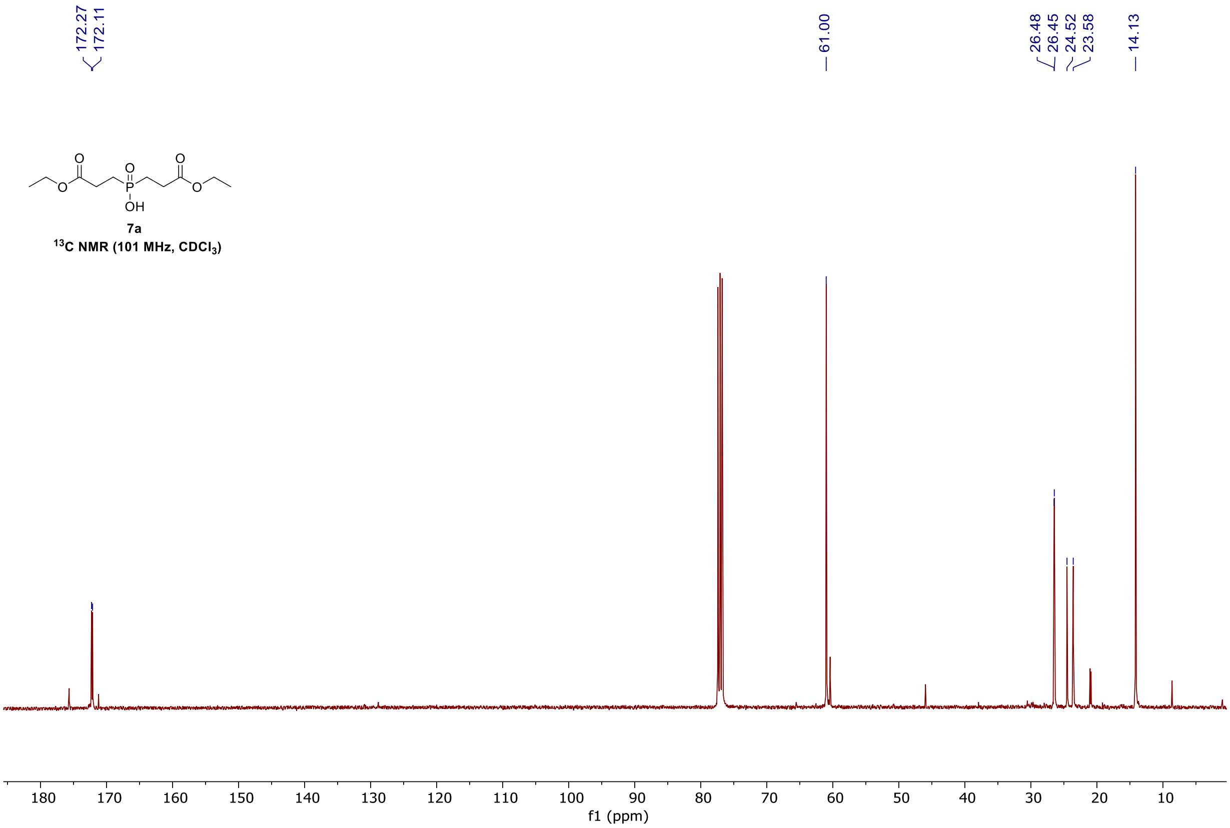

*Figure S25. ^{13}CNMR of compound **7a***

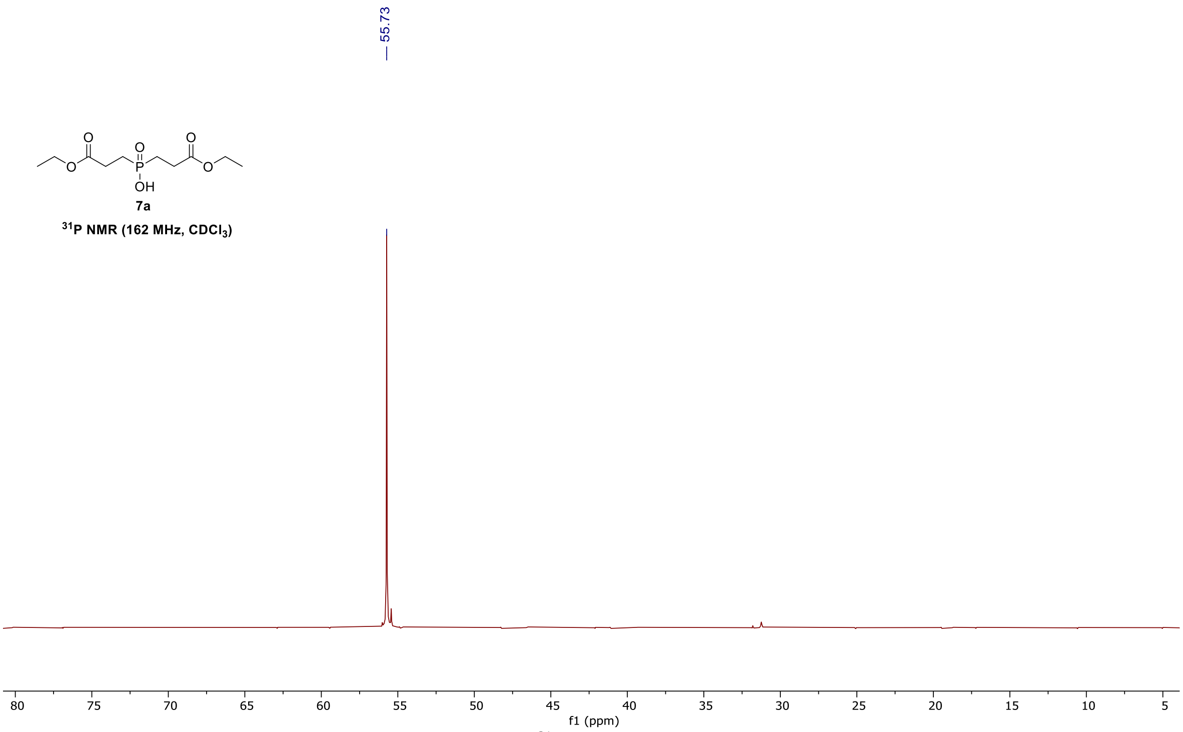

*Figure S26. ^{31}PNMR of compound **7a***

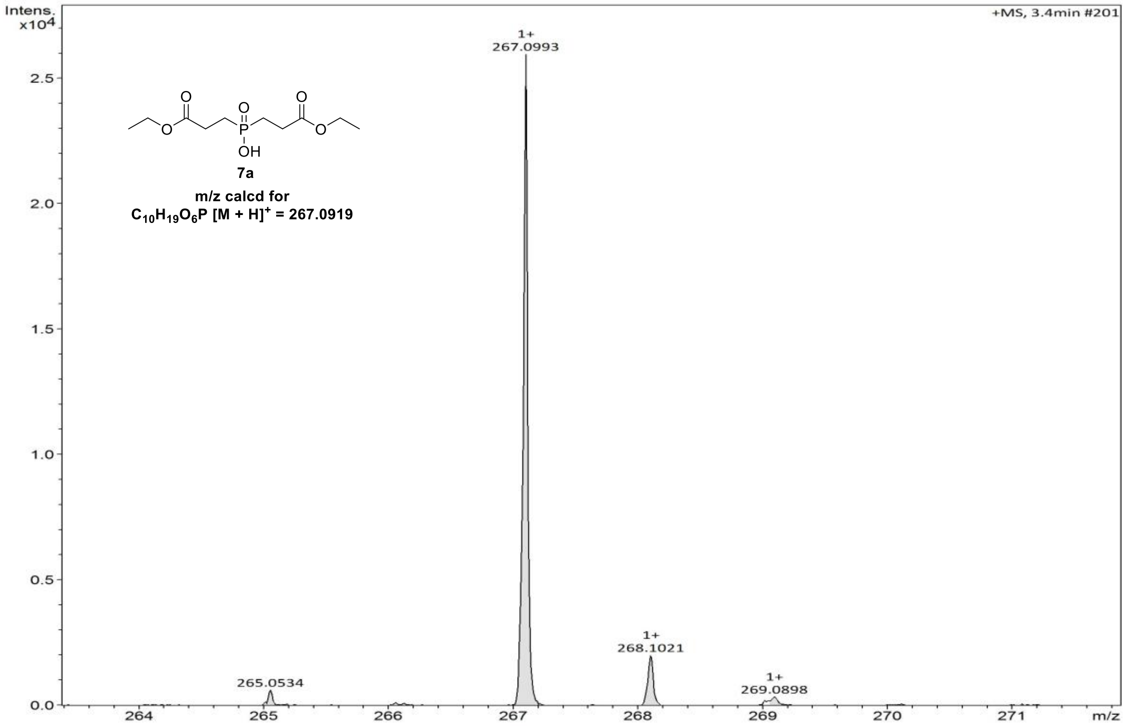

*Figure S27. MS spectrum of compound **7a***

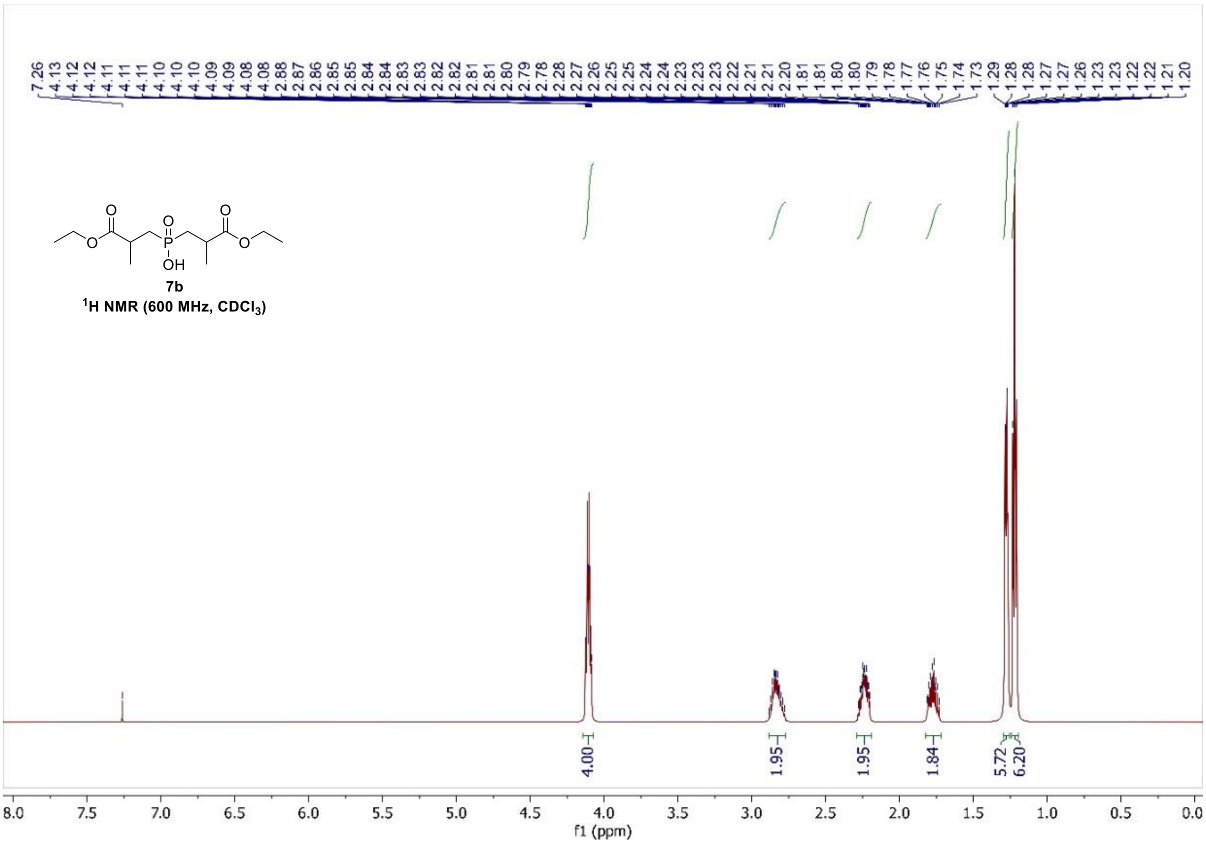

*Figure S28. ^{1}HNMR of compound **7b***

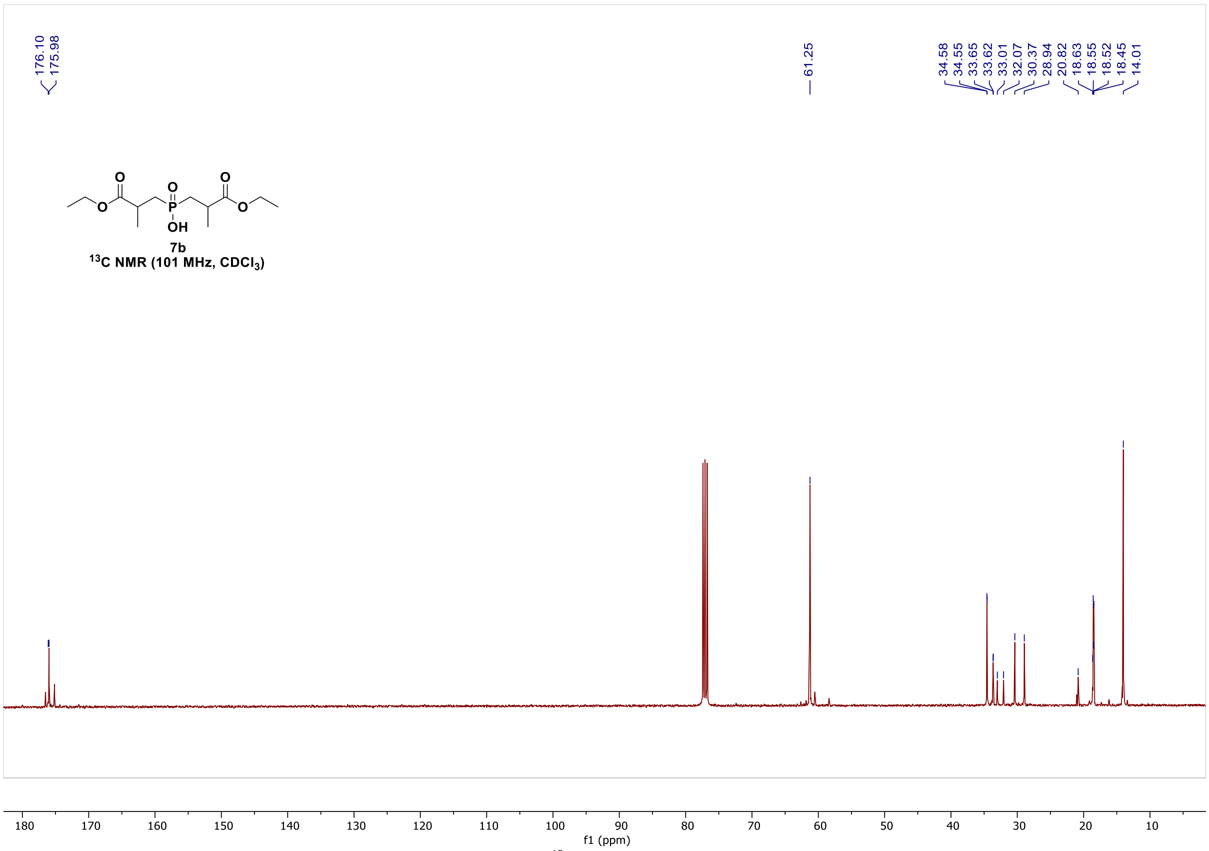

*Figure S29. ^{13}CNMR of compound **7b***

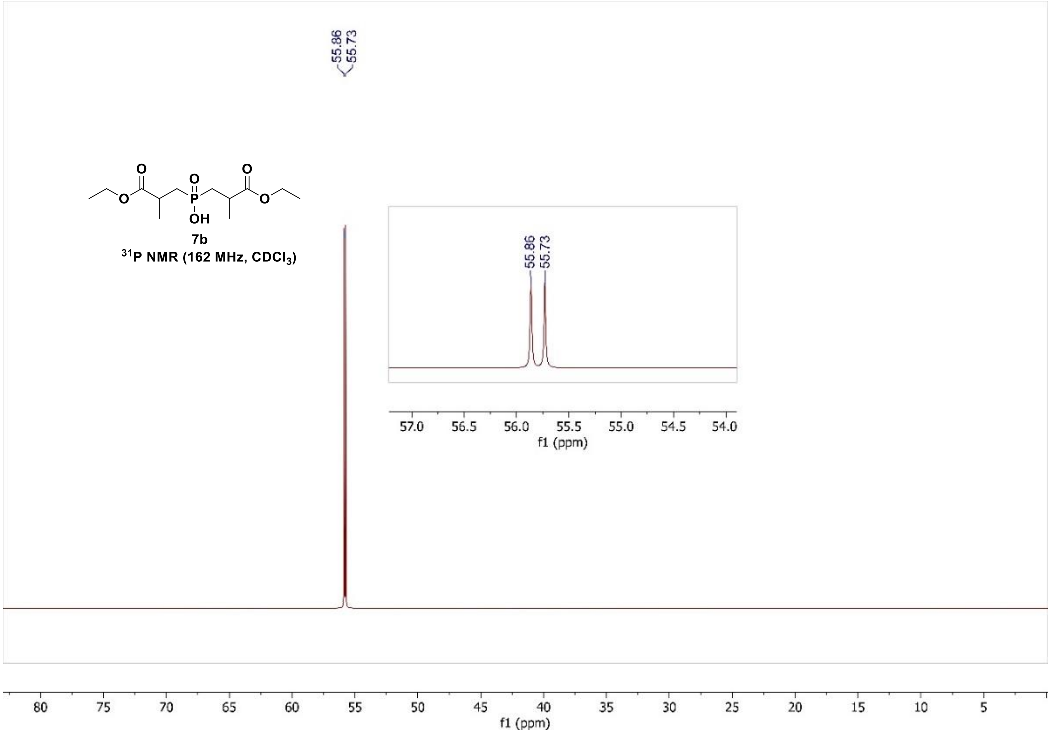

*Figure S30. ^{31}PNMR of compound **7b***

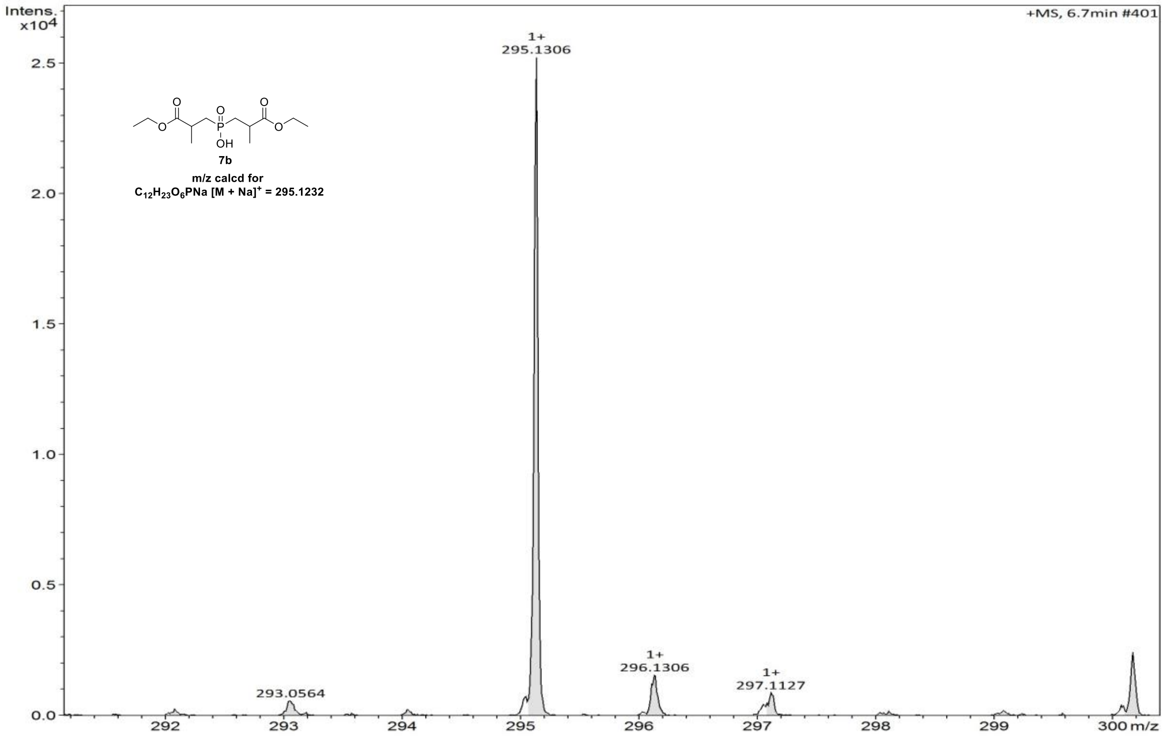

*Figure S31. MS spectrum of compound **7b***

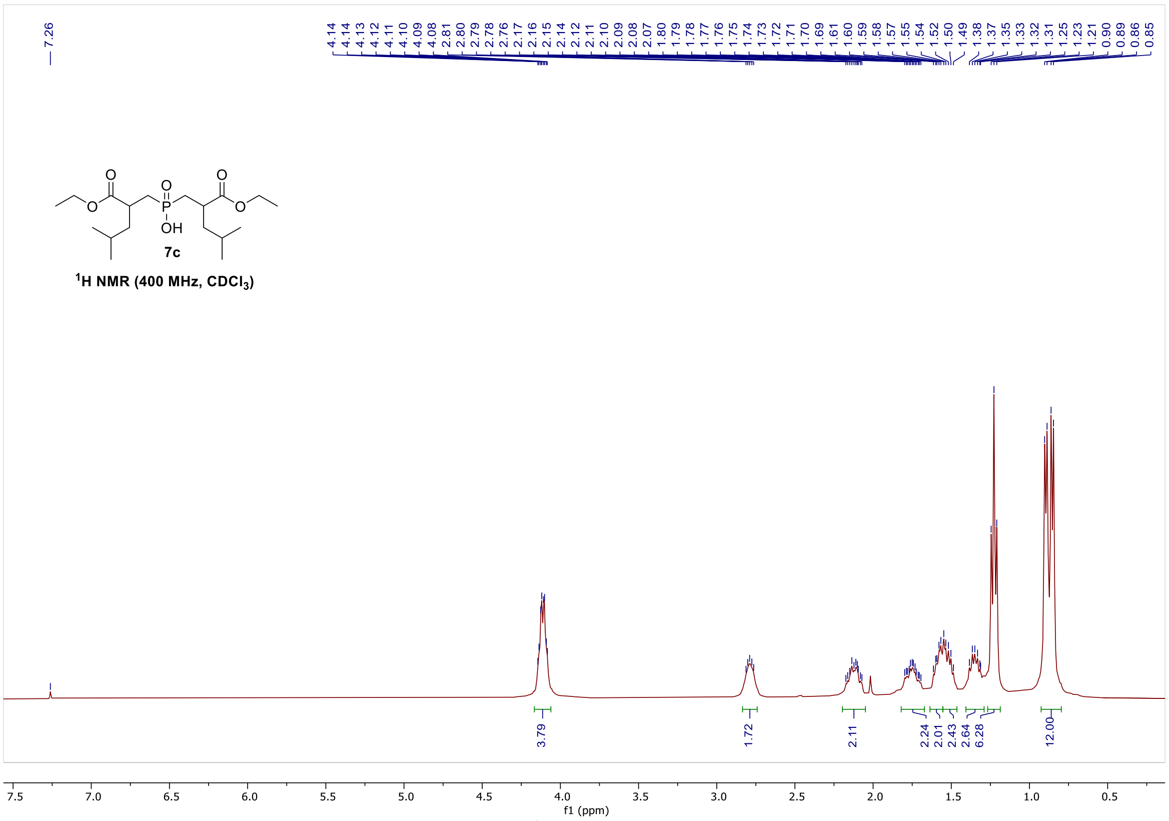

*Figure S32. ^{1}HNMR of compound **7c***

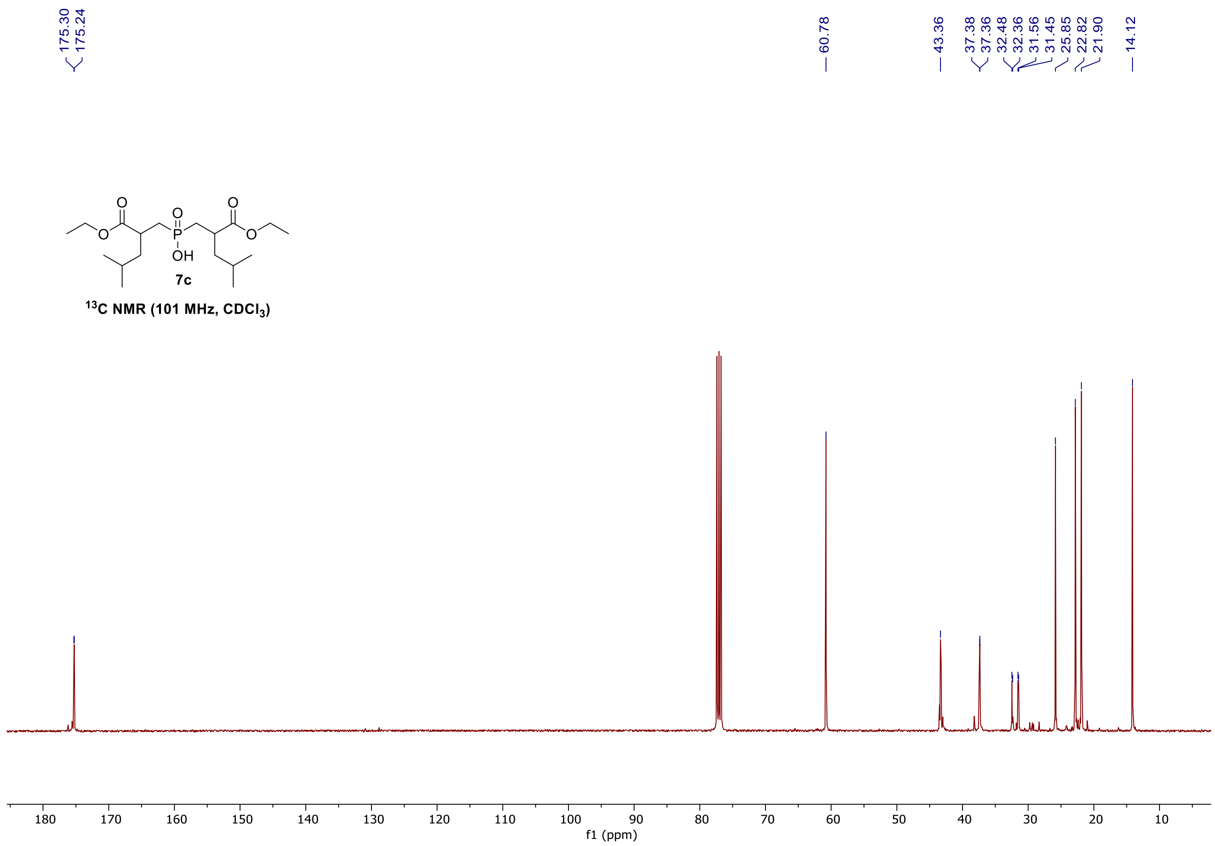

Figure S33.^{13}*CNMR of compound* ***7c***

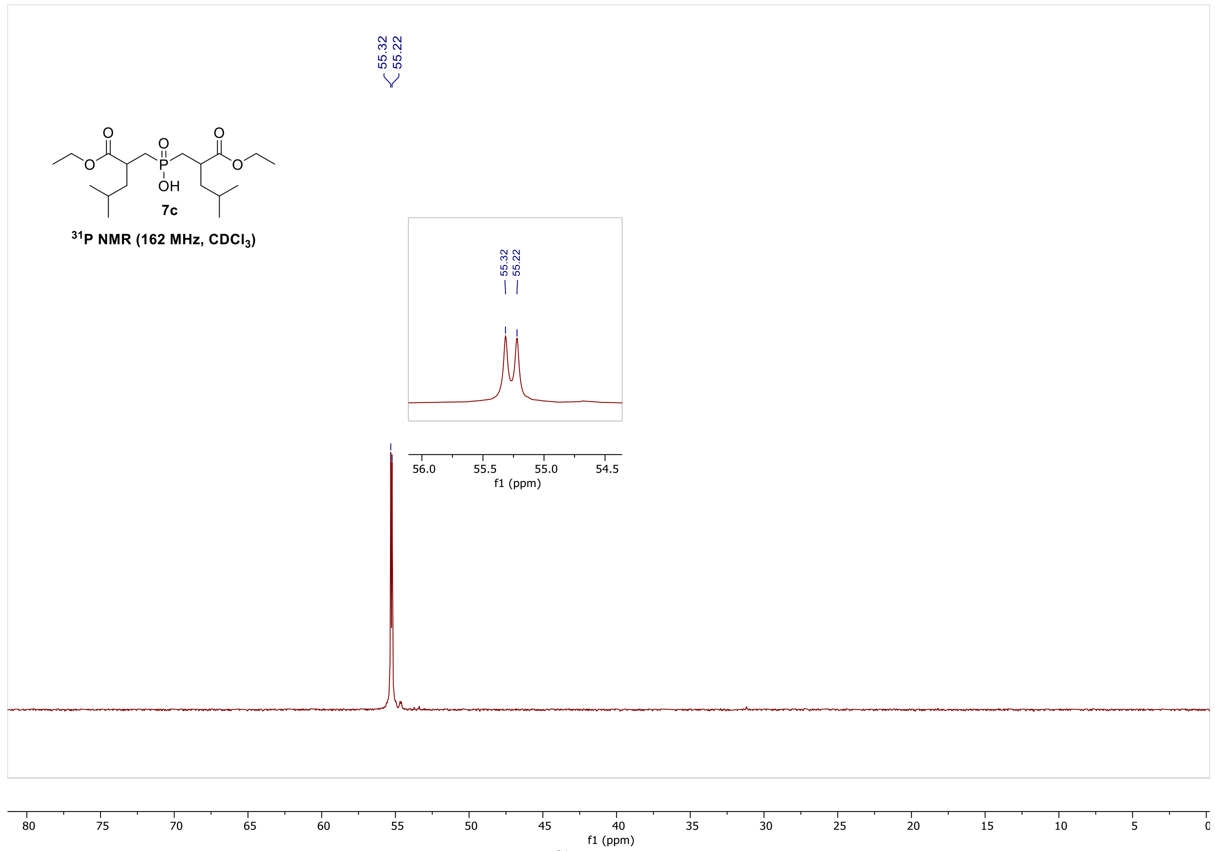

Figure S34. ^{31}*PNMR of compound* ***7c***

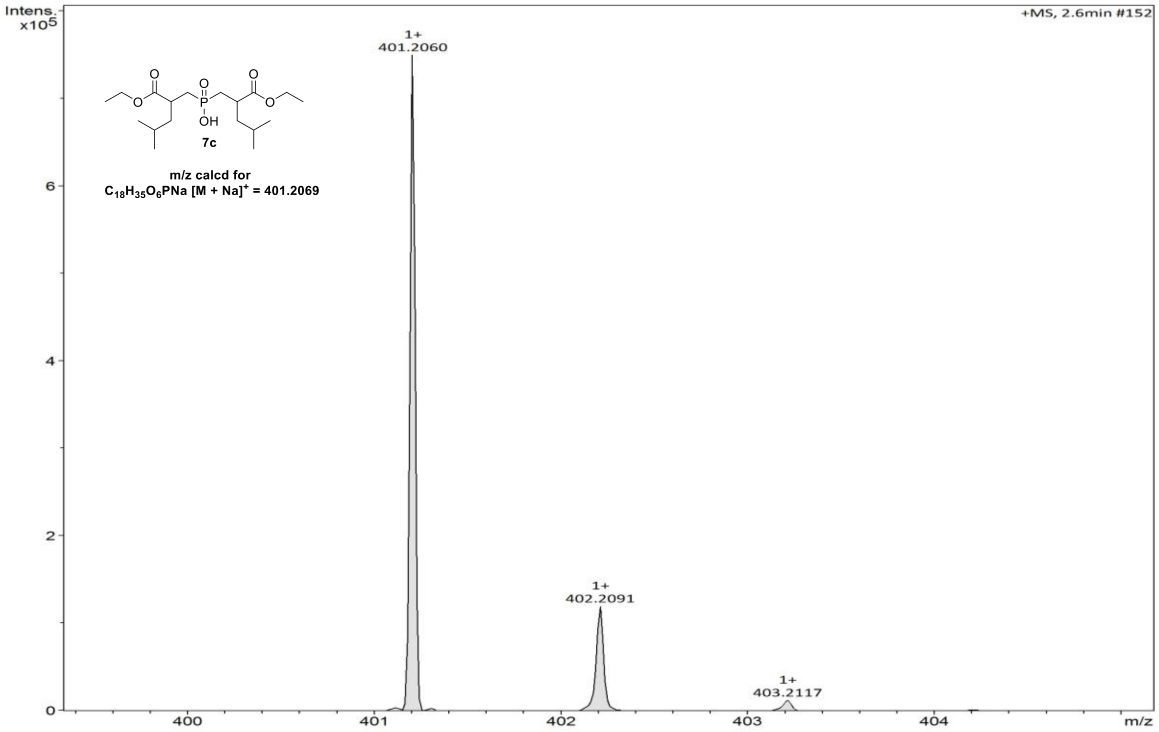

*Figure S35. MS spectrum of compound **7c***

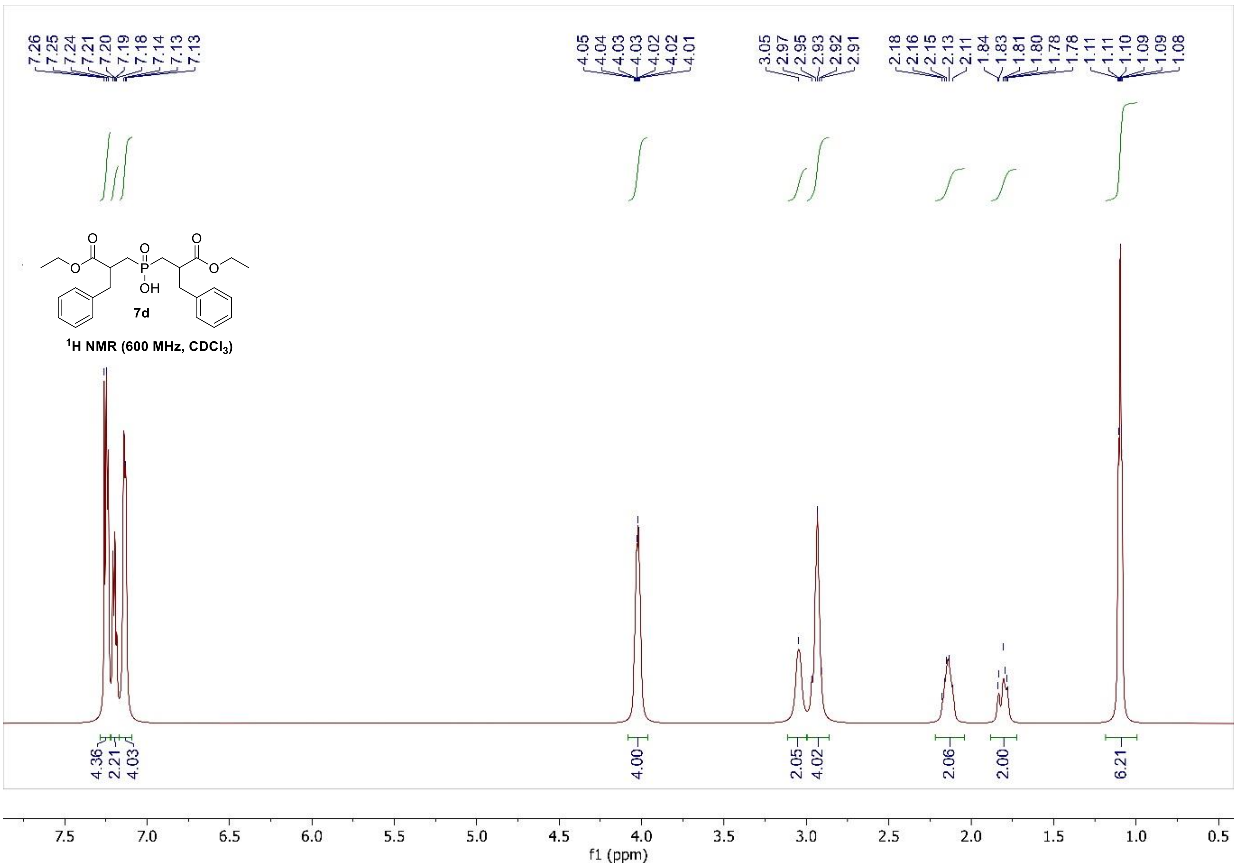

*Figure S36. ¹HNMR of compound **7d***

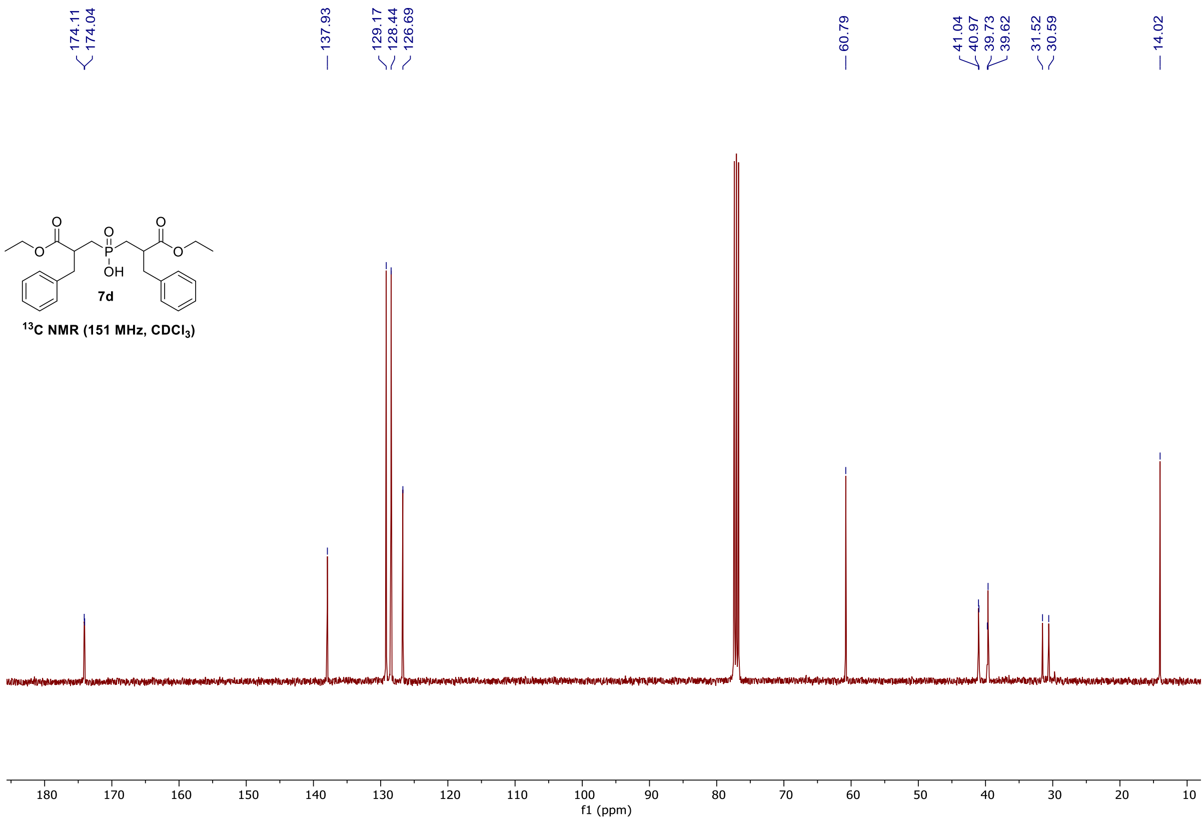

*Figure S37. ^{13}CNMR of compound **7d***

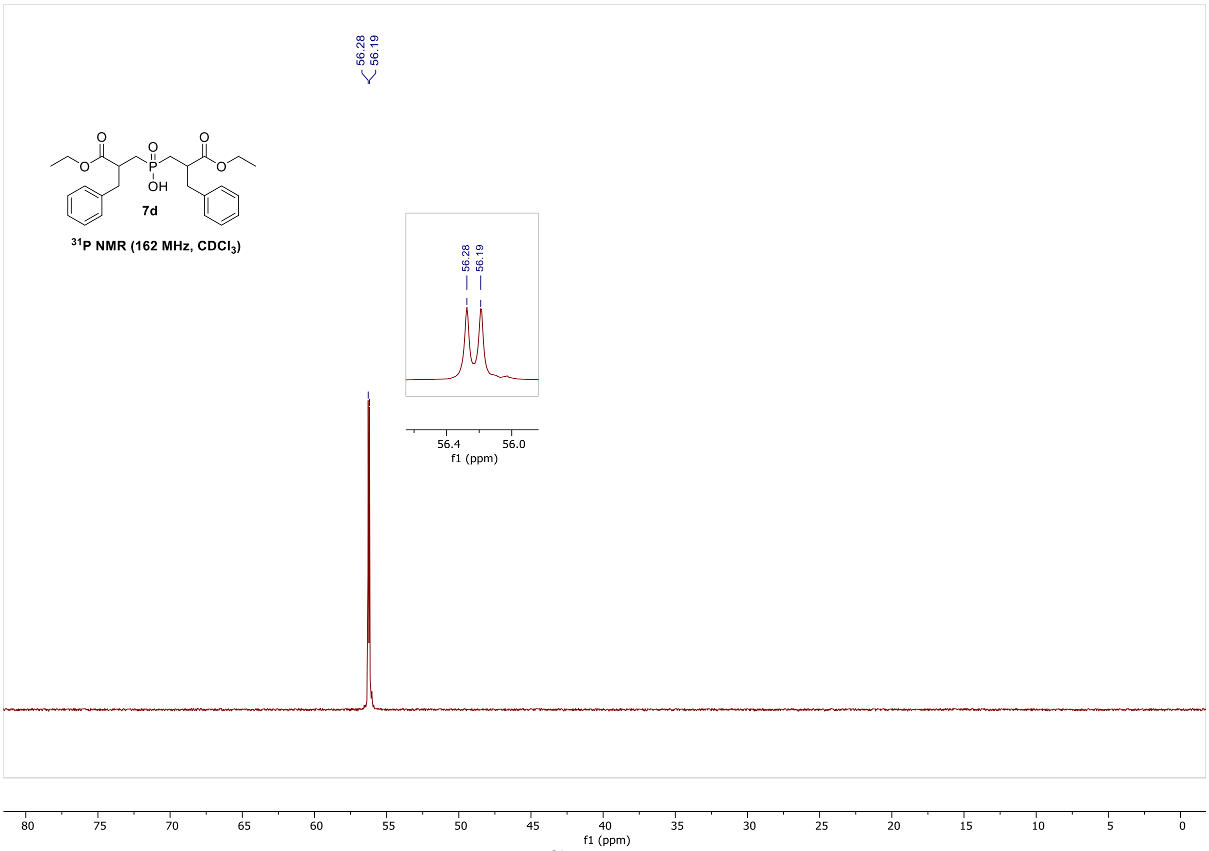

*Figure S38. ^{31}PNMR of compound **7d***

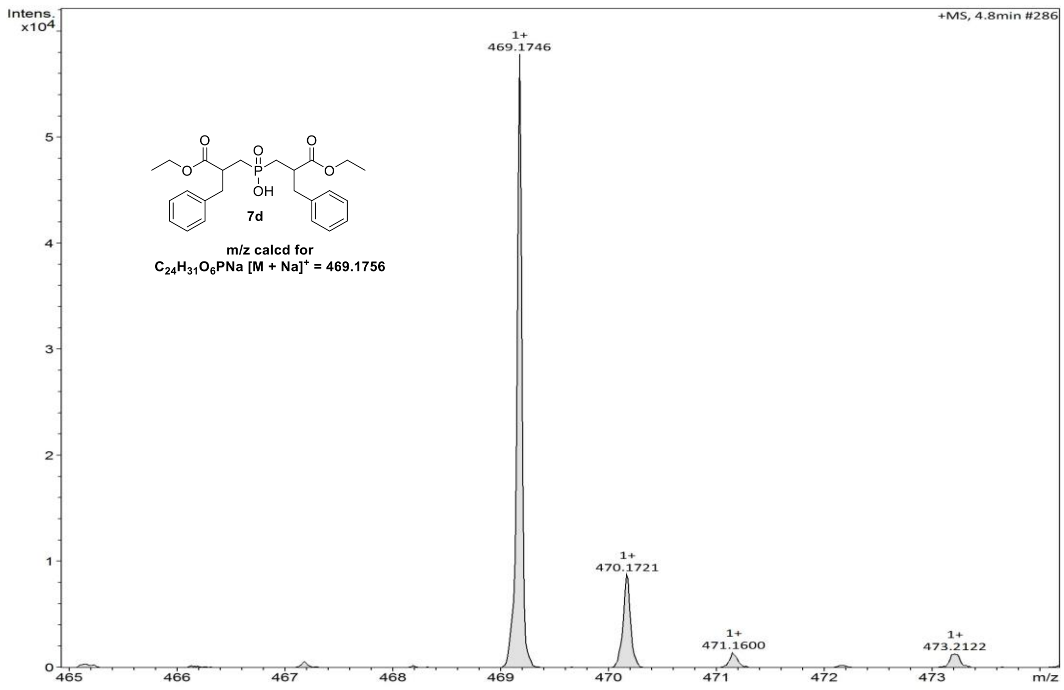

*Figure S39. MS spectrum of compound **7d***

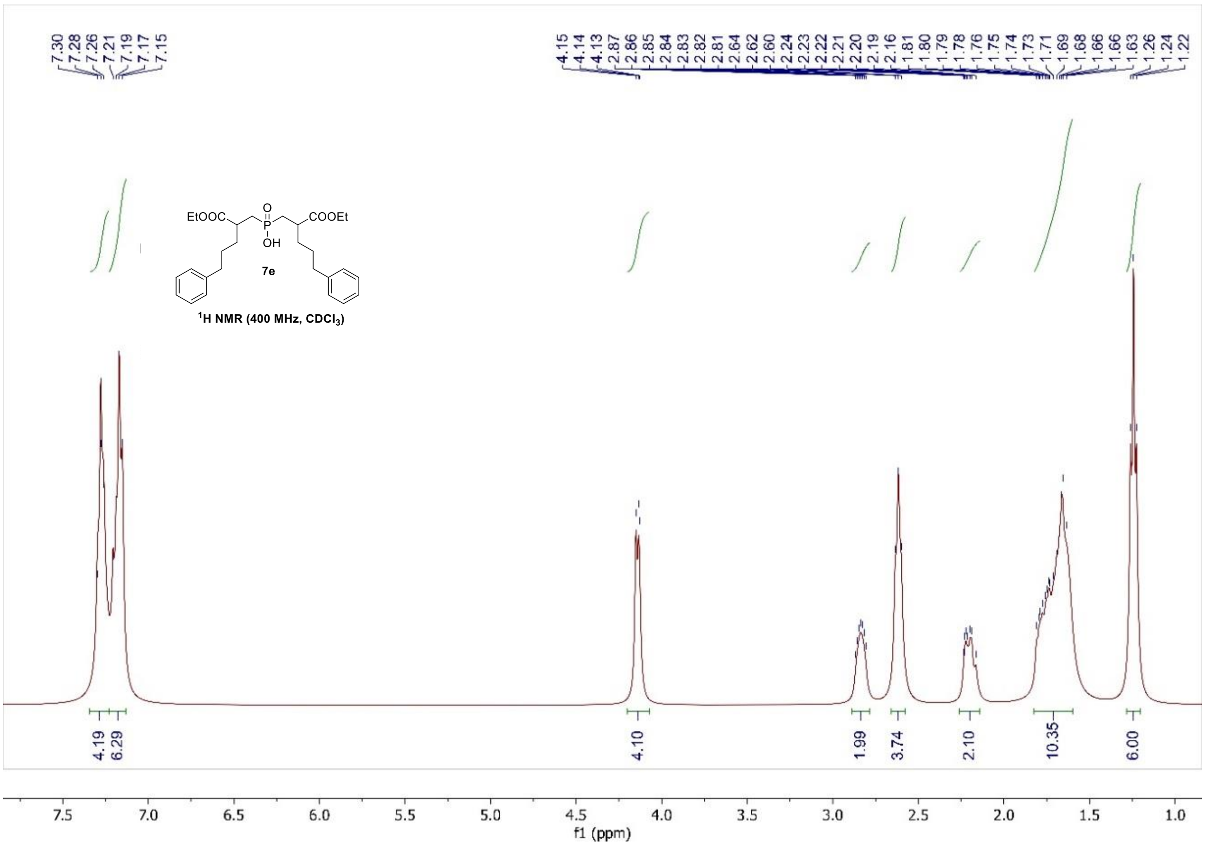

*Figure S40. ^{1}HNMR of compound **7e***

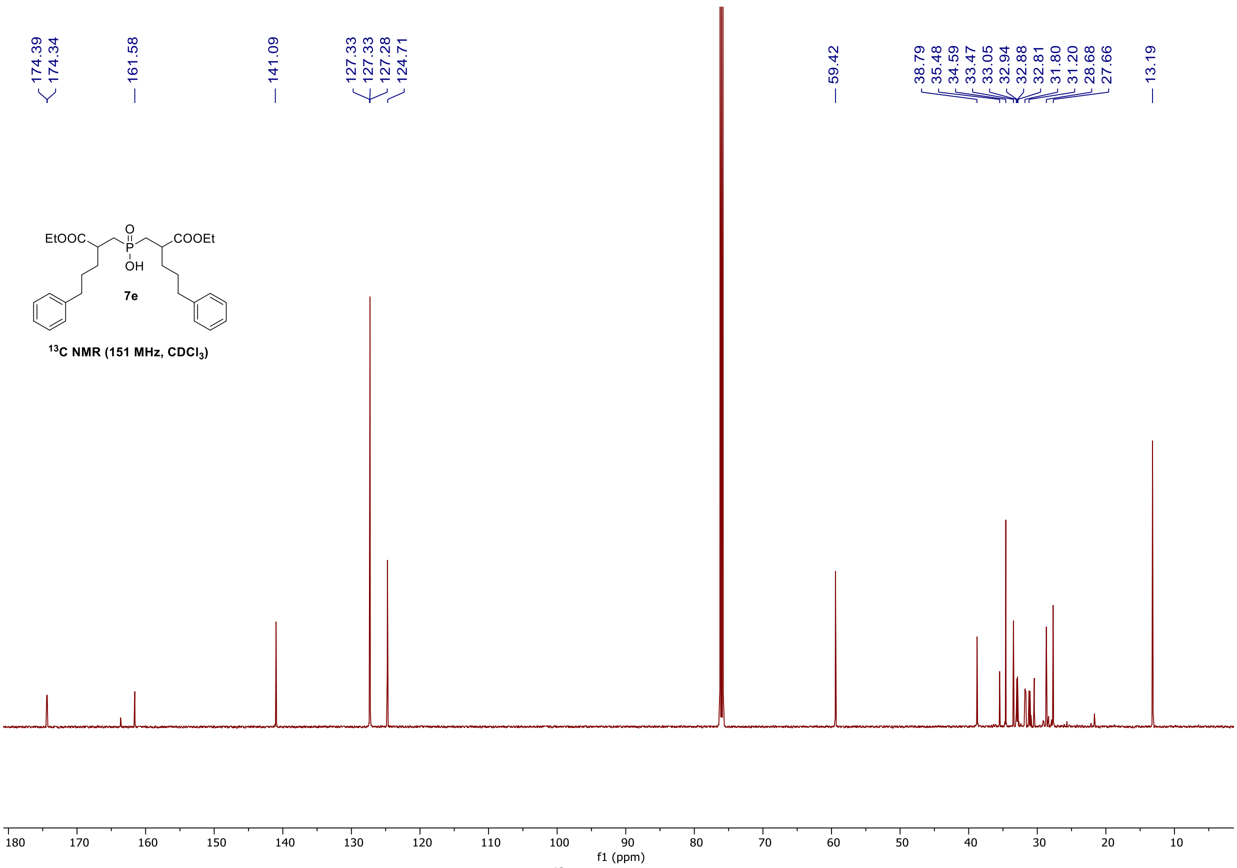

*Figure S41. ^{13}CNMR of compound **7e***

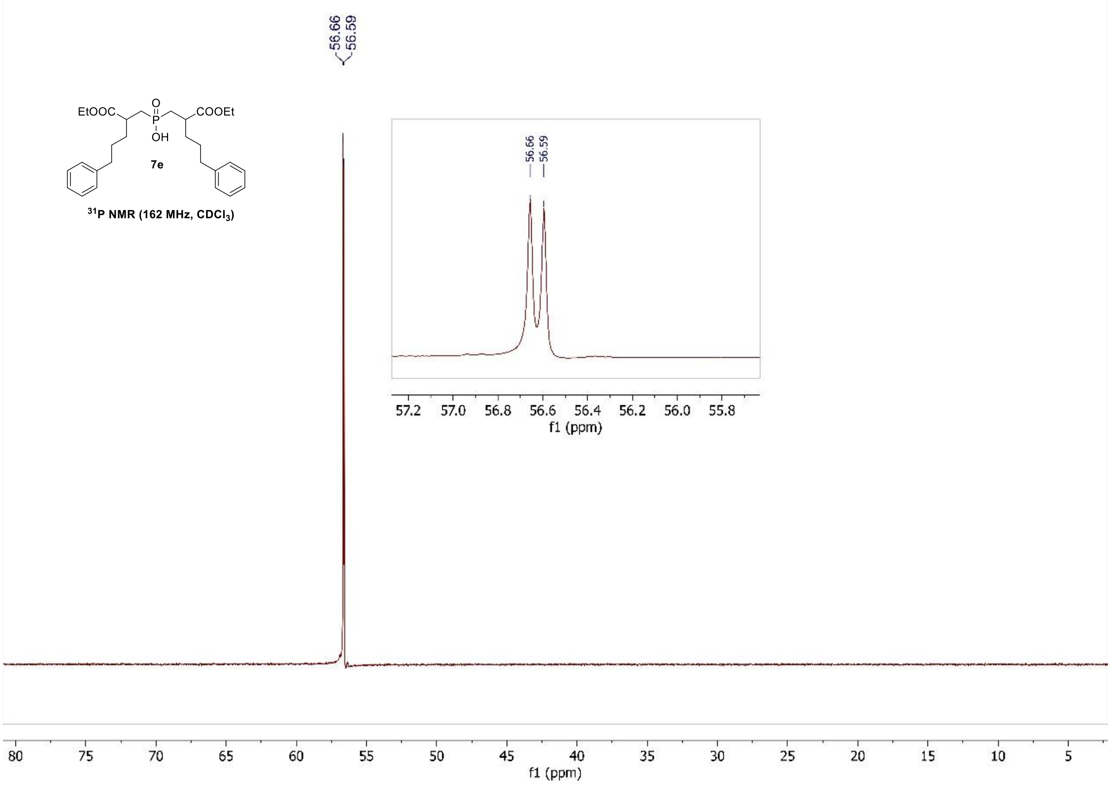

*Figure S42. ^{31}PNMR of compound **7e***

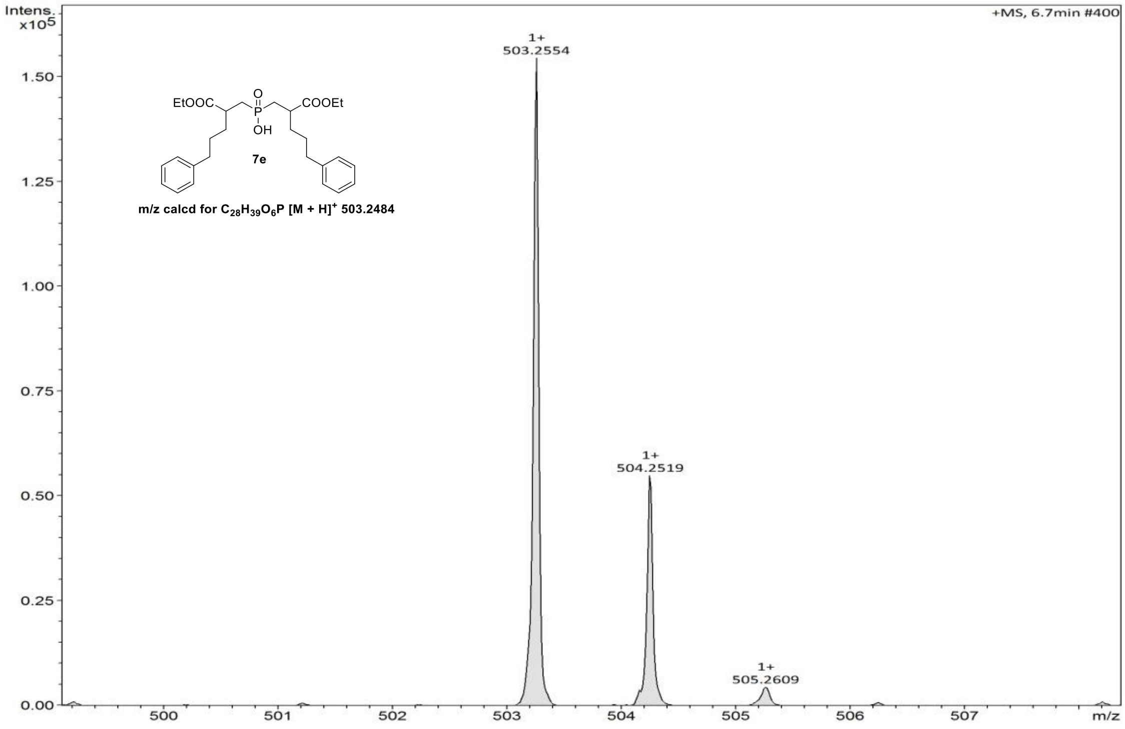

*Figure S43. MS spectrum of compound **7e***

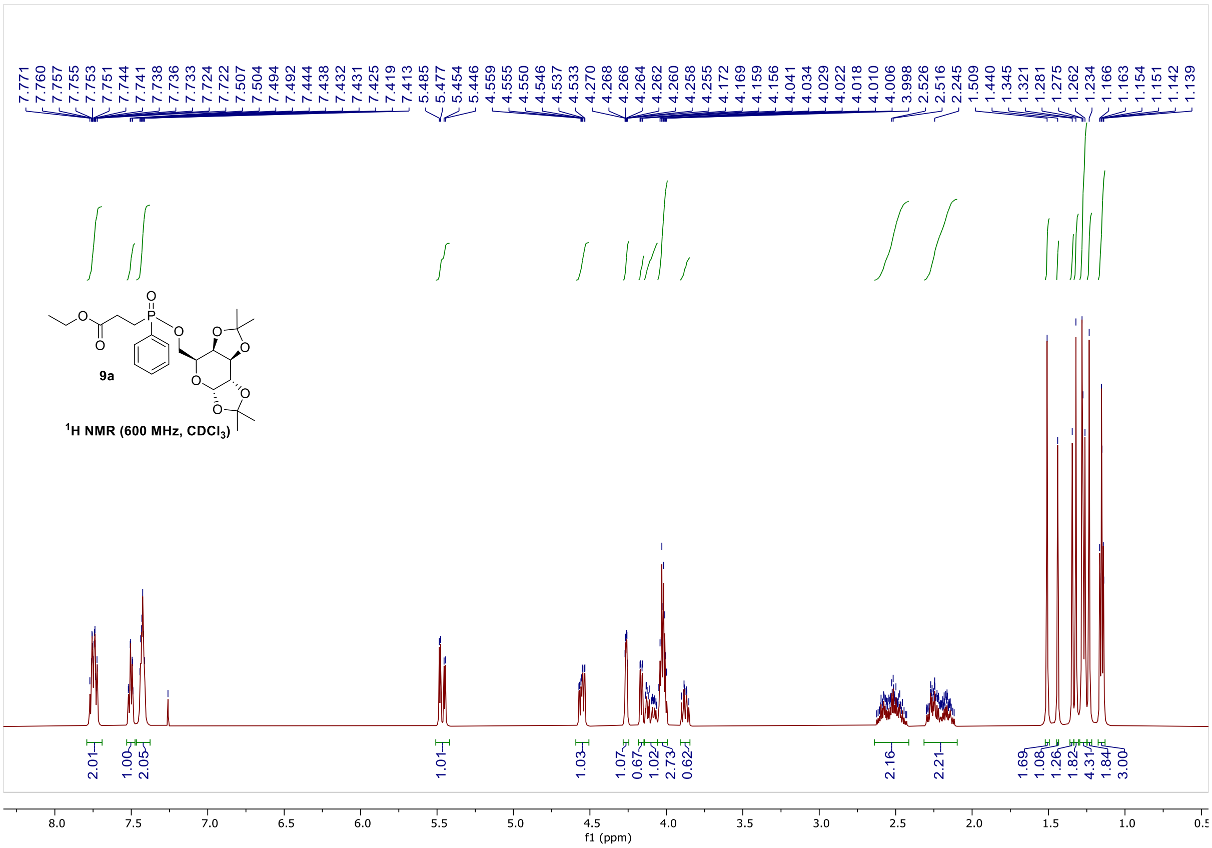

*Figure S44. 1HNMR of compound **9a***

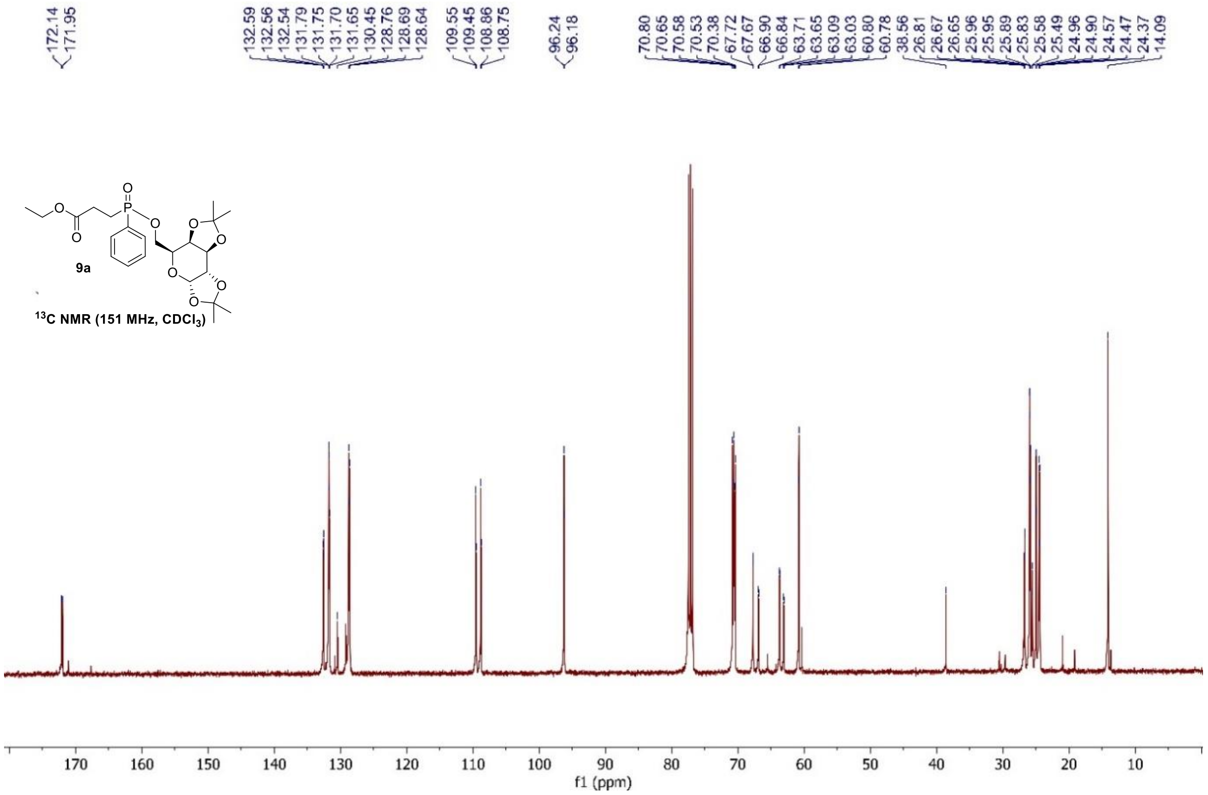

*Figure S45. ^{13}CNMR of compound **9a***

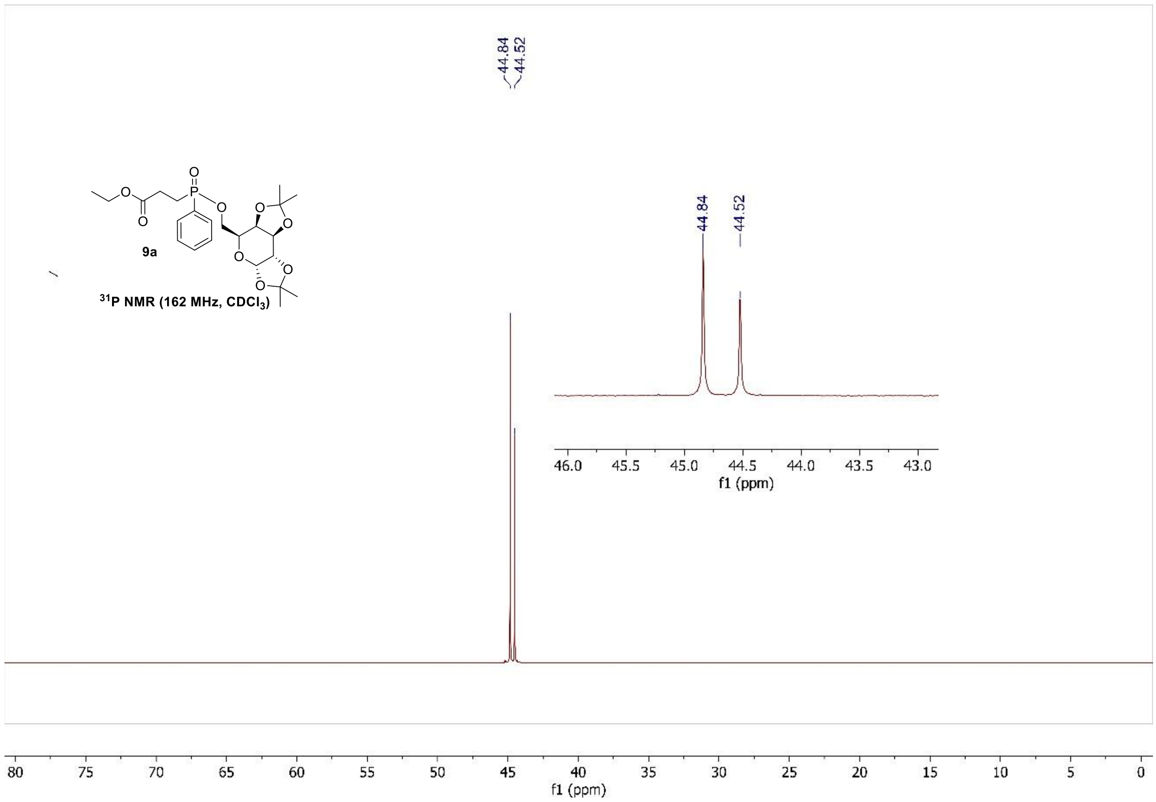

*Figure S46. ^{31}PNMR of compound **9a***

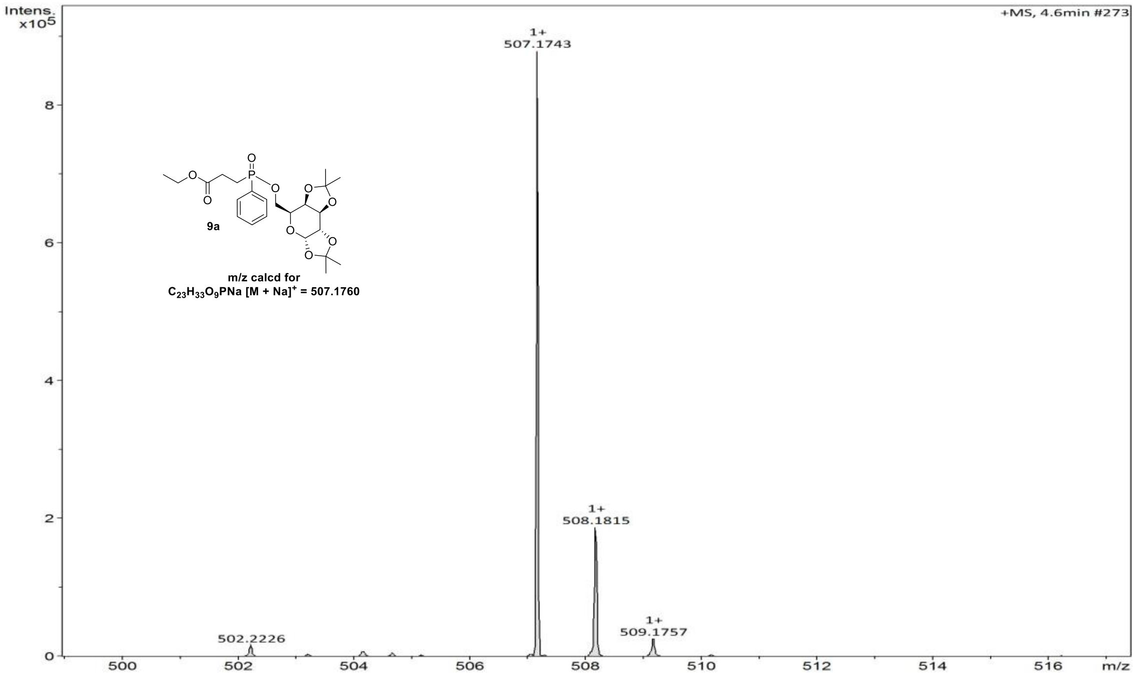

Figure S47. MS spectrum of compound ***9a***

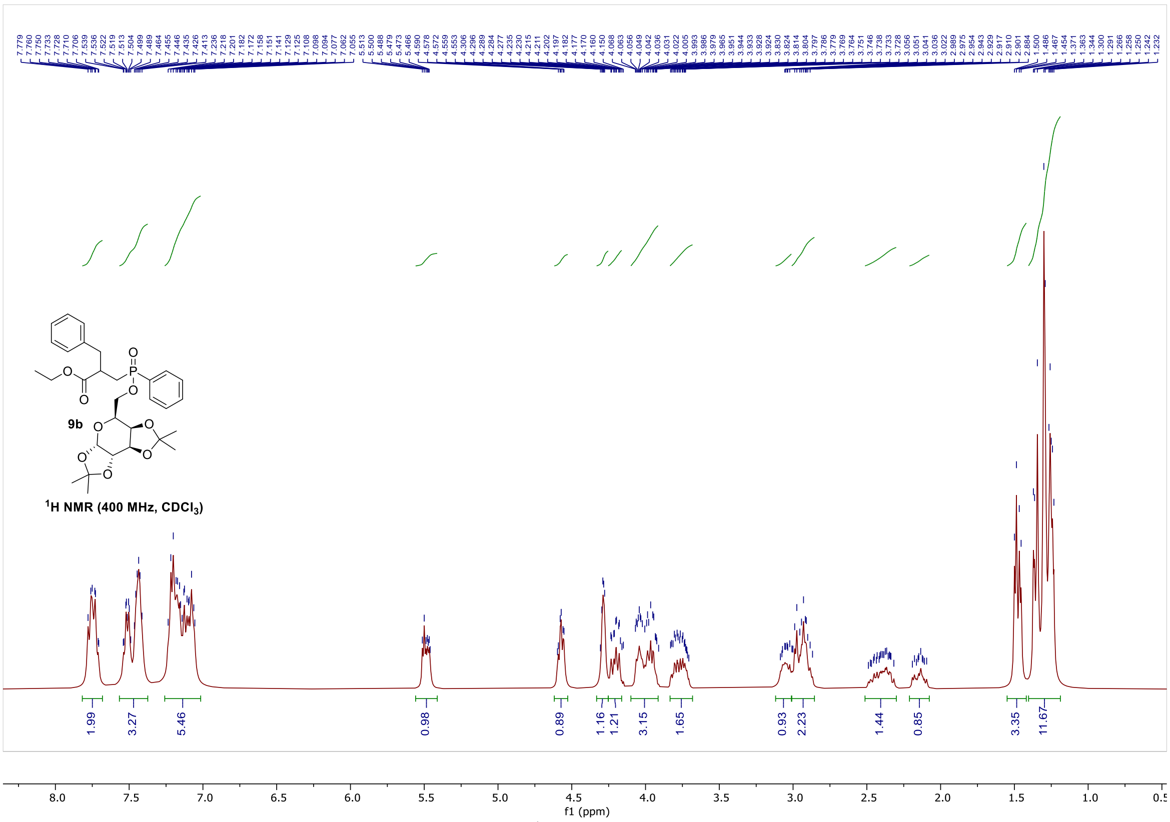

Figure S48. ^{1}HNMR of compound ***9b***

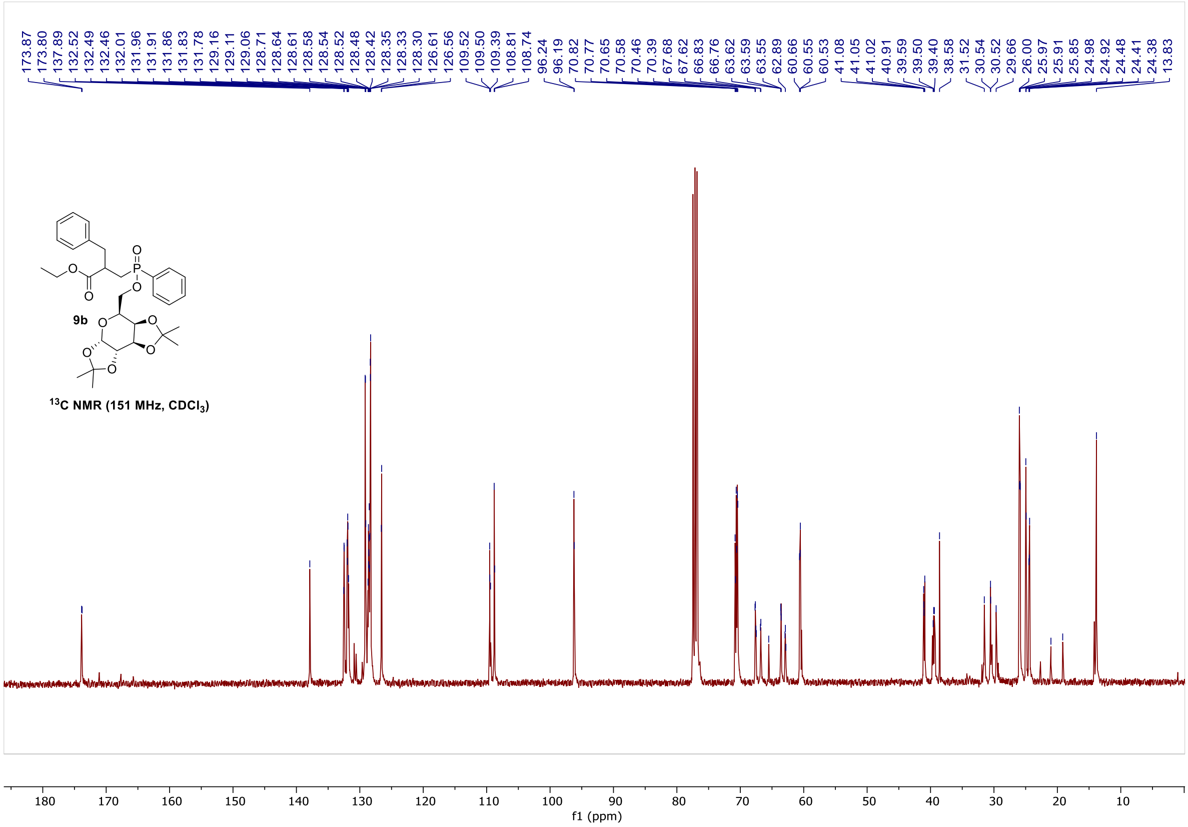

Figure S49. ^{13}CNMR of compound ***9b***

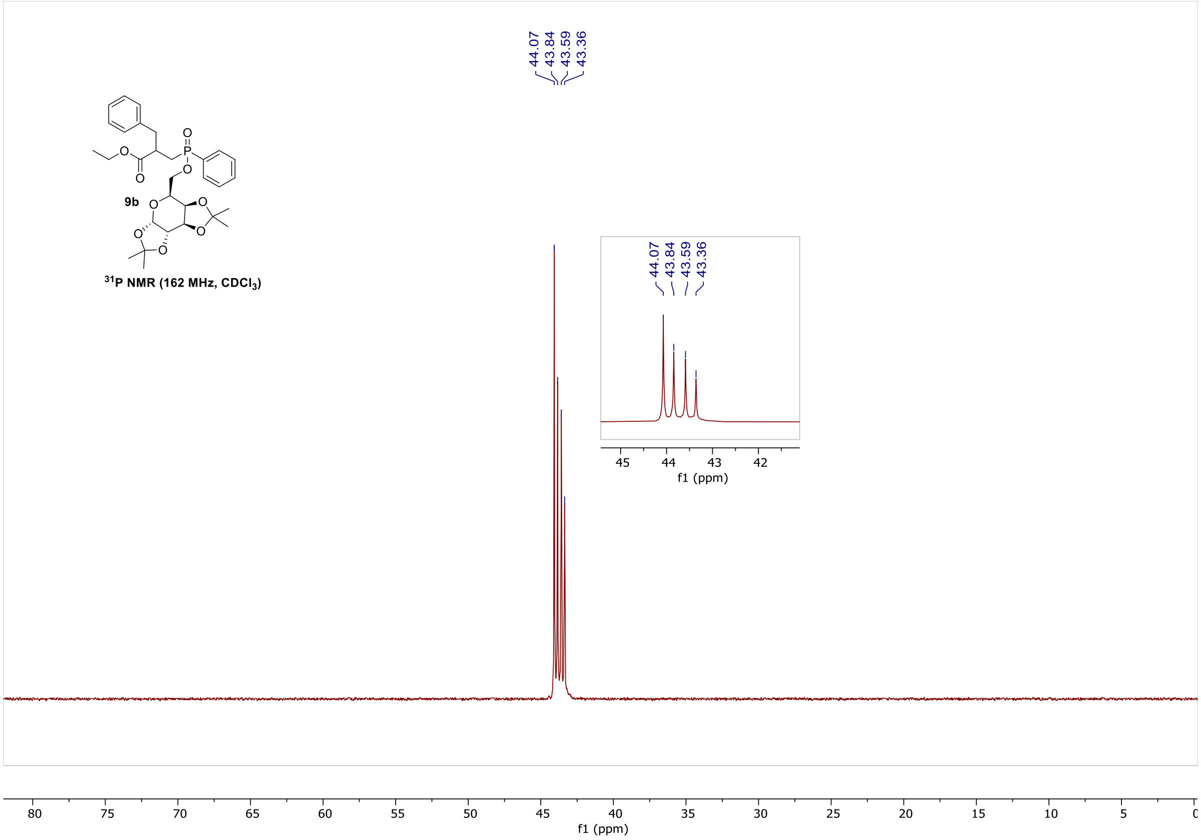

Figure S50. ^{31}PNMR of compound ***9b***

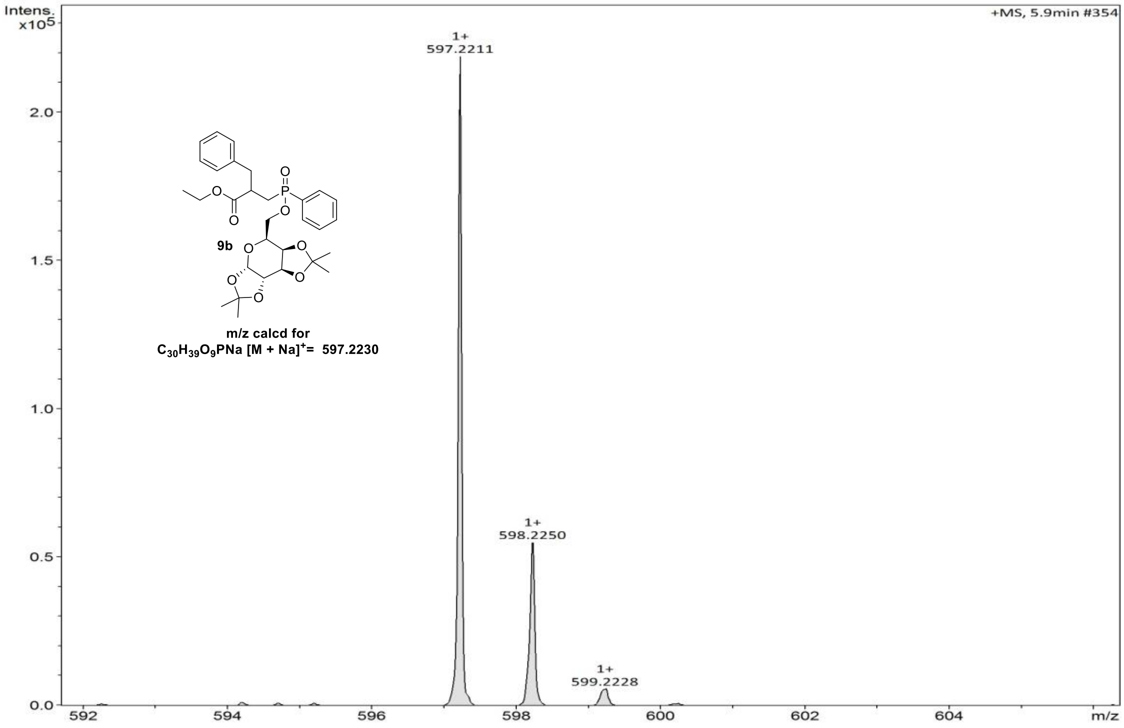

*Figure S51. MS spectrum of compound **9b***

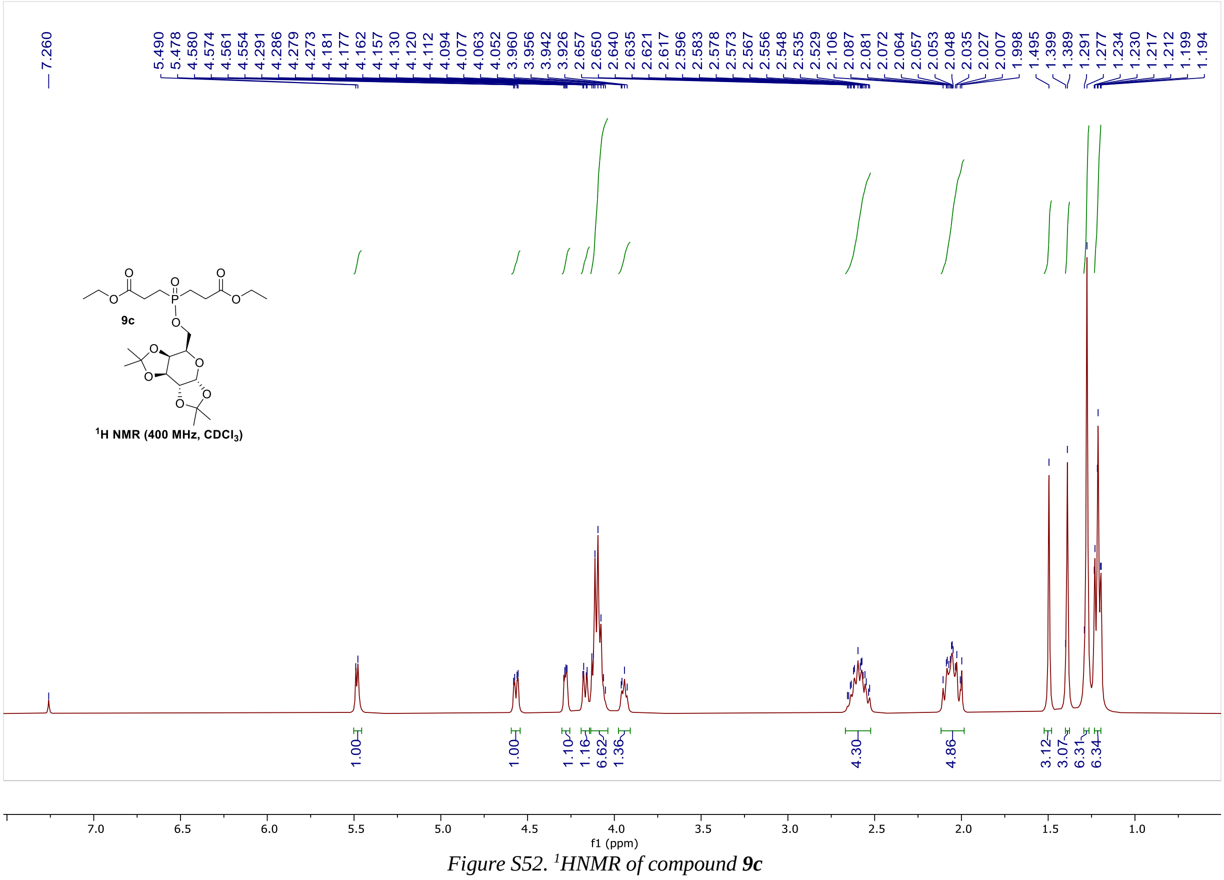

*Figure S52. ^{1}HNMR of compound **9c***

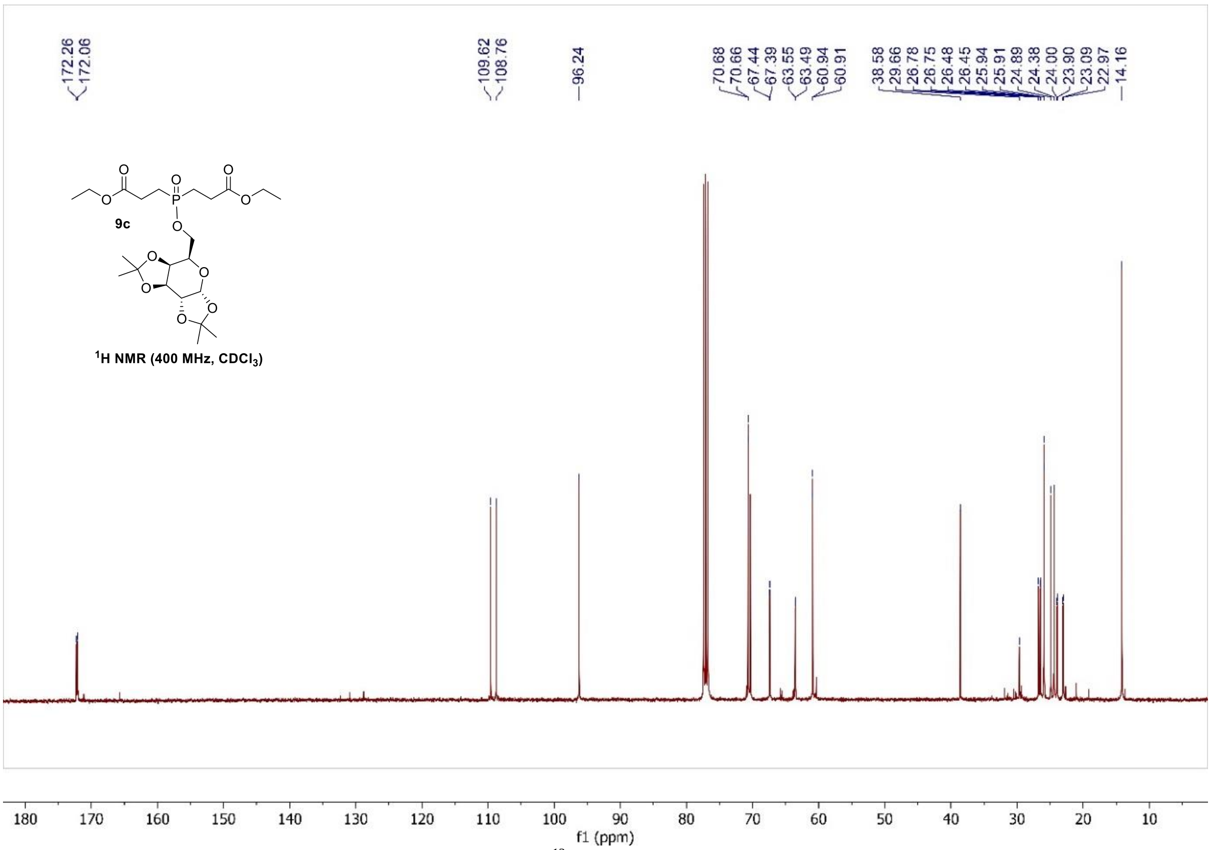

Figure S53. ^{13}CNMR of compound **9c**

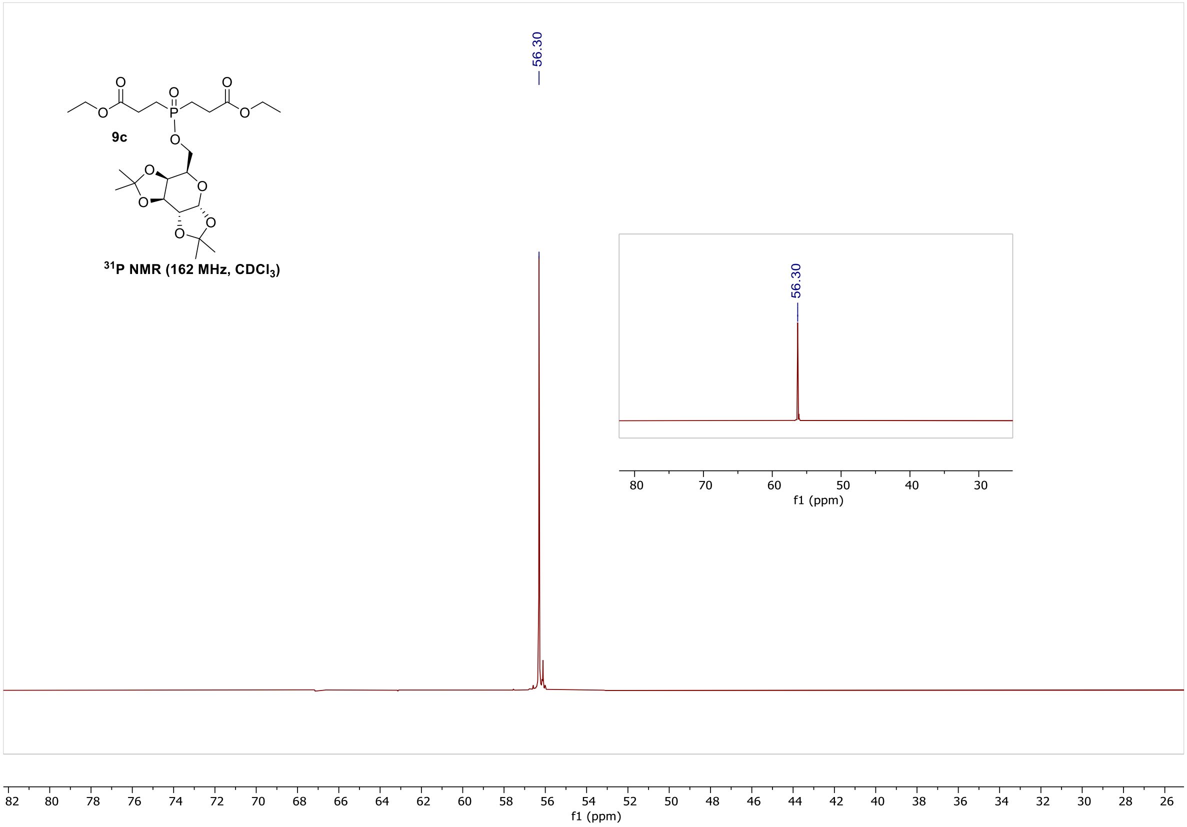

Figure S54. ^{31}PNMR of compound **9c**

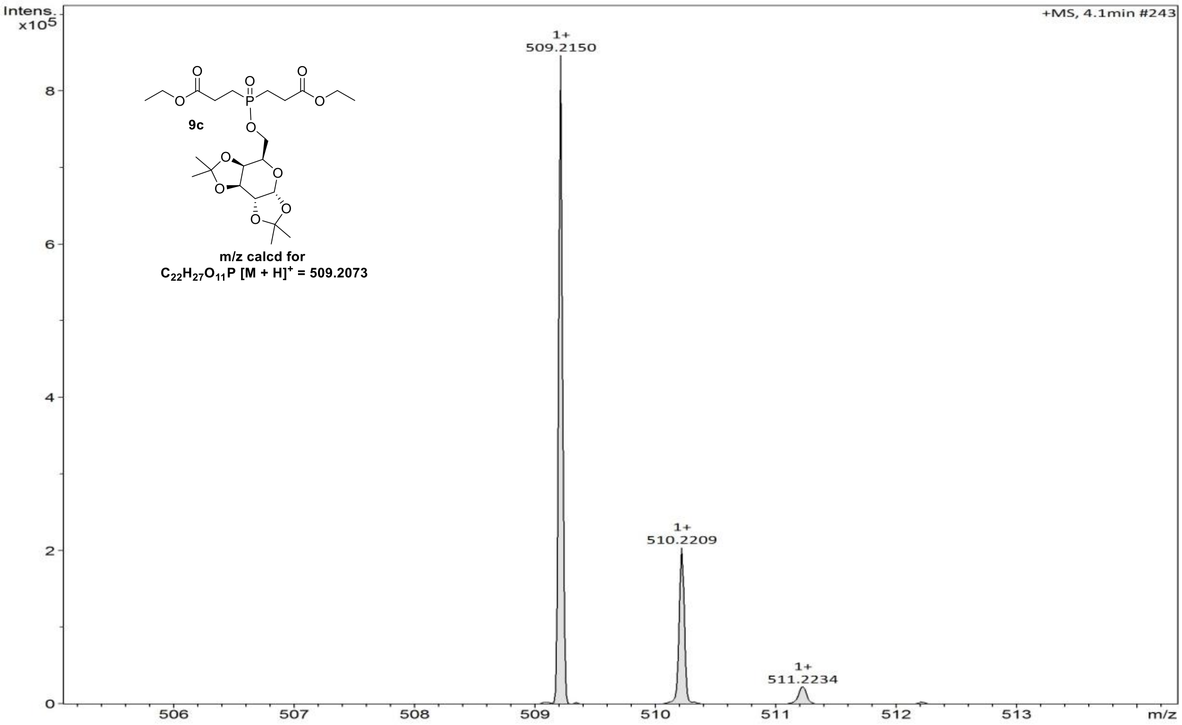

*Figure S55. MS spectrum of compound **9c***

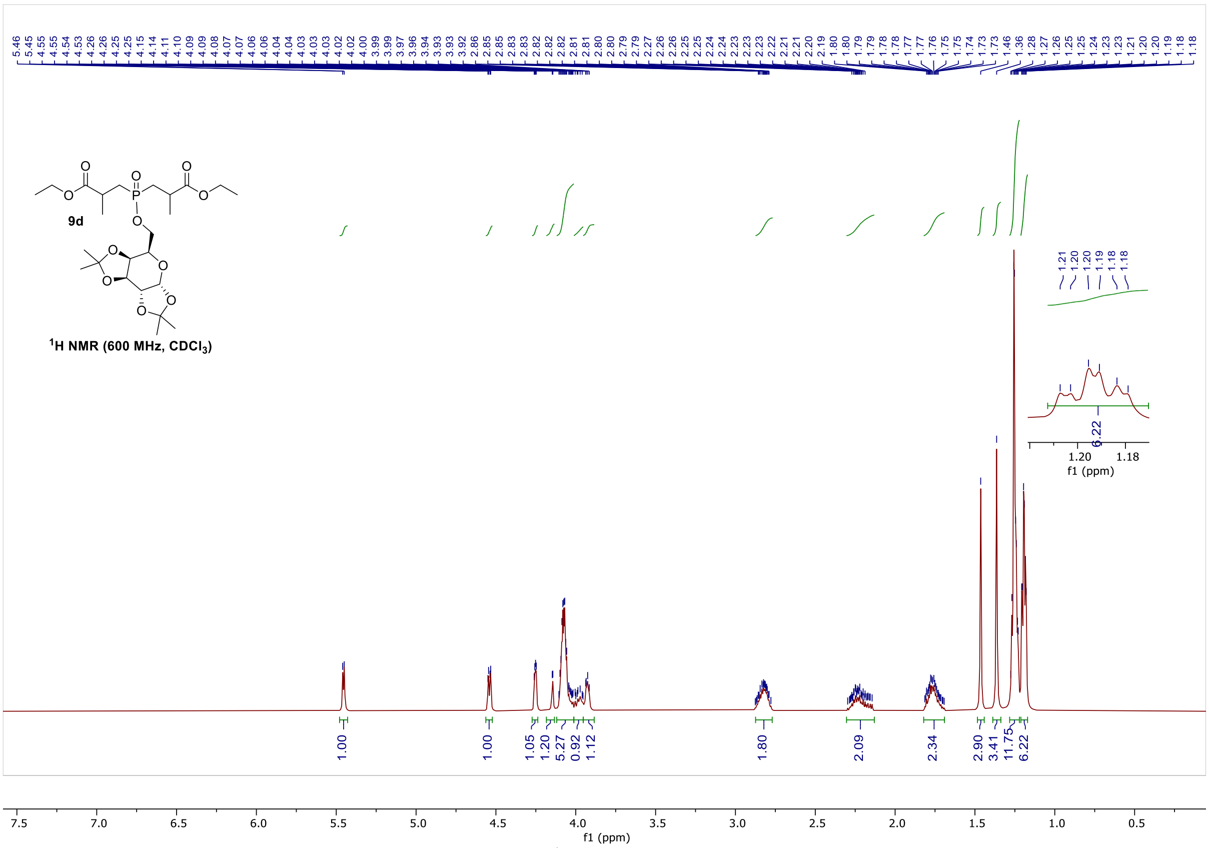

*Figure S56. ^{1}HNMR of compound **9d***

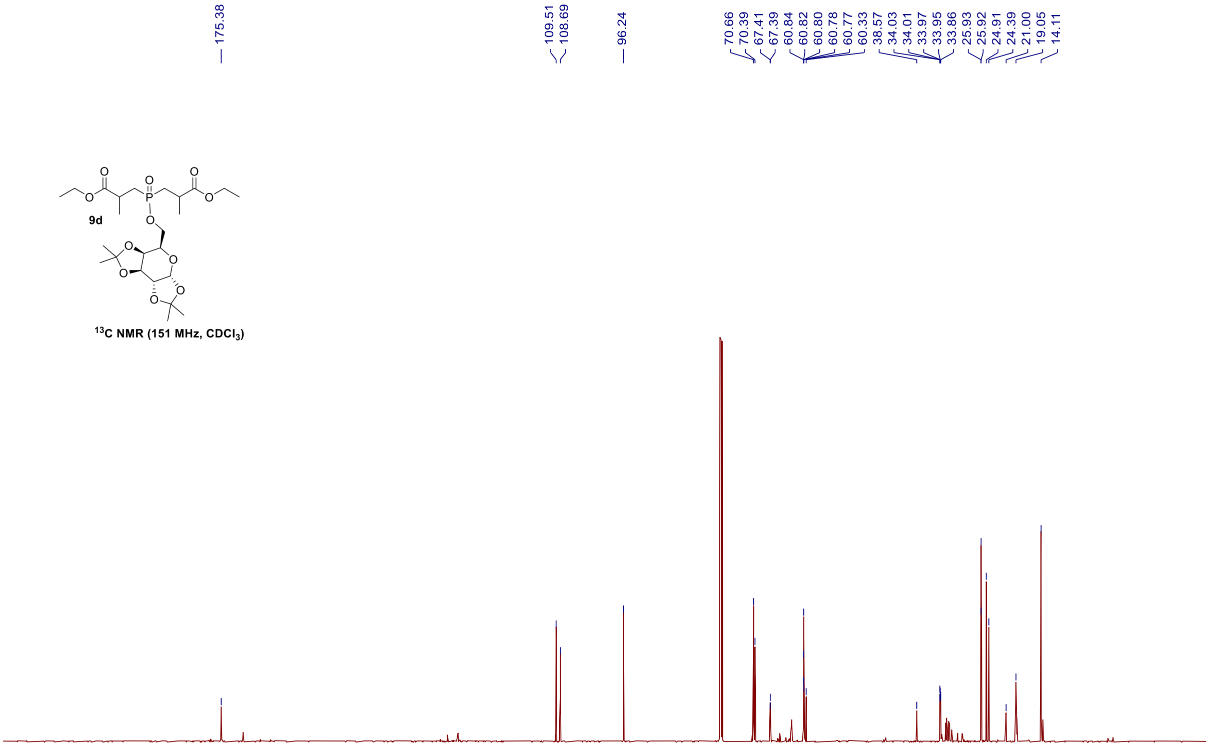

Figure S57. ^{13}CNMR of compound ***9d***

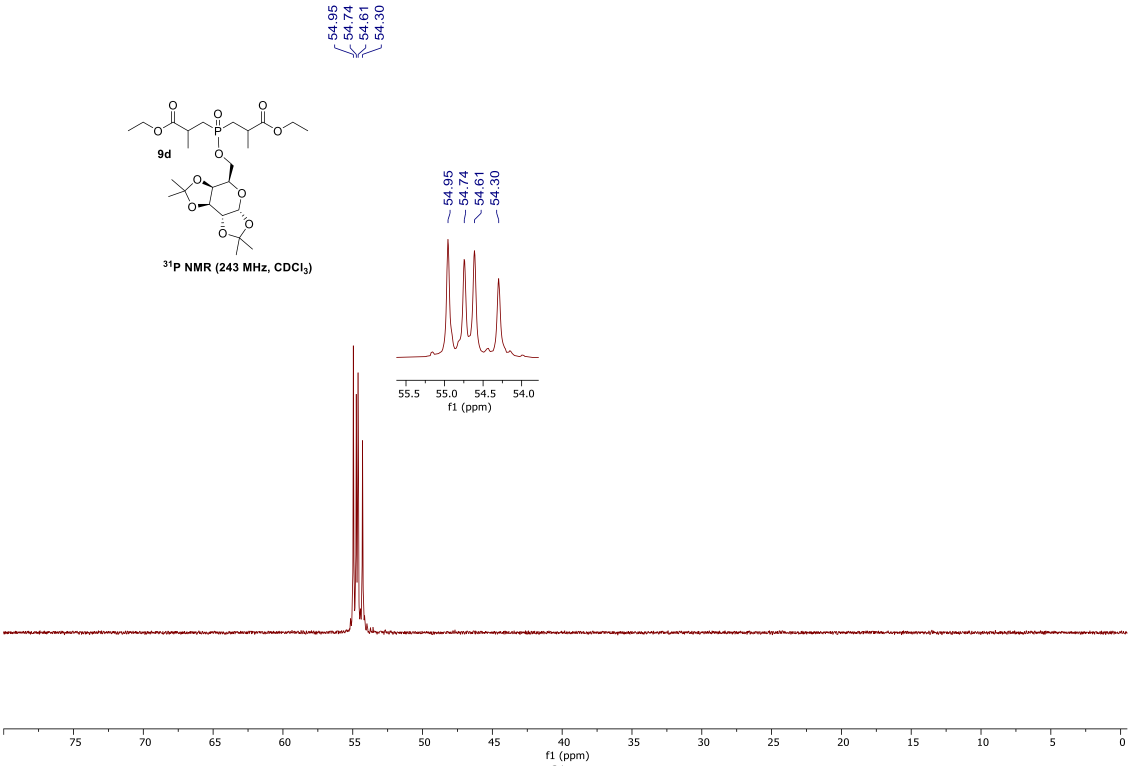

Figure S58. ^{31}PNMR of compound ***9d***

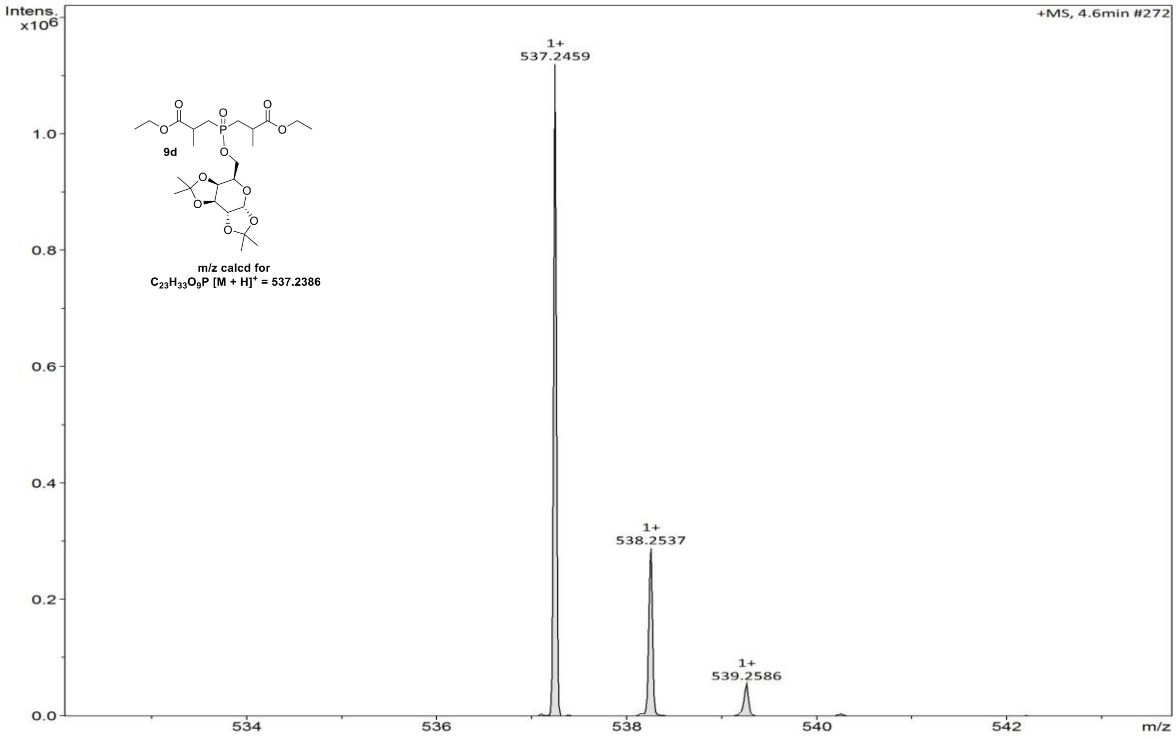

Figure S59. MS spectrum of compound ***9d***

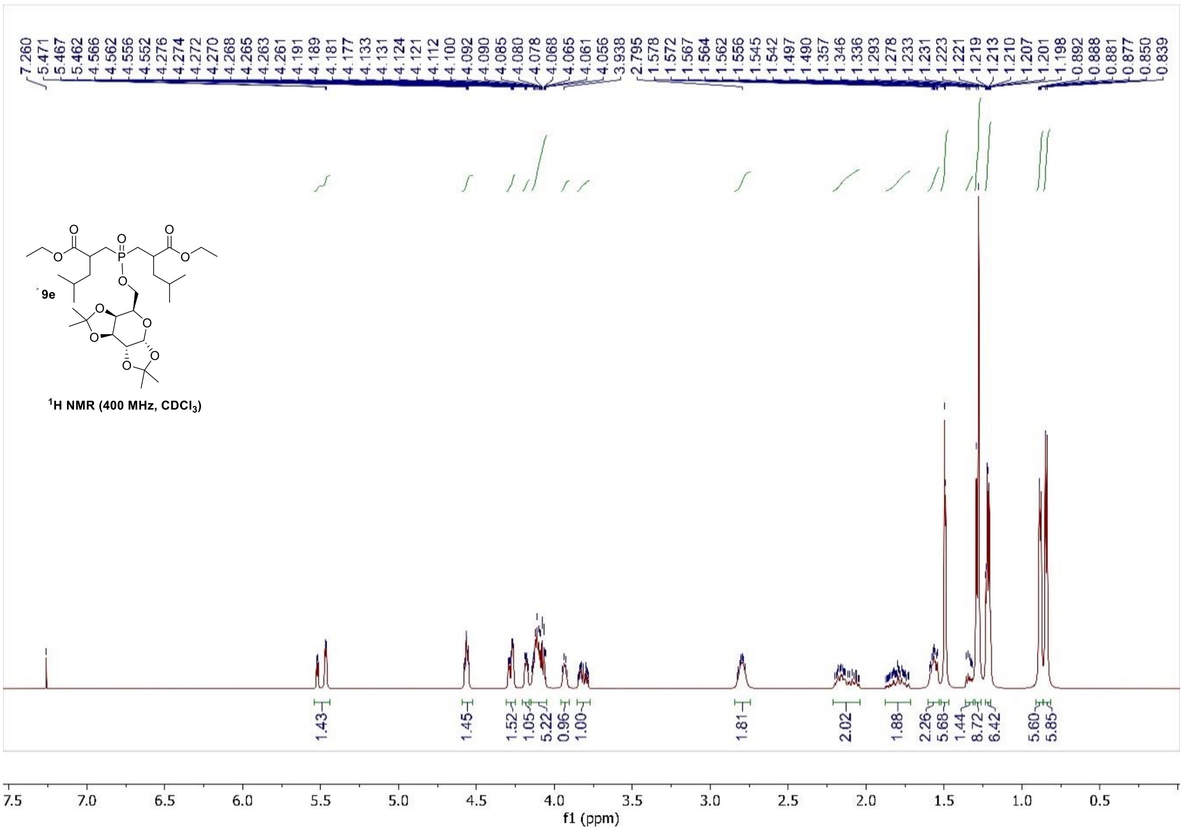

Figure S60. ^{1}HNMR of compound ***9e***

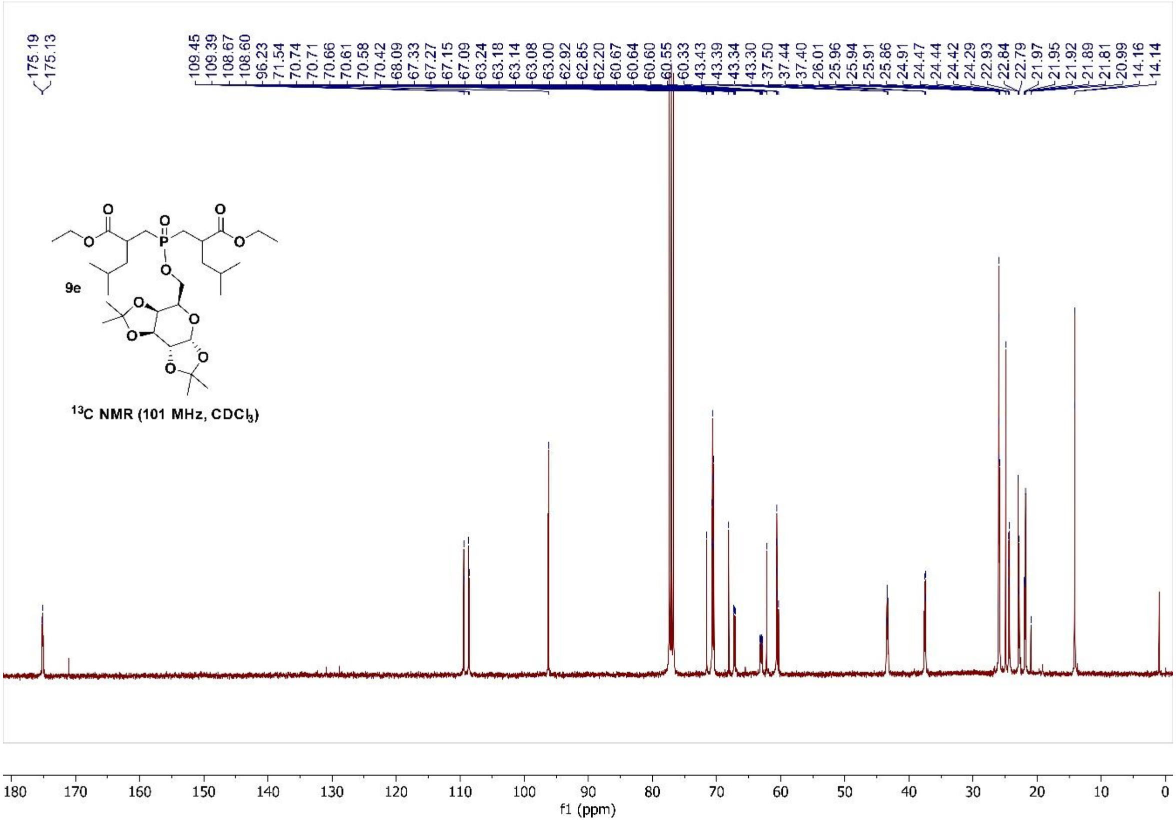

Figure S61. ^{13}CNMR of compound ***9e***

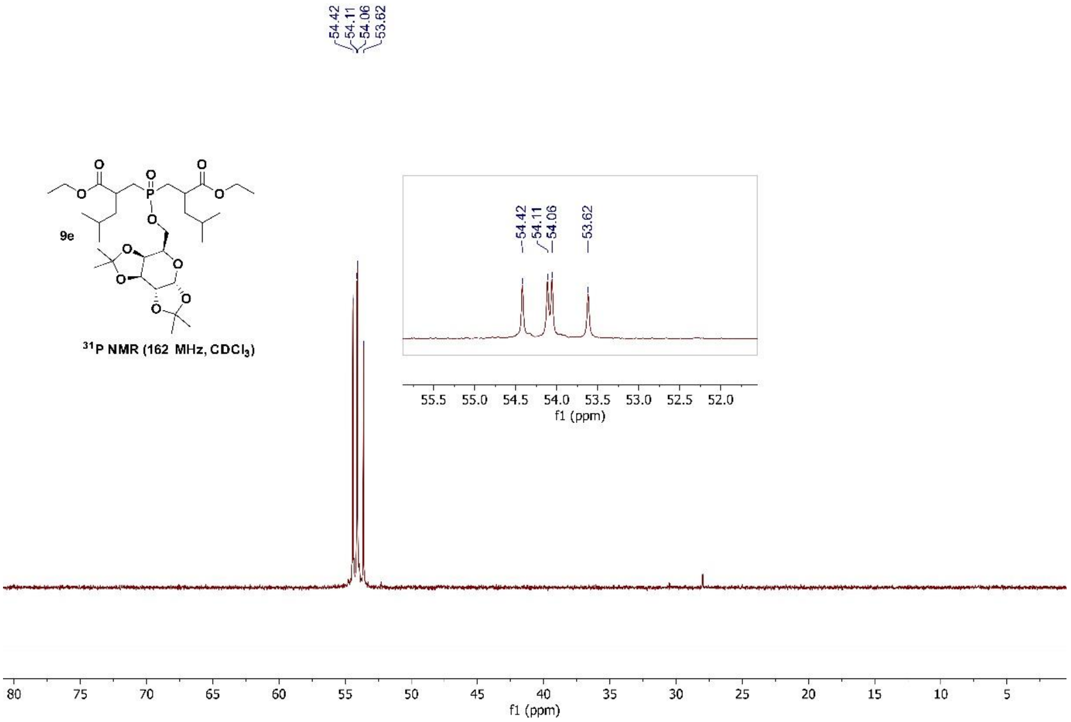

Figure S62. ^{31}PNMR of compound ***9e***

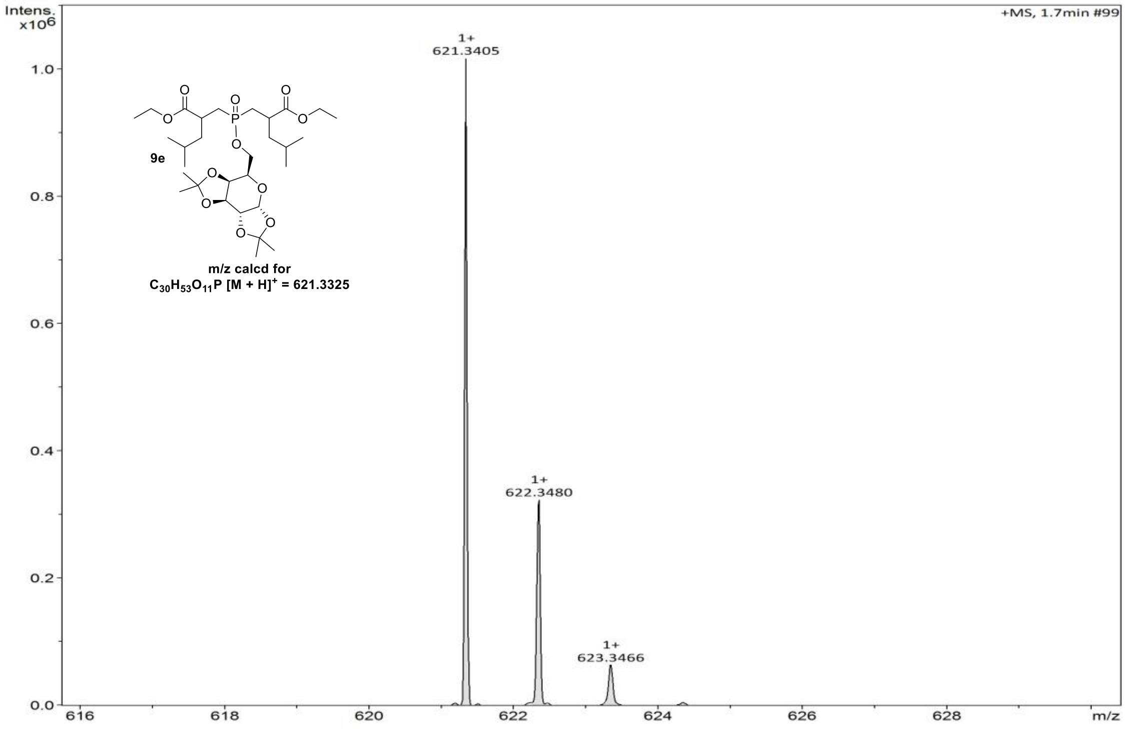

*Figure S63. MS spectrum of compound **9e***

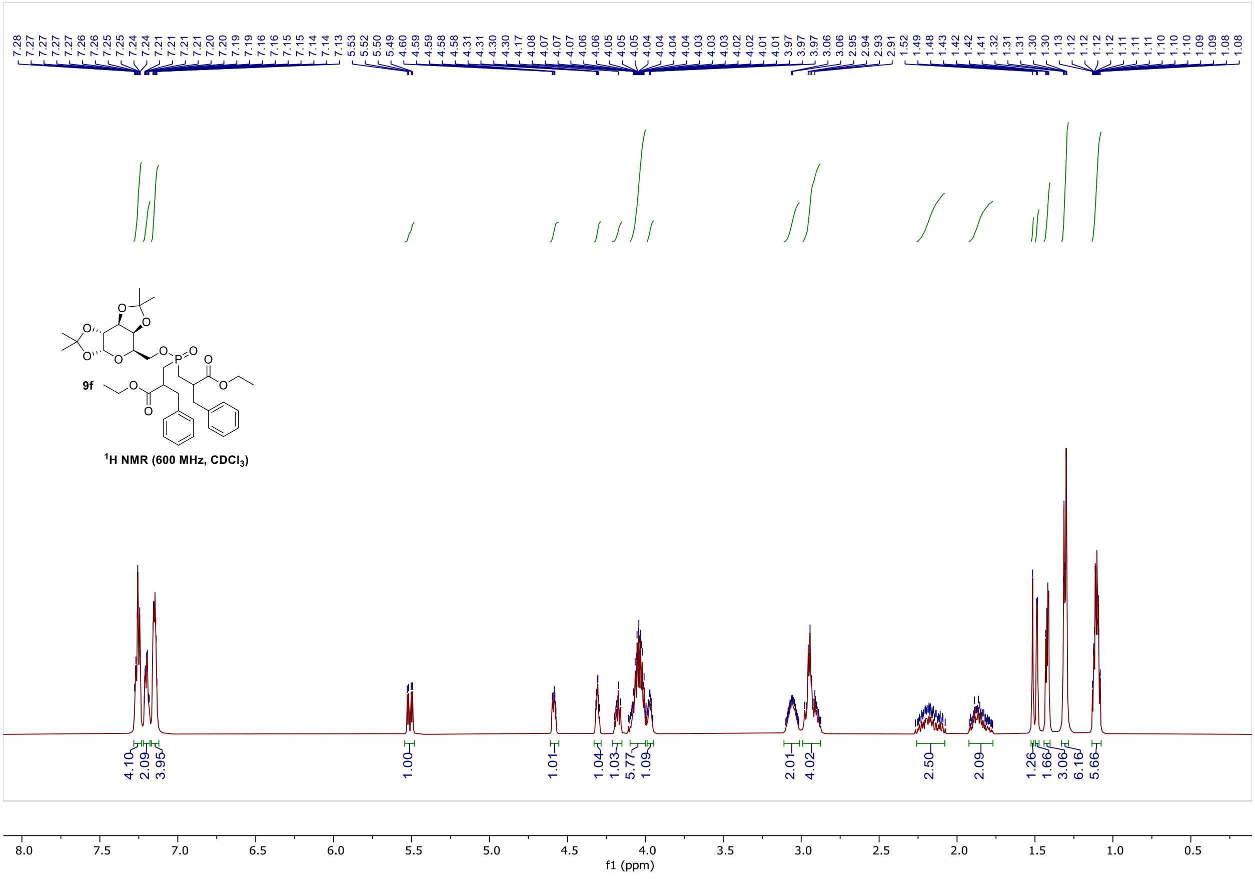

*Figure S64. ^{1}HNMR of compound **9f***

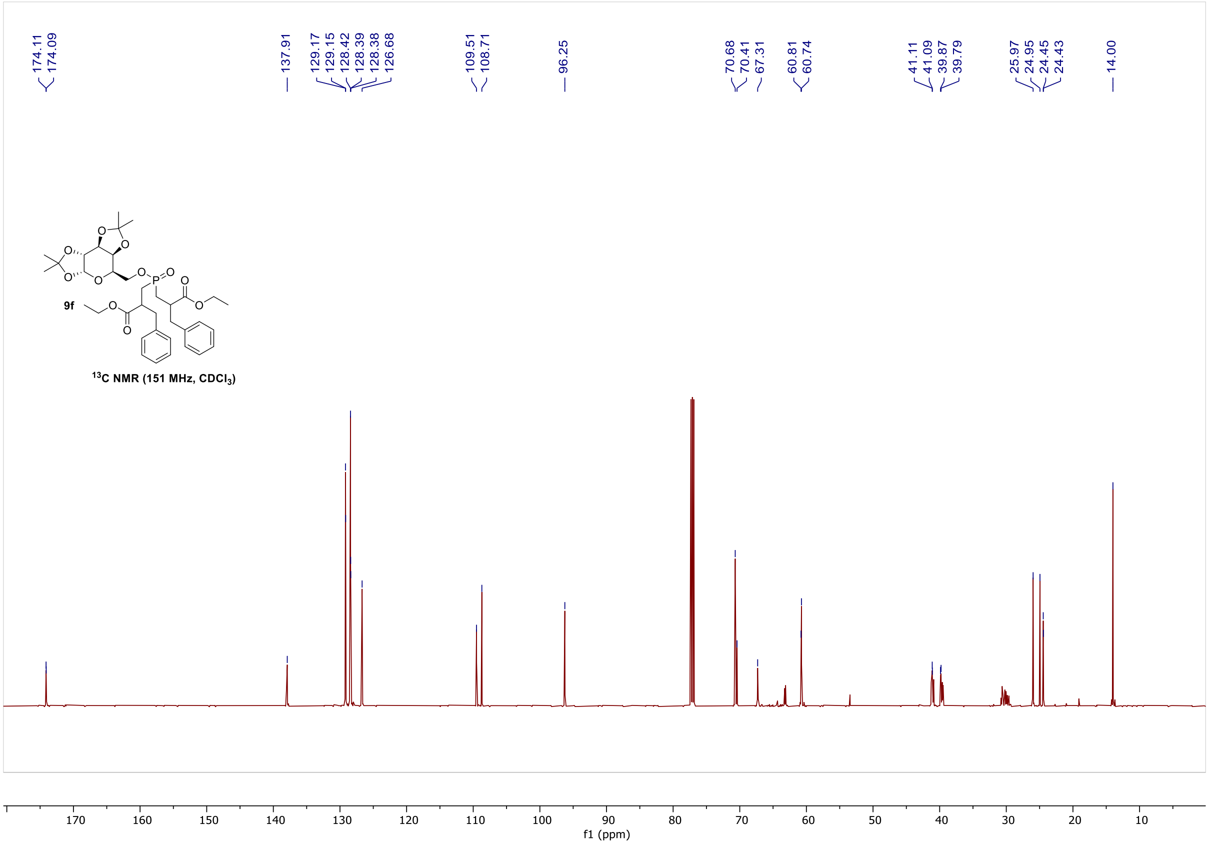

*Figure S65. ^{13}CNMR of compound **9f***

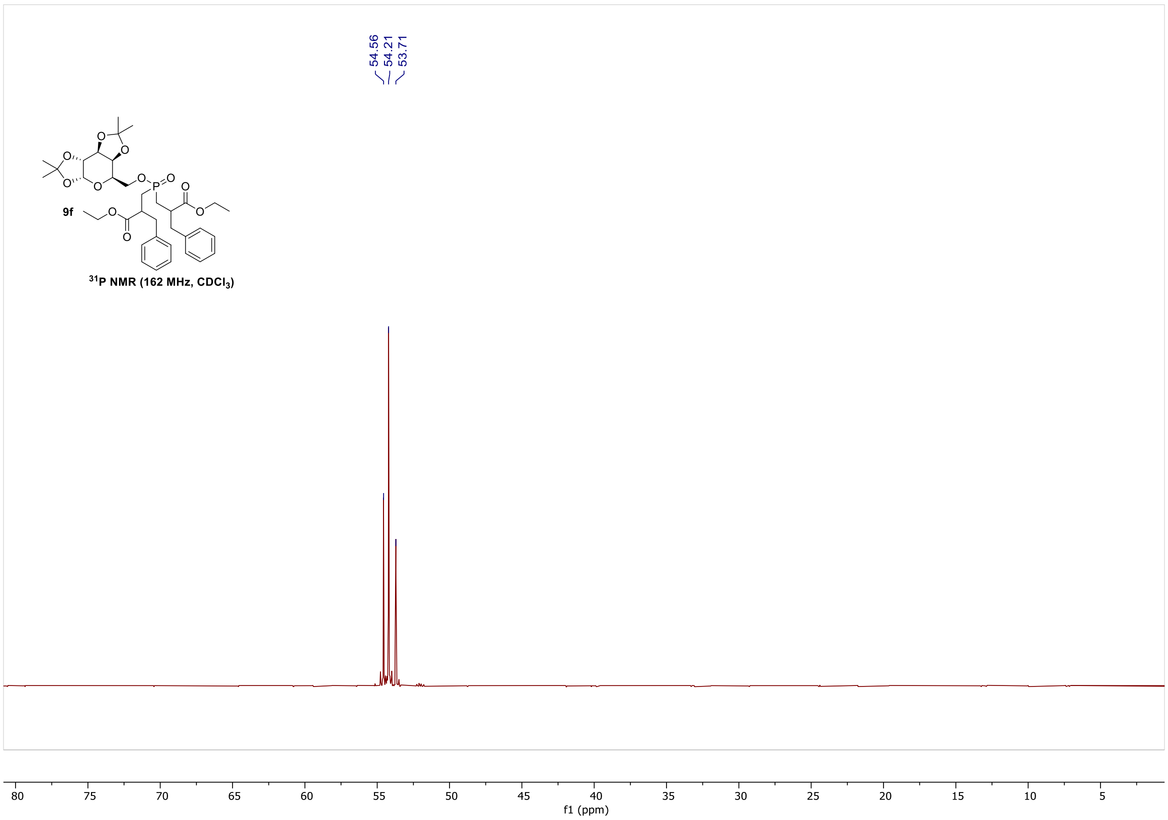

*Figure S66. ^{31}PNMR of compound **9f***

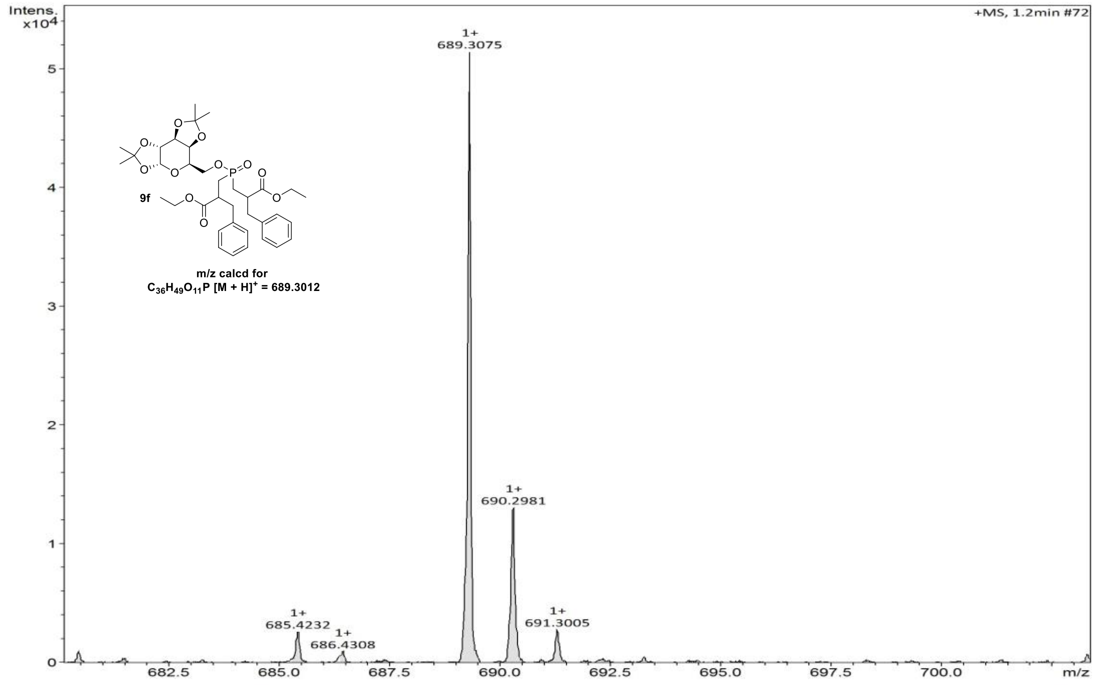

*Figure S67. MS spectrum of compound **9f***

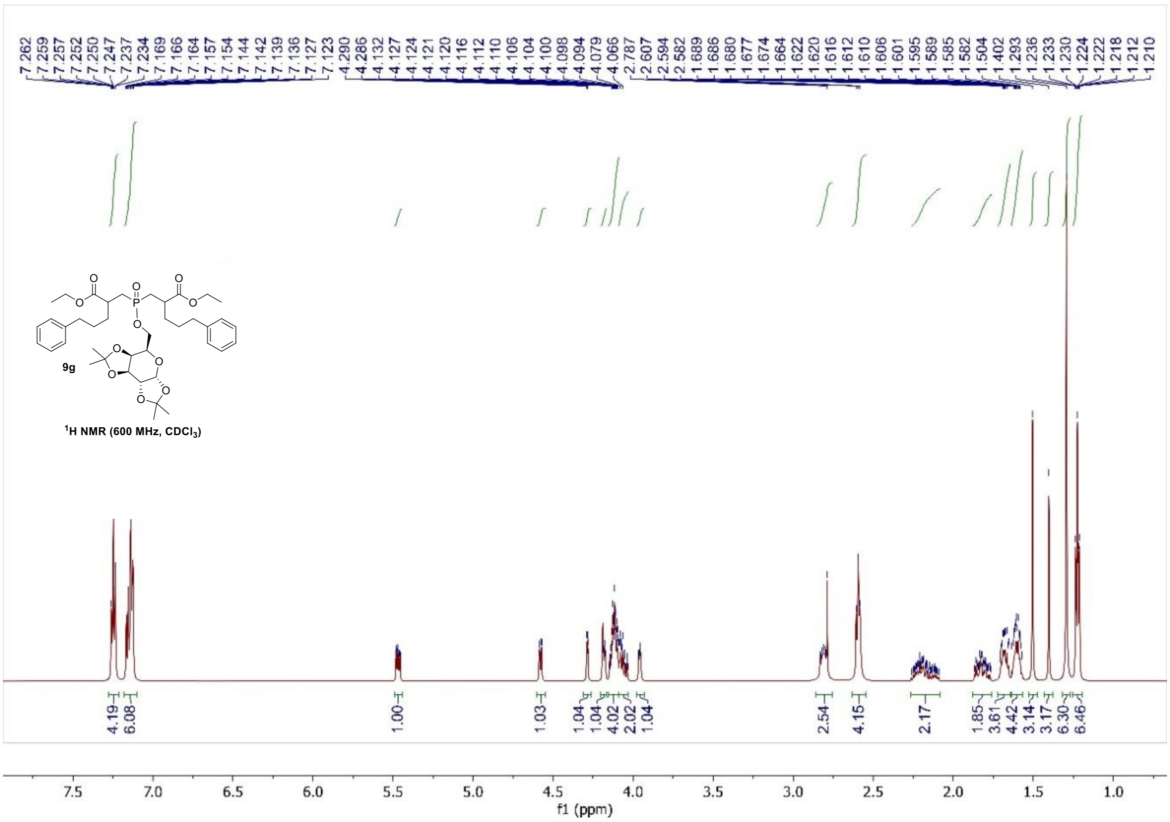

*Figure S68. ^{1}HNMR of compound **9g***

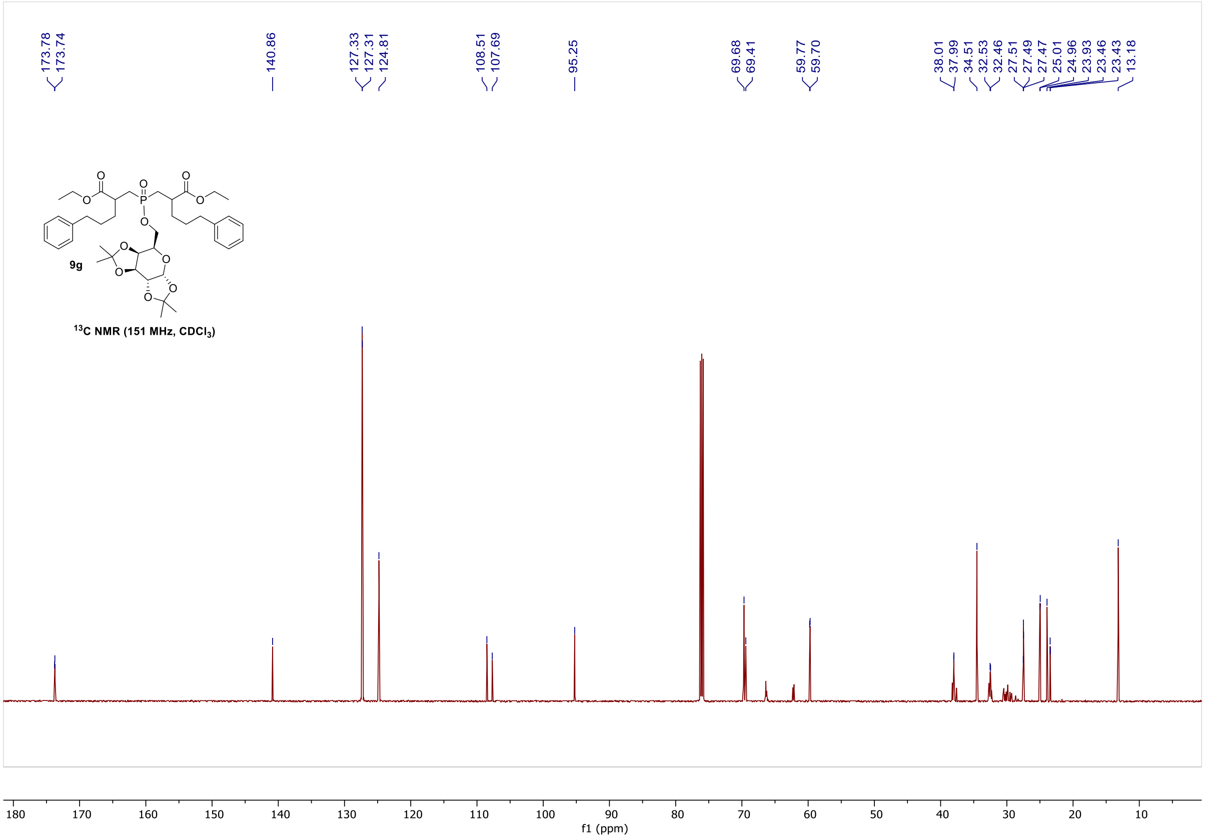

*Figure S69. ^{13}CNMR of compound **9g***

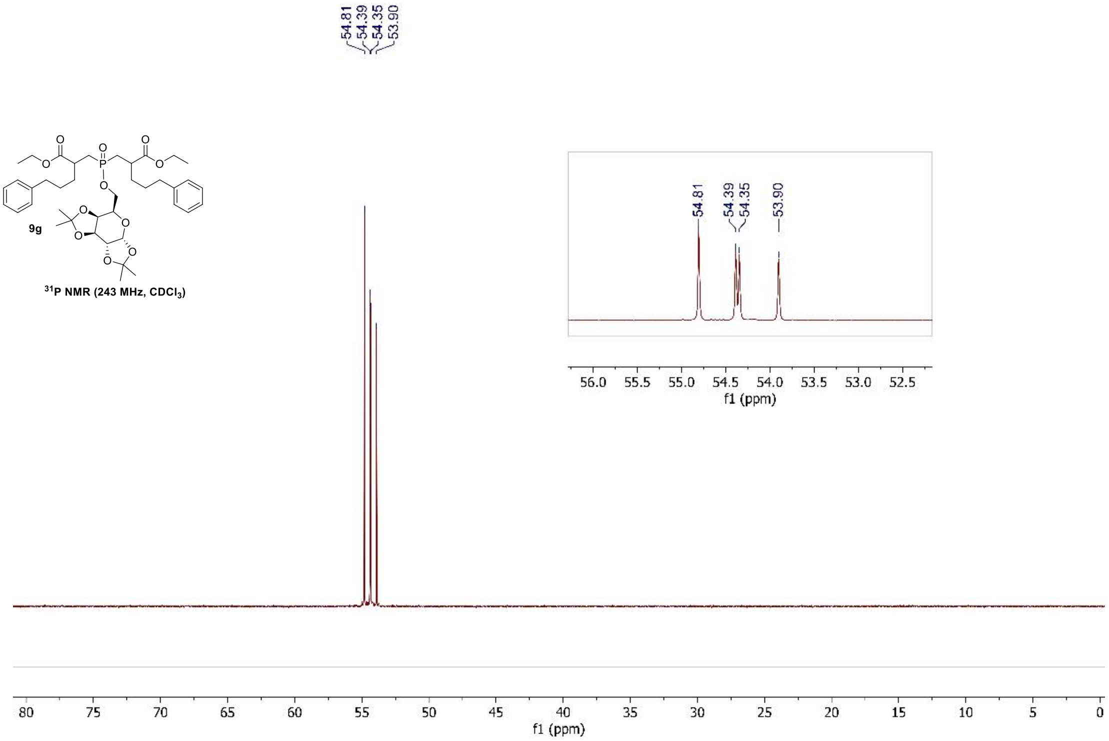

*Figure S70. ^{31}PNMR of compound **9g***

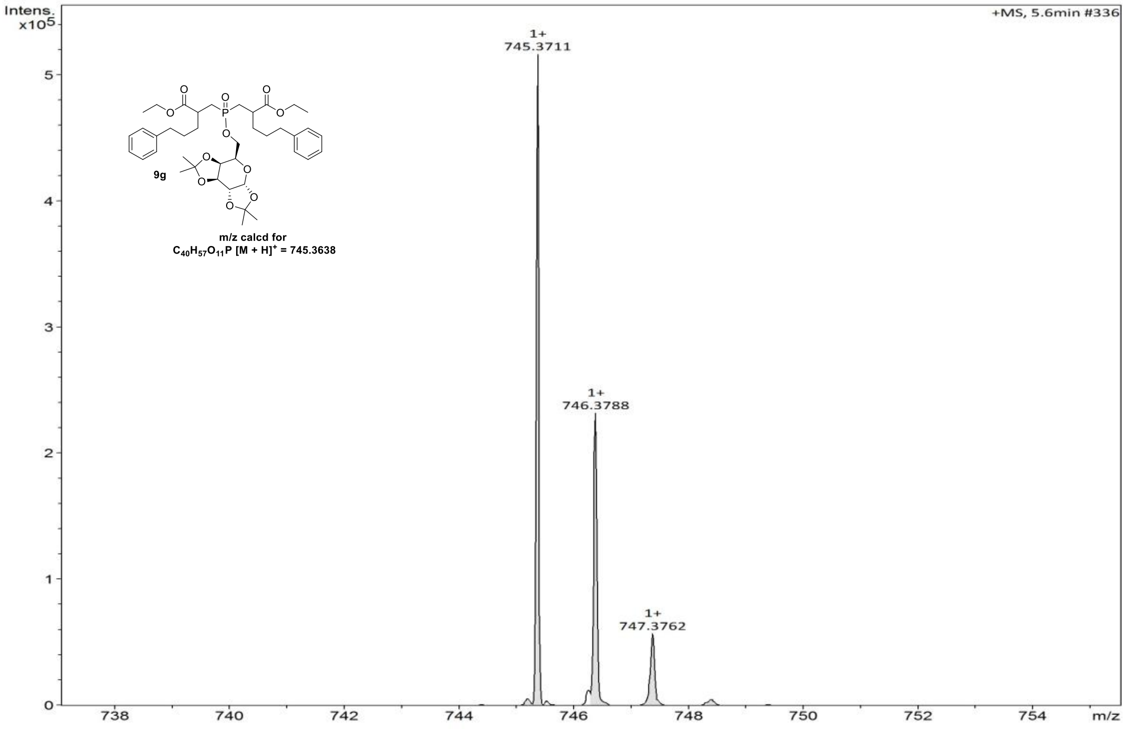

Figure S71. MS spectrum of compound ***9g***

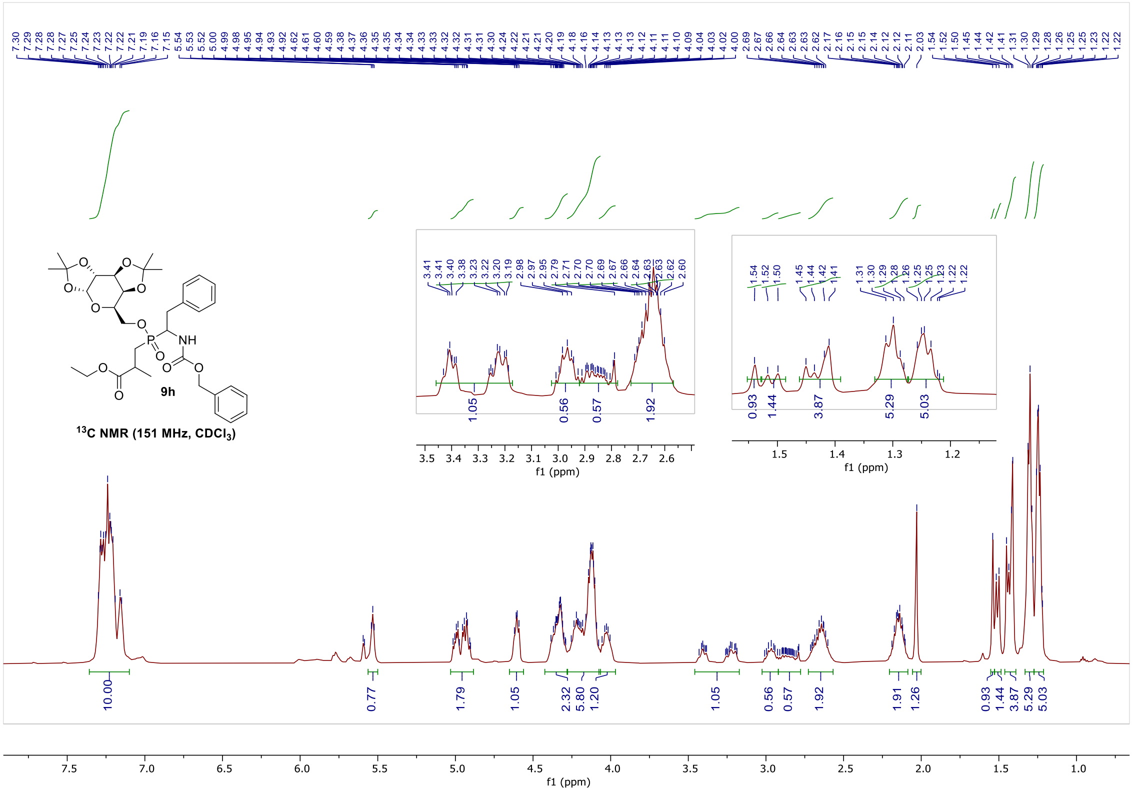

Figure S72. *^{1}HNMR of compound* ***9h***

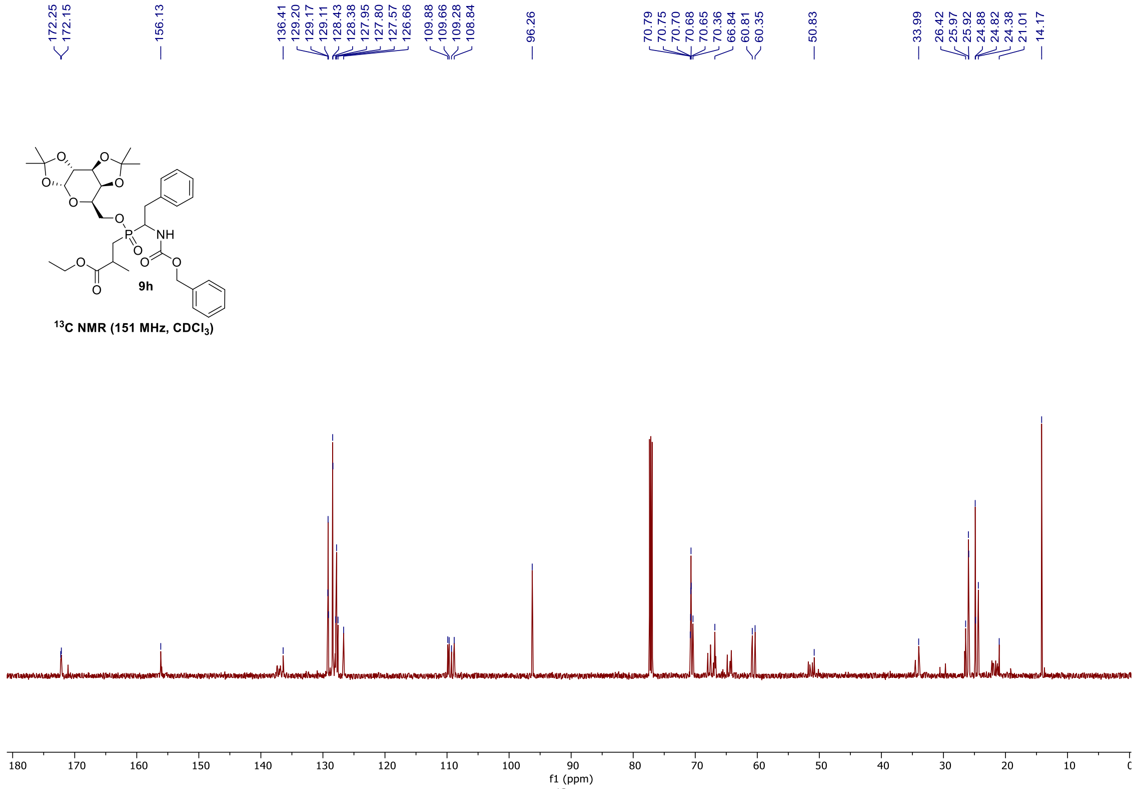

*Figure S73. ^{13}CNMR of compound **9h***

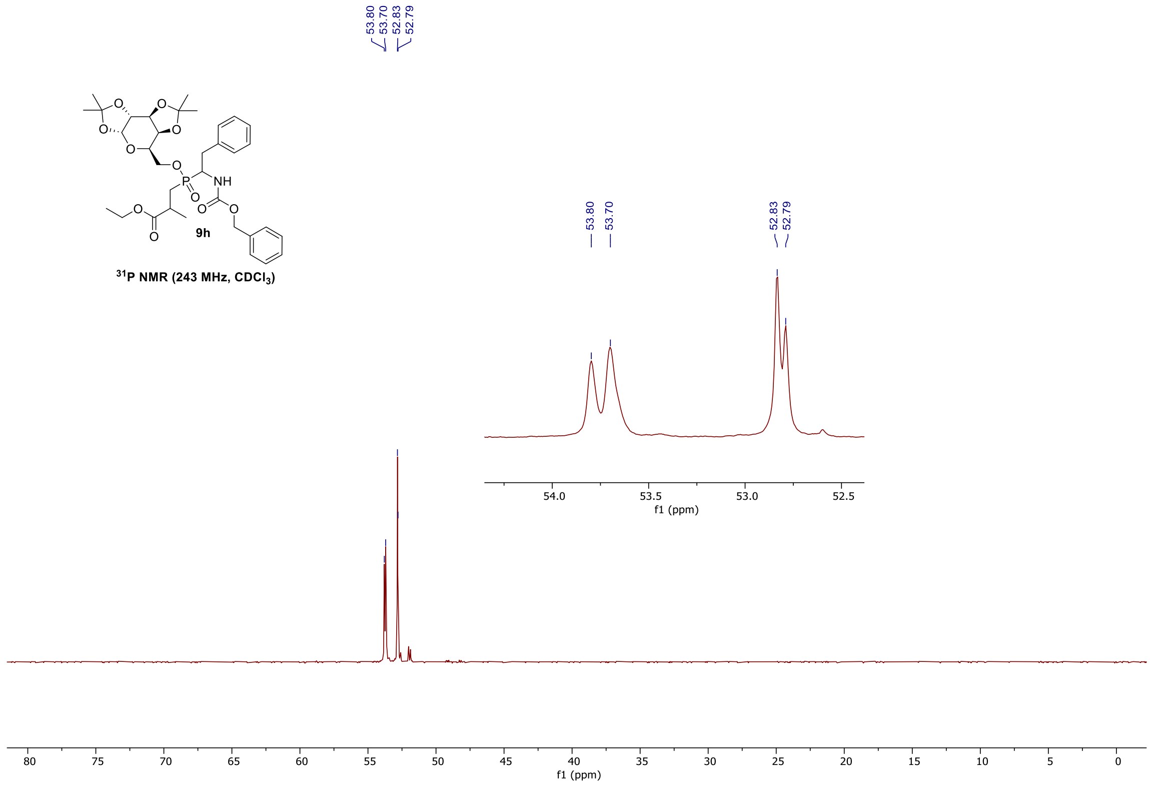

*Figure S74. ^{31}PNMR of compound **9h***

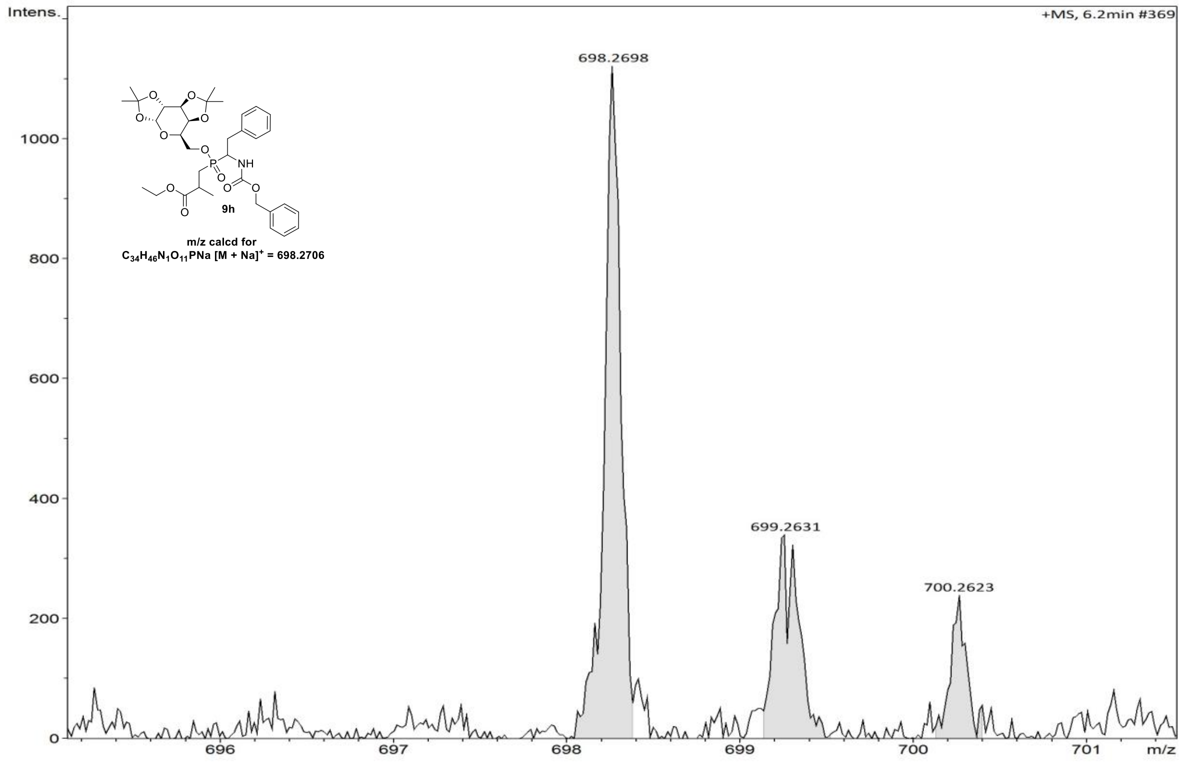

Figure S75. MS spectrum of compound ***9h***

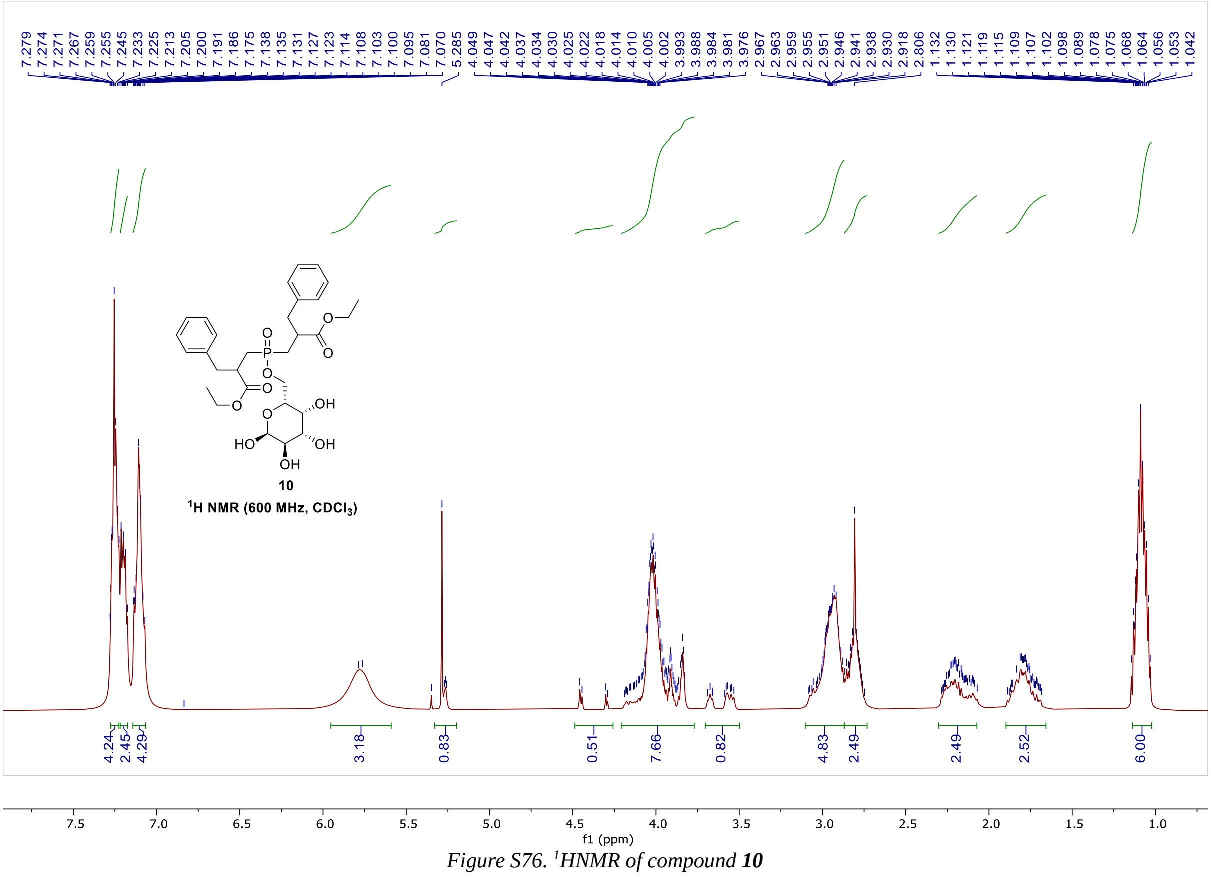

Figure S76. ^{1}HNMR of compound ***10***

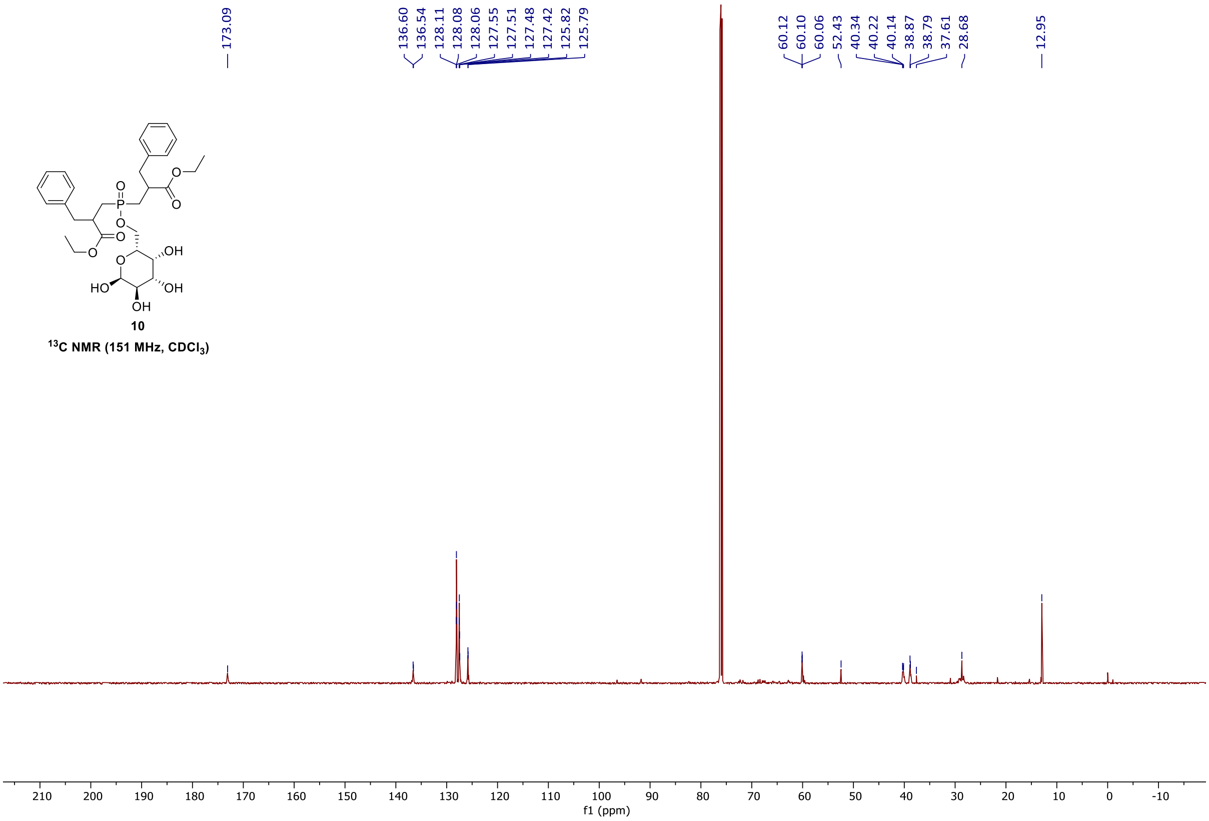

Figure S77. ^{13}CNMR of compound **10**

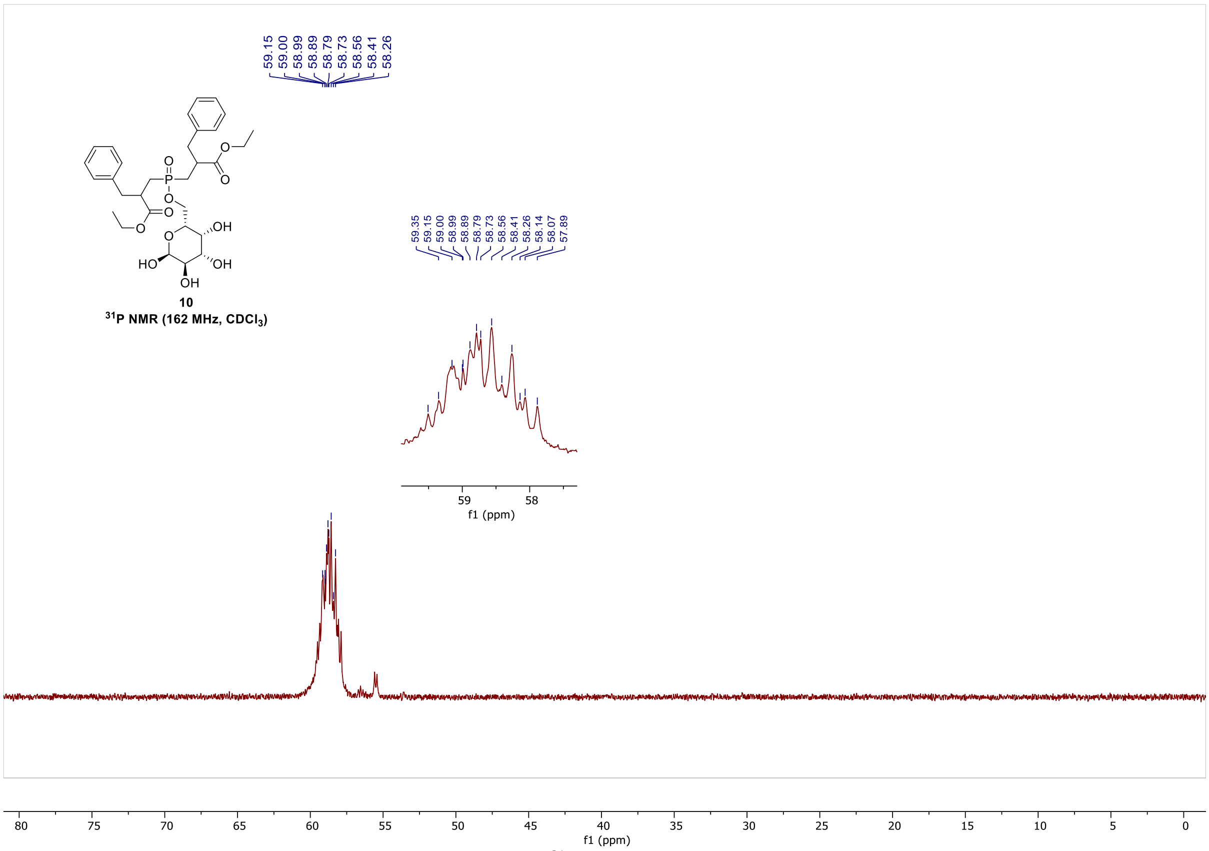

Figure S78. ^{31}PNMR of compound **10**

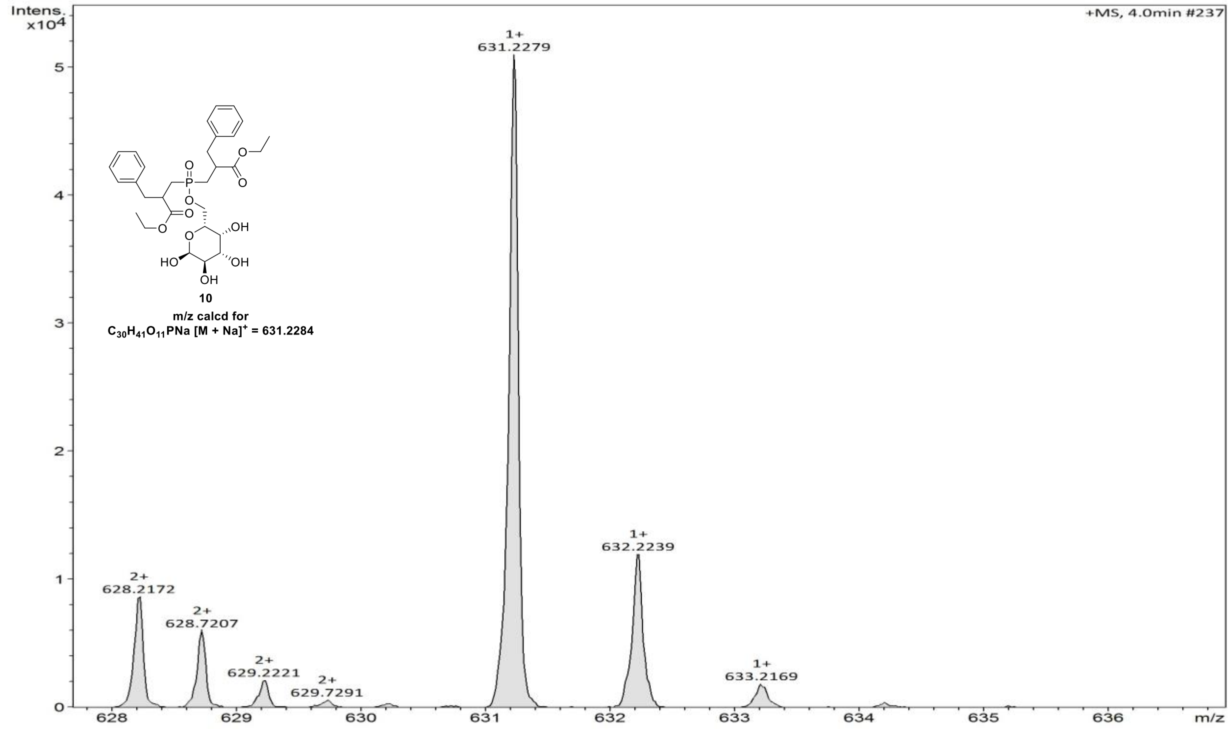

Figure S79. MS spectrum of compound ***10***

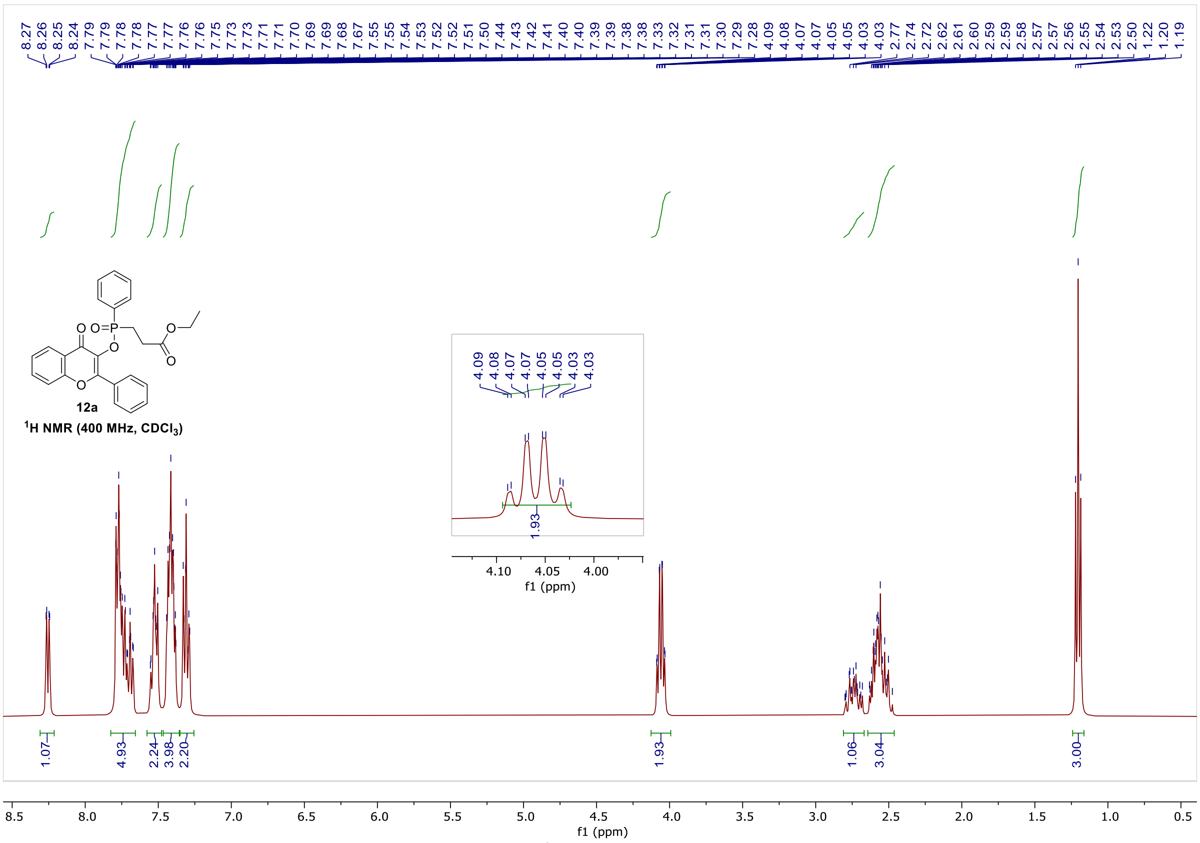

Figure S80. ^{1}HNMR of compound ***12a***

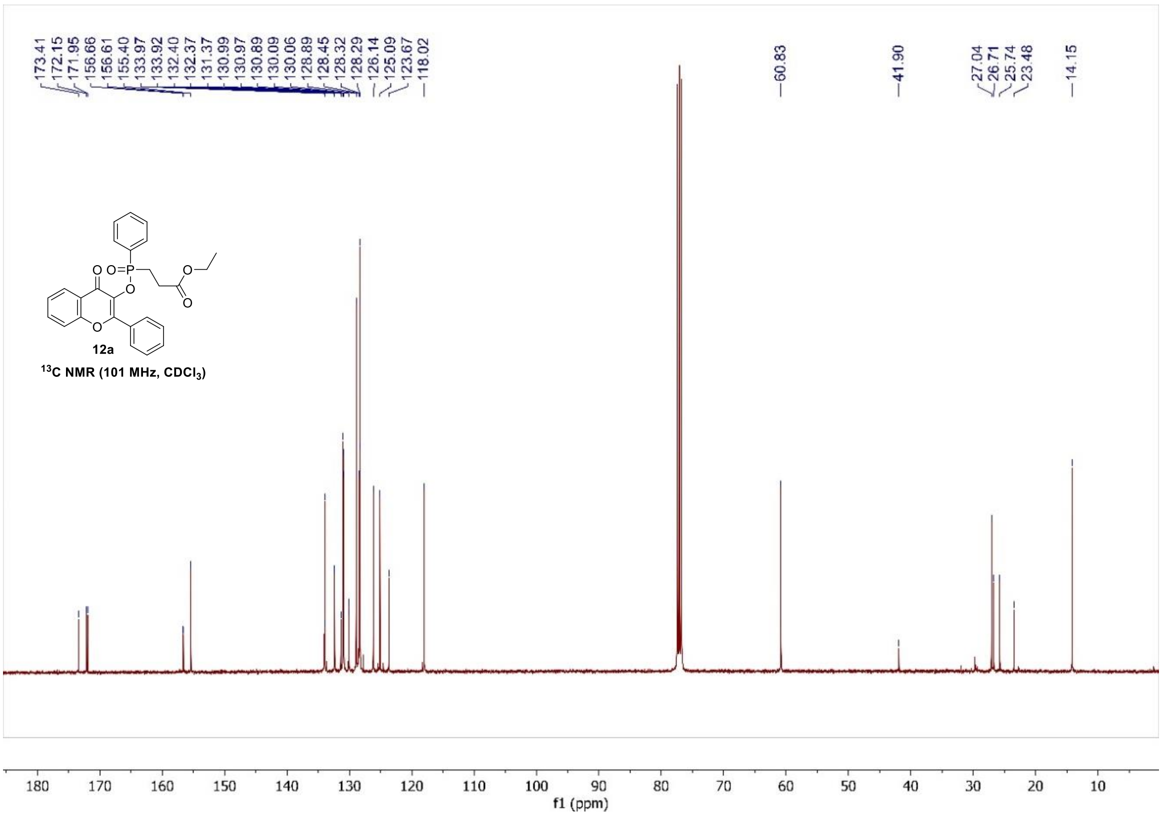

*Figure S81. ^{13}CNMR of compound **12a***

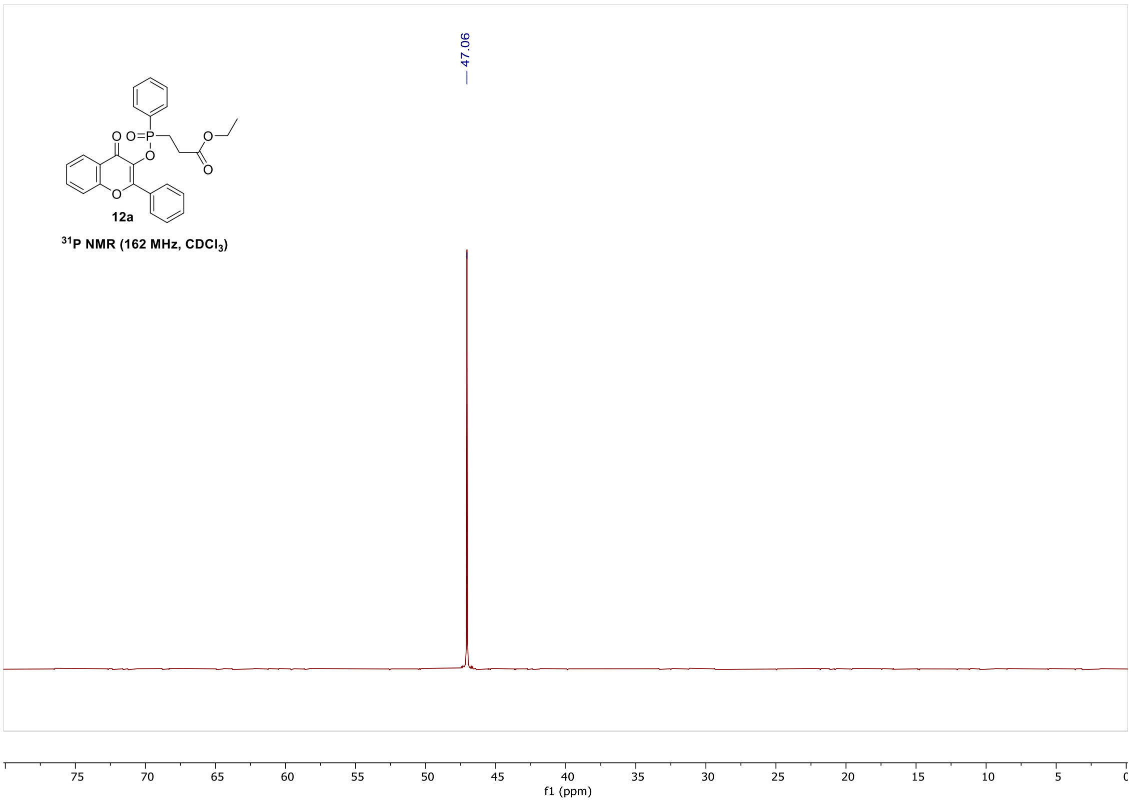

*Figure S82. ^{31}PNMR of compound **12a***

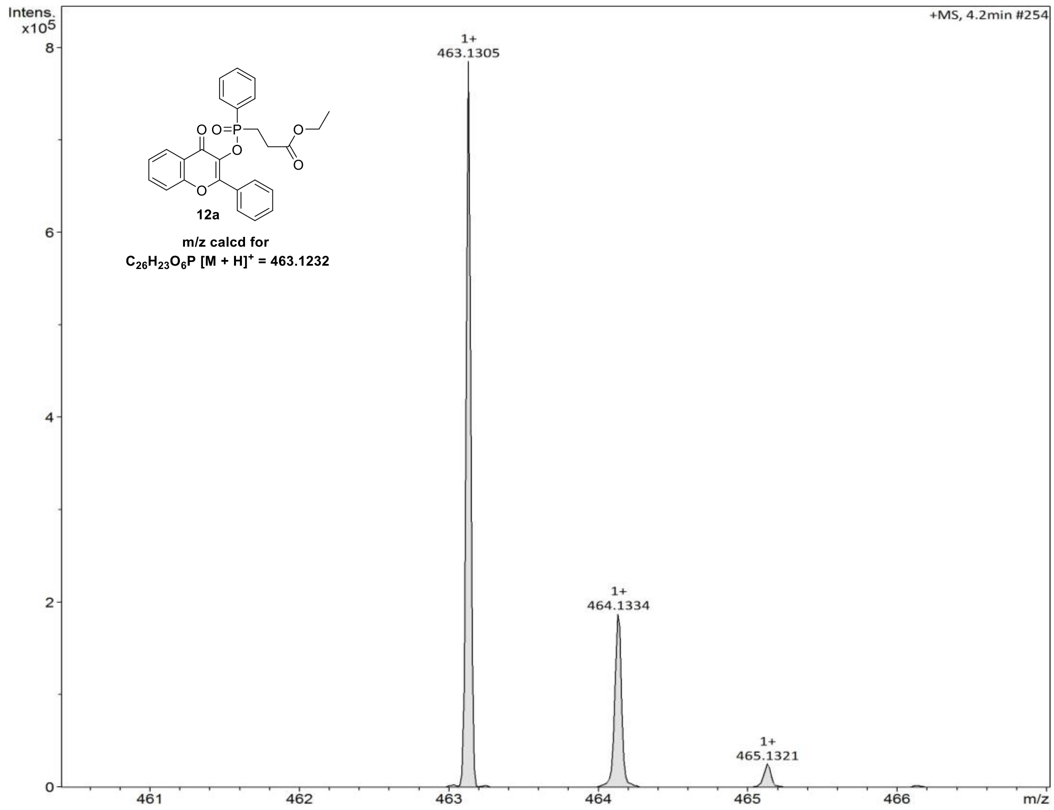

*Figure S83. MS spectrum of compound **12a***

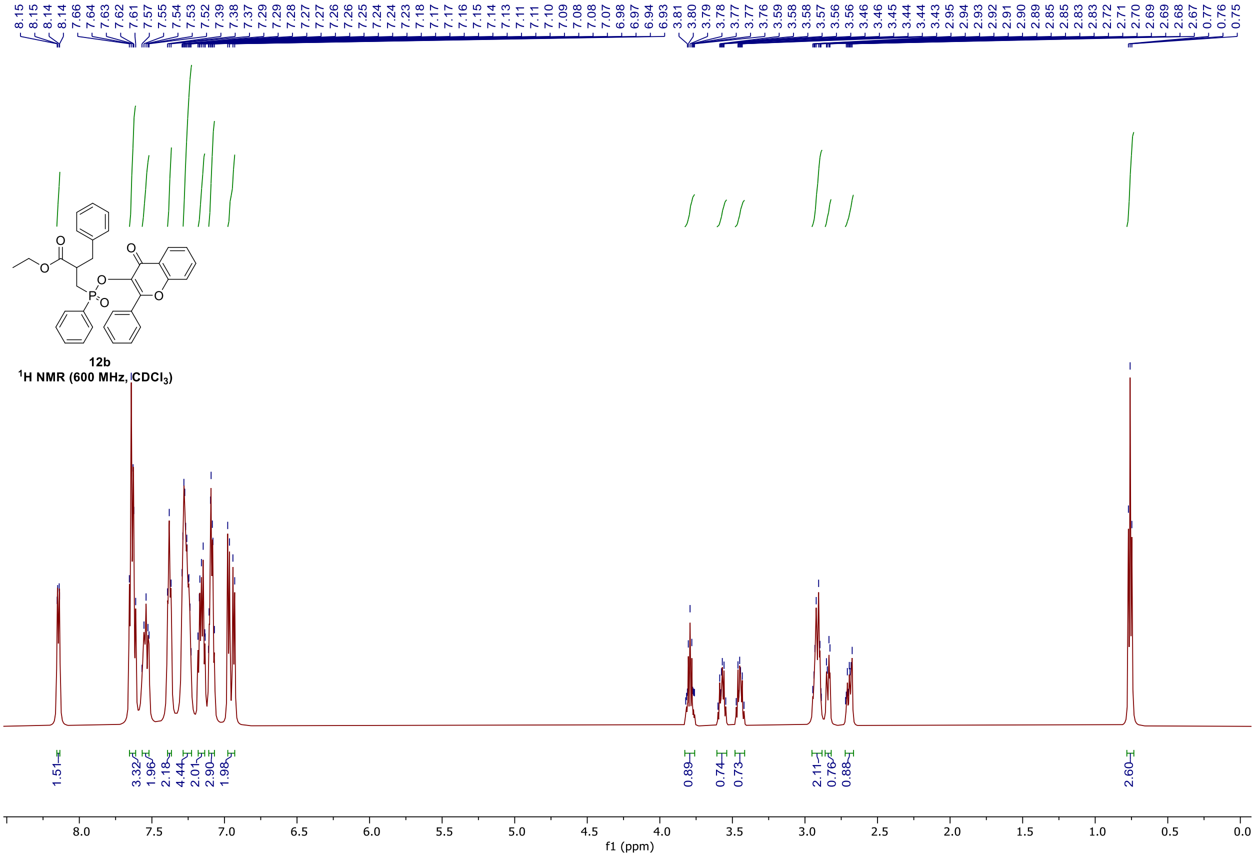

*Figure S84. ^{1}HNMR of compound **12b***

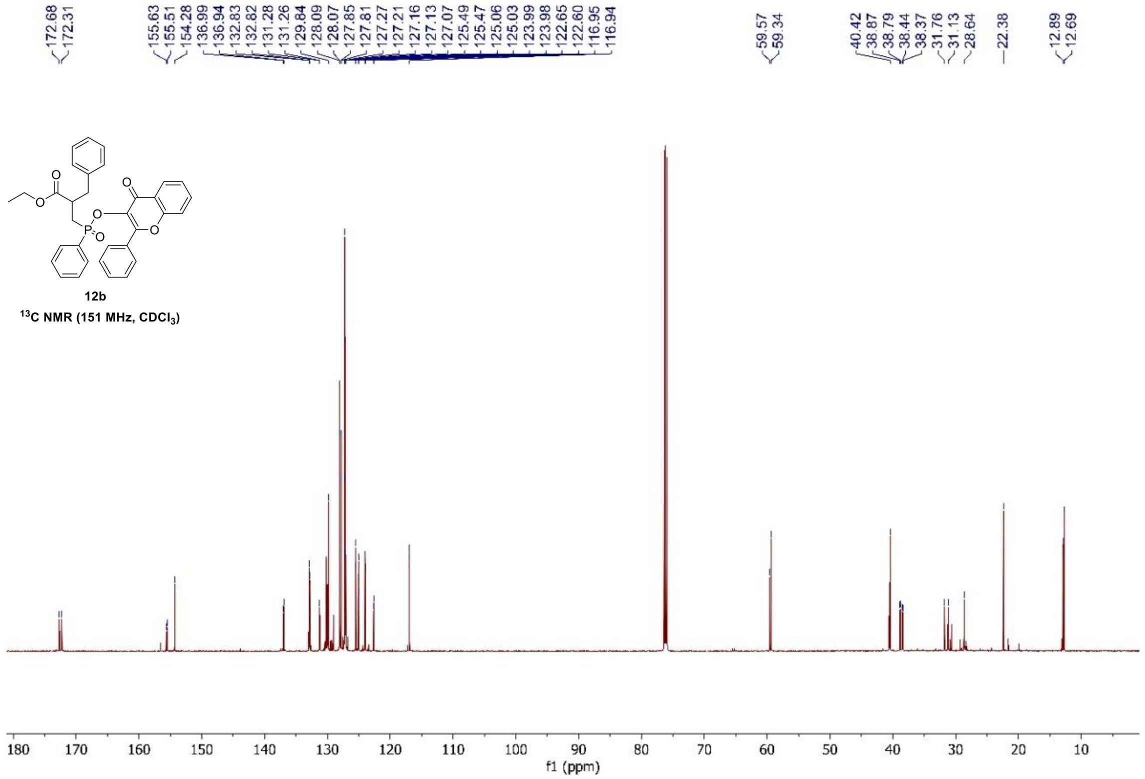

Figure S85. ^{13}CNMR of compound ***12b***

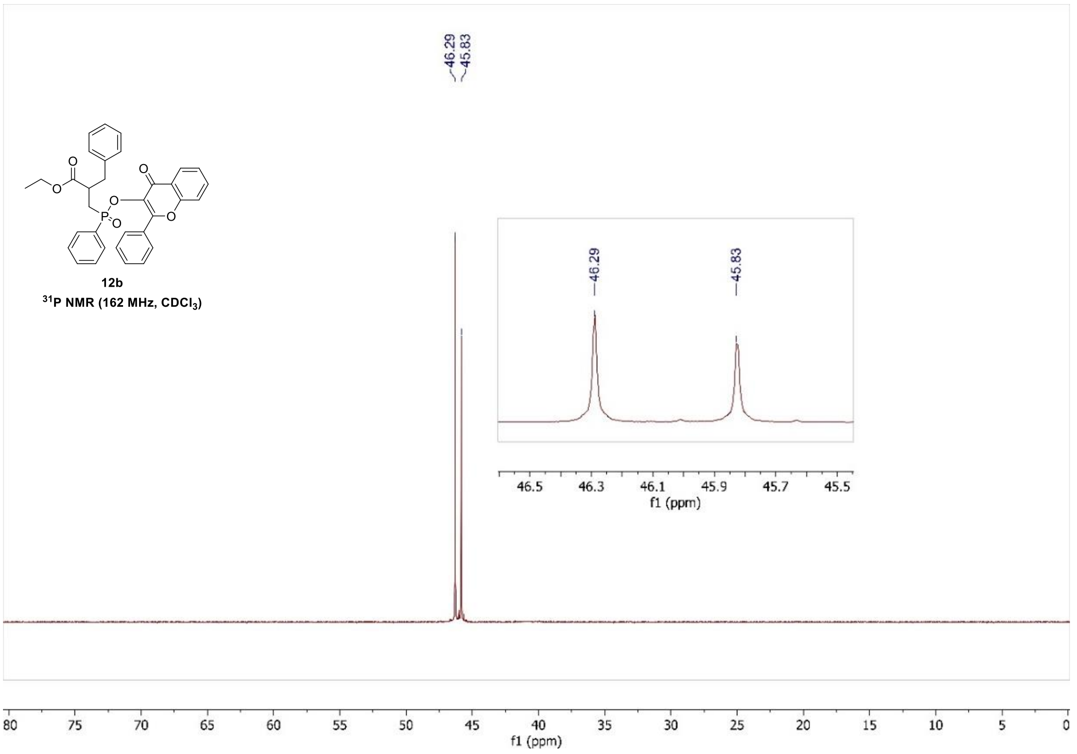

Figure S86. ^{31}PNMR of compound ***12b***

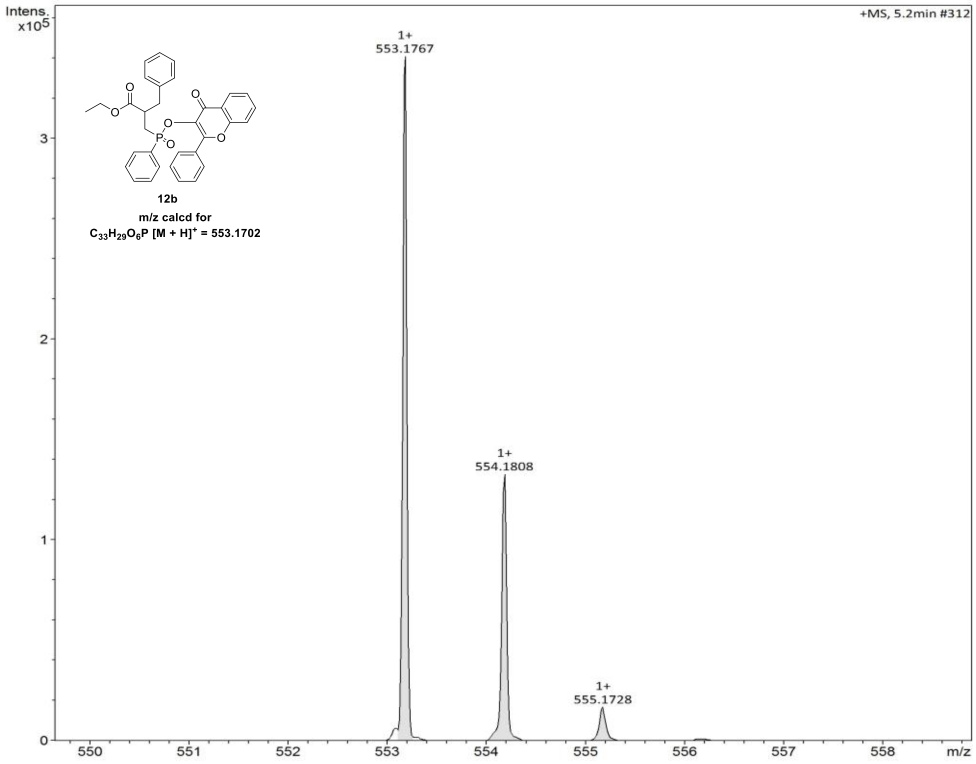

*Figure S87. MS spectrum of compound **12b***

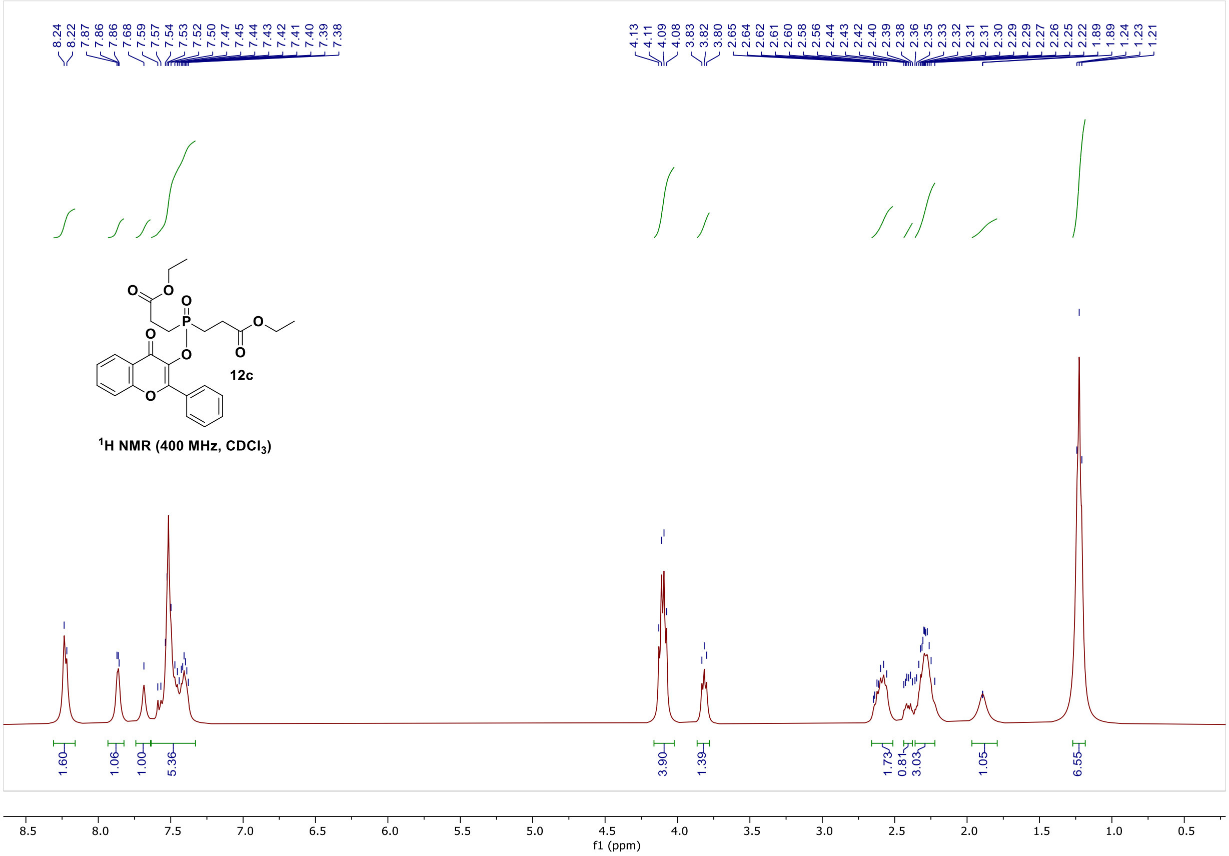

*Figure S88. ^{1}HNMR of compound **12c***

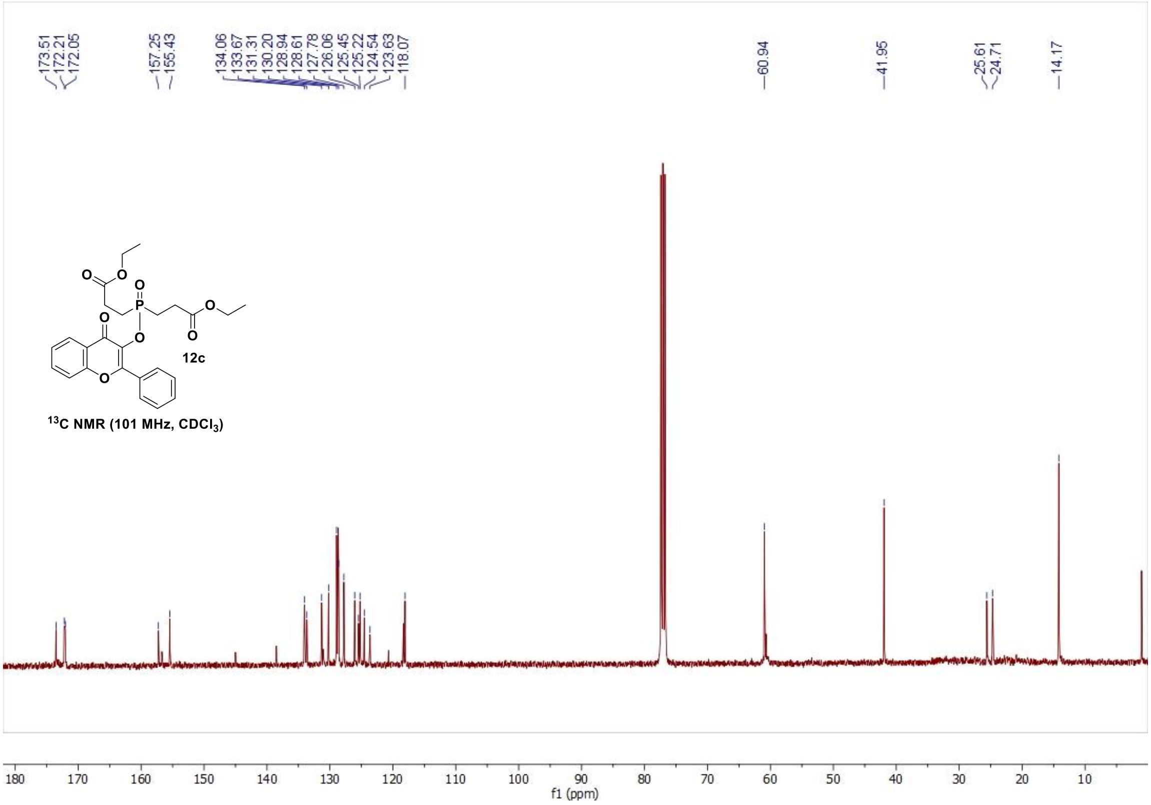

*Figure S89. ^{13}CNMR of compound **12c***

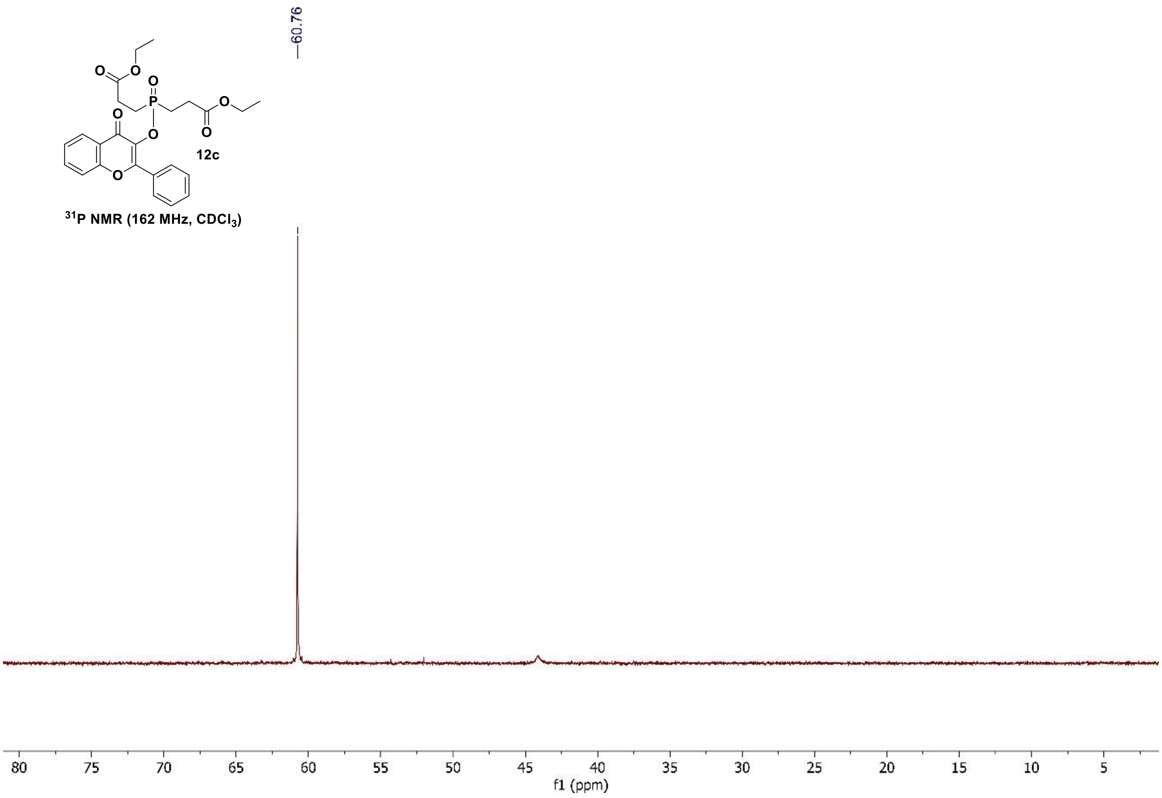

*Figure S90. ^{31}PNMR of compound **12c***

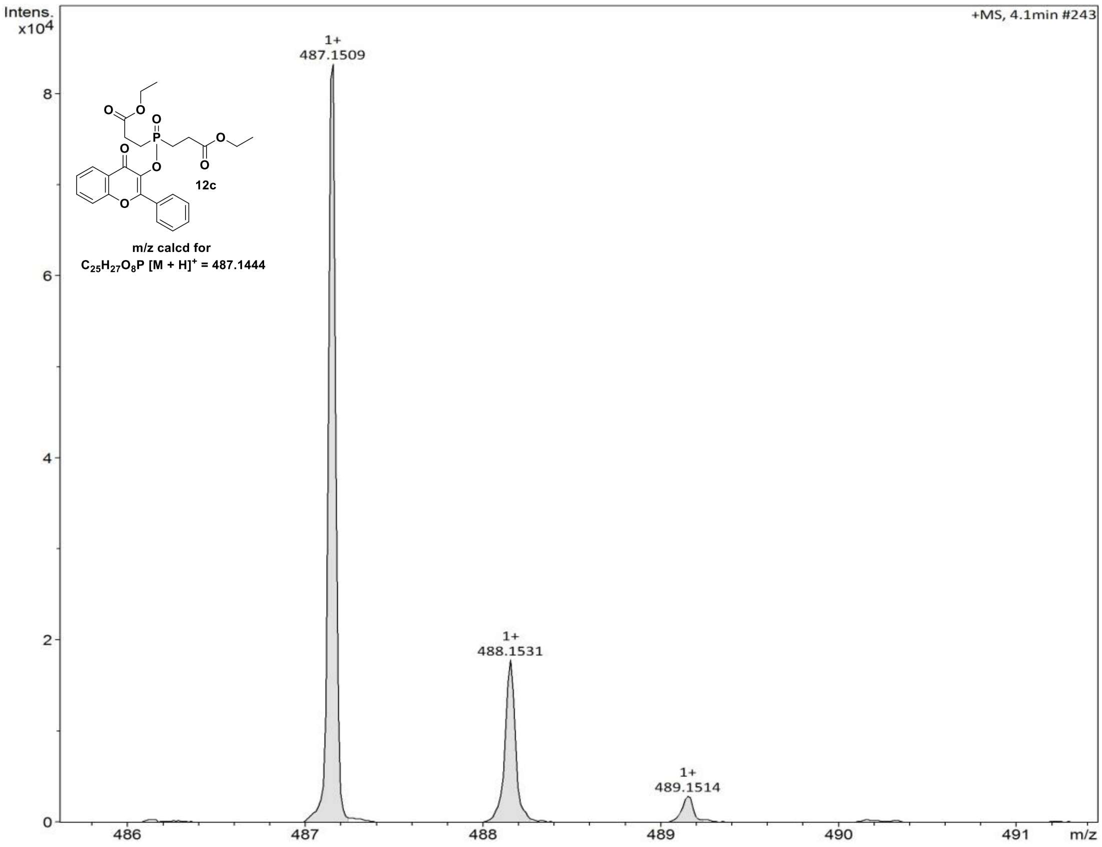

Figure S91. MS spectrum of compound ***12c***

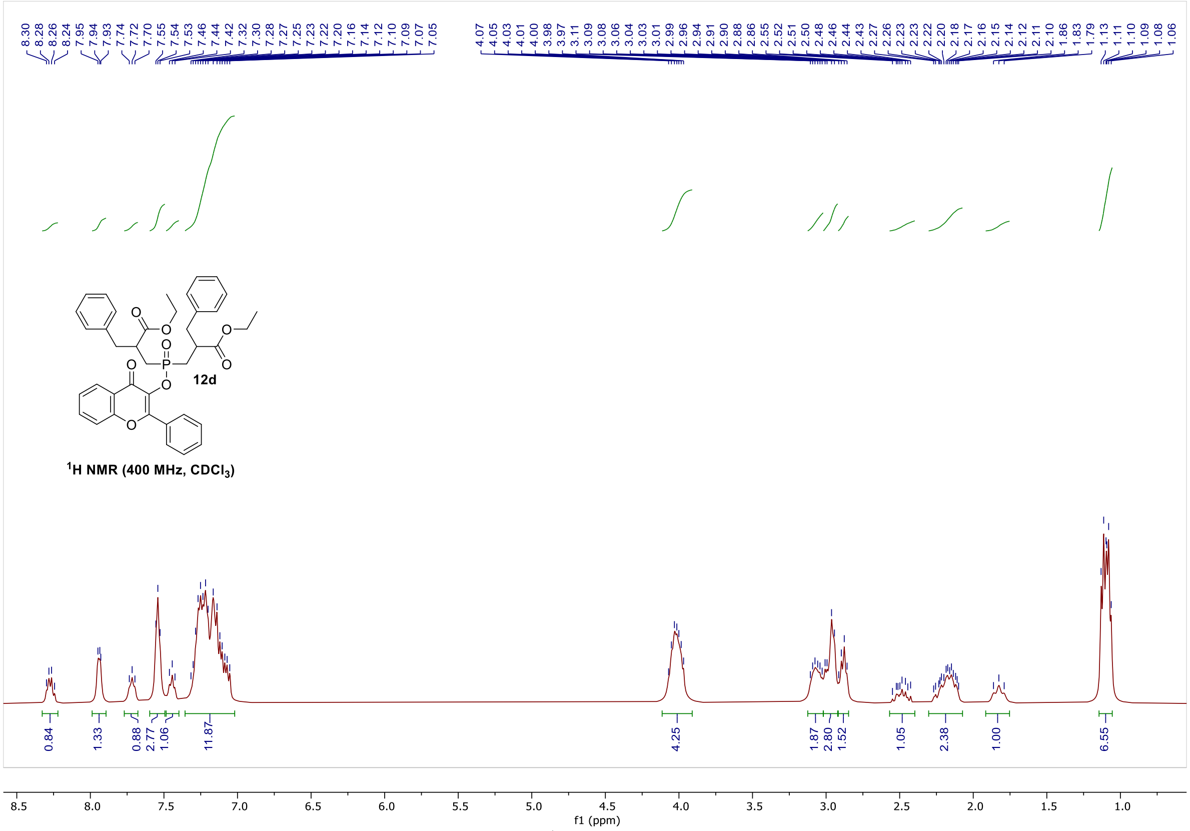

Figure S92. [1]*HNMR of compound* ***12d***

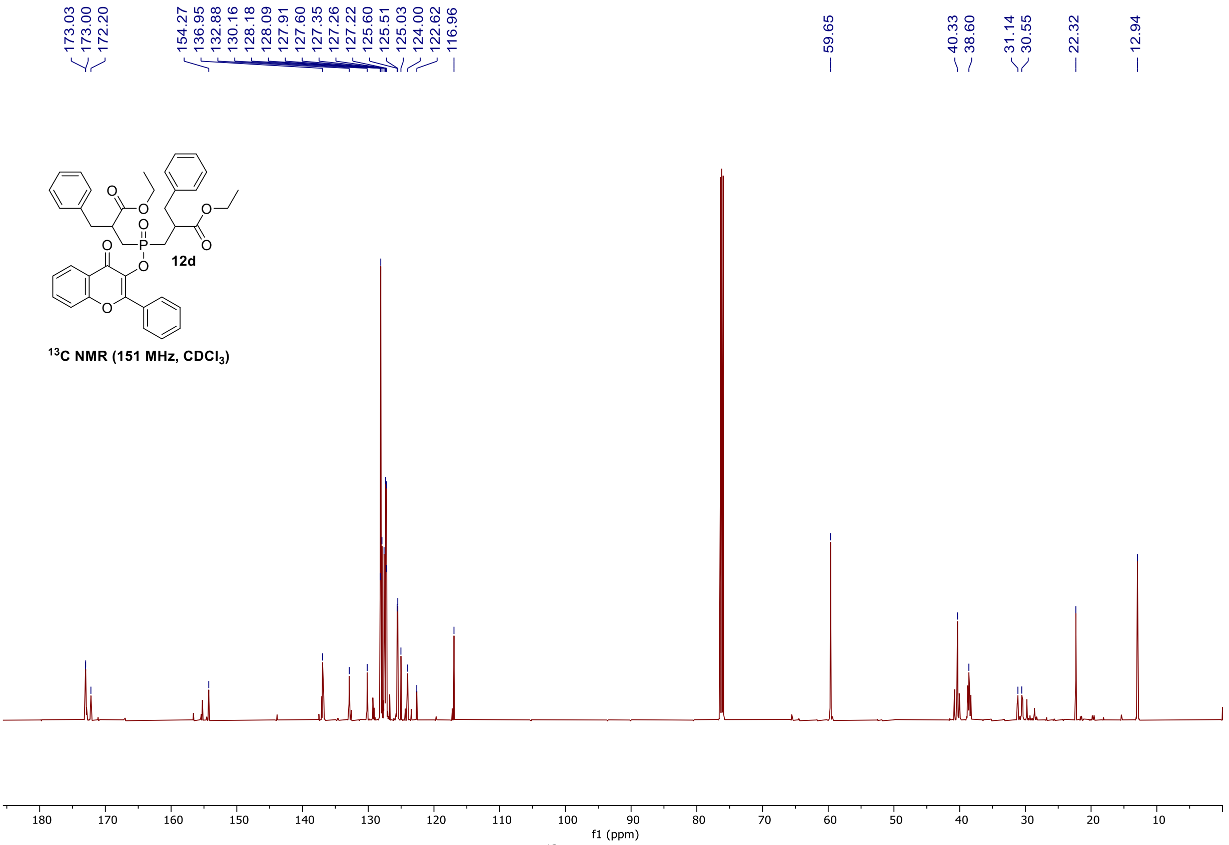

*Figure S93. ^{13}CNMR of compound **12d***

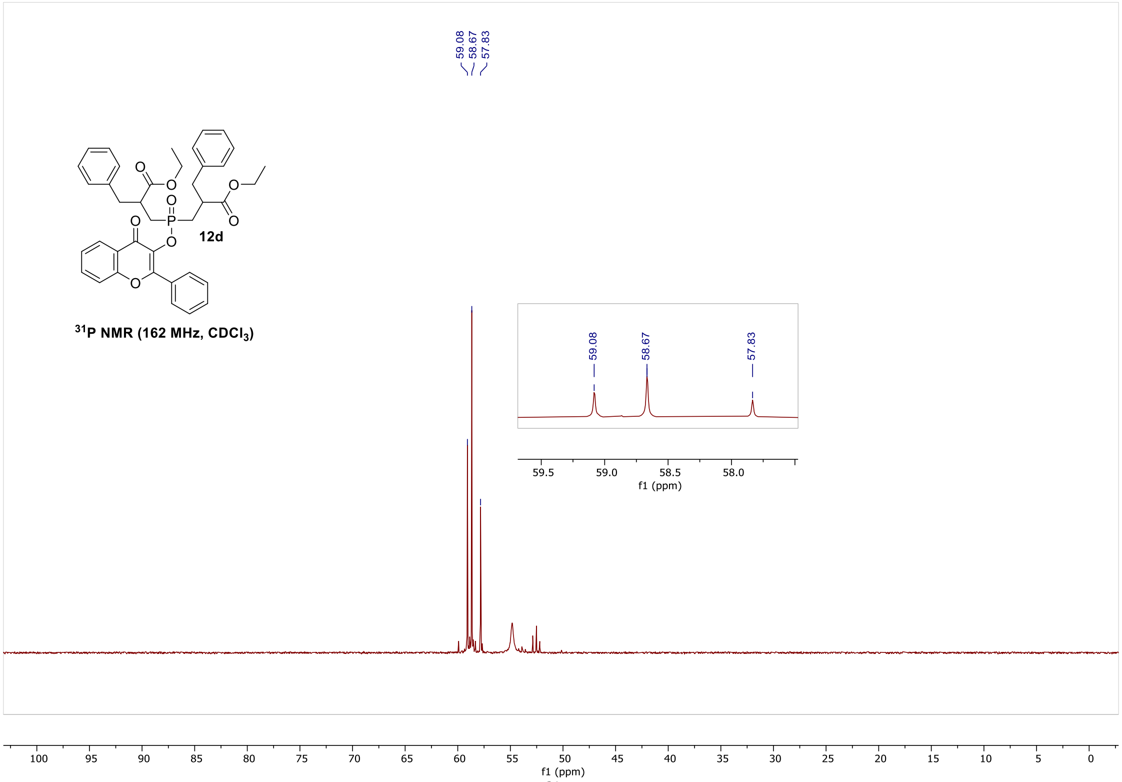

*Figure S94. ^{31}PNMR of compound **12d***

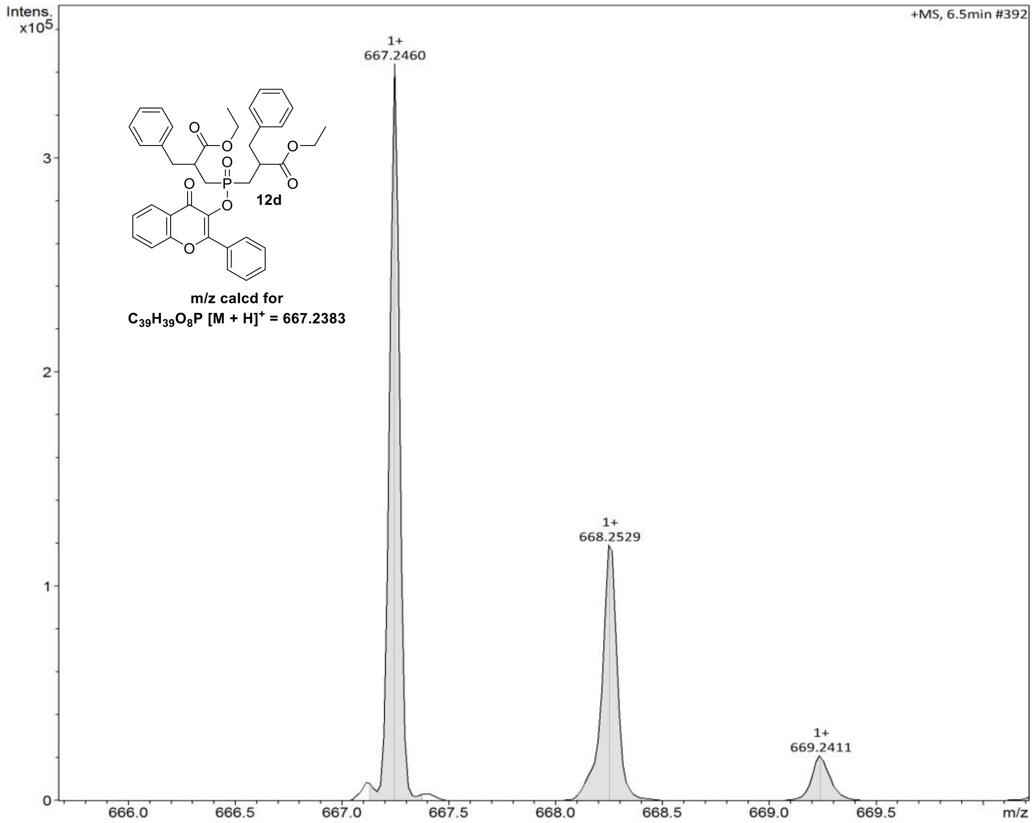

Figure S95. MS spectrum of compound ***12d***

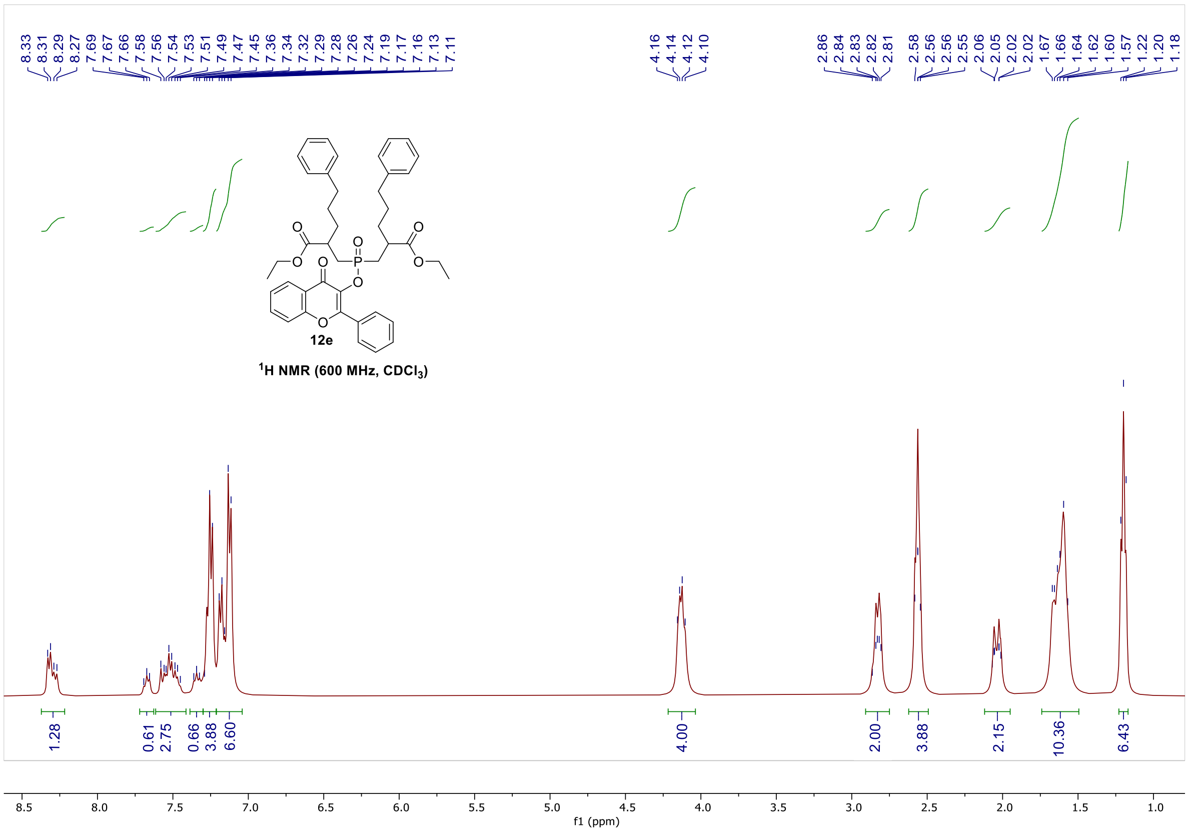

Figure S96. ^{1}*HNMR of compound* ***12e***

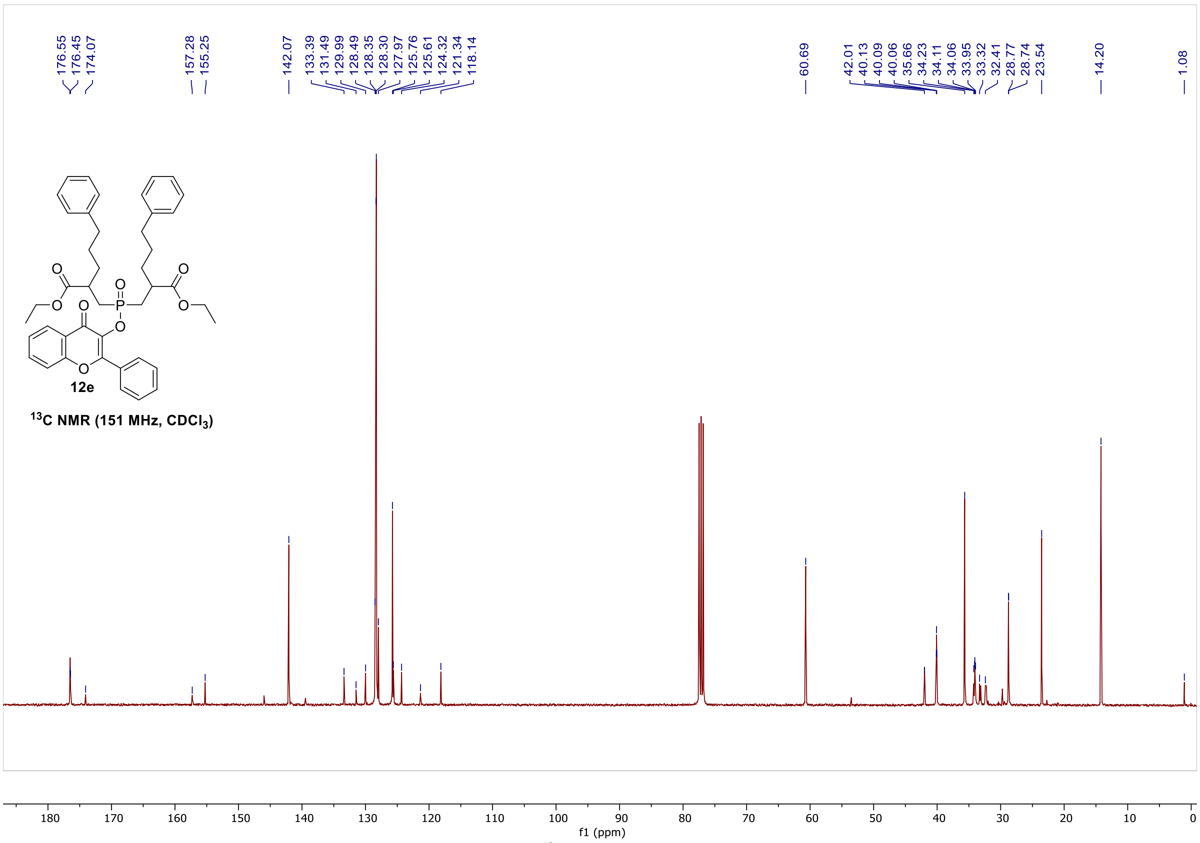

Figure S97. ^{13}CNMR of compound **12e**

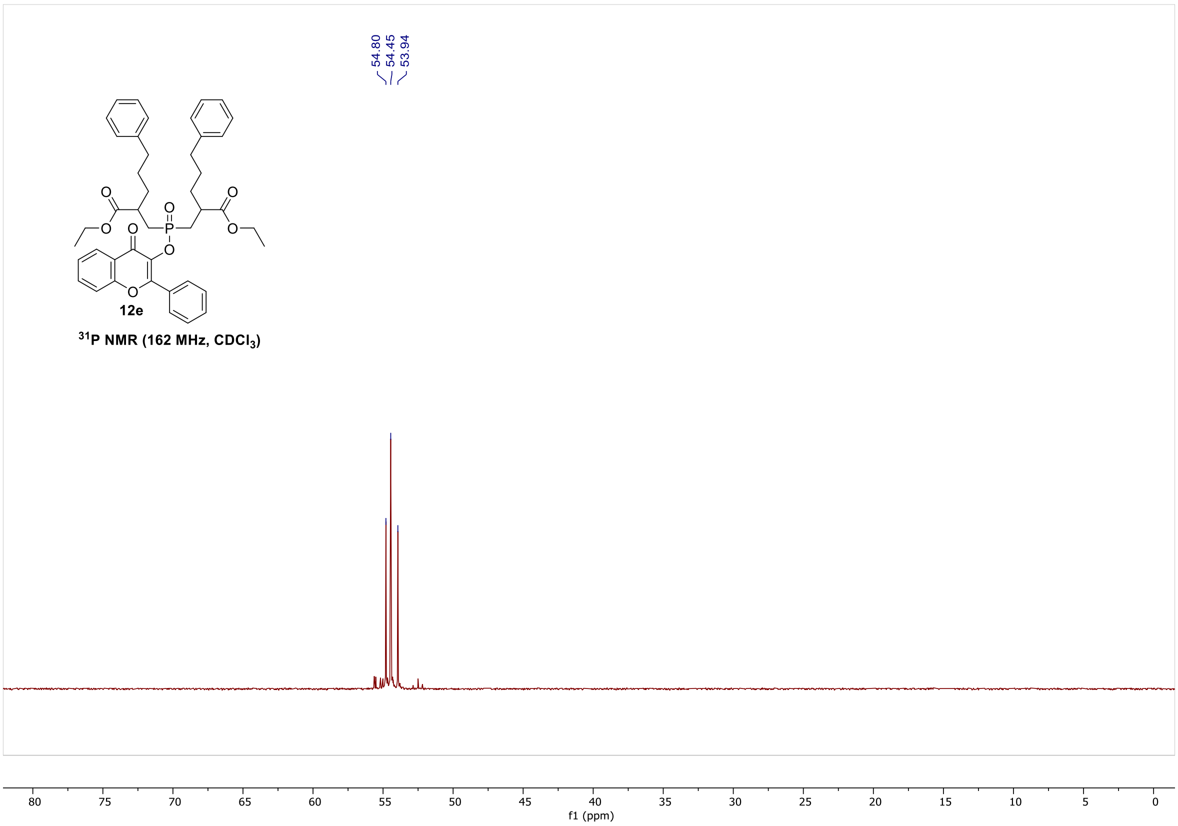

Figure S98. ^{31}PNMR of compound **12e**

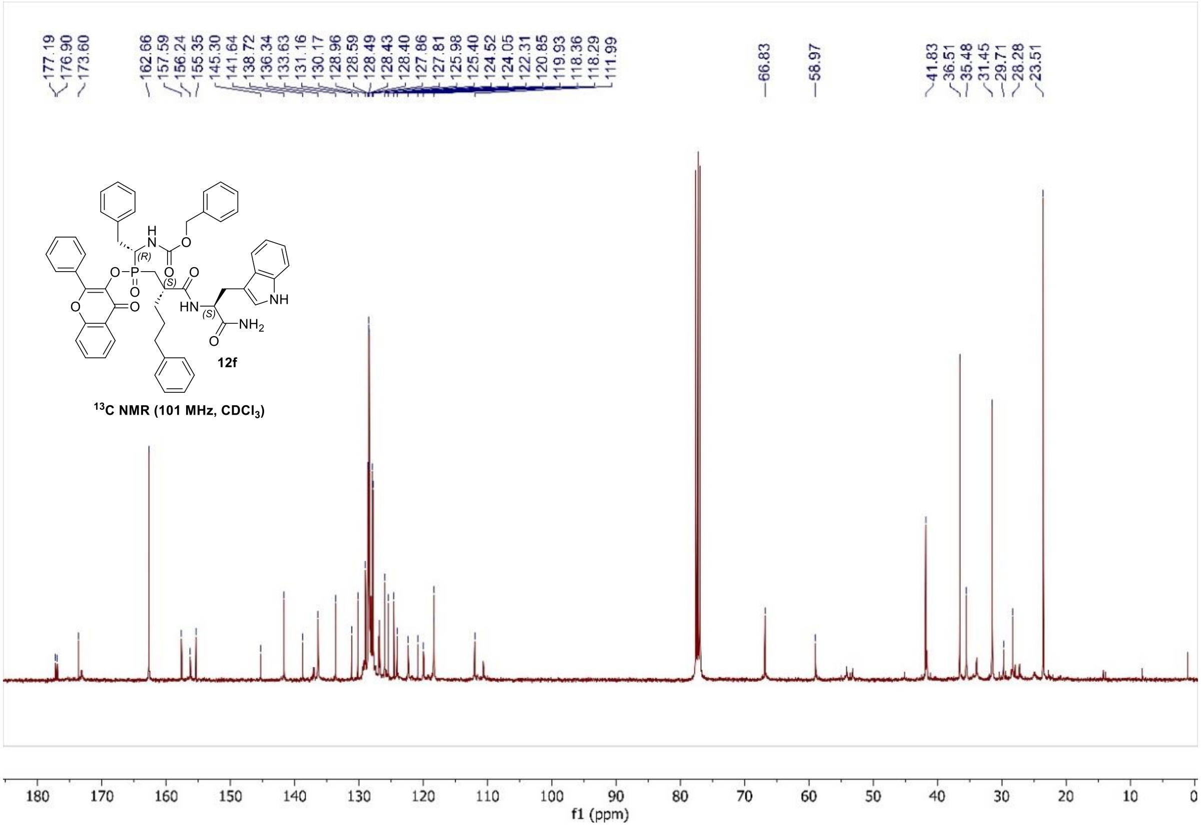

*Figure S99. ^{13}CNMR of compound **12f***

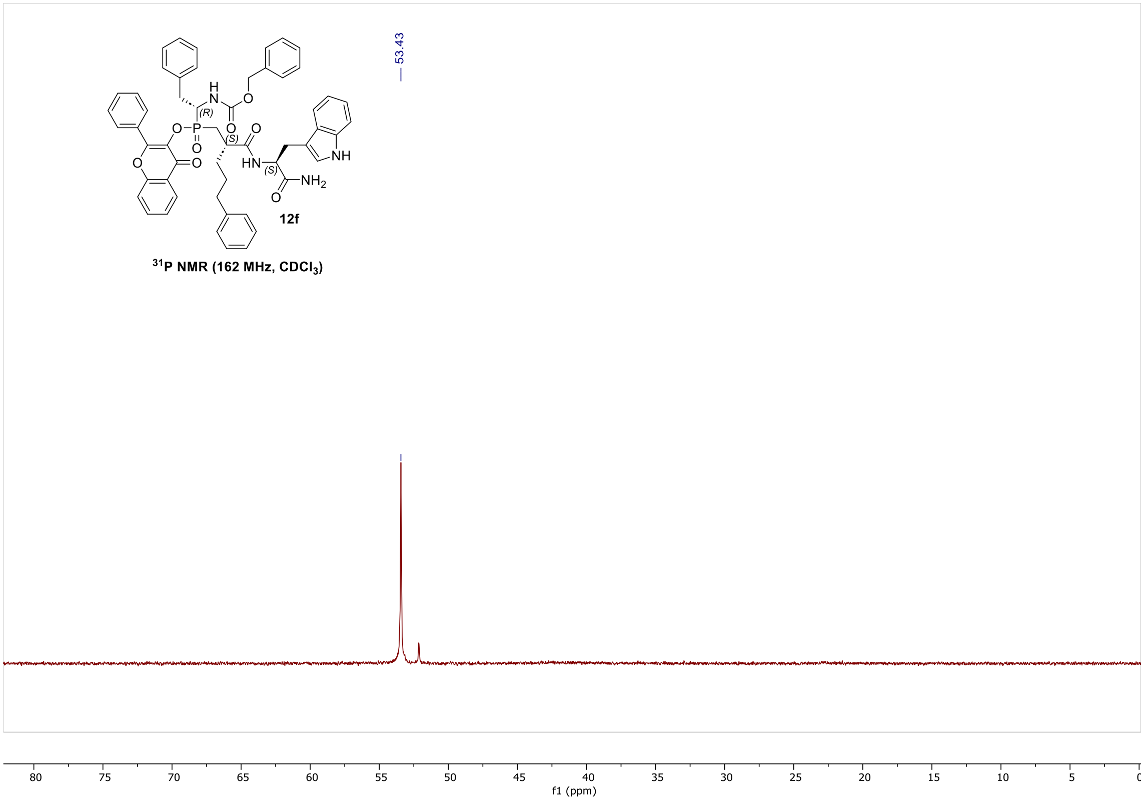

*Figure S100. ^{31}PNMR of compound **12f***

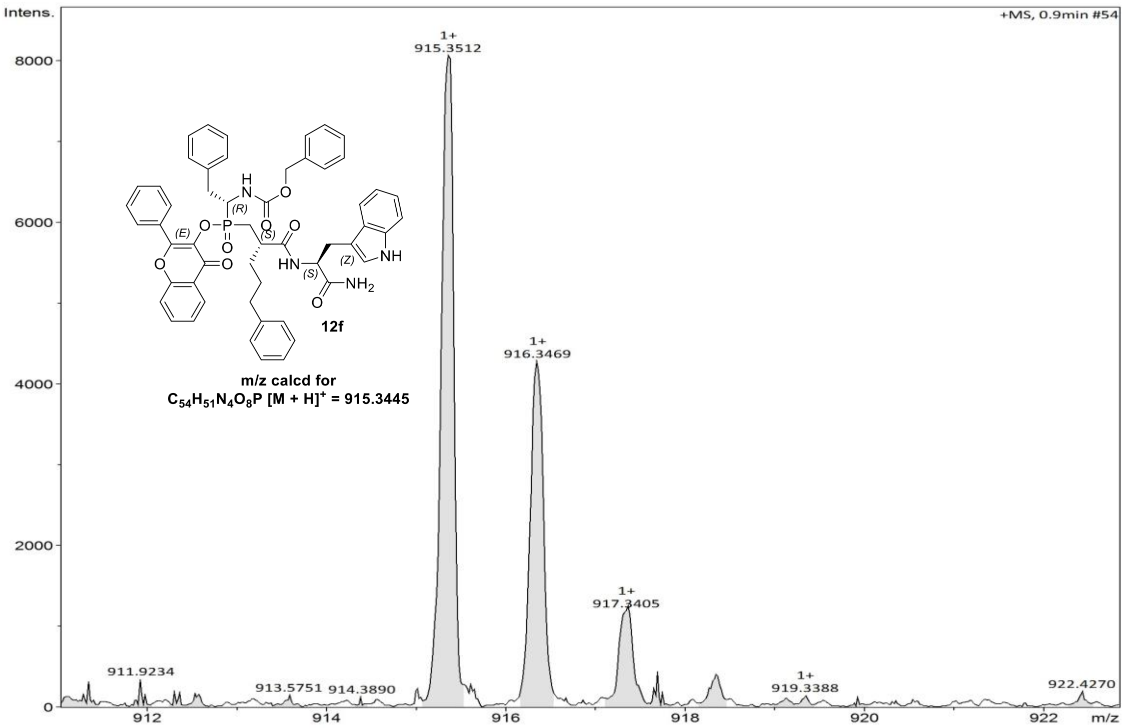

Figure S101. MS spectrum of compound ***12f***
